# Potent inhibitors of the human RNA ligase Rlig1 highlights its role in RNA integrity maintenance under oxidative cellular stress†

**DOI:** 10.1039/d4sc06542e

**Published:** 2025-01-18

**Authors:** Lisa A. Schlor, Maya Peußner, Silke Müller, Andreas Marx

**Affiliations:** a Department of Chemistry, University of Konstanz Universitätsstraße 10 78457 Konstanz Germany andreas.marx@uni-konstanz.de; b Konstanz Research School Chemical Biology, University of Konstanz Universitätsstraße 10 78457 Konstanz Germany; c Department of Biology, University of Konstanz Universitätsstraße 10 78457 Konstanz Germany; d Screening Center, University of Konstanz Universitätsstraße 10 78457 Konstanz Germany

## Abstract

Human RNA ligase 1 (Rlig1) catalyzes the ligation of 5′-phosphate to 3′-hydroxyl ends *via* a conserved three-step mechanism. Rlig1-deficient HEK293 cells exhibit reduced cell viability and RNA integrity under oxidative stress, suggesting Rlig1's role in RNA repair maintenance. Reactive oxygen species (ROS) are linked to various diseases, including neurodegenerative disorders and cancer, where RNA damage has significant effects. This study identifies and characterizes Rlig1 inhibitors to elucidate its role in RNA metabolism. We developed a fluorescence resonance energy transfer (FRET)-based assay to monitor RNA ligation and screened a library of 13 026 bioactive small molecules. SGI-1027 emerged as a promising lead compound, and structure–activity relationship (SAR) studies revealed that the terminal residues play a key role in its inhibitory effect. In total 22 SGI-1027 derivatives were synthesized and tested, providing insights into the structural requirements for effective Rlig1 inhibition. Three derivatives showed low micromolar IC_50_ values and minimal cytotoxicity in HEK293 cells under physiological conditions. The combination of Rlig1 inhibition and oxidative stress led to reduced cell viability and compromised RNA integrity, reinforcing Rlig1's role in RNA maintenance. These findings provide a foundation for developing novel therapeutics aimed at targeting RNA maintenance pathways in conditions of dysregulated ROS levels.

## Abstract 2

We report on the development of inhibitors of human RNA ligase 1 (Rlig1).

## Graphical abstract

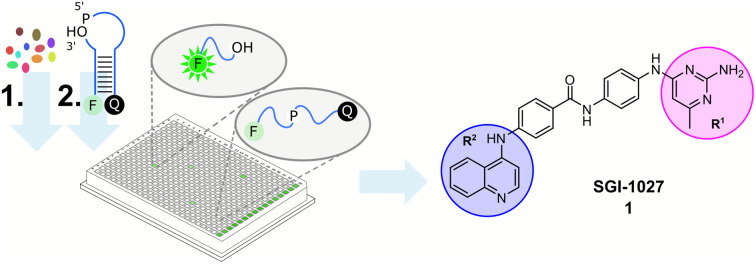

## Introduction

RNA ligases belong to the superfamily of nucleotidyl transferases and play a role in various cellular processes, such as RNA repair, splicing and editing.^1–4^ Human RNA ligase 1 (Rlig1) is the first human RNA ligase discovered to ligate 5′-phosphate to 3′-hydroxyl ends.^5^ Conserved across all vertebrates, Rlig1 operates through a distinct three step ligation mechanism. This process involves the formation of a covalent enzyme-adenylate intermediate at the K57 lysine in the catalytic site by auto-AMPylation to form Rlig1-AMP. The adenylate is subsequently transferred to the 5′-phosphate of the RNA, activating it for nucleophilic attack by the 3′-hydroxyl end. Rlig1 interacts with tRNA in cells and is capable of ligating 5′-ends of tRNA at the anticodon loop *in vitro*. Recent studies claimed that knockout mice exhibited altered tRNA levels in the brain, suggesting a potential role for Rlig1 in tRNA biology.^6^ Additionally, Rlig1-deficient human embryonic kidney (HEK293) cells showed significantly reduced viability and compromised RNA integrity compared to wild-type when treated with menadione, a molecule known to produce reactive oxygen species (ROS) in living cells.^5,7,8^ ROS are molecules that exhibit various physiological functions and are typically kept in balance by antioxidant systems.^9,10^ If this balance fails, the resulting oxidative stress will damage cellular macromolecules as carbohydrates, nucleic acids, lipids and proteins, and promote cell death.^11,12^ In experiments using identical ROS levels, only Rlig1-deficient cells showed reduced RNA integrity due to degradation of mainly 28S rRNA. This observation indicates that Rlig1 is likely involved in maintaining RNA integrity during cellular stress responses and it is hypothesized to be part of an RNA repair mechanism.^5^ While several DNA repair pathways, such as base-excision repair, mismatch repair or single- and double strand break repair, are well researched and understood, much less is known about how cells cope with oxidatively damaged RNA.^3,13–15^ RNA synthesis is energy consuming and repair could conserve energy and thus be evolutionarily beneficial. In general, cellular RNA is more susceptible to oxidative damage than DNA and damaged RNA can impair protein synthesis, which affects cell function and health.^16^ Increased oxidative stress and dysfunctioning repair mechanisms may contribute to the development of age-related neurodegenerative diseases, such as Alzheimer's and Parkinson's diseases.^17–21^ An increasing number of studies have highlighted that increased ROS levels are associated with various cancers, such as lung cancer, colorectal cancer, breast cancer, hepatocellular cancer, and cervical cancer.^22–26^ Notably, Rlig1 has also been found to be mutated and overexpressed in certain cancer cells, further underscoring its potential significance in cellular physiology and pathology.^27–29^ The sensitivity of Rlig1-deficient cells to ROS makes Rlig1 an attractive target. Selective inhibition of Rlig1 is critical to gain deeper insights into its role in RNA metabolism and its implication in a disease context. While DNA ligase inhibitors have been widely studied, no human RNA ligase inhibitors are known. This study focuses on the identification and characterization of such inhibitors, employing high-throughput screening (HTS) to discover potent compounds that can modulate Rlig1 activity. Therefore, a Förster resonance energy transfer (FRET)-based assay was developed to monitor RNA ligation and used to screen a library comprising of 13 026 bioactive small molecules. Among the identified small molecules, SGI-1027 (1) emerged as a promising lead compound, with its synthesized derivatives exhibiting low micromolar IC_50_ values and minimal cytotoxicity in HEK293 cells. Furthermore, our results demonstrate that Rlig1 inhibition, in conjunction with menadione-induced oxidative stress, reduces cell viability and accelerates RNA degradation, supporting the hypothesis that Rlig1 plays a critical role in the maintenance of RNA repair.

## Results and discussion

### High-throughput screening of bioactive compound library for Rlig1 inhibitors and hit validation

**Fig. 1:**
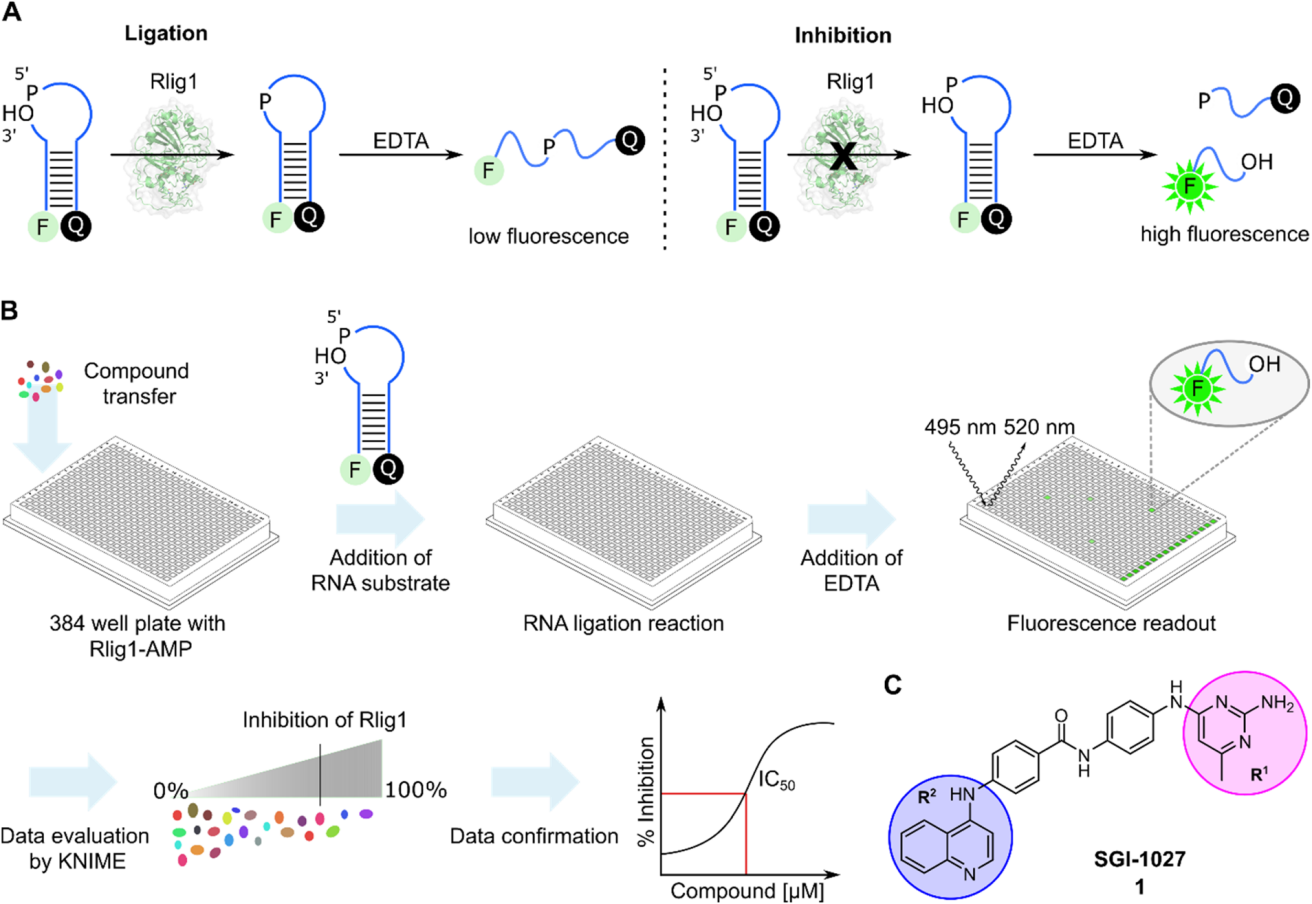
(A) General principle of the Rlig1 screen. A nicked RNA hairpin was labelled with FAM and BHQ1 at the 5′ and 3′ terminus, respectively. After ligation with Rlig1-AMP, EDTA is added to induce RNA unfolding, resulting in increased fluorescence intensity for the non-ligated substrate. In contrast, the fluorescence of the ligation product remains quenched, as the quencher and fluorophore are still in close proximity. RNA is illustrated in blue. (B) Schematic workflow of the high-throughput screening assay. Compound libraries were screened in a 384-well plate format to identify inhibitors of Rlig1-AMP. The data was evaluated using KNIME, followed by data confirmation and characterization. (C) Structure of SGI-1027 (1), which was identified as the most promising hit from the HTS, demonstrating over 70% inhibition at a 10 μM concentration. The indicated R^1^ and R^2^ residues were further investigated to understand their influence on the inhibitory effect on Rlig1-AMP activity.

In order to develop a fluorescent-based screen for Rlig1 activity, we modified a previously reported RNA substrate consisting of two RNA strands, with 6-carbofluorescein (FAM) and black hole quencher 1 (BHQ1) at the 5′ and 3′-ends, respectively.^5^ This RNA substrate forms a 8-nucleotide RNA stem with a 9-nucleotide 5′-phosphate overhang and a 2-nucleotide 3′-hydroxyl overhang, resulting in an open hairpin configuration. Upon addition of auto-AMPylated Rlig1 (Rlig1-AMP), the nick in the loop structure is sealed, forming a 27-nucleotide long sequence. To monitor the ligase activity by fluorescence, the RNA is subjected to denaturation by the addition of ethylendiaminetetraacetic acid (EDTA), resulting in single stranded oligonucleotides. For the substrate RNA this results in separating the donor and acceptor followed by an increase in fluorescence, whereas the product of the ligation reaction maintains the donor and acceptor in close proximity, resulting in low fluorescence intensity (Fig. 1A). With this principle, we screened a library comprising 13 026 biologically active compounds in a 384-well plate format (HY-L001, MedChemExpress) for their inhibition of Rlig1-AMP. The general procedure of the assay is depicted in Fig. 1B. Briefly, compounds dissolved in DMSO, water or ethanol were transferred to Rlig1-AMP at a final compound concentration of 10 μM. Controls were performed by the addition of DMSO to reactions with and without enzyme. After preincubation at 37 °C for 10 minutes, the RNA substrate was added, and the ligation reaction was performed at 37 °C for 20 minutes and was subsequently stopped with 20 mM EDTA. The reactions were incubated for additional 10 minutes, and the fluorescence intensity was measured in all wells using fluorescence excitation at 495 nm and emission at 520 nm. An additional control was performed to exclude effects of the small molecules on the fluorescence intensity by screening each compound plate also without enzyme. The relative enzymatic activity was calculated as the difference of the fluorescence measurement for each well with and without enzyme and normalized to the positive and negative control. In this approach, the reference maximal fluorescence difference is between the positive and negative control representing 100% inhibition. The percentage of inhibition for each compound was calculated using the KNIME analytics platform. As quality control *Z*′-values were calculated for each plate and the average *Z*′ score was 0.72 ± 0.04, demonstrating high reproducibility of the assay (Table S1†).^30^ Compounds were defined as hits if the inhibition was above 70%. Using this criterion, we identified 159 molecules as hits and selected 21 (1–21) of these for further testing based on their biological targets and chemical structure. The structure and targets of the corresponding hits are shown in Table S2 in the ESI.† The inhibitory potential of these molecules was validated using a radioactive ligation assay. In this assay, the 5′ open loop sequence was labelled with ^32^P-phosphate. Successful ligation would lead to a 27-mer oligonucleotide (RNA2-pRNA1) which is analyzed by denaturing polyacrylamide gel electrophoresis (PAGE). Formation of RNA-adenylate (AppRNA1) and self-cyclized RNA (cRNA1) could also be analyzed (Fig. S1†). Of the 21 compounds tested, 10 (1, 2, 6, 11–13, 17, 19–21) showed an inhibition of more than 70%. These 10 compounds have demonstrated to target proteins such as protein kinase, DNA methyltransferase, ATPases, and have also shown antibiotic activity.^31–34^ To further characterize the compounds, concentration-dependent assays were performed to determine IC_50_ values of all 10 compounds (Fig. S2 and S3†). Two of these compounds (11 and 13) exhibited IC_50_ values in the nanomolar range, but their toxoflavine-like structure made them susceptible to reduction and radical formation, potentially causing nonspecific inhibition and were thus not further investigated.^35^ Another initial hit from the screening was SGI-1027 (1), which exhibited an IC_50_ value of 3.96 μM for Rlig1-AMP. SGI-1027 (1) is a quinoline-based non-nucleoside that selectively inhibits DNA methyltransferases (DNMTs) with IC_50_ values of 6–13 μM and has low cytotoxicity.^36^1 is composed of four core aromatic moieties, including an aminopyrimidine group (R^1^) and a quinoline moiety (R^2^), which are critical for its interaction with DNMTs. Molecular docking studies of 1 with *Haemophilus haemolyticus* cytosine-5 DNA methyltransferase (MHhal C5 DNMT) demonstrated that SGI-1027 (1) fits within the enzyme's catalytic pocket with its R^1^ and R^2^ moieties.^37^ The inhibition mechanism involves competition with the cofactor *S*-adenosyl methionine.^36^ Here, 1 inhibited the formation of the RNA-adenylate intermediate (Fig. S3†). This suggests that the inhibitor may act through competitive binding with the RNA substrate or by the induction of an allosteric effect. However, due to the absence of a crystal structure for AMPylated Rlig1, molecular docking studies with 1 are not accessible. To identify the structural elements crucial for Rlig1 inhibition, truncated derivatives lacking the R^1^ or R^2^ groups were synthesized. The compounds were tested for their inhibitory potential for Rlig1-AMP at 10 μM in the radioactive *in vitro* ligation assay. These truncated compounds (22–25) exhibited no detectable activity at the chosen conditions, indicating that R^1^ and R^2^ are both essential for binding and their ability to inhibit Rlig1-AMP (Fig. S4†). Based on these findings, we synthesized new derivatives of SGI-1027 (1) modified at the R^1^ and R^2^ moiety to explore important structural motifs.

### Structure activity relationship (SAR) study and synthesis of SGI-1027 derivatives

**Fig. 2:**
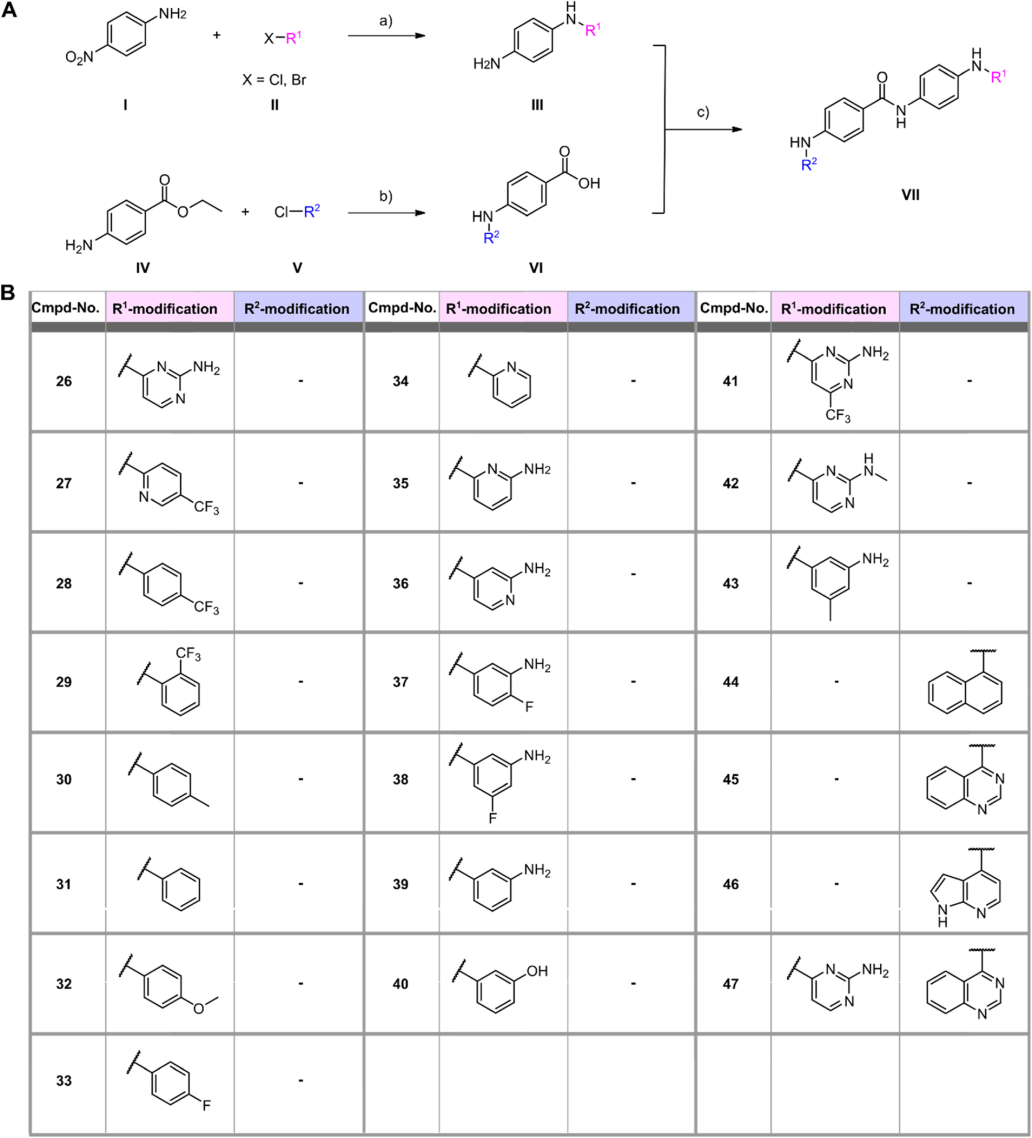
(A) General synthesis scheme for generating SGI-1027 derivatives. Reagents and conditions: (a) (1) Pd(OAc)_2_, XPhos, K_2_CO_3_, 110 °C, 1–24 h (*t*BuOH) (2) Pd/C, HCOONH_4_, r.t., 5 h (EtOH); (b) (1) Pd(OAc)_2_, XPhos, K_2_CO_3_, 110 °C, 1–24 h (*t*BuOH) (2) LiOH, H_2_O, r.t., 24 h (THF/MeOH); (c) HOBt, EDCl, NEt_3_, 70 °C, 6 h (DMF). (B) Structures of R^1^ and R^2^ modifications of all synthesized SGI-1027 derivatives.

**Fig. 3:**
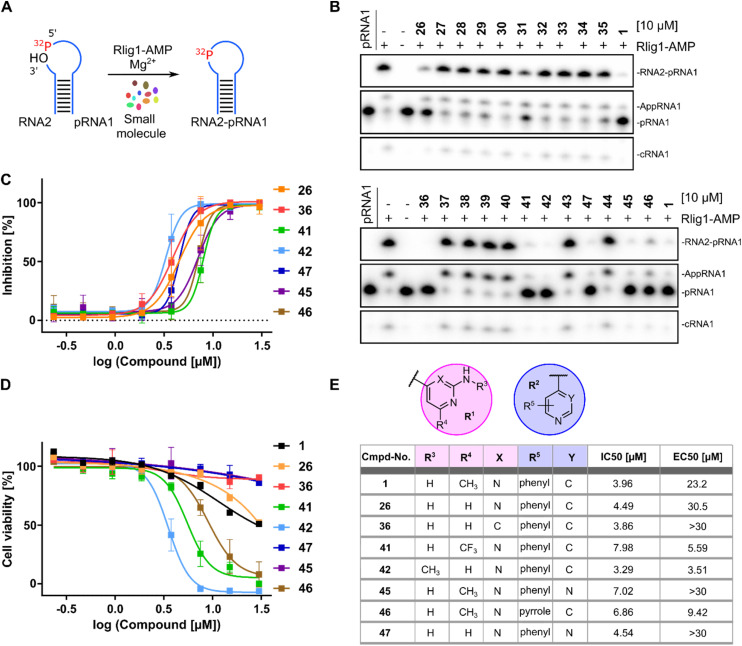
(A) Schematic representation of RNA ligation assay with ^32^P-labelled (red) RNA for biological testing of synthesized compounds. (B) Urea-PAGE analysis of reaction products after incubation with 500 nM Rlig1-AMP and 10 μM compounds for 20 minutes at 37 °C. Ligation was inhibited by compounds 26, 36, 41, 42, 45, 46 and 47. SGI-1027 (1) was used as reference. (C) Concentration-dependent curves for all active derivatives against Rlig1-AMP *in vitro*. The *Y*-axis shows the percentage inhibition normalized to the positive control, plotted against the logarithm of compound concentration. Error bars represent ± SEM (*n* = 3). (D) Cell viability plot of HEK293 cells treated with active compounds at different concentrations. The percentage of viable cells was normalized to untreated cells. Error bars represent ± SEM (*n* = 3). (E) Table of SGI-1027 (1) and R^1^ and R^2^ modifications with corresponding IC_50_ and EC_50_ values. Source data are provided as a Source Data file.

In a first series of compounds, the R^1^ residue was chemically modified with a variety of functional groups that were commercially available to verify the activity of R^1^. This included aromatic rings with different substituents. The synthesis of all derivatives followed a published route that involves five steps (Fig. 2).^38^ The intermediates (III) were synthesized in two steps starting from 4-nitroaniline (I) and the substituted aryl halides (II), which were cross-coupled using a Buchwald–Hartwig amination reaction. The nitro group was then reduced with Pd/C and ammonium formate to obtain intermediate (III). As detailed in the ESI,† benzene-1,4-diamine was used for coupling in some cases due to otherwise low yields, allowing intermediate (III) to be obtained in a single synthesis step. Compounds with free amine groups lacking hetero atoms in the aromatic ring were previously BOC-protected to reduce side reactions. Intermediate (VI) was synthesized through another Buchwald–Hartwig amination reaction involving ethyl 4-aminobenzoate (IV) and the respective aryl chlorides (V). Saponification with lithium hydroxide yielded the free carboxylic acid intermediate (VI). Final compound (VII) is obtained through a condensation reaction using 1-ethyl-3-(3-dimethylaminopropyl)carbodiimide (EDCl) and hydroxybenzotriazole (HOBt) (Fig. 2A). In total 18 derivatives (26–43) with R^1^ modifications were synthesized (Fig. 2B). Next, the compounds were assayed for their ability to inhibit the catalytic activity of Rlig1-AMP at 10 μM by use of the *in vitro* radioactive ligation assay as described above, with lead compound SGI-1027 (1) as reference (Fig. 3B and S5†). While compound 26, 36, 41 and 42 fully inhibited the activity of Rlig1-AMP, all other derivatives showed no inhibition. Interestingly, all those four compounds are characterized by the presence of the 2-amino pyridine group. A lack of the methyl group for compound 26 or the exchange to a trifluoromethyl group at the C6 position group has no effect on the inhibition. Also, methylation of the 2-amino group resulted in inhibition of Rlig1. The significance of the 2-aminopyridine moiety as a crucial structural motif is underscored by its potential role in facilitating hydrogen bonding interactions. It is likely involved in forming key hydrogen bonds with critical amino acids in the active site of Rlig1-AMP, potentially within the RNA binding pocket.

In a second small series of derivatives, three modifications were introduced on the R^1^ residue and assayed towards Rlig1-AMP inhibition. Here, compound 44, which lacks the pyridine nitrogen, was inactive, while compound 45 and 46 inhibited ligase activity. Lastly, one derivative with modified R^1^ and R^2^, previously shown to be active, was synthesized to determine whether dual modifications are tolerated. This derivative 47 was also found to inhibited Rlig1-AMP at 10 μM concentration (Fig. 3B).

### Evaluation of the SGI-1027 derivatives

In the next step, all active SGI-1027 derivatives were evaluated for their relative inhibitory potential by determining IC_50_ values. For this purpose, the radioactive ligation assay was performed as described above at various concentrations in triplicates. Overall, none of the derivatives significantly exceeded the inhibitory potential of the initial hit SGI-1027 (1) (Fig. 3C, E and S6†). To identify molecules that combine high enzymatic activity with low cytotoxicity against living cells, all bioactive compounds were tested for their cytotoxic properties using the HEK293 cell line, which has been previously utilized in Rlig1 investigations.^5,8^ Cytotoxicity was assessed using a luminescence cell viability assay, as described by Yuan *et al.*^5^ Cells were cultured for 24 hours, followed by the addition of compounds in a dose-dependent manner using a 384-well format. After an additional 24 hours incubation, the assay reagent was added to lyse the cells and convert cellular ATP into a luminescent signal, which serves as a key biomarker of metabolically active cells. Healthy cells produce a higher luminescent signal compared to dead cells, allowing for the quantification of cytotoxic effects. For lead compound SGI-1027 (1), the determined EC_50_ value was 23.2 μM. Among the derivatives, three compounds (36, 45, 47) exhibited no detectable cytotoxicity at the highest concentration of 30 μM, whereas three compounds (41, 42, 46) showed comparatively high cytotoxicity. Compound 26 performed similarly to the lead compound 1, with an EC_50_ value of 30.5 μM. The results indicated that modification of the R^1^ position on the C6 position of the pyrimidine ring with a trifluoromethyl group in 41 increased cytotoxicity. Additionally, methylation of the amine group in 42 also resulted in higher cytotoxicity. For the R^2^ residue, modifying the second benzene ring to a pyrrole ring also led to increased cytotoxicity (Fig. 3D and E).

### Reduced cell viability and RNA integrity of HEK293 cells under oxidative stress

**Fig. 4:**
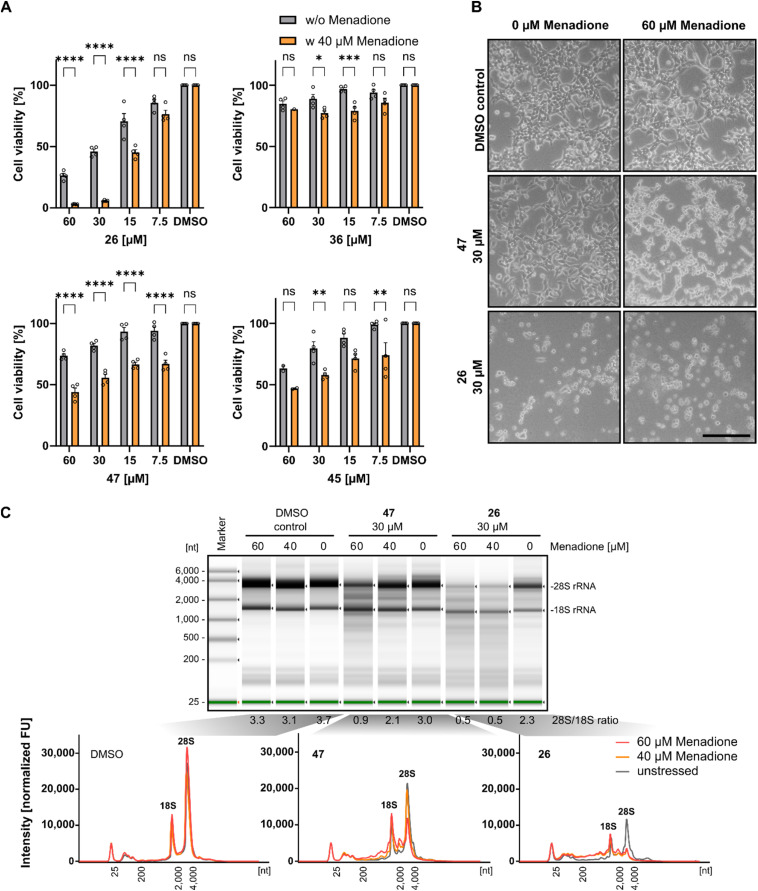
(A) Comparison of cell viability of HEK293 cells treated with compound 26, 36, 45 and 47 with and without 5 hours treatment with 40 μM menadione. Percentage cell viability was normalized to HEK293 cells treated without inhibitor or menadione. Hollow circles represent individual data points for *n* = 4 biological replicates. Error bars represent ± SEM. Statistical significance was calculated using two-way ANOVA with Sidak's multiple comparisons test: ^ns^*P* > 0.05; *P ≤ 0.05; **P ≤ 0.01, ***P ≤ 0.001, ****P < 0.0001. (B) Light microscopy of HEK293 treated with 30 μM 47 and 26, followed by 5 hours treatment of 60 μM menadione. Scale bar is 500 μm. (C) Analysis of the total RNA from cell extract of HEK293 cells treated with inhibitor for 24 h, followed by treatment with 40 μM and 60 μM menadione. RNA was isolated and analyzed using TapeStation (version 4.1.1). (top) Separation profile of analyzed samples. The ratio of 28S and 18S rRNA intensities is listed below the profile. (bottom) Electropherograms of total RNA from cells treated with DMSO as control, 47 and 26 at different menadione concentrations. The 18S/28S peaks are annotated. Experiments with DMSO and 40 μM menadione were conducted in triplicate, while those with 60 μM menadione were performed in duplicate. Data for compound 1 is shown in Fig. S7.† Source data are provided as a Source Data file.

Previous investigations demonstrated that Rlig1-deficient HEK293 cells exhibit similar phenotypes to wild-type cells in terms of growth, adherence, and appearance. However, significant differences in viability levels were observed when cells were exposed to 40 μM menadione, a compound known to induce ROS-based cellular stress.^5,39^ Based on this, we next investigated if Rlig1 inhibition manifests similar effects on wild-type HEK293 cells. Cells were treated with various concentrations of the respective inhibitors (1, 26, 36, 45, 47) for 24 hours, followed by exposure to menadione for five hours to assess effects on cell viability. Compounds 41, 42, and 46 were excluded from this experiment because of their inherent cytotoxicity to HEK293 cells. The cell viability was normalized to the DMSO controls with or without menadione. Our results showed that inhibitor-treated cells exhibited significantly reduced viability for all tested compounds in the range of 15–60 μM inhibitor compared to cells without oxidative stress. The most pronounced effects were observed for compound 1, 26 and 47 (Fig. 4A and S7A†). For compound 1 and 26, which reduced the cell viability to 50% at 30 μM concentration without oxidative stress, cells exhibited almost complete loss of viability under stress conditions. Differences in cell morphology were also observed using light microscopy for treated cells with compound 1, 26 and 47, displaying poor adherence and cell shrinkage, which are characteristics of menadione induced cell death previously noted in the Rlig1-deficient HEK293 cell line (Fig. 4B and S7B†).^5,40,41^ Our most promising candidate, 47, showed significantly reduced viability only when the cells were stressed. Morphological changes became more prominent at 60 μM menadione. These results align with previous findings for a Rlig1-deficient HEK293 cell line and confirm a potential role for Rlig1 in responding to oxidative stress.^5^ However, the increased sensitivity to oxidative stress might also result from a synergistic effect when cells are treated with both inhibitor and menadione. To investigate this further, we isolated and analyzed the total RNA from HEK293 cells treated first with 30 μM of 1, 26 and 47 followed by exposure to 40 and 60 μM menadione. As controls, cells were grown without either inhibitor or menadione, instead DMSO was added. RNA integrity was assessed by analyzing the 28S and 18S rRNA, with an RNA 28S : 18S ratio of 2 or greater indicating high quality.^42^ RNA degradation was observed only when cells were co-treated with both the inhibitor and menadione, and this degradation increased with higher concentrations of menadione. The 18S rRNA signal intensity remained relatively unchanged, whereas the 28S rRNA showed a greater susceptibility to ROS-induced damage. Interestingly, cells treated solely with 1 and 26 showed reduced viability, as previously demonstrated, but maintained an RNA integrity level above the threshold of 2 (Fig. 4C and S7C†). These findings underscore the crucial role of Rlig1 in maintaining RNA integrity under oxidative stress, supporting the hypothesis that Rlig1 is involved in an RNA maintenance mechanism in humans.^5^

### No inhibitory effect on human DNA ligase 1

**Fig. 5:**
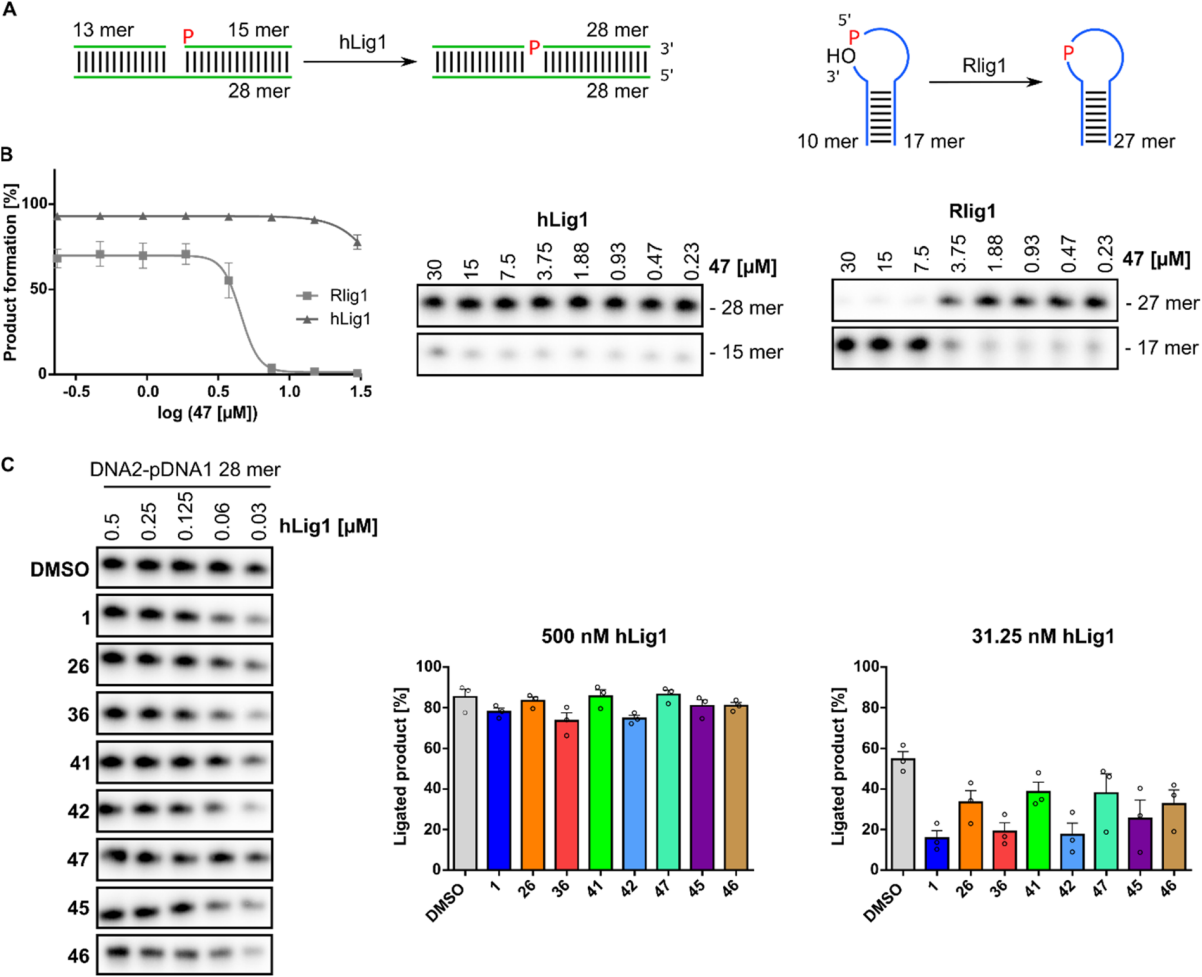
(A) Schematic representation of the DNA substrate and ligation assay with human DNA ligase 1 (hLig1) compared to Rlig1. DNA and RNA are illustrated in green and blue (B) inhibition analysis of compound 47: (left) plot of the percentage product formation at varying concentrations of compound 47 for hLig1 and Rlig1 ligation reaction for 20 minutes at 37 °C. Error bars represent ± SEM (*n* = 3). (right) Urea-PAGE gel of hLig1 and Rlig1 promoted ligation turnover at various concentrations of compound 47. (C) DNA product formation across all active SGI-1027 derivatives: (left) representative images of ligation reaction at various concentrations of hLig1 for 30 minutes at 37 °C. (right) Plot of percentage product formation for all derivatives at 500 nM and 31.3 nM hLig1 concentration. Hollow circles represent individual data points for *n* = 3 biological replicates. Error bars represent ± SEM. Source data are provided as a Source Data file.

Human DNA ligase 1 (hLig1) belongs to the ATP-dependent nucleotidyl transferase family and plays a crucial role in DNA replication and repair.^43^ Mutations in hLig1 are known to promote aberrant DNA replication, repair issues, hypersensitivity to DNA-damaging agents, and can lead to immunodeficiency and increased cellular apoptosis.^44–46^ To evaluate the selectivity of the identified active Rlig1 inhibitors, we expressed and purified the hLig1 adapted from a published protocol.^47^ A nicked double-stranded DNA oligonucleotide construct, previously reported as an hLig1 substrate, was used in the radioactive ligation assay.^48^ Initially, we matched the enzyme and substrate concentration to those used in Rlig1 experiments and titrated the concentration of inhibitor 47 in a 1 : 2 dilution series starting from 30 μM compound. No effect was observed on DNA ligase activity under these conditions (Fig. 5A, B and S8†). Given the high activity and rapid kinetics of hLig1, we next titrated the concentration of hLig1. All seven active compounds, along with the lead compound 1, were analyzed at a constant concentration of 10 μM. DNA product formation is shown in Fig. 5B and S8.† The percentage of product formation at 500 nM and 31.3 nM hLig1 for each compound was plotted for biological triplicates in Fig. 5C. The results demonstrated that hLig1 remains highly active even at very low concentrations. At 500 nM hLig1, no significant effects on ligase activity were observed for any of the compounds. However, at 31.3 nM hLig1, variations in DNA ligase activity were noted, including the DMSO control. As shown in Fig. 5C, 41 and 47 exhibited the least impact on hLig1 activity, suggesting their selectivity for Rlig1-AMP.

## Conclusion

Rlig1 is an RNA ligase that covalently joins 5′-phosphates to 3′-hydroxyl ends.^5,6^ Previous research has demonstrated that Rlig1-deficient cells exhibit increased sensitivity to ROS.^5^ Dysregulated ROS levels are implicated in the development of several diseases, including neurodegenerative disorders and cancer.^17–26^ Understanding the biological role of Rlig1 in this context is therefore crucial. Selective targeting of Rlig1 could enhance our knowledge of its involvement in RNA maintenance mechanisms and potentially offer therapeutic applications. In this study we identified SGI-1027 (1) as an initial inhibitor of Rlig1 through high-throughput screening using a FRET-pair labelled RNA substrate. Based on this discovery, we synthesized 22 derivatives, most of them not previously reported, and identified seven with IC_50_ values of less than 8 μM against Rlig1-AMP. The compounds were further assessed for cytotoxicity in HEK293 cells. Three compounds showed no cytotoxicity (36, 45, 47), 26 and lead compound SGI-1027 (1) exhibited moderate cytotoxicity, and three compounds (41, 42, 46) showed high cytotoxicity. Additional analysis revealed that Rlig1 inhibition under oxidative stress conditions leads to reduced cell viability and RNA degradation, mirroring the phenotypes observed in Rlig1-deficient cells. To evaluate the selectivity of the identified inhibitors, we investigated their effects on the activity of human DNA ligase hLig1. The synthesized derivatives did not significantly inhibit its activity, suggesting a selective inhibition of Rlig1. Among the tested compounds, compound 47 emerged as the most promising inhibitor, exhibiting low cytotoxicity, enhanced sensitivity to ROS, and high selectivity for Rlig1. Our findings not only provide insights into the structural requirements for effective inhibition of Rlig1 in living cells but also highlight its critical role in RNA maintenance under stress conditions. The observed effects of Rlig1 inhibition on RNA integrity and cell viability, particularly under oxidative stress, underscore its potential as a key player in cellular stress response pathways. These results serve as a foundation for developing innovative new therapeutics targeting RNA repair mechanisms, offering new avenues for treatments in conditions where reactive oxygen species (ROS) are dysregulated.

## Data availability

All data required is provided in the manuscript and the ESI.† Source data are provided with this paper.

## Author contributions

L. A. S. and A. M. conceived and designed the project; L. A. S. and S. M. performed the high-throughput screening and data evaluation. L. A. S. performed protein expression, biological assays and synthesis. M. P. performed synthesis. L. A. S. and A. M. wrote the manuscript with input from all authors.

## Conflicts of interest

There are no conflicts to declare.

## Supplementary Material

SC-016-D4SC06542E-s001

SC-016-D4SC06542E-s002
