# Supplementary material for: Potent inhibitors of the human RNA ligase Rlig1 highlights its role in RNA integrity maintenance under oxidative cellular stress
Source: Chem Sci. 2025 Jan 18;16(7):3313–22. doi: 10.1039/d4sc06542e (PMC11747885; doi:10.1039/d4sc06542e)
Supplement: SC-016-D4SC06542E-s002 [file SC-016-D4SC06542E-s002.pdf]

Supplementary Information (SI) for Chemical Science.
This journal is © The Royal Society of Chemistry 2025

Supplementary Information

Potent inhibitors of the human RNA ligase Rlig1 highlights its role in RNA integrity maintenance under oxidative cellular stress

Lisa A. Schlor[a,b], Maya Peußner[a], Silke Müller[c,d], Andreas Marx[a,b]

a Department of Chemistry, University of Konstanz, Universitätsstraße 10, 78457 Konstanz, Germany. E-mail: andreas.marx@uni-konstanz.de

b Konstanz Research School Chemical Biology, University of Konstanz, Universitätsstraße 10, 78457 Konstanz, Germany.

c Department of Biology, University of Konstanz, Universitätsstraße 10, 78457 Konstanz, Germany.

d Screening Center, University of Konstanz, Universitätsstraße 10, 78457 Konstanz, Germany.

Table of contents

1 Supplementary figures 4

2 Supplementary tables 10

3 Methods 15

3.1 Biochemical experiments 15

Plasmid construction 15

Expression and purification of recombinant proteins 16

High-throughput screening of inhibitors for Rlig1-AMP 17

Preparation of 5’ ^{32}P-labelled oligonucleotides....18

Radioactive ligation assays 18

Urea-PAGE and autoradiographic imaging 19

3.2 Cell culture 20

Cell viability assay of HEK293 cells treated with inhibitors 20

Cell viability assay of HEK293 cells treated with inhibitors and menadione 20

Light-microscopy of inhibitor- and menadione-treated HEK293 cells 21

RNA integrity analysis of HEK293 cells treated with inhibitors and different concentrations of menadione 21

3.3 Chemical synthesis 22

General experimental details 22

Analytical methods 22

General procedures 23

General procedure A: Boc-protection of aryl amines 23

General procedure B: Buchwald-Hartwig amination 24

General procedure C: reduction of nitroaryls 36

General procedure D: ester hydrolysis 43

General procedure E: amide coupling 46

Synthesis of truncated SGI-1027 structures 64

4 Supplementary references 67

5 Appendix 67

Amino acid sequence of expressed proteins 67

Sequence of plasmids used in this study..67

NMR Spectra 70

1 Supplementary figures

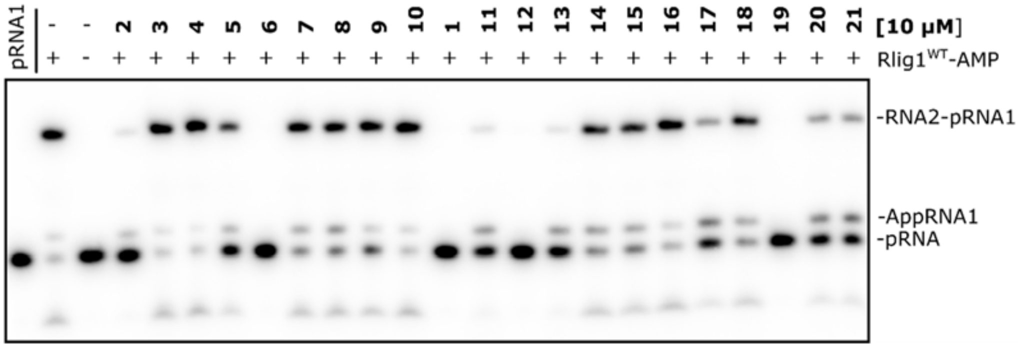

Fig. S1: Hit validation of 21 compounds derived from high-throughput screening in a radioactive RNA ligation assay. PAGE analysis of reaction products after incubation with Rlig1 and 10 µM of each compound at 37°C for 20 minutes (representative image of n = 2).

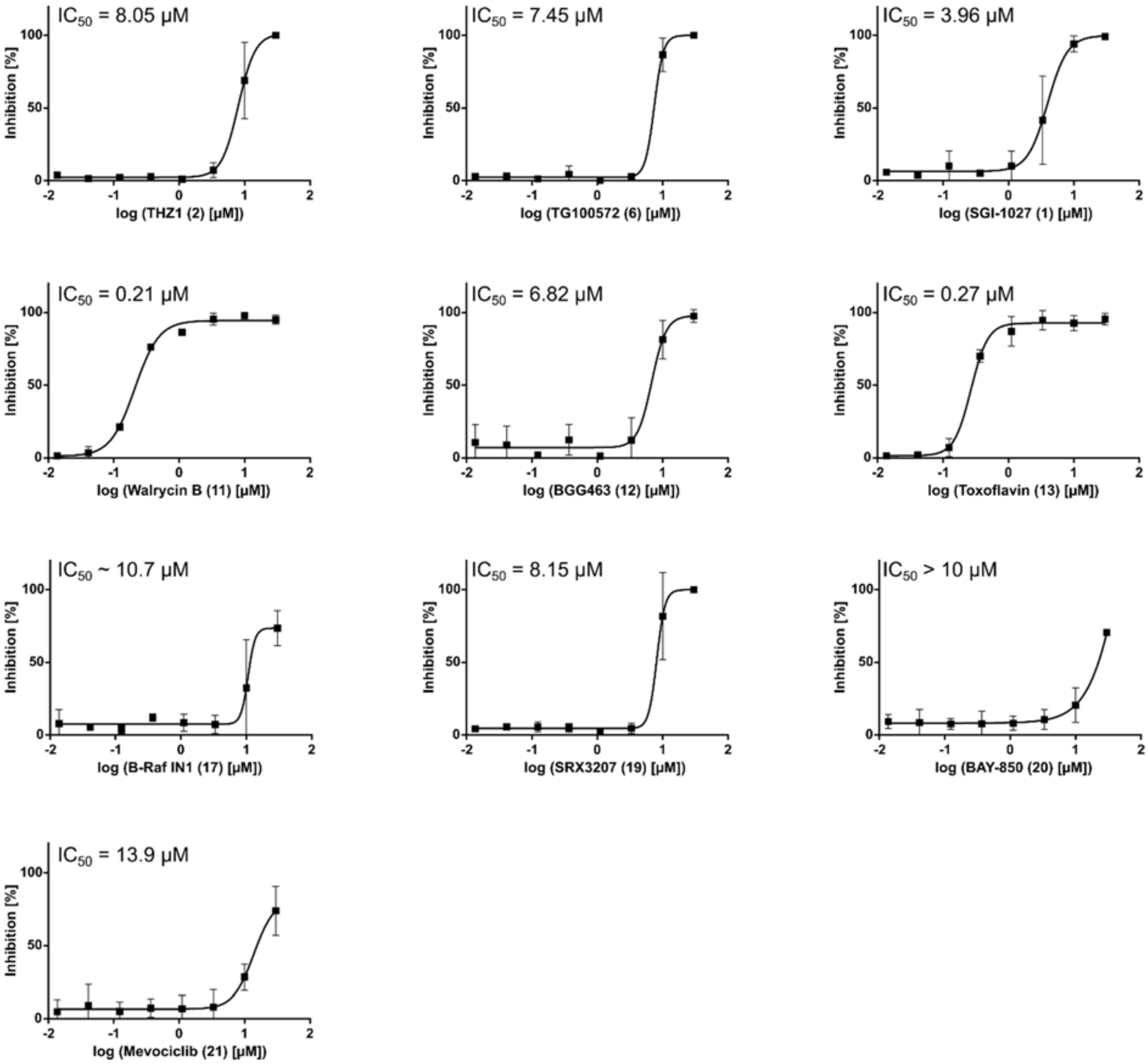

Fig. S2: Dose-response curves of active hits *in vitro* against Rlig1-AMP. The IC_{50} values were calculated by a non-linear curve fit/dose-response curve in GraphPad Prism. All data represent the mean value for three biological replicates. Error bars represent ± SEM. Source data are provided as a Source Data file.

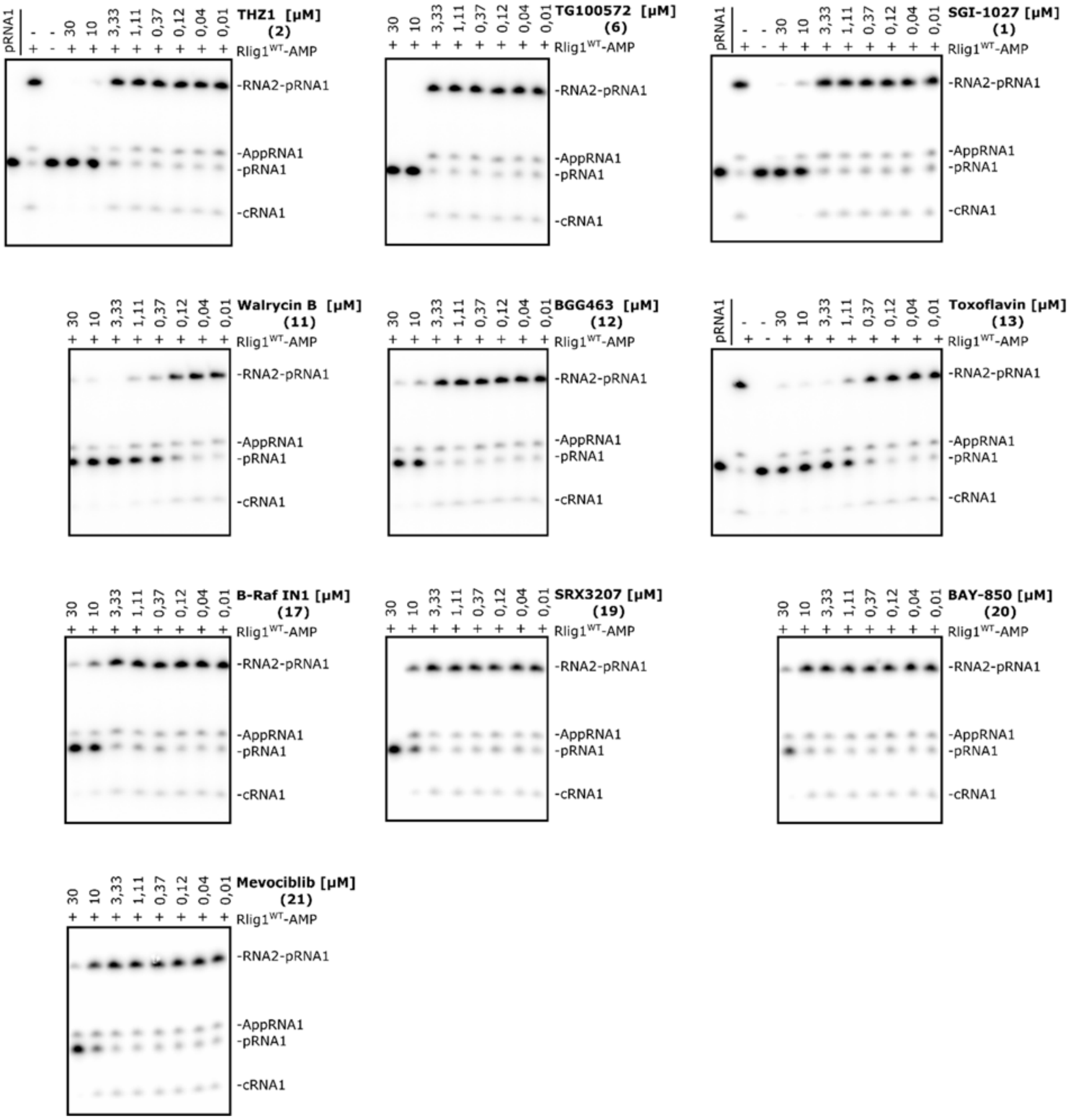

Fig. S3: Urea-PAGE analysis of dose-response experiment for active MCE hits (representative image of n = 3). RNA ligation reactions were performed with 500 nM Rlig1-AMP and 50 nM pRNA for 20 min at 37 °C at varying concentrations of compounds (30 – 0.01 µM). The rate of RNA-pRNA formation was quantified by the intensities of the bands for each chamber and normalized to the positive control.

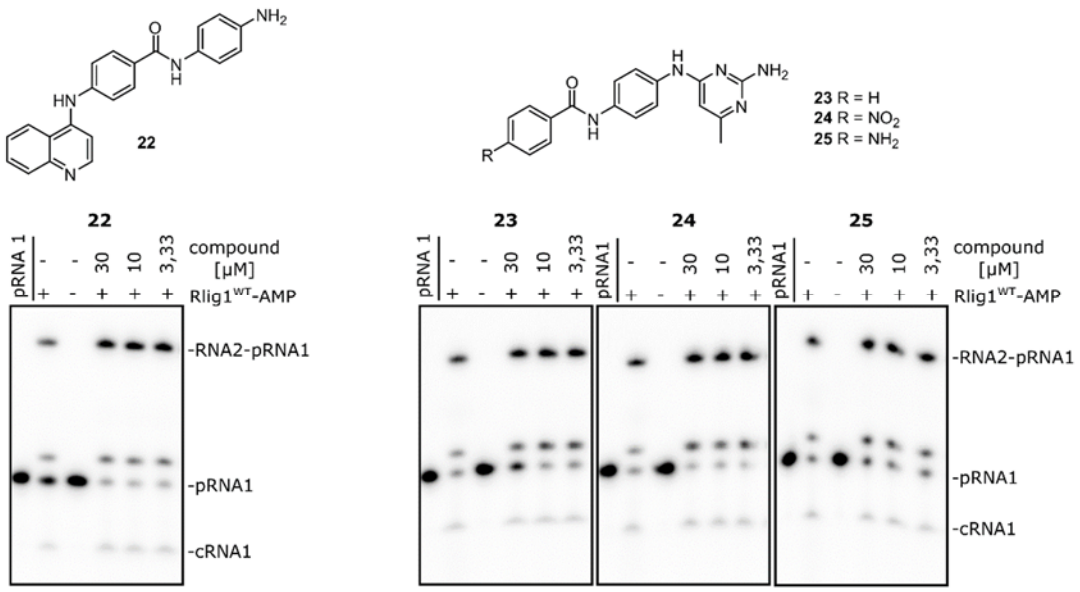

Fig. S4: Structure of the synthesized truncated SGI-1027 derivatives **22**-**25** and biological evaluation by urea-PAGE analysis. RNA ligation reactions were performed with 500 nM Rlig1-AMP and 50 nM pRNA1 for 20 min at 37 °C at varying concentrations of compounds (30, 10, 3.33 µM).

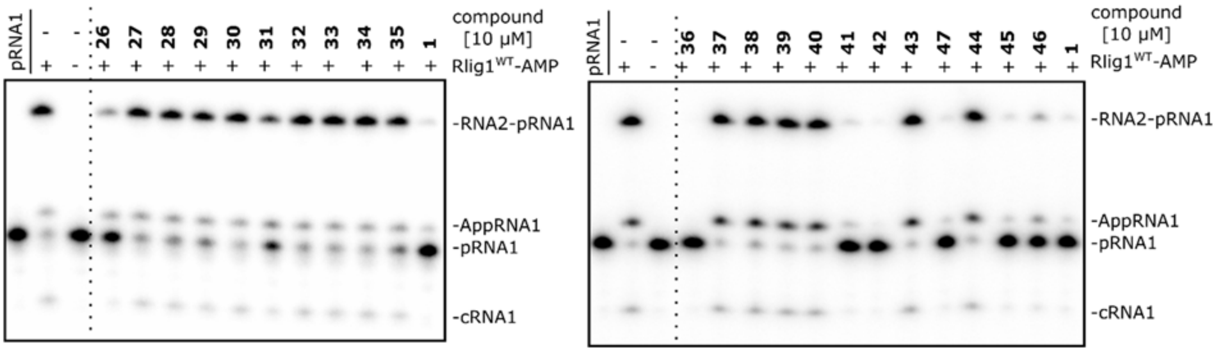

Fig. S5: Non-cropped PAGE analysis depicted in **Fig. 3B**. RNA ligation reactions were performed with 500 nM Rlig1-AMP and 50 nM pRNA1 for 20 min at 37 °C at a constant compound concentration of 10 µM.

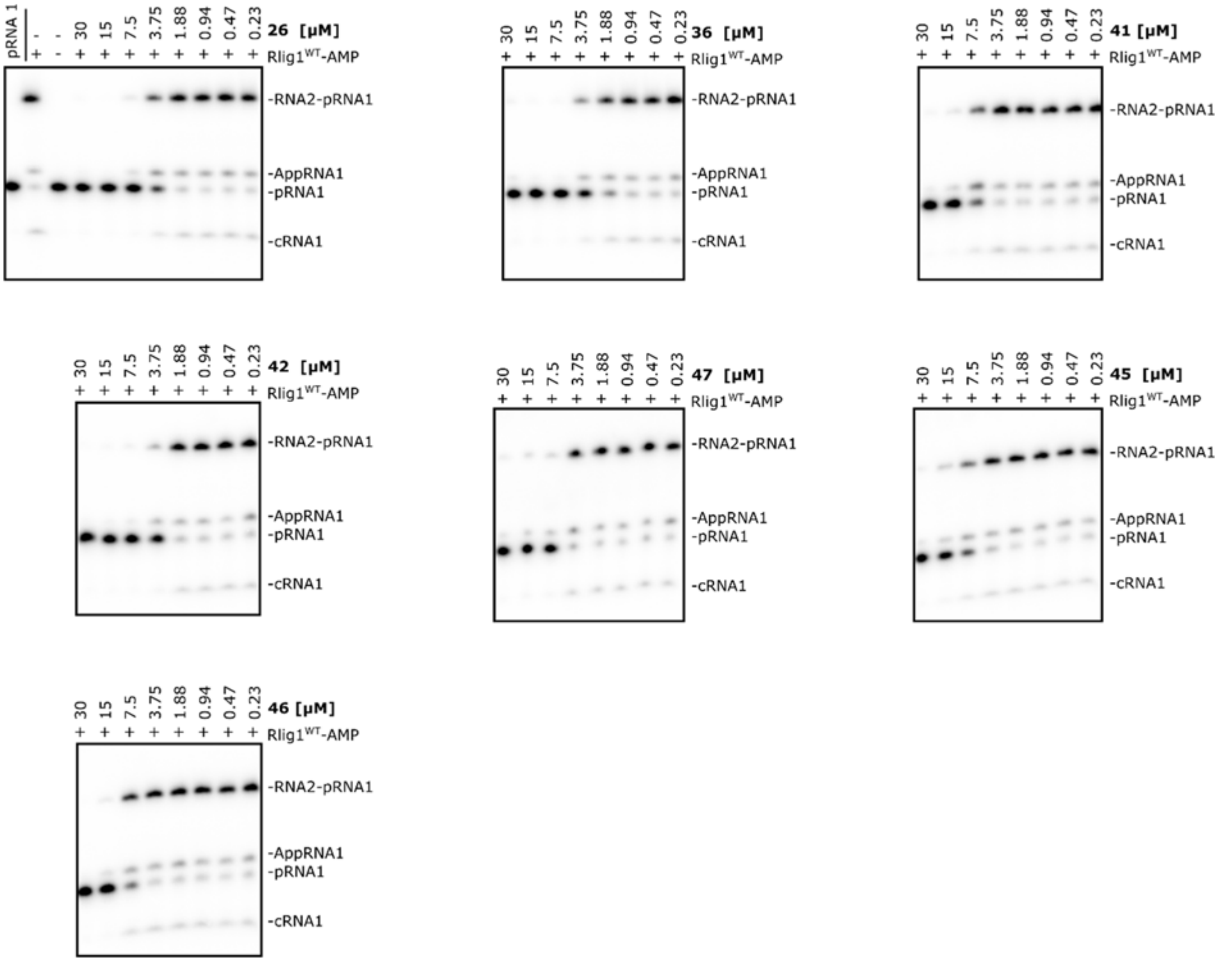

Fig. S6: Urea-PAGE analysis of dose-response experiment for active SGI-1027 derivatives (representative image of n = 3). RNA ligation reactions were performed with 500 nM Rlig1-AMP and 50 nM pRNA1 for 20 min at 37 °C at varying concentrations of compounds (30 – 0.23 µM). The rate of RNA2-pRNA1 formation was quantified by the intensities of the bands for each chamber and normalized to the positive control. Source data are provided as a Source Data file.

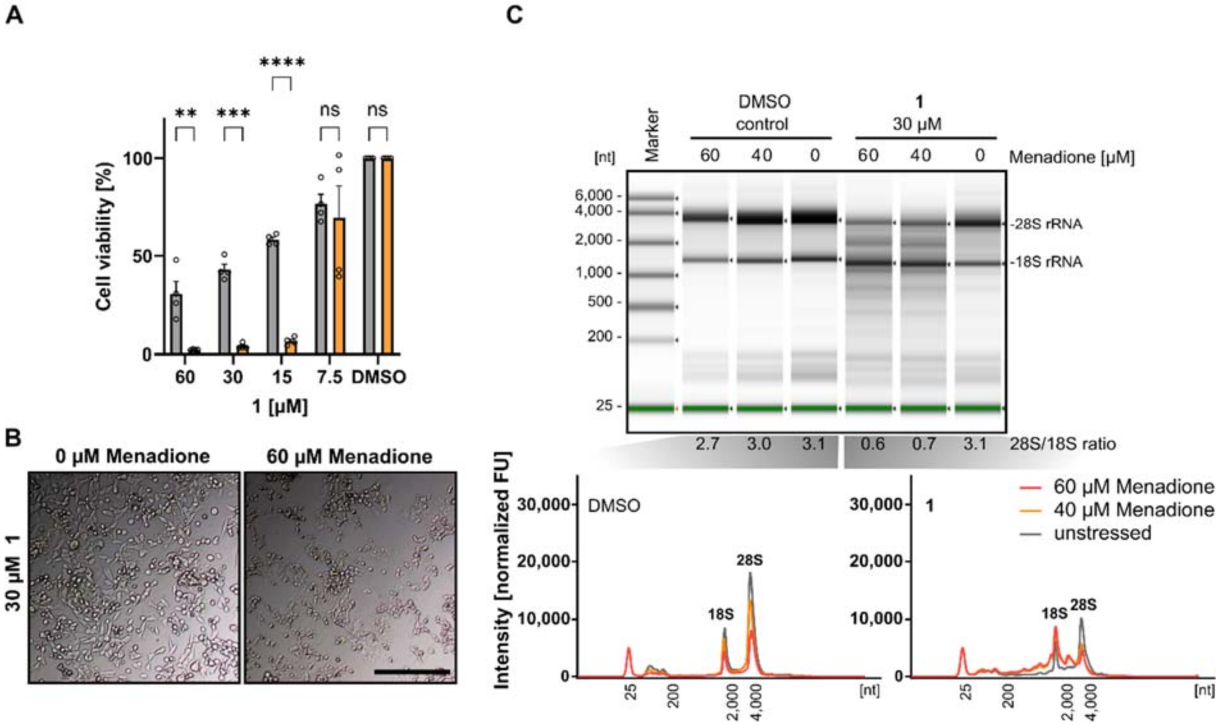

Fig. S7: A) Comparison of cell viability of HEK293 cells treated with compound **1** with and without 5-hour treatment with 40 μM menadione. Percentage cell viability was normalized to HEK293 cells treated without inhibitor or menadione. Hollow circles represent individual data points for n = 4 biological replicates. Error bars represent ± SEM. Statistical significance was calculated using two-way ANOVA with Sidak's multiple comparisons test: $^{ns}P > 0.05$; $^{*}P \leq 0.05$; $^{**}P \leq 0.01$, $^{***}P \leq 0.001$, $^{****}P < 0.0001$. B) Light microscopy of HEK293 treated with 30 μM **1,** followed by 5-hour treatment of 60 μM menadione. Scale bar is 500 μm. C) Analysis of the total RNA from cell extract of HEK293 cells treated with compound **1** for 24 h, followed by treatment with 40 μM and 60 μM menadione. RNA was isolated and analyzed using TapeStation (version 4.1.1). (top) Separation profile of analyzed samples. The ratio of 28S and 18S rRNA intensities is listed below the profile. (bottom) Electropherograms of total RNA from cells treated with DMSO as control and compound **1** at different menadione concentrations. The 18S/28S peaks are annotated. Experiments were conducted in duplicate. Source data are provided as a Source Data file.

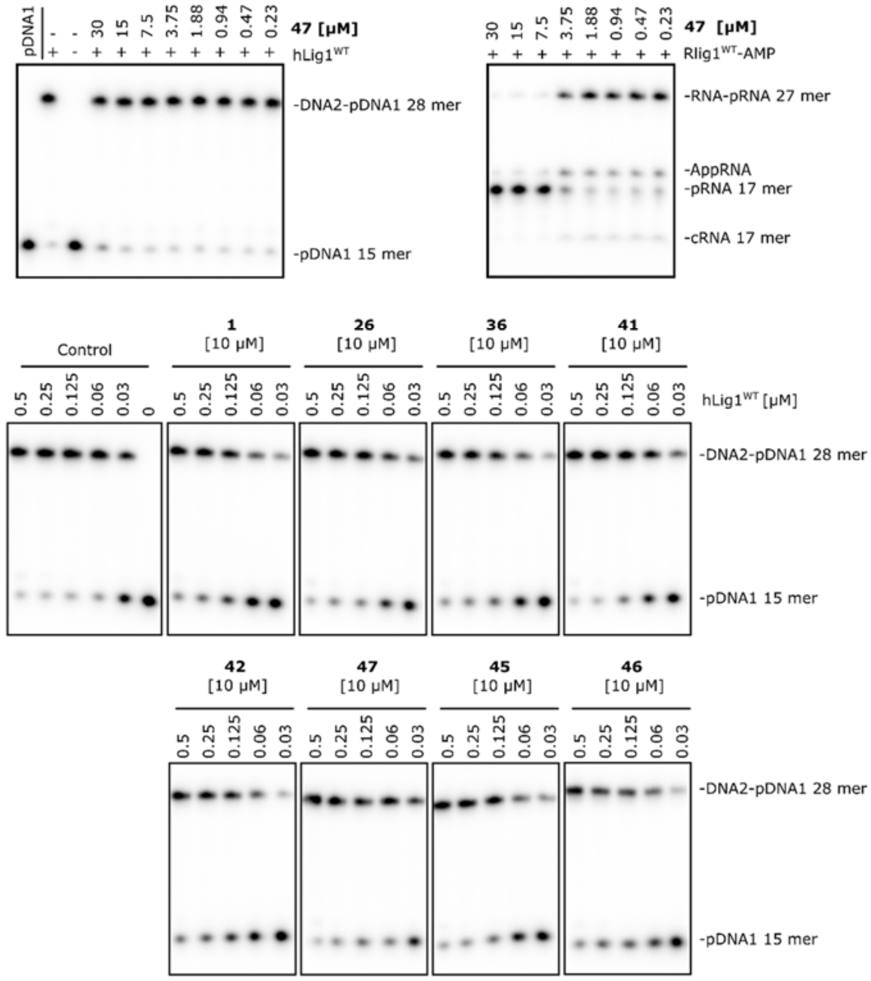

Fig. S8: (top) Urea-PAGE analysis of comparison of DNA ligation with hLig1 and RNA ligation with Rlig1 across various concentrations of compound **47**. Reactions were performed for 20 min at 37 °C. (bottom) Urea-PAGE analysis of DNA ligation turnover at varying hLig1 concentrations with a constant 10 µM concentration of all active SGI-1027 derivatives. Reactions were performed for 30 min at 37 °C (representative images, n = 3).

2 Supplementary tables

Table S1: Overview of all compound plates that were tested in HTS with corresponding Z' values.

Plates	Filename-timepoint	Z'-Factor	Plates	Filename-timepoint	Z'-Factor
1	220704023HYCPK27668b-2	0.719	24	220714035HYCPK27691b-2	0.767
2	220704025HYCPK27669b-2	0.711	25	220714037HYCPK27692b-2	0.758
3	220704027HYCPK27670b-2	0.716	26	220714039HYCPK27693b-2	0.738
4	220704029HYCPK27671b-2	0.817	27	220714041HYCPK27694b-2	0.731
5	220704031HYCPK27672b-2	0.727	28	220714042HYCPK27695b-2	0.825
6	220704033HYCPK27673b-2	0.692	29	220718025HYCPK27696b-2	0.609
7	220704035HYCPK27674b-2	0.778	30	220718027HYCPK27697b-2	0.692
8	220704036HYCPK27675b-2	0.708	31	220718029HYCPK27698b-2	0.666
9	220707025HYCPK27676b-2	0.678	32	220718031HYCPK27699b-2	0.685
10	220707027HYCPK27677b-2	0.718	33	220718033HYCPK27700b-2	0.730
11	220707029HYCPK27678b-2	0.731	34	220718035HYCPK27701b-2	0.745
12	220707031HYCPK27679b-2	0.725	35	220718037HYCPK27702b-2	0.736
13	220707033HYCPK27680b-2	0.740	36	220718039HYCPK27703b-2	0.670
14	220707035HYCPK27681b-2	0.685	37	220718041HYCPK27704b-2	0.719
15	220707037HYCPK27682b-2	0.697	38	220718042HYCPK27705b-2	0.732
16	220707039HYCPK27683b-2	0.712	39	220720023HYCPK27706b-2	0.698
17	220707041HYCPK27684b-2	0.711	40	220720025HYCPK27707b-2	0.722
18	220707042HYCPK27685b-2	0.682	41	220720027HYCPK27712b-2	0.790
19	220714025HYCPK27686b-2	0.799	42	220720029HYCPK27713b-2	0.754
20	220714027HYCPK27687b-2	0.673	43	220720031HYCPK27714b-2	0.787
21	220714029HYCPK27688b-2	0.717	44	220720033HYCPK27715b-2	0.720
22	220714031HYCPK27689b-2	0.741	45	220720035HYCPK27716b-2	0.743
23	220714033HYCPK27690b-2	0.742	46	220725017HYCPK27667b-2	0.681

Table S2: List of initial hits from the high-throughput screening with their biological targets.

Compound	Name	Structure	Targets
**2**	THZ1 (Hydrochloride)	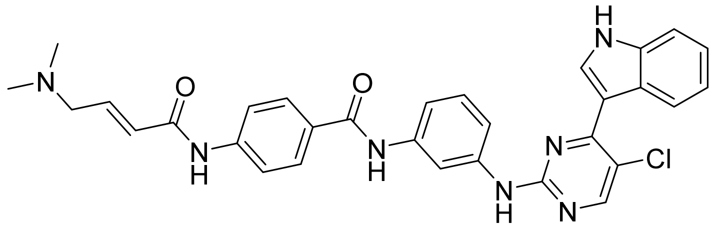	CDK

**3**	CX-6258	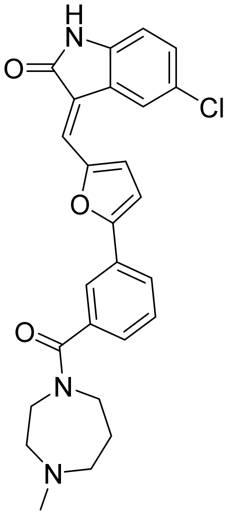	Pim
**4**	KY19382	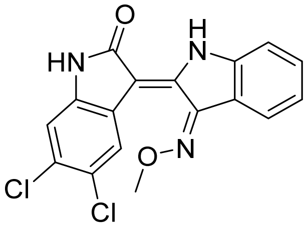	GSK-3; Wnt; β-catenin
**5**	AZ82	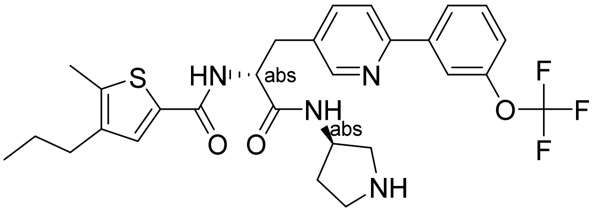	Kinesin
**6**	TG 100572 (Hydrochloride)	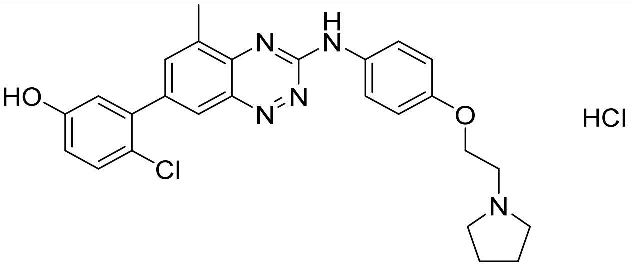	FGFR; PDGFR; Src; VEGFR
**7**	G-749	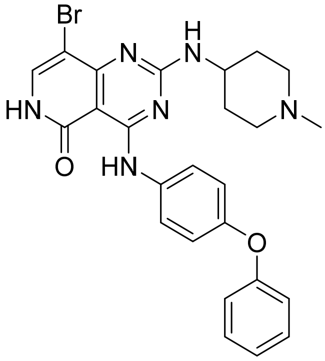	Apoptosis; FLT3

**8**	HM43239	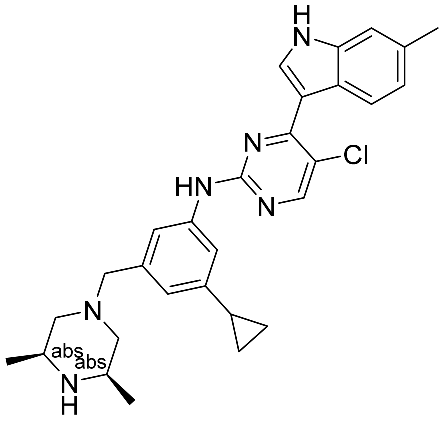	Apoptosis; FLT3
**9**	Eltrombopag	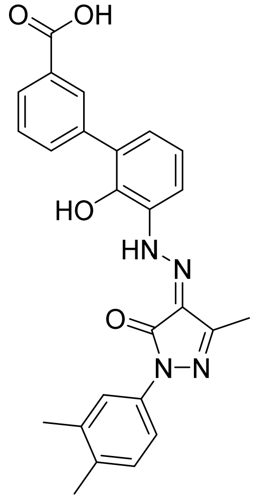	Bacterial; Thrombopoietin Receptor
**10**	Lp-PLA2-IN-3	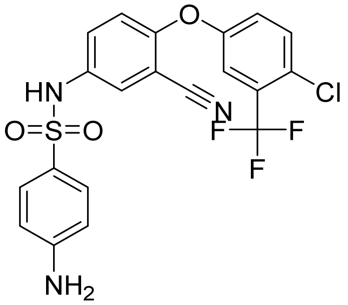	Phospholipase
**1**	SGI-1027	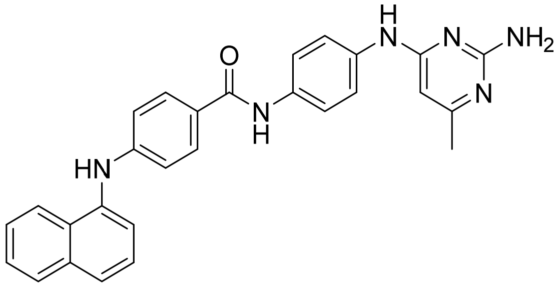	Apoptosis; DNA Methyltransferase
**11**	Walrycin B	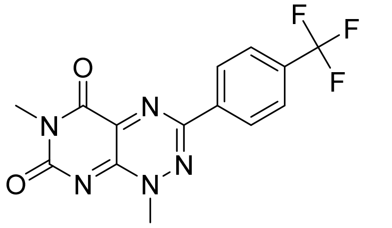	Antibiotic; Bacterial

**12**	BGG463	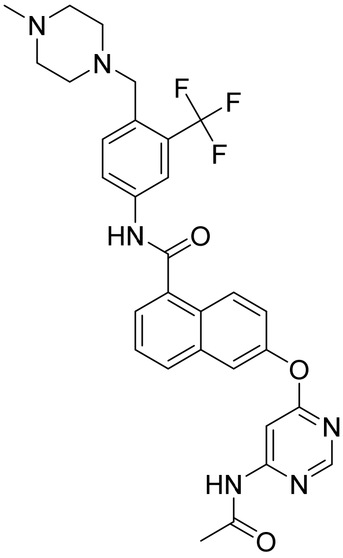	CDK
**13**	Toxoflavin	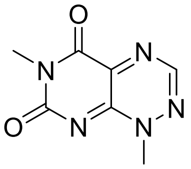	Antibiotic; Bacterial; β-catenin
**14**	PHA-665752	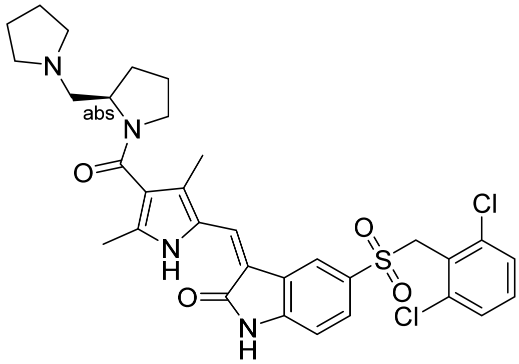	Apoptosis; Autophagy; c-Met/HGFR
**15**	THZ-P1-2	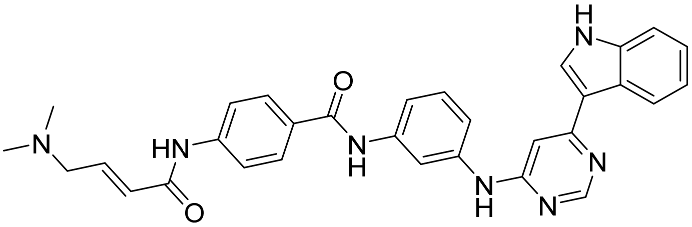	Autophagy
**16**	Peficitinib	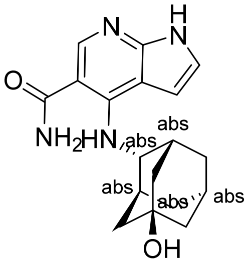	JAK

**17**	B-Raf IN 1	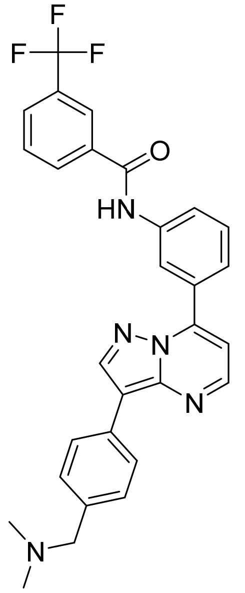	Raf
**18**	Indirubin Derivative E804	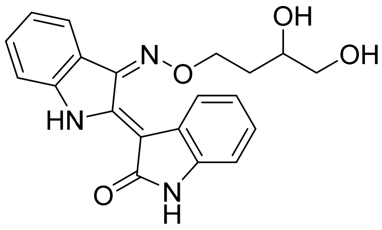	IGF-1R
**19**	SRX3207	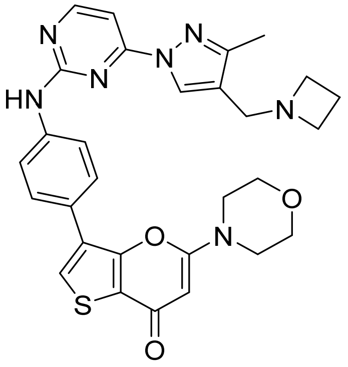	PI3K; Syk
**20**	BAY-850	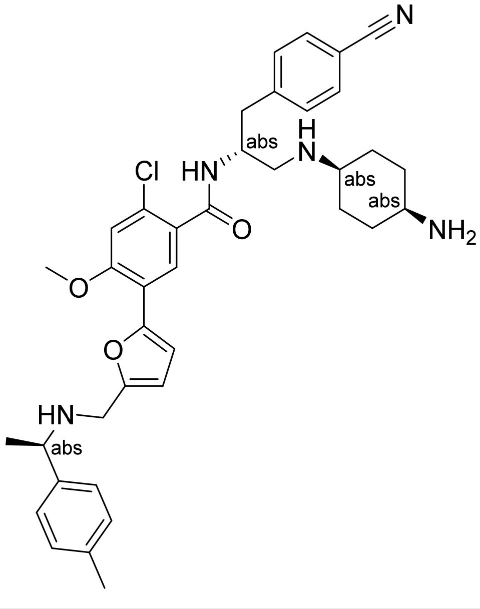	Epigenetic Reader Domain

**21**	Mevociclib	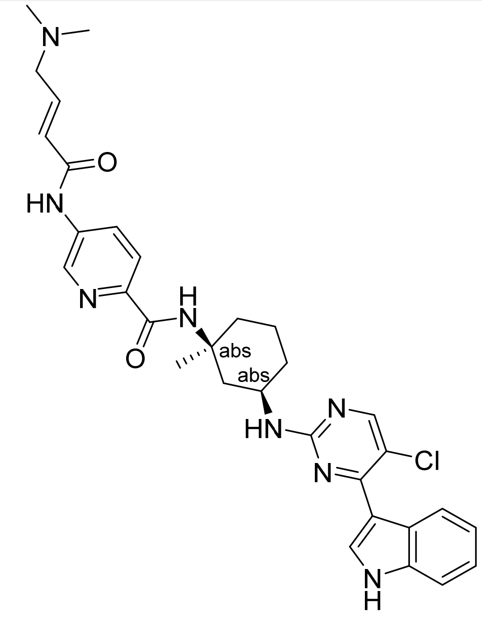	CDK

Table S3: List of oligonucleotide sequences used in this study. All oligonucleotides were obtained from Biomers or Integrated DNA Technologies (IDT).

Oligonucleotide	Sequence 5' - 3'
**RNA1**	GGC ACU CAG ACU CAG AG
**RNA2**	CUC UGA GUA A
**RNA1-BHQ1**	GGC ACU CAG ACU CAG AG-**BHQ1**
**RNA2-FAM**	**FAM**-CUC UGA GUA A
**DNA1**	GTC GGA CTG ATT CGG
**DNA2**	GTG CTG ATG CGT C
**DNA3**	CCG AAT CAG TCC GAC GAC GCA TCA GCA C

3 Methods

3.1 Biochemical experiments

Biological assays were adapted from previously described procedures.[1]

Plasmid construction

The plasmid construct of pET15b-Rlig1 was obtained from Yizhi Yuan and transformed into *Escherichia coli* (*E. coli)* BL21 (DE3) competent cells. The gene fragment of hLig1 bearing 5'-NdeI and 3'-XhoI restriction cleavage sites was synthesized by Integrated DNA technologies. The gene fragment was ligated into pJET1.2/blunt vector using CloneJET PCR Cloning Kit for amplification. The NdeI and XhoI restriction digested gene fragments and pET28a vector were isolated and ligated. The plasmid construct of pET28a-hLig1 was

transformed in Rosetta™ BL21 (DE3) *E. coli* cells. Sequence information is listed in the appendix.

Expression and purification of recombinant proteins

Rlig1-AMP

Rlig1-AMP was expressed and purified from a pET15b vector in BL21 (D3) *E. coli* cells with an N-terminal His-tag according to the protocol developed by Yuan *et al.*[1] Cells were cultured in LB with 100 mg/L carbenicillin overnight at 37°C and 220 rpm. 1 L of LB_{carb} was inoculated with the overnight culture to an OD_{600} of 0.1 and incubated at 37°C and 220 rpm until an OD_{600} of 0.6 was reached. Gene expression was induced with 1 mM IPTG and incubated at 20 °C for 18 hours and 220 rpm. The cells were harvested by centrifugation (4000 rpm, 30 min, 4 °C), resuspended in lysis buffer (50 mM Tris-HCl pH 8.0, 150 mM NaCl, 20 mM imidazole, 1 mM DTT, 0.1% Trition-X100, 1 mg/mL Pefabloc, 1 µg/µL Aprotinin/Leupeptin) and lysed by sonication (5 cycles, 20% intensity, 45 sec) on ice. The lysate was centrifuged (18000 rpm, 30 min, 4 °C) and the supernatant was filtered through a 0.45 µm syringe filter. The AMPylated His_6-Rlig1 was purified using a 5 mL HisTrap™ FF crude column with a step gradient of 10% buffer B (buffer A: 50 mM Tris-HCl pH 8.0, 150 mM NaCl, 20 mM imidazole, 1 mM DTT; buffer B: 50 mM Tris-HCl pH 8.0, 150 mM NaCl, 500 mM imidazole, 1 mM DTT). Fractions were analyzed by SDS-PAGE and fractions containing AMPylated His_6-Rlig1 were pooled and dialyzed against 50 mM Tris-HCl pH 8.0, 100 mM NaCl, 1mM DTT at 4 °C overnight. The protein was further purified using a 5 mL HiTrap™ Q HP column (buffer A: 50 mM Tris-HCl pH 8.0, 100 mM NaCl, and 1.0 mM DTT; buffer B: 50 mM Tris-HCl, 1000 mM NaCl, and 1.0 mM DTT). Fractions were analyzed by SDS-PAGE, and those containing only Rlig1-AMP were pooled, concentrated, and stored at -20 °C in storage buffer containing 50 mM Tris-HCl pH 8.0, 100 mM NaCl, 1 mM DTT and 50% (v/v) glycerol.

hLig1

hLig1 was expressed and purified from a pET28a vector in Rosetta™ BL21 (DE3) *E. coli* cells with an N-terminal His-tag adapted from the protocol of Pascal *et al.*[2] Cells were cultured in LB with 100 mg/mL kanamycin overnight at 37°C and 220 rpm. 1 L of LB_{kana} was inoculated with the overnight culture to an OD_{600} of 0.1 and incubated at 37°C and 220 rpm until an OD_{600} of 0.6 was reached. Gene expression was induced by the addition of 0.5 mM IPTG and the culture was incubated at 30°C for four hours and 220 rpm. The cells were harvested by centrifugation (4000 rpm, 30 min, 4°C), resuspended in 20 mL lysis buffer (50 mM Tris-HOAC [pH 7.5], 100 mM NaCl, 10% glycerol, 1 mM EDTA, 0.1% Triton-X100, 1mM DTT, 10 mM imidazole, 25µL/mL lysozyme, 1 mg/mL Pefabloc, 1 µg/µL Aprotinin/Leupeptin) and lyzed by sonication (5 cycles, 18% intensity, 45 sec) on ice. The lysate was centrifuged (18000 rpm,

30 min, 4°C) and the supernatant was filtered through a 0.45 µm syringe filter. His_6-hLig1 was purified using a 5 mL HisTrap™ FF crude column with a gradient of 0% to 100% buffer B (buffer A: 50 mM Tris-HCl pH 8.0, 150 mM NaCl, 20 mM imidazole, 1 mM DTT; buffer B: 50 mM Tris-HCl pH 8.0, 150 mM NaCl, 500 mM imidazole, 1 mM DTT). Fractions containing His_6-hLig1 were pooled, concentrated and rebuffered to SEC buffer (50 mM Tris-HCl [pH 8], 100 mM NaCl, 1 mM DTT). The protein was further purified with SEC (HiLoad 16/600 Superdex 200 pg, GE Healthcare) using SEC buffer. Fractions containing His_6-hLig1 were pooled, concentrated and stored at -80°C in 25 mM Tris-HCl [pH 8.0], 50 mM NaCl, 0.5 mM DTT and 50% (v/v) glycerol.

High-throughput screening of inhibitors for Rlig1-AMP

The bioactive chemical library was purchased from MedChemExpress (Art.-Nu.: HY-L001) and screened using a fully automated Freedom evo screening unit from Tecan. His_6-Rlig-AMP, which was recombinantly expressed in *E. coli*, was diluted to 750 nM in 1x RNA ligation buffer (50 mM Tris-HCl [pH 7.1], 5 mM $MgCl_2$ and 1 mM DTT). 40 µL of the cooled enzyme solution was dispensed into all wells of columns 1-23 of a 384-well plate (white, non-binding, Greiner) using a multichannel arm (MCA) and an MCA 384 adapter for disposable tips. Column 24 was filled with 40 µL of the same buffer without the enzyme using the four-channel liquid handling arm (LiHa). The library compounds were transferred in a final concentration of 10 µM into all wells of columns 1-24 of the plate using the MCA with a 384 fixed tips tool. The plate was incubated at 37 °C for 10 min with shaking at 8.5 Hz. 5'-Phosphorylated RNA1-BHQ1 (225 nM) and RNA2-FAM (150 nM) were dissolved in 1x RNA ligation buffer and subsequently cooled to 4°C. The BHQ1-labelled RNA was used in excess to ensure efficient quenching of the FAM-labelled strand. A 20 µL aliquot of this solution was dispensed into each well using a Multidrop 384 reagent dispenser (Thermo Scientific). The plate was incubated at 37 °C for 20 minutes. The reactions were stopped by adding 20 µL of an 80 mM EDTA solution in buffer containing 50 mM Tris-HCl (pH 7.1) and incubated at 37 °C for an additional 10 min. Fluorescence intensity was immediately measured using a Spark microplate reader (Tecan) with excitation at 495 nm and detection of fluorescence emission at 520 nm. The compounds were also treated under the same conditions without enzyme, and relative enzymatic activity was calculated as the difference in fluorescence intensity readouts between the two conditions and the controls. Compounds were considered hits if the inhibition was above 70%. Z'-factors were calculated for each plate as quality control, using the mean (µ) and standard deviation (σ) of the positive (c +, 100% activity) and negative (c -, 0% activity) controls. Data analysis was performed using the KNIME Analytics platform.

$$Z' = 1 - \frac{3\sigma_{c+} + 3\sigma_{c-}}{|\mu_{c+} - \mu_{c-}|}$$

Preparation of 5' ^{32}P-labelled oligonucleotides

RNA or DNA oligonucleotides were radioactively labelled by 5'-phosphorylation with [γ-^{32}P]-ATP. 1.0 µM oligonucleotides were incubated with 0.4 u/µL of T4 polynucleotide kinase (PNK) and 200 µM 0.555 MBq γ-^{32}P-ATP (185 TBq/mmol, Hartmann Analytic, SRP-401) in 1x T4 PNK reaction buffer at 37 °C for 1 h in a total volume of up to 50 µL. The reaction was stopped by heating to 95 °C for 2 min. Unreacted ATP was removed by gel filtration using columns packed with 750 µL Superdex™ G-25 Superfine (GE Healthcare) and centrifuged at 3300 rpm for 2 min. The gel filtration material was prepared by suspending 10 g Superdex™ G-25 Superfine in 75 mL H_2O containing 60 mg NaN_3. Prior to filtration, the packed gel filtration columns were washed with 400 µL H_2O, to remove NaN_3.

Radioactive ligation assays

General RNA ligation assay

50 nM 5' ^{32}P-labelled RNA1 and 250 nM unlabelled RNA2 were incubated with 1 µM or 500 nM Rlig1-AMP and 200 µM ATP in 1x RNA ligation buffer (50 mM Tris-HOAc pH 7.0, 5 mM $MgCl_2$, and 1 mM DTT) at 37 °C for 20 min in a total volume of 10 µL. The reactions were stopped by the addition of 90 µL stopping solution (80% (v/v) formamide, 20 mM EDTA, 0.025% (w/v) bromophenol blue, and 0.025% (w/v) xylene cyanol) and heated at 95 °C for 2 min. 1 µL samples were resolved by urea-PAGE and analyzed by autoradiographic imaging.

Hit validation assay and determination of IC_{50} values

Hits from the HTS were purchased from MedChemExpress at a concentration of 10 mM in DMSO. For the hit validation assay, a 1 mM stock solution of compound dissolved in DMSO was used. For the concentration-dependent assay, the compounds were diluted in 1:2 steps, starting at 3 mM and ending at 0.02 mM in DMSO. Before the reaction, 1 µL of the compound in DMSO was diluted in 19 µL Milli-Q water. A 4 µL sample of Mastermix (1.25 µM His_6-Rlig1-AMP in 1.5x RNA ligation buffer with 200 µM ATP) was mixed with 2 µL of diluted compound or 5% DMSO as control. The mixture was preincubated at 37 °C for 10 min. Then, 4 µL of Mastermix (125 nM RNA1, 625 nM RNA2 in 1x RNA ligation buffer) was added, and the mixture was incubated at 37 °C for 20 min. The reactions were stopped by the addition of stopping solution, resulting in 0.005 µM 5'-^{32}P-RNA1, and heated at 95°C for 2 min. The samples were resolved using urea-PAGE and analyzed by autoradiographic imaging. Ligation turnover was quantified by ImageLab (Version 6.0.1) and the IC_{50} value was calculated using a non-linear curve fit/ dose-response curve in GraphPad Prism (Version 10.2.3). All data represent the mean and standard error of triplicates.

*DNA ligation assay with hLig1 and **47***

Compound **47** was diluted in 1:2 steps, starting at 3 mM and ending at 0.02 mM in DMSO. Before the reaction, 1 µL of the dilution series in DMSO was transferred into 19 µL Milli-Q water. A 4 µL sample of Mastermix [1.25 µM hlig1, 500 µM ATP in 1x DNA ligation buffer (50 mM Tris-HOAc [pH 7.5], 50 mM NaCl, 5 mM $MgCl_2$, 0.05 mg/mL BSA and 1 mM DTT)] and 2 µL of compound dilution were mixed and preincubated at 37 °C for 10 min. 4 µL of DNA Mastermix (125 nM pDNA1 , 250 nM DNA2, 250 nM DNA3 in 1x DNA ligation buffer) was added to the samples, followed by incubation at 37°C for 20 min. The reactions were stopped by adding 90 µL stopping solution and heating at 95°C for 2 min. 1 µL samples were resolved via urea-PAGE and analyzed by autoradiographic imaging. Ligation turnover was quantified using ImageLab, and product formation was plotted against the logarithmic compound concentration in GraphPad Prism.

DNA ligation assay with various hLig1 concentration at constant inhibitor concentration

Compounds dissolved in DMSO were 1:20 diluted in Milli-Q water to a concentration of 50 µM. 6 µL of Mastermix with various hLig1 concentration (5, 2.5, 1.25, and 0.625 µM in 1 x DNA ligation buffer) and 2 µL of Mastermix (16.6 µM compound, 333 µM ATP in 1.25x DNA ligation buffer) were incubated at 37 °C for 30 min. Following preincubation, 2 µL DNA Mastermix (500 nM DNA1, 1.00 µM DNA2, 1.00 µM DNA3 in Milli-Q water) was added, and the samples were incubated at 37 °C for 30 min. The reactions were stopped by adding 90 µL stopping solution and heating at 95°C for 2 min. 1 µL samples were resolved via urea-PAGE and analyzed by autoradiographic imaging. Ligation turnover was quantified using ImageLab, and product formation at the indicated compound concentrations was visualized in GraphPad Prism.

Urea-PAGE and autoradiographic imaging

For the separation of oligonucleotide samples, a denaturing 12 % urea-PAGE sequencing gel was used. 1 µL of the denatured sample was loaded onto a pre-warmed urea-PAGE sequencing gel. Electrophoresis was performed in 1x TBE buffer (90 mM Tris-HCl [pH 8.0], 90 mM boric acid, 2.0 mM EDTA) at 100 W and a maximal temperature of 45 °C for 45 min. The gel was transferred onto a Whatman filter paper and dried at 80 °C for 2 h under vacuum. After cooling to room temperature, the gel was exposed to a storage phosphor screen overnight. The autoradiographic imaging was performed by Typhoon FLA-9500 (GE Healthcare) in phosphorimaging mode. Gels were further analyzed using ImageLab.

3.2 Cell culture

Multipipetting was performed using Integra Viaflo or Integra Voyager pipettes. For automated multipipetting, an Integra Assist Plus pipetting robot was employed.
HEK293 cells were maintained in DMEM GlutaMAX™ medium (Gibco™) supplemented with 10% (v/v) FCS. Cells were cultured at 37 °C in an humidified incubator with 7.5% CO_2.

Cell viability assay of HEK293 cells treated with inhibitors

For the cell viability assay, cells were seeded at a density of $1x10^4$ cells per well in a black 384-well plate (µCLEAR®, cell culture, Greiner) in a total volume of 40 µL medium. The plate was incubated at 37 °C, 7.5% CO_2, and 95% humidity for 24 h. Stock solutions of the compounds were prepared in DMSO at a concentration of 10 mM and 1:2 diluted in DMSO in a 96-well plate. Prior to addition to the cells, the compounds were further diluted in medium. Then, 20 µL of the compound dilution was added to the corresponding wells of the cell plate, resulting in a final volume of 60 µL per well and 0.9% DMSO. The plate was incubated for an additional 24 h at 37 °C. Cell viability was assessed using the CellTiter-Glo® 2.0 Cell Viability Assay (Promega). Upon addition of 15 µL of CellTiterGlo® reagent per well using the Multidrop 384 reagent dispenser (Thermo Scientific), the plate was centrifuged for 1 min at 800 rpm and incubated for 40 min on a shaker at 25°C and 800 rpm. The luminescence was read out at 25 °C with a Spark microplate reader (Tecan). The luminescence values of the treated cells were compared to the value of the cells treated with DMSO as control (equals 100% viability). The EC_{50} values were determined using GraphPad Prism software by fitting the data using a non-linear curve fit/ dose-response curve (fitting with a variable slope with four parameters logistic). All data represent mean ± standard error of three independent experiments consisting of four technical replicates each.

Cell viability assay of HEK293 cells treated with inhibitors and menadione

1000x menadione stock solutions were prepared in DMSO and frozen in aliquots. Prior to treatment, aliquots were thawed and diluted in medium to 4-fold concentration. Upon 24 h treatment with the inhibitors at the indicated concentrations, 20 µL of menadione in medium was added to a final menadione concentration of 40 µM or 60 µM in 80 µL total volume. For the control wells with and without compound, 20 µL of DMSO in medium were added. The plate was incubated at 37 °C, 7.5% CO_2, and 95% humidity for 5 h. Subsequently, 40 µL was removed from each well. Cell viability was assessed using the CellTiter-Glo® 2.0 Cell Viability Assay (Promega) as described before. The percentual cell viability was normalized to the control wells with and without menadione and graphs were plotted using GraphPad Prism software. All data represent mean ± standard error of four independent experiments consisting of four technical replicates each.

Light-microscopy of inhibitor- and menadione-treated HEK293 cells

5 x 10^5 cells were seeded in 1.5 mL DMEM GlutaMAX™ medium supplemented with 10% (v/v) FCS in a 6-well plate (Sarstedt). The plate was incubated at 37 °C, 7.5% CO_2, and 95% humidity for 24 h. The cells were treated with 250 µL medium with either DMSO (control) or 30 µM compound **1**, **47** or **26**. After 24 h, 250 µL medium with either DMSO (control) or menadione in DMSO was added to a final concentration of 60 µM. After 5 h, pictures were taken with a light microscope using a 10x objective with 25 mm field of view.

RNA integrity analysis of HEK293 cells treated with inhibitors and various concentrations of menadione

5.0 x 10^5 cells were seeded in 1.5 mL DMEM GlutaMAX™ medium supplemented with 10% (v/v) FCS in a 6-well plate (Sarstedt). The plate was incubated at 37 °C, 7.5% CO_2, and 95% humidity for 24 h. Compound stocks at 10 mM in DMSO were pre-diluted in medium before being added to the cells. The cells were treated with 250 µL of the compound dilution to a final concentration of 30 µM. DMSO was used as a control. The plate was incubated for an additional 24 h at 37 °C. 1000x menadione stock solution in DMSO was diluted in medium and 250 µL were added to a final menadione concentration of 40 µM or 60 µM. After 5 h, the cells were detached from the surface by vigorously pipetting up and down. The cell suspensions were then transferred to a 2 mL Eppendorf tube and centrifuged (350 g, 5 min, 4 °C). The cell pellets were washed with 1 mL DPBS (Gibco™), centrifuged again (350 g, 5 min, 4 °C) and snap-frozen in liquid nitrogen. For the RNA extraction, 600 µL TRIzol Reagent (Invitrogen) were added, and the pellets were resuspended by vortexing for 10 min at r.t. After 5 min incubation at r.t., 150 µL chloroform were added, the samples were vortexed for 1 min and centrifuged (12000 g, 15 min, 4 °C). The upper aqueous phases were transferred into new Eppendorf tubes and 150 µL chloroform were added. The samples were vortexed for 1 min, incubated for 5 min at r.t. and centrifuged (12000 g, 15 min, 4 °C). The upper clear phases were transferred into new Eppendorf tubes and 360 µL ice-cold isopropanol were added. The samples were mixed by shaking and stored for 24 h at -80 °C. The samples were centrifuged (20700 g, 30 min, 4 °C) and the supernatant was discarded. The RNA pellet was resuspended in 600 µL ice-cold ethanol and centrifuged (20700 g, 10 min, 4 °C). The supernatant was discarded and the ethanol washing step was repeated once again. The pellet was dried on air for 10 min and then dissolved in 40 µL Milli-Q. RNA concentration was determined by Nanodrop (Thermo Scientific) and 250 ng/µL RNA were analyzed according to the manufacturer instructions on an Agilent 4150 TapeStation system. Experiments with DMSO and 40 µM menadione were conducted in triplicate, while those with 60 µM menadione were performed in duplicate.

3.3 Chemical synthesis

General experimental details

Commercially available starting materials, reagents and anhydrous solvents were obtained from commercial suppliers (Sigma Aldrich, Merck, VWR chemicals, Carl Roth, Thermo Fisher Scientific, Fluka, TCI, ABCR and aablocks) and used without further purification if not stated otherwise. Dry solvents were purchased. For chemical synthesis desalted water was used. All air or water sensitive reactions were carried out under nitrogen atmosphere in dried reaction flasks.

The final compounds were further purified by precipitation. Therefore, the compound was diluted in a minimal amount of iso-propanol (2-5 mL) under reflux. The solution was transferred to a 50 mL falcon and 15-20 mL ice-cold water were added. The falcon was centrifuged (4000 rpm, 20 min, 4 °C), the precipitate was washed with 15 mL water and centrifuged again (4000 rpm, 20 min, 4 °C). The compound was dried under reduced pressure.

Analytical methods

Analytical thin layer chromatography (TLC)

Analytical thin layer chromatography was recorded on silica gel plates (aluminum) from *Merck* (Type 60-F254, 0.2 mm silica). For visualization, TLC plates were exposed to UV-light (λ = 254 nm), and/or stained with $KMnO_4$ (1.50 g $KMnO_4$, 10.0 g K_2CO_3 and 1.25 mL 10% NaOH in 200 mL water) or ninhydrin (1 g ninhydrin in 100 mL EtOH) with subsequent heat treatment (ca. 250 °C).

Nuclear magnetic resonance spectroscopy (NMR)

NMR spectra were recorded at room temperature on Bruker Avance III 400 (400 MHz), Bruker Avance III 500 (500 MHz) or Bruker Avance III 600 (600 MHz) spectrometers. The chemical shifts (δ) are reported in parts per million (ppm) and ^1H, ^{13}C and ^{19}F spectra are referenced to residual proton and carbon signals of the deuterated solvents MeOD and DMSO-d_6. Multiplicities are denoted as s = singlet, d = doublet, t = triplet, q = quartet, p = quintet, dd = doublet of doublet, dt = doublet of triplet, m = multiplet, br = broad. NMR data were processed by MestReNova (version 14.2.0-26256). The coupling constants, J, are reported in Hertz (Hz). Since some signals could not be assigned clearly, they were marked with an asterisk (*); the corresponding assignments are interchangeable.

Mass-spectrometry

High resolution mass-spectrometry (HR-MS) were performed by QTOF 6546 (ESI-TOF) from Agilent or TQ-Orbitrap Discovery mass spectrometer from Thermo Scientific.

General procedures

The procedures were adapted from published protocols.[3-4]

<u>*General procedure A: Boc-protection of aryl amines*</u>

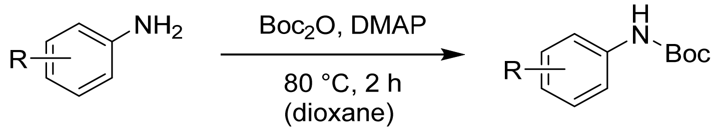

To a stirred solution of aryl amine (1.00 eq) in anhydrous dioxane was added a solution of di-*tert*-butyl dicarbonate (1.00 or 2.00 eq) in 1 mL anhydrous dioxane and 4-dimethylaminopyridine (0.100 eq). The reaction mixture was stirred at 80 °C for 2-4 hours. Upon cooling to room temperature, the reaction was stopped with sat. $NaHCO_3$ solution and extracted with EtOAc (3 x 100 mL). The combined organic layer was dried over $MgSO_4$, filtered and concentrated. Each residue was purified by silica gel column chromatography to afford the Boc protected aryl amine.

tert-Butyl (_tert_-butoxycarbonyl)(5-chloro-2-fluorophenyl)carbamate & _tert_-butyl (5-chloro-2-fluorophenyl)carbamate (48)

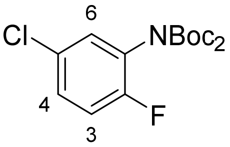

$C_{16}H_{21}ClFNO_4$
345.80 g/mol

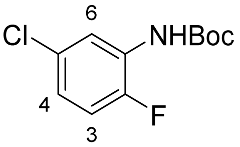

$C_{11}H_{13}ClFNO_2$
245.68 g/mol

88% **12%**

Following general procedure A, 5-chloro-2-fluoroaniline (350 mg, 2.10 mmol, 1.00 eq), Boc_2O (916 mg, 4.20 mmol, 2.00 eq) and DMAP (24.0 mg, 196 µmol, 0.10 eq) in 5 mL dioxane was stirred at 80 °C for 4 h. The reaction mixture was purified by extraction and silica gel column chromatography [10% → 100% EtOAc /pentane] to provide **48** (485 mg, 1.46 mmol, 70%) as a yellow solid.

^{1}H-NMR (DMSO, 400 MHz) δ = 7.60 (dd, J = 6.7, 2.7 Hz, 1H, H-6), 7.52 – 7.44 (m, 1H, H-3*), 7.42 – 7.32 (m, 1H, H-4*), 1.37 (s, 18H, Boc_2).

tert-Butyl (tert-butoxycarbonyl)(5-chloro-3-fluorophenyl)carbamate & _tert_-butyl (5-chloro-3-fluorophenyl)carbamate (49)

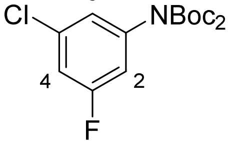

$C_{16}H_{21}ClFNO_4$
345.80 g/mol

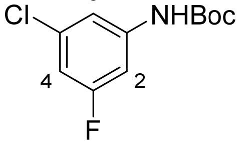

$C_{11}H_{13}ClFNO_2$
245.68 g/mol

80% **20%**

Following general procedure A, 3-chloro-5-fluoroaniline (340 mg, 2.34 mmol, 1.00 eq), Boc_2O (1.00 g, 4.60 mmol, 2.00 eq) and DMAP (28.5 mg, 233 µmol, 0.10 eq) in 5 mL dioxane was stirred at 80 °C for 2 h. The reaction mixture was purified by silica gel column chromatography [10% EtOAc /pentane] to provide **49** (655 mg, 2.05 mmol, 88%) as a yellow solid.
^{1}H-NMR (DMSO, 400 MHz) δ = 7.44 (dt, J = 8.7, 2.1 Hz, 1H, H-6), 7.31 – 7.28 (m, 2H, H-2, H-4), 1.39 (s, 18H, Boc_2).

General procedure B: Buchwald-Hartwig amination

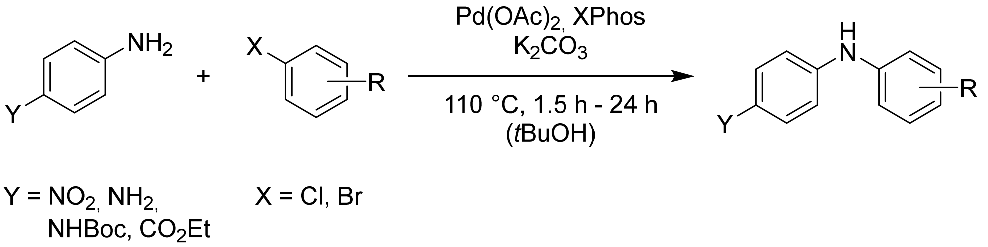

An oven-dried 25 mL flask with $Pd(OAc)_2$ (2 mol%) and XPhos (6 mol%) was evacuated and flushed with nitrogen trice. Degassed *tert*-butanol (*t*BuOH) and degassed water (2 mol%) were added and the suspension was heated to 80 °C for one minute. Subsequently, the aryl halide (1.00 eq), the aryl amine (1.20 eq) and K_2CO_3 (1.45 eq) were added, and the reaction mixture was stirred at 110 °C for 1.5 – 24 hours. Completion of the reaction was monitored by TLC. Upon cooling to room temperature, the suspension was diluted with EtOAc and sat. $NaHCO_3$ solution and extracted with EtOAc (3 x 100 mL). The combined organic layer was dried over $MgSO_4$, filtered and concentrated. Each residue was purified by silica gel column chromatography to afford the coupled aryl amine compound.

Ethyl 4-(quinolin-4-ylamino)benzoate (50)

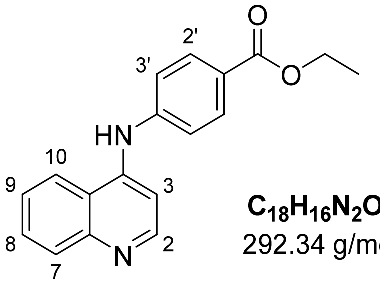

$C_{18}H_{16}N_2O_2$
292.34 g/mol

Following general procedure B, a mixture of ethyl-4-aminobenzoate (500 mg, 3.03 mmol, 1.24 eq), 4-chloroquinoline (320 µL, 400 mg, 2.44 mmol, 1.00 eq), $Pd(OAc)_2$ (12.0 mg, 50.0 µmol, 0.02 eq), XPhos (59.0 mg, 120 µmol, 0.05 eq), K_2CO_3 (476 mg, 3.44 mmol, 1.41 eq) in 4 mL *t*BuOH and 3 µL water was stirred at 110 °C for three hours. The product **50** was isolated by silica column chromatography [DCM, 1% TEA → DCM, 0.5% MeOH 1% TEA] as a light yellow solid (669 mg, 2.29 mmol, 94%).
TLC: R_f = 0.38 (5% MeOH/1%TEA/ DCM) [UV].

^{1}**H-NMR** (DMSO-d_6, 400 MHz) δ= 9.32 (s, 1H, NH), 8.60 (d, J = 5.14 Hz, 1H, H-2), 8.34 (d, J = 8.39 Hz, 1H, H-10), 7.94 (d, J = 8.44 Hz, 2H, H-2'), 7.93 (t, J = 8.26 Hz, 1H, H-7), 7.74 (t, J = 7.63 Hz, 1H, H-8), 7.58 (d, J = 7.63 Hz, 1H, H-9), 7.44 (d, J = 8.44 Hz, 2H, H-3'), 7.27 (d, J = 5.14 Hz, 1H, H-3), 4.29 (q, J = 7.11 Hz, 2H, CH_2), 1.32 (t, J = 7.11 Hz, 3H, CH_3).
These data are in accordance with previous literature reports.[3]

6-Methyl-*N*⁴-(4-nitrophenyl)pyrimidine-2,4-diamine (51)

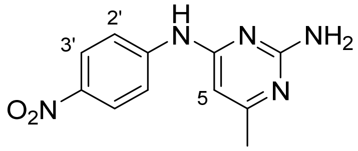

$C_{11}H_{11}N_5O_2$
245.24 g/mol

Following general procedure B, 4-nitroaniline (2.35 g, 17.0 mmol, 1.22 eq), 2-amino-4-chloro-6-methyl pyrimidine (2.00 g, 13.9 mmol, 1.00 eq), $Pd(OAc)_2$ (40.0 mg, 180 µmol, 0.013 eq), XPhos (213 mg, 450 µmol, 0.032 eq) and K_2CO_3 (2.80 g, 20.3 mmol, 1.45 eq) in 28 mL *t*BuOH and 9 µL water was stirred at 110 °C for 90 minutes. The product was isolated by silica column chromatography [0.5% MeOH/ DCM, 1% TEA → DCM, 5% MeOH 1% TEA] as a yellow solid (1.78 g, 7.23 mmol, 52%).
TLC: R_f = 0.66 (5% MeOH/1%TEA/ DCM) [UV].
^{1}**H-NMR** (DMSO-d_6, 400 MHz) δ = 9.78 (s, 1H, NH), 8.13 (d, J = 9.30 Hz, 2H, H-3'), 8.01 (d, J = 9.30 Hz, 2H, H-2'), 6.40 (s, 2H, NH_2), 5.99 (s, 1H, H-5), 2.14 (s, 3H, CH_3).
These data are in accordance with previous literature reports.[3]

***N*⁴-(4-Nitrophenyl)pyrimidine-2,4-diamine (52)**

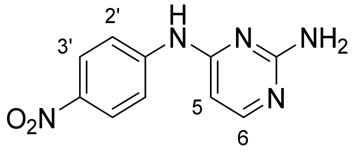

$C_{10}H_9N_5O_2$
231.22 g/mol

Following general procedure B, 4-nitroaniline (320 mg, 2.32 mmol, 1.22 eq), 2-amino-4-chloro-pyrimidine (250 mg, 1.93 mmol, 1.00 eq) $Pd(OAc)_2$ (5.60 mg, 25.0 µmol, 0.013 eq), XPhos (29 mg, 61.0 µmol, 0.032 eq) and K_2CO_3 (386 mg, 2.79 mmol, 1.45 eq) in 4 mL *t*BuOH and 2 µL water was stirred at 110 °C for 90 minutes. Purification by silica gel column chromatography afforded [0.5% MeOH/1% TEA/ DCM → 3% MeOH/1% TEA / DCM] compound **52** (228 mg, 98.4 µmol, 51%) as a yellow solid.
TLC: R_f = 0.38 (5% MeOH/1%TEA/ DCM) [UV].
^{1}**H-NMR** (DMSO, 400 MHz) δ = 9.89 (s,1H,NH), 8.14 (d, J 9.34 Hz,2H, H-3'),8.03 (d, J = 9.34 Hz, 2H, H-2'),7.95 (d, J = 5.62 Hz, 1H, H-6), 6.12 (d, J = 5.62 Hz, 1H, H-5).
^{13}**C-NMR** (DMSO, 101 MHz) δ = 162.84, 160.15, 157.39, 147.34, 140.18, 124.85, 118.08, 97.66.
HR-MS m/z calcd. for $C_{10}H_{10}N_5O_2^+$ $(M + 1)^+$ 232.0834, found 232.0841.

N-(4-Nitrophenyl)-5-(trifluoromethyl)pyridin-2-amine (53)

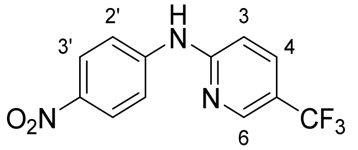

$C_{12}H_8F_3N_3O_2$
283.21 g/mol

Following general procedure B, 4-nitroaniline (232 mg, 1.68 mmol, 1.22 eq), 2-chloro-5-(trifluoromethyl)pyridine (250 mg, 1.38 mmol, 1.00 eq), $Pd(OAc)_2$ (4.00 mg, 18.0 µmol, 0.013 eq), XPhos (21.0 mg, 44.0 µmol, 0.032 eq) and K_2CO_3 (276 mg, 2.00 mmol, 1.45 eq) in 5 mL *t*BuOH and 4 µL water was stirred at 110 °C for three hours. The reaction mixture was quenched with 10 mL $NaHCO_3$ solution and extracted with EtOAc (3 x 100 mL). Purification by silica gel column chromatography [0.5% TEA/ DCM] afforded **53** (123 mg, 441 µmol, 32%) as yellow solid.

TLC: R_f = 0.49 (1%TEA/ DCM) [UV].

^{1}H-NMR (DMSO, 400 MHz) δ = 8.53 (s, 1H, H-6), 8.18 (d, J = 9.28 Hz, 2H, H-3‘), 7.95 (d, J = 9.28 Hz, 2H, H-2‘), 7.86 (dd, J = 2.54, 8.78 Hz, 1H, H-4), 7.00 (d, J = 8.78 Hz, 1H, H-3).

^{13}C-NMR (DMSO, 101 MHz) δ = 158.74, 148.39, 146.15, 142.59, 135.68, 125.95, 118.84, 112.82.

HR-MS m/z calcd. for $C_{12}H_9F_3N_3O_2^+$ $(M + 1)^+$ 284.0641, found 284.0649.

4-Nitro-*N*-(4-(trifluoromethyl)phenyl)aniline (54)

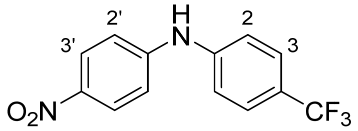

$C_{13}H_9F_3N_2O_2$
282.22 g/mol

Following general procedure B, 4-nitroaniline (229 mg, 1.66 mmol, 1.20 eq), 1-chloro-4-(trifluoromethyl)benzene (250 mg, 1.38 mmol, 1.00 eq), $Pd(OAc)_2$ (7.00 mg, 30.0 µmol, 0.02 eq), XPhos (38.0 mg, 80.0 µmol, 0.06 eq) and K_2CO_3 (276 mg, 2.00 mmol, 1.45 eq) in 3 mL *t*BuOH and 2 µL water was stirred at 110 °C for three hours. The reaction mixture was quenched with 10 mL $NaHCO_3$ solution and extracted with EtOAc (3 x 100 mL). Purification by silica gel column chromatography [5%EtOAc/1%TEA/pentane → 15%EtOAc/1%TEA/pentane] afforded **54** (321 mg, 1.15 mmol, 83%) as yellow solid.

TLC: R_f = 0.55 (20% EtOAc/80% pentane) [UV].

^{1}H-NMR (DMSO, 400 MHz) δ = 9.63 (s, 1H, NH), 8.15 (d, J = 9.24 Hz, 2H, H-3‘), 7.68 (d, J = 8.45 Hz, 2H, H-3), 7.39 (d, J = 8.45 Hz, 2H, H-2), 7.24 (d, , J = 9.24 Hz, 2H, H-2‘).

^{13}C-NMR (DMSO, 101 MHz) δ = 149.02, 144.34, 139.33, 126.74, 125.99, 118.85, 115.20.

HR-MS m/z calcd. for $C_{13}H_{10}F_3N_2O_2^+$ $(M + 1)^+$ 283.0689, found 283.0688.

N-(4-Nitrophenyl)-2-(trifluoromethyl)aniline (55)

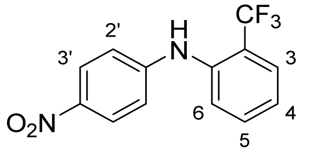

$C_{13}H_9F_3N_2O_2$
282.22 g/mol

Following general procedure B, 4-nitroaniline (229 mg, 1.66 mmol, 1.20 eq), 1-chloro-2-(trifluoromethyl)benzene (186 µL, 250 mg, 1.38 mmol, 1.00 eq), $Pd(OAc)_2$ (7.00 mg, 30.0 µmol, 0.02 eq), XPhos (38.0 mg, 80.0 µmol, 0.06 eq) and K_2CO_3 (276 mg, 2.00 mmol, 1.45 eq) in 3 mL *t*BuOH and 2 µL water was stirred at 110 °C for two hours. The reaction mixture was quenched with 10 mL $NaHCO_3$ solution and extracted with EtOAc (3 x 100 mL). Purification by silica gel column chromatography [1% TEA/pentane → 20% EtOAc/1% TEA/pentane] afforded **55** (366 mg, 1.30 mmol, 94%) as yellow, crystalline solid.

TLC: R_f = 0.86 (20% EtOAc/80% pentane) [UV].

^{1}H-NMR (DMSO, 400 MHz) δ = 8.87 (s, 1H, NH), 8.09 – 8.00 (m, 2H, H-3‘), 7.83 (d, J = 7.7 Hz, 1H, H-3), 7.74 (t, J = 7.8 Hz, 1H, H-5), 7.54 (d, J = 7.8 Hz, 1H, H-6), 7.50 (t, J = 7.6 Hz, 1H, H-4), 6.84 – 6.76 (m, 2H, H-2‘).

^{13}C-NMR (DMSO, 101 MHz) δ= 152.82, 138.04, 137.98, 134.09, 129.00, 127.25, 126.69, 126.05, 113.19.

HR-MS m/z calcd. for $C_{13}H_{10}F_3N_2O_2^+$ $(M + 1)^+$ 283.0689, found 283.0691.

4-Methyl-*N*-(4-nitrophenyl)aniline (56)

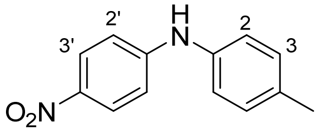

$C_{13}H_{12}N_2O_2$
228.25 g/mol

Following general procedure B, 4-nitroaniline (327 mg, 2.37 mmol, 1.20 eq), 4-chlorotoluene (250 mg, 1.97 mmol, 1.00 eq), $Pd(OAc)_2$ (9.00 mg, 40.0 µmol, 0.02 eq), XPhos (57.0 mg, 120 µmol, 0.06 eq) and K_2CO_3 (395 mg, 2.86 mmol, 1.45 eq) in 3 mL *t*BuOH and 2 µL water was stirred at 110 °C for 3 hours. The reaction mixture was quenched with 15 mL $NaHCO_3$ solution and extracted with EtOAc (3 x 100 mL). Purification by silica gel column chromatography [5% EtOAc/1% TEA/pentane → 10% EtOAc/1% TEA/pentane] afforded **56** (369 mg, 1.61 mmol, 82%) as orange solid.

TLC: R_f = 0.66 (20% EtOAc/80% pentane) [UV].

^{1}H-NMR (DMSO, 400 MHz) δ = 9.21 (s, 1H, NH), 8.06 (d, J = 9.3 Hz, 2H, H-3‘), 7.20 (d, J = 8.4 Hz, 2H, H-3), 7.13 (d, J = 8.4 Hz, 2H, H-2), 6.98 (d, J = 9.3 Hz, 2H, C-2‘), 2.29 (s, 3H, CH_3).

^{13}C-NMR (DMSO, 101 MHz) δ = 151.34, 137.50, 137.30, 132.91, 129.96, 126.23, 121.46, 112.89, 20.47.

HR-MS m/z calcd. for $C_{13}H_{13}N_2O_2^+$ $(M + 1)^+$ 229.0972, found 229.0974.

4-Nitro-*N*-phenylaniline (57)

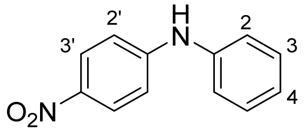

$C_{12}H_{10}N_2O_2$
214.22 g/mol

Following general procedure B, 4-nitroaniline (368 mg, 2.66 mmol, 1.20 eq), chlorobenzene (250 mg, 2.22 mmol, 1.00 eq), $Pd(OAc)_2$ (10.0 mg, 44.0 µmol, 0.02 eq), XPhos (63.0 mg, 133 µmol, 0.06 eq) and K_2CO_3 (445 mg, 3.22 mmol, 1.45 eq) in 3 mL *t*BuOH and 2 µL water was stirred at 110 °C for 2.5 hours. The reaction mixture was quenched with 15 mL $NaHCO_3$ solution and extracted with EtOAc (3 x 100 mL). Purification by silica gel column chromatography [1% TEA/pentane → 20% EtOAc/1% TEA/pentane] afforded **57** (347 mg, 1.62 mmol, 73%) as yellow crystals.

TLC: R_f = 0.59 (20% EtOAc/80% pentane) [UV].

^{1}H-NMR (DMSO, 400 MHz) δ = 9.30 (s, 1H, NH), 8.09 (d, J = 9.3 Hz, 2H, H-3‘), 7.39 (dd, J = 8.5, 7.3 Hz, 2H, H-3), 7.24 (d, J = 8.5, 1.2 Hz, 2H, H-2), 7.10 (t, J = 7.3 Hz, 1H, H-4), 7.06 (d, J = 9.3 Hz, 2H, H-2‘).

^{13}C-NMR (DMSO, 101 MHz) δ = 150.81, 140.05, 137.90, 129.53, 126.20, 123.45, 120.85, 113.35.

HR-MS m/z calcd. for $C_{12}H_{11}N_2O_2^+$ (M + 1)$^+$ 215.0815, found 215.0818.

4-Methoxy-*N*-(4-nitrophenyl)aniline (58)

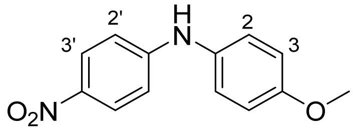

$C_{13}H_{12}N_2O_3$
244.25 g/mol

Following general procedure B, 4-nitroaniline (290 mg, 2.10 mmol, 1.20 eq), 4-chloroanisole (250 mg, 1.73 mmol, 1.00 eq), $Pd(OAc)_2$ (8.00 mg, 34.6 µmol, 0.02 eq), XPhos (50.0 mg, 104 µmol, 0.06 eq) and K_2CO_3 (346 mg, 2.50 mmol, 1.45 eq) in 3 mL *t*BuOH and 2 µL water was stirred at 110 °C for two hours. The reaction mixture was quenched with 15 mL $NaHCO_3$ solution and extracted with EtOAc (3 x 100 mL). Purification by silica gel column chromatography [5% EtOAc/1% TEA/pentane → 15% EtOAc/1% TEA/pentane] afforded **58** (300 mg, 1.22 mmol, 71%) as orange solid.

TLC: R_f = 0.33 (20% EtOAc/80% pentane) [UV].

^{1}H-NMR (DMSO, 400 MHz) δ = 9.11 (s, 1H, NH), 8.04 (d, J = 9.3 Hz, 2H, H-3‘), 7.18 (d, J = 8.9 Hz, 2H, H-2), 6.98 (d, J = 8.9 Hz, 2H, H-3), 6.88 (d, J = 9.3 Hz, 2H, H-2‘), 3.76 (s, 3H, CH3).

^{13}C-NMR (DMSO, 101 MHz) δ = 156.15, 152.14, 137.12, 132.51, 126.31, 124.02, 114.80, 112.29, 55.29.

These data are in accordance with previous literature reports. Literature spectra were recorded in deuterated $CDCl_3$.[5]

4-Fluoro-*N*-(4-nitrophenyl)aniline (59)

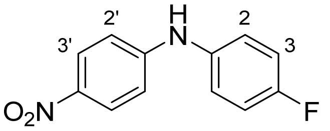

$C_{12}H_9FN_2O_2$
232.21 g/mol

Following general procedure B, 4-nitroaniline (316 mg, 2.29 mmol, 1.20 eq), 1-chloro-4-fluorobenzene (204 µL, 250 mg, 1.91 mmol, 1.00 eq), $Pd(OAc)_2$ (9.00 mg, 38.2 µmol, 0.02 eq), XPhos (55.0 mg, 115 µmol, 0.06 eq) and K_2CO_3 (381 mg, 2.76 mmol, 1.45 eq) in 3 mL *t*BuOH and 2 µL water was stirred at 110 °C for two hours. The reaction mixture was quenched with 15 mL $NaHCO_3$ solution and extracted with EtOAc (3 x 100 mL). Purification by silica gel column chromatography [7.5% EtOAc/1% TEA/pentane → 10% EtOAc/1% TEA/pentane] afforded **59** (288 mg, 1.24 mmol, 65%) as orange solid.

TLC: R_f = 0.60 (20% EtOAc/80% pentane) [UV].

^{1}H-NMR (DMSO, 400 MHz) δ = 9.25 (s, 1H, NH), 8.08 (d, J = 9.3 Hz, 2H, H-3'), 7.35 – 7.14 (m, 4H, H-2, H-3), 6.98 (d, J = 9.3 Hz, 2H, H-2').

^{13}C-NMR (DMSO, 101 MHz) δ = 151.22, 137.82, 136.30, 126.23, 123.49, 123.41, 116.33, 116.11, 112.95.

HR-MS m/z calcd. for $C_{12}H_{10}FN_2O_2^+$ $(M + 1)^+$ 233.0721, found 233.0722.

***N*²-(4-Nitrophenyl)pyridine-2,6-diamine (60)**

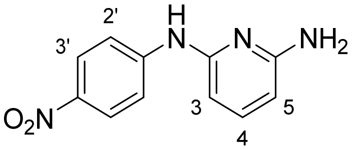

$C_{11}H_{10}N_4O_2$
230.23 g/mol

Following general procedure B, 4-nitroaniline (383 mg, 2.77 mmol, 1.20 eq), 2-amino-6-bromopyridine (400 mg, 2.31 mmol, 1.00 eq), $Pd(OAc)_2$ (10.0 mg, 46.0 µmol, 0.02 eq), XPhos (66.0 mg, 139 µmol, 0.06 eq) and K_2CO_3 (463 mg, 3.35 mmol, 1.45 eq) in 5 mL *t*BuOH and 3.5 µL water was stirred at 110 °C for four hours. The reaction mixture was quenched with 15 mL $NaHCO_3$ solution and extracted with EtOAc (3 x 100 mL). Purification by silica gel column chromatography [20% EtOAc → 50% EtOAc/1% TEA/pentane] afforded **60** (222 mg, 1.18 mmol, 51%) as orange solid.

TLC: R_f = 0.52 (50% EtOAc/1% TEA/ pentane) [UV].

^{1}H-NMR (DMSO, 400 MHz) δ = 9.56 (s, 1H, NH), 8.08 (d, J = 9.4 Hz, 2H, H-3'), 7.91 (d, J = 9.4 Hz, 1H, H-2'), 7.30 (t, J = 7.9 Hz, 1H, H-4), 6.11 (d, J = 7.7 Hz, 1H, H-3), 6.02 (d, J = 7.9 Hz, 1H, H-5), 5.93 (s, 2H, NH_2).

^{13}**C-NMR** (DMSO, 101 MHz) δ = 158.47, 153.27, 148.96, 138.79, 138.50, 125.15, 116.16, 99.93, 99.13.

***N*⁴-(4-Nitrophenyl)pyridine-2,4-diamine (61)**

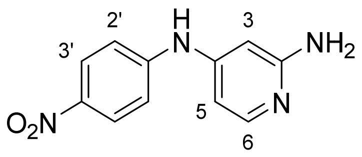

$C_{11}H_{10}N_4O_2$
230.23 g/mol

Following general procedure B, 4-nitroaniline (516 mg, 3.73 mmol, 1.20 eq), 4-chloropyridine-2-amine (400 mg, 3.11 mmol, 1.00 eq), $Pd(OAc)_2$ (14.0 mg, 60.0 µmol, 0.02 eq), XPhos (91.0 mg, 190 µmol, 0.06 eq) and K_2CO_3 (622 mg, 4.50 mmol, 1.45 eq) in 5 mL *t*BuOH and 3.5 µL water was stirred at 110 °C for four hours. The reaction mixture was quenched with 15 mL $NaHCO_3$ solution and extracted with EtOAc (3 x 100 mL). Purification by silica gel column chromatography [1% MeOH → 5% MeOH/1% TEA/ DCM] afforded **61** (257 mg, 1.12 mmol, 36%) as yellow solid.

TLC: R_f = 0.30 (5% MeOH/1% TEA/ DCM) [UV].

^{1}**H-NMR** (DMSO, 400 MHz) δ = 9.34 (s, 1H, NH), 8.15 (d, J = 9.2 Hz, 2H, H-3‘), 7.78 (d, J = 5.7 Hz, 1H, H-6), 7.23 (d, J = 9.3 Hz, 2H, H-2‘), 6.34 (dd, J = 5.8, 2.0 Hz, 1H, H-5), 6.28 (d, J = 2.0 Hz, 1H, H-3), 5.84 (s, 2H, NH_2).

^{13}**C-NMR** (DMSO, 101 MHz) δ = 160.76, 148.74, 148.40, 139.37, 125.74, 116.15, 103.11, 94.61.

***tert*-Butyl (*tert*-butoxycarbonyl)(2-fluoro-5-((4-nitrophenyl)amino)phenyl)carbamate (62)**

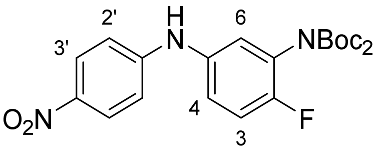

$C_{22}H_{26}FN_3O_6$
447.46 g/mol

99%

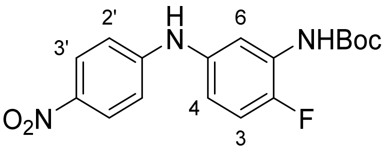

$C_{17}H_{18}FN_3O_4$
347.35 g/mol

1%

Following general procedure B, 4-nitroaniline (134 mg, 980 µmol, 1.20 eq), 4-chloropyridine-2-amine (200 mg, 810 µmol, 1.00 eq), $Pd(OAc)_2$ (5.00 mg, 20.0 µmol, 0.02 eq), XPhos (23.2 mg, 50.0 µmol, 0.06 eq) and K_2CO_3 (162 mg, 1.17 mmol, 1.45 eq) in 3 mL *t*BuOH and 2 µL water was stirred at 110 °C for four hours. The reaction mixture was quenched with 15 mL sat. $NaHCO_3$ solution and extracted with EtOAc (3 x 100 mL). Purification by silica gel column chromatography [1% TEA/ DCM] afforded **62** (175 mg, 410 µmol, 50%) as yellow solid.

^{1}**H-NMR** (DMSO, 400 MHz) δ = 9.30 (s, 1H, NH), 8.09 (d, J = 9.2 Hz, 2H, H-3‘), 7.33 (t, J = 9.2 Hz, 1H, H-6), 7.31 – 7.17 (m, 2H, H-3, H-4), 6.97 (d, J = 9.3 Hz, 2H, H-2‘), 1.40 (s, 18H, Boc_2).

tert-Butyl (tert-butoxycarbonyl)(3-fluoro-5-((4-nitrophenyl)amino)phenyl)carbamate & tert-butyl (3-fluoro-5-((4-nitrophenyl)amino)phenyl)carbamate (63)

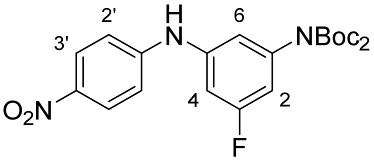

$C_{22}H_{26}FN_3O_6$
447.46 g/mol

88%

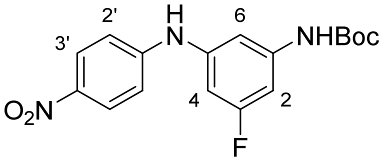

$C_{17}H_{18}FN_3O_4$
347.35 g/mol

12%

Following general procedure B, 4-nitroaniline (134 mg, 980 µmol, 1.20 eq), 4-chloropyridine-2-amine (200 mg, 810 µmol, 1.00 eq), $Pd(OAc)_2$ (5.00 mg, 20.0 µmol, 0.02 eq), XPhos (23.2 mg, 50 µmol, 0.06 eq) and K_2CO_3 (162 mg, 1.17 mmol, 1.45 eq) in 3 mL *t*BuOH and 2 µL water was stirred at 110 °C for 5.5 hours. The reaction mixture was quenched with 15 mL sat. $NaHCO_3$ solution and extracted with EtOAc (3 x 100 mL). Purification by silica gel column chromatography [1% TEA/ DCM] afforded **63** (161 mg, 380 µmol, 46%) as yellow solid.

^{1}H-NMR (DMSO, 400 MHz) δ = 9.49 (s, 1H, NH), 8.14 (d, J = 9.2 Hz, 2H, H-3’), 7.14 (d, J = 9.3 Hz, 2H, H-2’), 7.06 (dt, J = 10.6, 2.2 Hz, 1H, H-6*), 6.93 – 6.80 (m, 2H, H-2*, H-4*), 1.42 (s, 18H, Boc_2).

***N*^{4}-(4-Nitrophenyl)-6-(trifluoromethyl)pyrimidine-2,4-diamine (64)**

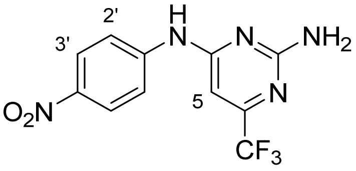

$C_{11}H_8F_3N_5O_2$
299.21 g/mol

Following general procedure B, 4-nitroaniline (334 mg, 2.42 mmol, 1.20 eq), 4-chloropyridine-2-amine (400 mg, 2.02 mmol, 1.00 eq), $Pd(OAc)_2$ (9.00 mg, 40.0 µmol, 0.02 eq), XPhos (57.0 mg, 120 µmol, 0.06 eq) and K_2CO_3 (404 mg, 2.93 mmol, 1.45 eq) in 5 mL *t*BuOH and 3.5 µL water was stirred at 110 °C for 5 hours. The reaction mixture was quenched with 15 mL sat. $NaHCO_3$ solution and extracted with EtOAc (3 x 100 mL). Purification by silica gel column chromatography [10% EtOAc →30% EtOAc/1% TEA/ pentane] afforded **64** (238 mg, 788 µmol, 39%) as yellow solid.

TLC: R_f = 0.37 (20% EtOAc/1% TEA/ pentane) [UV].

^{1}H-NMR (DMSO, 400 MHz) δ = 10.28 (s, 1H, NH), 8.18 (d, J = 9.3 Hz, 2H, H-3’), 8.05 (d, J = 9.3 Hz, 2H, H-2’), 7.13 (s, 2H, NH2), 6.46 (s, 1H, H-5).

^{13}C-NMR (DMSO, 101 MHz) δ = 163.32, 161.24, 154.41, 154.07, 146.32, 141.05, 124.88, 118.80, 93.54.

N[1]-(3-Nitrophenyl)benzene-1,4-diamine (65)

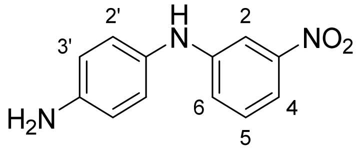

$C_{12}H_{11}N_3O_2$
229.24 g/mol

Following general procedure B, benzene-1,4-diamine (321 mg, 2.97 mmol, 1.20 eq), 1-bromo-3-nitrobenzene (400 mg, 1.98 mmol, 1.00 eq), $Pd(OAc)_2$ (9.00 mg, 40.0 µmol, 0.02 eq), XPhos (57.0 mg, 120 µmol, 0.06 eq) and K_2CO_3 (397 mg, 2.87 mmol, 1.45 eq) in 5 mL *t*BuOH and 3.5 µL water was stirred at 110 °C for 4 hours. The reaction mixture was quenched with 15 mL $NaHCO_3$ solution and extracted with EtOAc (3 x 100 mL). Purification by silica gel column chromatography [1% MeOH →5% MeOH/1% TEA/ DCM] afforded **65** (257 mg, 1.13 mmol, 57%) as red oil.

TLC: R_f = 0.50 (5% MeOH/1% TEA/ DCM) [UV].

^{1}H-NMR (DMSO, 400 MHz) δ = 8.10 (s, 1H, NH), 7.46 (s, 1H, H-2), 7.41 – 7.37 (m, 1H, H-6), 7.33 (t, J = 8.0 Hz, 1H, H-4), 7.15 – 7.07 (m, 1H H-5), 6.88 (d, J = 8.5 Hz, 2H, H-2‘), 6.59 (d, J = 8.6 Hz, 2H, H-3‘), 4.96 (s, 2H, NH2).

^{13}C-NMR (DMSO, 101 MHz) δ = 148.82, 148.59, 145.45, 130.16, 129.33, 124.22, 119.27, 114.76, 110.79, 106.07, 40.15, 39.94, 39.73, 39.52, 39.31, 39.10, 38.89, 24.03.

N[1]-(3-Methyl-5-nitrophenyl)benzene-1,4-diamine (66)

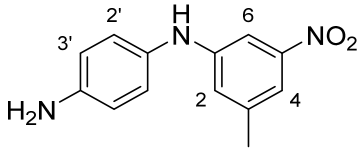

$C_{13}H_{13}N_3O_2$
243.27 g/mol

Following general procedure B, benzene-1,4-diamine (346 mg, 3.20 mmol, 1.20 eq), 1-chloro-3-methyl-5-nitrobenzene (400 mg, 2.13 mmol, 1.00 eq), $Pd(OAc)_2$ (10.0 mg, 43.0 µmol, 0.02 eq), XPhos (61.0 mg, 123 µmol, 0.06 eq) and K_2CO_3 (427 mg, 3.08 mmol, 1.45 eq) in 5 mL *t*BuOH and 3.5 µL water was stirred at 110 °C for 5 hours. The reaction mixture was quenched with 15 mL $NaHCO_3$ solution and extracted with EtOAc (3 x 100 mL). Purification by silica gel column chromatography [2% MeOH/1% TEA/ DCM] afforded **66** (321 mg, 1.32 mmol, 62%) as red oil.

TLC: R_f = 0.33 (5% MeOH/1% TEA/ DCM) [UV].

^{1}H-NMR (DMSO, 400 MHz) δ = 8.01 (s, 1H, NH), 7.28 (s, 1H, H-6), 7.24 (s, 1H, H-4), 6.91 (s, 1H, H-2), 6.86 (d, J = 8.6 Hz, 2H, H-2’), 6.59 (d, J = 8.5 Hz, 2H, H-3’), 4.95 (s, 2H, NH_2), 2.28 (s, 1H, CH_3).

^{13}C-NMR (DMSO, 101 MHz) δ = 148.80, 148.46, 145.42, 140.04, 129.39, 124.34, 119.48, 114.71, 111.46, 103.85, 20.98.

N-(4-Nitrophenyl)pyridin-2-amine (67)

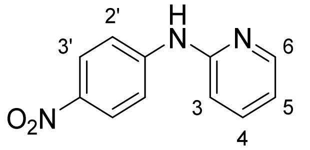

$C_{11}H_9N_3O_2$
215.21 g/mol

Following general procedure B, 4-nitroaniline (419 mg, 3.04 mmol, 1.20 eq), 4-bromopyridine (400 mg, 2.53 mmol, 1.00 eq), $Pd(OAc)_2$ (11.0 mg, 50.0 µmol, 0.02 eq), XPhos (72.0 mg, 150 µmol, 0.06 eq) and K_2CO_3 (507 mg, 3.67 mmol, 1.45 eq) in 7 mL *t*BuOH and 3.5 µL water was stirred at 110 °C for 5 hours. The reaction mixture was quenched with 15 mL sat. $NaHCO_3$ solution and extracted with EtOAc (3 x 100 mL). Purification by silica gel column chromatography [1% TEA/ DCM] afforded **67** (512 mg, 2.38 mmol, 94%) as yellow solid.

^{1}H-NMR (DMSO, 400 MHz) δ = 8.33 – 8.22 (m, 1H, H-6), 8.17 (d, J = 9.3 Hz, 1H, H-3'), 7.93 (d, J = 9.3 Hz, 2H, H-2'), 7.78 – 7.58 (m, 1H, H-4), 6.98 (d, J = 8.4 Hz, 1H, H-3), 6.97 – 6.92 (m, 1H, H-5).

^{13}C-NMR (DMSO, 101 MHz) δ = 154.46, 148.25, 147.32, 139.28, 137.99, 125.31, 116.62, 116.45, 112.49.

3-((4-Nitrophenyl)amino)phenol (68)

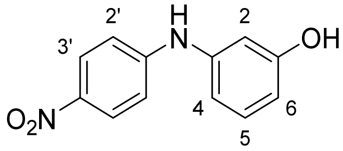

$C_{12}H_{10}N_2O_3$
230.22 g/mol

Following general procedure B, 4-nitroaniline (516 mg, 3.73 mmol, 1.20 eq), 3-chlorophenol (400 mg, 3.11 mmol, 1.00 eq), $Pd(OAc)_2$ (16.0 mg, 70.0 µmol, 0.02 eq), XPhos (100.0 mg, 210 µmol, 0.06 eq) and K_2CO_3 (705 mg, 4.50 mmol, 1.45 eq) in 4 mL *t*BuOH and 3 µL water was stirred at 110 °C for 16 hours. The reaction mixture was quenched with 15 mL $NaHCO_3$ solution and extracted with EtOAc (3 x 100 mL). Purification by silica gel column chromatography [3% MeOH/1% TEA/DCM] afforded **68** (704 mg, 3.06 mmol, 98%) as yellow solid.

^{1}H-NMR (DMSO, 400 MHz) δ = 9.54 (br. s, 1H, OH), 9.20 (s, 1H, NH), 8.08 (d, J = 9.3 Hz, 2H, H-3'), 7.15 (t, J = 8.0 Hz, 1H, H-5), 7.05 (d, J = 9.3 Hz, 2H, H-2'), 6.70 – 6.60 (m, 2H, H-2, H-4), 6.50 (ddd, J = 8.1, 2.3, 0.9 Hz, 1H, H-6).

^{13}C-NMR (DMSO, 101 MHz) δ = 158.36, 150.82, 141.16, 137.83, 130.24, 126.19, 113.57, 111.40, 110.71, 107.57.

tert-Butyl (4-((2-(ethylamino)pyrimidin-4-yl)amino)phenyl)carbamate (69)

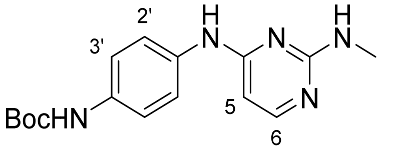

$C_{16}H_{21}N_5O_2$
315.38 g/mol

Following general procedure B, *tert*-butyl (4-aminophenyl)carbamate (872 mg, 4.19 mmol, 1.20 eq), 4-chloro-*N*-methylpyrimidin-2-amine (400 mg, 3.49 mmol, 1.00 eq), Pd(OAc)$_2$ (16.0 mg, 70.0 µmol, 0.02 eq), XPhos (100.0 mg, 210 µmol, 0.06 eq) and K_2CO_3 (699 mg, 5.00 mmol, 1.45 eq) in 3 mL *t*BuOH and 2 µL water was stirred at 110 °C for five hours. The reaction mixture was quenched with 15 mL $NaHCO_3$ solution and extracted with EtOAc (3 x 100 mL). Purification by silica gel column chromatography [2% → 5% MeOH/1% TEA/DCM] afforded **69** (523 mg, 1.82 mmol, 52%) as white solid.

^{1}H-NMR (DMSO, 400 MHz) δ = 9.16 (s, 1H, NH) 8.96 (s, 1H, NH), 7.79 (d, J = 5.7 Hz, 1H, H-6), 7.57 (d, J = 8.8 Hz, 2H, H-3'), 7.34 (d, J = 8.8 Hz, 1H, H-2'), 5.92 (d, J = 5.7 Hz, 1H, H-5), 2.77 (d, J = 4.6 Hz, 3H, CH_3), 1.47 (s, 9H, Boc).

The impure compound was used without further purification in the next step.

Ethyl 4-(naphthalen-1-ylamino)benzoate (70)

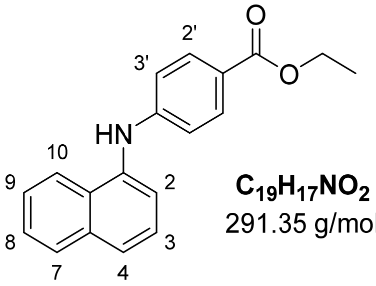

$C_{19}H_{17}NO_2$
291.35 g/mol

Following general procedure B, a mixture of ethyl-4-aminobenzoate (487 mg, 2.95 mmol, 1.20 eq), 1-chloronaphthalene (400 mg, 2.46 mmol, 1.00 eq), Pd(OAc)$_2$ (11 mg, 49.1 µmol, 0.02 eq), XPhos (70.3 mg, 148 µmol, 0.06 eq), K_2CO_3 (493 mg, 3.57 mmol, 1.45 eq) in 4 mL *t*BuOH and 3 µL water was stirred at 110 °C for 3 hours. The product was isolated by silica column chromatography [15% EtOAc/ 1% TEA /pentane] as light yellow solid (628 mg, 2.26 mmol, 87%).

TLC: R_f = 0.50 (15% EtOAc/ 1% TEA /pentane) [UV].

^{1}H-NMR (DMSO, 400 MHz) δ = 8.83 (s, 1H, NH), 8.06 – 8.02 (m, 1H, H-10), 7.97 – 7.93 (m, 1H, H-7), 7.77 (d, *J* = 8.8 Hz, 2H, H-2'), 7.73 (d, *J* = 8.0 Hz, 1H, H-4), 7.57 – 7.48 (m, 3H, H-3, H-8, H-9), 7.45 (d, *J* = 7.5, 1.3 Hz, 1H, H-2), 6.93 (d, *J* = 8.8 Hz, 2H, H-3'), 4.23 (q, *J* = 7.1 Hz, 2H, CH2), 1.28 (t, *J* = 7.1 Hz, 3H, CH3).

^{13}C-NMR (DMSO, 101 MHz) δ = 165.62, 150.63, 136.93, 134.41, 130.95, 128.40, 128.29, 126.31, 126.09, 125.75, 124.21, 122.91, 119.19, 118.86, 113.78, 59.82, 14.33.

Ethyl 4-(quinazolin-4-ylamino)benzoate (71)

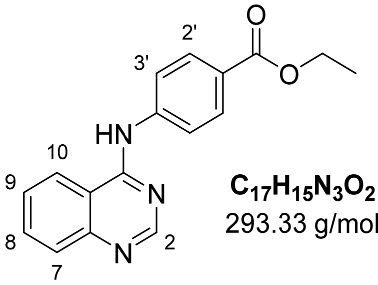

$C_{17}H_{15}N_3O_2$
293.33 g/mol

Following general procedure B, a mixture of ethyl-4-aminobenzoate (482 mg, 2.92 mmol, 1.20 eq), 4-chloroquinazoline (400 mg, 2.43 mmol, 1.00 eq), $Pd(OAc)_2$ (11.0 mg, 48.6 µmol, 0.02 eq), XPhos (70.0 mg, 146 µmol, 0.06 eq), K_2CO_3 (487 mg, 3.52 mmol, 1.45 eq) in 4 mL *t*BuOH and 3 µL water was stirred at 110 °C for 6 hours. The product was isolated by silica column chromatography [30% EtOAc/ 1% TEA /pentane] as light yellow solid (530 mg, 1.81 mmol, 35%).

TLC: R_f = 0.18 (30% EtOAc/ 1% TEA /pentane) [UV].

^{1}H-NMR (DMSO, 400 MHz) δ = 10.05 (s, 1H, NH), 8.71 (s, 1H, H-2), 8.61 (d, *J* = 8.3 Hz, 1H, H-7), 8.13 (d, *J* = 8.7 Hz, 2H, H-2'), 7.99 (d, *J* = 8.7 Hz, 2H, H-3'), 7.91 (t, *J* = 7.6 Hz, 1H, H-8), 7.84 (d, *J* = 8.5 Hz, 1H, H-10), 7.69 (t, *J* = 8.3 Hz, 1H, H-9), 4.31 (q, *J* = 7.1 Hz, 2H, CH_2), 1.33 (t, *J* = 7.1 Hz, 3H, CH_3).

Ethyl 4-((3a,7a-dihydro-1H-pyrrolo[2,3-b]pyridin-4-yl)amino)benzoate (72)

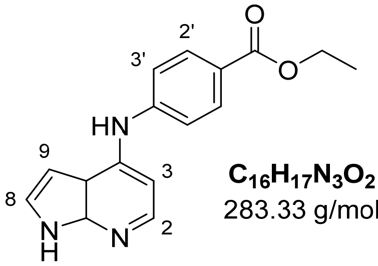

$C_{16}H_{17}N_3O_2$
283.33 g/mol

Following general procedure B, a mixture of ethyl-4-aminobenzoate (515 mg, 3.12 mmol, 1.20 eq), 4-chloro-7H-pyrrolo[2,3-d]pyrimidine (400 mg, 2.46 mmol, 1.00 eq), $Pd(OAc)_2$ (12.0 mg, 52.4 µmol, 0.02 eq), XPhos (75.0 mg, 157 µmol, 0.06 eq), K_2CO_3 (525 mg, 3.80 mmol, 1.45 eq) in 4 mL *t*BuOH and 3 µL water was stirred at 110 °C for 6 hours. The product **72** was isolated by silica column chromatography [30% EtOAc/ 1% TEA /pentane] as light yellow solid (259 mg, 921 µmol, 35%).

^{1}H-NMR (DMSO, 400 MHz) δ = 11.48 (s, 1H, NH), 9.08 (s, 1H, NH), 8.01 (d, *J* = 5.4 Hz, 1H, H-2), 7.90 (d, *J* = 8.8 Hz, 2H, H-2'), 7.34 (d, *J* = 8.8 Hz, 2H, H-3'), 7.28 (dd, *J* = 3.4, 2.1 Hz, 1H, H-8), 6.89 (d, *J* = 5.4 Hz, 1H, H-3), 6.57 (dd, *J* = 3.5, 1.6 Hz, 1H, H-9), 4.28 (q, *J* = 7.1 Hz, 2H, CH2), 1.31 (t, *J* = 7.1 Hz, 3H, CH3).

^{13}C-NMR (DMSO, 101 MHz) δ = 165.46, 150.00, 146.50, 143.74, 141.37, 130.67, 123.09, 121.64, 117.46, 110.11, 101.27, 98.33, 60.17, 14.29.

General procedure C: reduction of nitroaryls

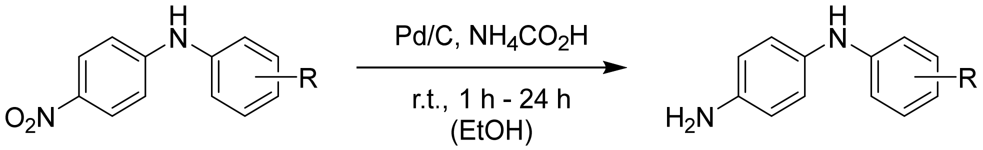

To a stirred solution of nitro compound (1.00 eq) in EtOH was added 10 wt% palladium on carbon (1.10 eq) and ammonium formate (10.0 eq). The reaction mixture was stirred at room temperature for 1 – 24 hours. Upon completion of the reaction, the suspension was filtered over Celite and washed with EtOAc or DCM. The filtrate was concentrated and dissolved in EtOAc and sat. $NaHCO_3$ solution. The aqueous phase was washed with EtOAc (3 x 100 mL) and the combined organic layer was dried over $MgSO_4$, filtered and concentrated.

N^4-(4-Aminophenyl)-6-methylpyrimidine-2,4-diamine (73)

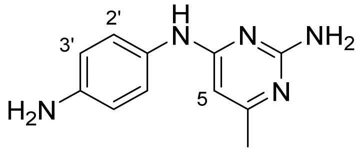

$C_{11}H_{13}N_5$
215.26 g/mol

Following general procedure C, **51** (591 mg, 2.40 mmol, 1.00 eq), ammonium formate (1.73 g, 27.4 mmol, 11.4 eq) and 10 wt% Pd/C (450 mg, 4.23 mmol, 1.76 eq) in 25 mL ethanol was stirred at room temperature for 4.5 hours. The residue was filtrated, washed with DCM and concentrated. Extraction with EtOAc afforded pure compound **73** as brown-orange solid (536 mg, 2.11 mmol, 88%).

TLC: R_f = 0.48 (5% MeOH/1% TEA/DCM) [UV].

^{1}H-NMR (DMSO-d_6, 400 MHz) δ = 8.36 (s, 1H, NH), 7.14 (d, J = 8.61 Hz, 2H, H-2’), 6.51 (d, J = 8.61 Hz, 2H, H-3’), 5.87 (s, 2H, NH_2), 5.67 (s, 1H, H-5), 4.81 (s, 2H, NH_2), 2.01 (s, 3H, CH_3). These data are in accordance with previous literature reports.[3]

N^4-(4-Aminophenyl)pyrimidine-2,4-diamine (74)

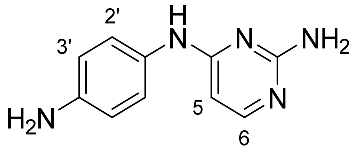

$C_{10}H_{11}N_5$
201.23 g/mol

Following general procedure C, **52** (226 mg, 977 µmol, 1.00 eq), ammonium formate (702 mg, 11.1 mmol, 11.4 eq) and 10 wt% Pd/C (182 mg, 1.71 mmol, 1.76 eq) were stirred in 10 mL ethanol at room temperature for 5 hours. The residue was filtrated, washed with EtOAc and concentrated. Extraction with EtOAc afforded pure compound **74** as brown-orange solid (154 mg, 762 µmol, 78%).

TLC: R_f = 0.20 (5% MeOH/1%TEA/DCM) [UV].

^{1}H-NMR (DMSO, 400 MHz) δ = 8.51 (s, 1H, NH), 7.67 (d, J = 5.80 Hz, 1H, H-6), 7.17 (d, J = 8.60 Hz, 2H, H-2‘), 6.51 (d, J = 8.60 Hz, 2H, H-3‘), 5.93 (s, 2H, NH_2), 5.80 (d, J = 5.80 Hz, 1H, H-5), 4.81 (bs, 2H, NH_2).
^{13}C-NMR (DMSO, 101 MHz) δ = 163.13, 161.54, 155.80, 144.48, 129.08, 122.80, 114.14, 95.39.
HR-MS m/z calcd. for $C_{10}H_{12}N_6^+$ (M + 1)$^+$ 202.1093, found 202.1095.

N[1]-(5-(Trifluoromethyl)pyridin-2-yl)benzene-1,4-diamine (75)

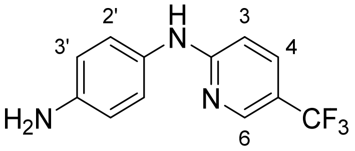

$C_{12}H_{10}F_3N_3$
253.23 g/mol

Following general procedure C, **53** (63.0 mg, 222 µmol, 1.00 eq), ammonium formate (160 mg, 2.53 mmol, 11.4 eq) and 10 wt% Pd/C (41 mg, 387 µmol, 1.76 eq) in 5 mL ethanol were stirred at room temperature overnight. The residue was filtrated, washed with EtOAc and concentrated. Extraction with EtOAc afforded pure compound **75** as brown solid (55.0 mg, 218 µmol, 98%).
^{1}H-NMR (MeOD, 400 MHz) δ = 8.25 (s, 1H, H-6), 7.64 (dd, J = 8.92 Hz, 1H, H-4), 7.19 (d, J = 8.65 Hz, 2H, H-2‘), 6.75 (d, J = 8.65 Hz, 2H, H-3‘), 6.70 (d, J = 8.92 Hz, 1H, H-3).
^{13}C-NMR (MeOD, 101 MHz) δ = 161.03, 146.36, 145.38, 135.36, 131.75, 124.92, 117.24, 109.46.
HR-MS m/z calcd. for $C_{12}H_{11}H_3N_3^+$ (M + 1)$^+$ 254.0900, found 254.0902.

N[1]-(4-(Trifluoromethyl)phenyl)benzene-1,4-diamine (76)

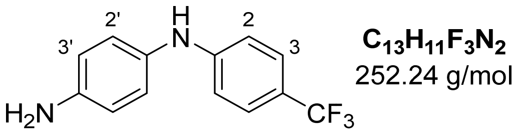

$C_{13}H_{11}F_3N_2$
252.24 g/mol

Following general procedure C, **54** (317 mg, 1.12 mmol, 1.00 eq), ammonium formate (777 mg, 1.90 mmol, 11.0 eq) and 10 wt% Pd/C (202 mg, 1.90 mmol, 1.76 eq) were stirred in 10 mL ethanol at room temperature overnight. The reaction mixture was filtrated, washed with EtOAc and concentrated. Compound **76** was obtained as brown solid (269 mg, 1.06 mmol, 95%).
^{1}H-NMR (DMSO, 400 MHz) δ = 8.08 (s, 1H, NH), 7.38 (d, J = 8.6 Hz, 2H, H-3), 6.86 (d, J = 8.6 Hz, 2H, C-2‘), 6.80 (d, J = 8.5 Hz, 2H, C-2), 6.57 (d, J = 8.6 Hz, 2H, C-3‘), 4.93 (s, 2H, NH_2).
^{13}C-NMR (DMSO, 101 MHz) δ = 150.87, 145.78, 129.82, 126.76, 124.67, 115.10, 112.81.
HR-MS m/z calcd. for $C_{13}H_{12}F_3N_2^+$ (M + 1)$^+$ 253.0947, found 253.0984.

N^1-(2-(Trifluoromethyl)phenyl)benzene-1,4-diamine (77)

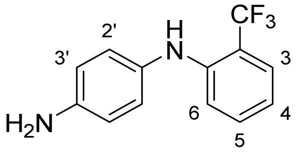

$C_{13}H_{11}F_3N_2$
252.24 g/mol

Following general procedure C, **55** (360 mg, 1.27 mmol, 1.00 eq), ammonium formate (880 mg, 14.0 mmol, 11.0 eq) and 10 wt% Pd/C (162 mg, 1.53 mmol, 1.20 eq) in 10 mL ethanol were stirred at room temperature overnight. The reaction mixture was filtrated, washed with EtOAc and concentrated. Compound **77** (306 mg, 1.22 mmol, 96%) was obtained as brown liquid.

^{1}H-NMR (DMSO, 400 MHz) δ = 7.45 (d, J = 6.1 Hz, 1H, H-3), 7.29 (t, J = 7.8 Hz, 1H, H-4), 6.92 (s, 1H, NH), 6.85 (d, J = 8.5 Hz, 2H, H-2'), 6.80 – 6.67 (m, 2H,H-5, H-6), 6.57 (d, J = 8.5 Hz, 2H, H-3'), 4.98 (s, 2H, NH_2).

^{13}C-NMR (DMSO, 101 MHz) δ = 145.88, 145.44, 133.06, 129.41, 126.41, 126.35, 126.15, 116.60, 115.03, 114.56.

HR-MS m/z calcd. for $C_{13}H_{12}F_3N_2^+$ $(M + 1)^+$ 253.0947, found 253.0949.

N^1-(*p*-Tolyl)benzene-1,4-diamine (78)

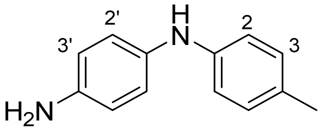

$C_{13}H_{14}N_2$
198.27 g/mol

Following general procedure C, **56** (365 mg, 1.60 mmol, 1.00 eq), 10 wt% Pd/C (187 mg, 1.76 mmol, 1.10 eq) and ammonium formate (1.00 g, 16.0 mmol, 10.0 eq) in 20 mL EtOH were stirred at room temperature overnight. The reaction mixture was filtrated, washed with EtOAc and concentrated to obtain **78** (216 mg, 1.09 mmol, 68%) as light brown solid.

^{1}H-NMR (DMSO, 400 MHz) δ = 8.25 (s, 1H, NH), 7.28 (s, 1H, NH_2), 6.90 (d, J = 8.4 Hz, 2H, H-2'), 6.78 (d, J = 8.6 Hz, 2H), 6.70 (d, J = 8.4 Hz, 2H), 6.51 (d, J = 8.6 Hz, 1H), 2.16 (s, 3H, CH_3).

^{13}C-NMR (DMSO, 101 MHz) δ = 167.91, 163.99, 144.09, 143.33, 132.36, 129.36, 125.62, 121.69, 114.75, 114.14, 20.14.

HR-MS m/z calcd. for $C_{13}H_{15}N_2^+$ $(M + 1)^+$ 199.1230, found 199.1234.

N^1-Phenylbenzene-1,4-diamine (79)

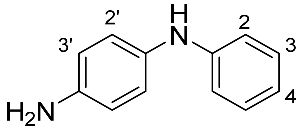

$C_{12}H_{12}N_2$
184.24 g/mol

Following general procedure C, **57** (344 mg, 1.61 mmol, 1.00 eq), 10 wt% Pd/C (188 mg, 1.77 mmol, 1.10 eq), and ammonium formate (1.02 g, 16.1 mmol, 10.0 eq) in 13 mL EtOH

were stirred at room temperature overnight. The reaction mixture was filtrated, washed with DCM and concentrated to obtain **79** (279 mg, 1.50 mmol, 93%) as brown oil.

^{1}H-NMR (DMSO, 400 MHz) δ = 7.44 (s, 1H, NH), 7.21 – 6.96 (m, 2H, H-3), 6.82 (d, J = 8.6 Hz, 2H, H-2'), 6.76 (d, J = 7.5 Hz, 2H, H-2), 6.59 (t, J = 7.2 Hz, 1H, H-4), 6.53 (d, J = 8.5 Hz, 2H, H-3'), 4.75 (s, 2H, NH_2).

^{13}C-NMR (DMSO, 101 MHz) δ = 146.72, 143.87, 131.55, 128.94, 122.55, 116.96, 114.71, 113.60.

HR-MS m/z calcd. for $C_{12}H_{13}N_2^+$ (M + 1)$^+$ 185.1073, found 185.1075.

*N*¹-(4-Methoxyphenyl)benzene-1,4-diamine (80)

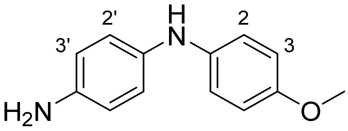

$C_{13}H_{14}N_2O$
214.27 g/mol

Following general procedure C, **58** (296 mg, 1.21 mmol, 1.00 eq), 10 wt% Pd/C (142 mg, 1.33 mmol, 1.10 eq), and ammonium formate (762 mg, 12.1 mmol, 10.0 eq) in 15 mL EtOH were stirred at room temperature overnight. The reaction mixture was filtrated, washed with EtOAc and concentrated to obtain **80** (249 mg, 1.21 mmol, 96%) as brown solid.

^{1}H-NMR (DMSO, 400 MHz) δ = 8.18 (s, 1H, NH), 7.13 (s, 2H, NH_2), 6.82 – 6.64 (m, 6H, H-2', H-2, H-3), 6.50 (d, J = 8.6 Hz, 2H, H-3'), 3.65 (s, 3H, CH_3).

^{13}C-NMR (DMSO, 101 MHz) δ = 163.42, 151.84, 142.75, 139.94, 133.49, 120.56, 116.10, 114.87, 114.50, 55.25.

HR-MS m/z calcd. for $C_{13}H_{15}N_2O^+$ (M + 1)$^+$ 215.1179, found 215.1184.

*N*¹-(4-Fluorophenyl)benzene-1,4-diamine (81)

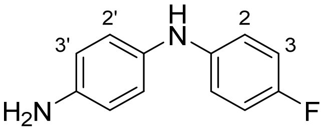

$C_{12}H_{11}FN_2$
202.23 g/mol

Following general procedure C, **59** (285 mg, 1.23 mmol, 1.00 eq), 10 wt% Pd/C (144 mg, 1.35 mmol, 1.10 eq), and ammonium formate (775 mg, 12.3 mmol, 10.0 eq) in 10 mL EtOH were stirred at room temperature overnight. The reaction mixture was filtrated, washed with EtOAc and concentrated to obtain **81** (193 mg, 1.23 mmol, 77%) as brown solid.

^{1}H-NMR (DMSO, 400 MHz) δ = 7.40 (s, 1H, NH), 6.93 (t, J = 8.9 Hz, 2H, H-2), 6.79 (d, J = 8.6 Hz, 2H, H-2'), 6.77 – 6.70 (m, 2H, H-3), 6.53 (d, J = 8.6 Hz, 2H, H-3').

^{13}C-NMR (DMSO, 101 MHz) δ = 163.51, 156.00, 153.69, 143.79, 143.29, 132.01, 122.14, 115.45, 115.23, 114.83, 114.75.

HR-MS m/z calcd. for $C_{12}H_{12}FN_2^+$ (M + 1)$^+$ 203.0979, found 203.0982.

N^2-(4-Aminophenyl)pyridine-2,6-diamine (82)

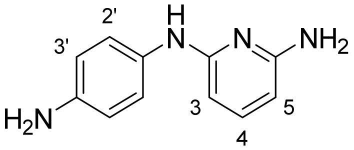

$C_{11}H_{12}N_4$
200.25 g/mol

Following general procedure C, **60** (195 mg, 850 µmol, 1.00 eq), 10 wt% Pd/C (99.0 mg, 930 µmol, 1.10 eq), and ammonium formate (536 mg, 8.50 mmol, 10.0 eq) in 20 mL EtOH were stirred at room temperature overnight. The reaction mixture was filtrated, washed with EtOAc and concentrated. Extraction with EtOAc and sat. $NaHCO_3$ solution afforded **82** (156 mg, 773 µmol, 91%) as dark red solid.

TLC: R_f = 0.52 (5% MeOH/1% TEA/ DCM) [UV].

^{1}H-NMR (DMSO, 400 MHz) δ = 7.80 (s, 1H, NH), 7.14 (d, J = 8.6 Hz, 2H, H-2'), 7.06 (t, J = 7.8 Hz, 1H, H-4), 6.49 (d, J = 8.6 Hz, 1H, H-3'), 5.78 (d, J = 7.9, 1H, H-3), 5.70 (d, J = 7.7 Hz, 1H, H-5), 5.44 (s, 2H, NH_2), 4.68 (s, 2H, NH_2).

^{13}C-NMR (DMSO, 101 MHz) δ = 158.98, 156.82, 143.51, 138.50, 131.82, 122.07, 114.73, 96.40, 95.88.

N^4-(4-Aminophenyl)pyridine-2,4-diamine (83)

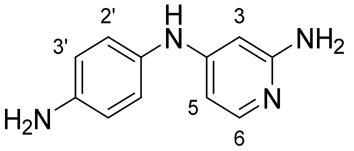

$C_{11}H_{12}N_4$
200.25 g/mol

Following general procedure C, **61** (257 mg, 1.11 mmol, 1.00 eq), 10 wt% Pd/C (131 mg, 1.23 mmol, 1.10 eq), and ammonium formate (700 mg, 11.1 mmol, 10.0 eq) in 20 mL EtOH were stirred at room temperature overnight. The reaction mixture was filtrated, washed with EtOAc and concentrated to obtain **83** (206 mg, 1.02 mmol, 92%) as brown solid.

^{1}H-NMR (DMSO, 400 MHz) δ = 7.78 (s, 1H, NH), 7.48 (d, J = 5.8 Hz, 1H, H-6), 6.81 (d, J = 8.5 Hz, 2H, H-2'), 6.55 (d, J = 8.5 Hz, 2H, H-3'), 5.93 (dd, J = 5.9, 2.1 Hz, 1H, H-5), 5.74 (d, J = 2.0 Hz, 1H, H-3), 5.38 (s, 2H, NH_2), 4.89 (s, 2H, NH_2).

^{13}C-NMR (DMSO, 101 MHz) δ = 160.36, 153.53, 147.37, 145.14, 129.27, 124.45, 114.49, 100.59, 89.02.

tert-Butyl (5-((4-aminophenyl)amino)-2-fluorophenyl)(tert-butoxycarbonyl)carbamate & *tert*-butyl (5-((4-aminophenyl)amino)-2-fluorophenyl)carbamate (84)

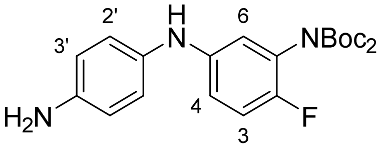

$C_{22}H_{28}FN_3O_4$
417.48 g/mol

94%

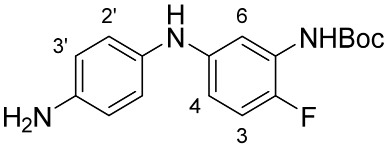

$C_{17}H_{20}FN_3O_2$
317.36 g/mol

6%

Following general procedure C, **62** (175 mg, 410 µmol, 1.00 eq), 10 wt% Pd/C (52.3 mg, 49.0 µmol, 1.10 eq), and ammonium formate (259 mg, 4.11 mmol, 10.0 eq) in 20 mL EtOH were stirred at room temperature overnight. The reaction mixture was filtrated, washed with EtOAc and concentrated to obtain **84**(158 mg, 377 µmol, 92%) as brown solid.

^{1}H-NMR (DMSO, 400 MHz) δ = 7.49 (s, 1H, NH), 7.02 (t, J = 9.4 Hz, 1H, H-6), 6.78 (d, J = 8.6 Hz, 2H, H-2'), 6.74 – 6.68 (m, 1H, H-3), 6.60 – 6.51 (m, 3H, H-4, H-3'), 4.80 (s, 2H, NH_2), 1.38 (s, 18H, Boc_2).

N[4]-(4-Aminophenyl)-6-(trifluoromethyl)pyrimidine-2,4-diamine (85)

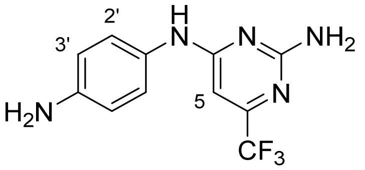

$C_{11}H_{10}F_3N_5$
269.23 g/mol

Following general procedure C, **64** (235 mg, 790 µmol, 1.00 eq), 10 wt% Pd/C (92.0 mg, 860 µmol, 1.10 eq), and ammonium formate (497 mg, 7.90 mmol, 10.0 eq) in 20 mL EtOH were stirred at room temperature overnight. The reaction mixture was filtrated, washed with EtOAc and concentrated to obtain **85** (175 mg, 648 µmol, 82%) as grey solid.

^{1}H-NMR (DMSO, 400 MHz) δ = 9.08 (s, 1H, NH), 7.23 (br. s, 2H, H-2'), 6.60 (s, 2H, NH_2), 6.53 (d, J = 8.7 Hz, 2H, H-3'), 6.15 (br.s, 1H, H-5), 4.91 (br.s, 2H, NH_2).

N-(4-((3-Aminophenyl)amino)phenyl)-4-(quinolin-4-ylamino)benzamide (39)

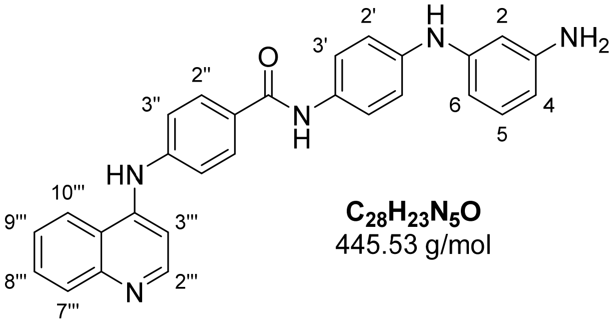

$C_{28}H_{23}N_5O$
445.53 g/mol

Following general procedure C, **86** (320 mg, 670 µmol, 1.00 eq), 10 wt% Pd/C (78.0 mg, 737 µmol, 1.10 eq), and ammonium formate (422 mg, 6.70 mmol, 10.0 eq) in 20 mL EtOH were stirred at room temperature overnight. The reaction mixture was filtrated, washed with EtOAc and concentrated. The crude product was purified by silica gel column chromatography [2% MeOH → 5% MeOH/1% TEA/DCM] and precipitated with isopropanol (2 mL) and water (15 mL) to obtain **39** (71.0 mg, 154 µmol, 23%) as yellowish solid.

TLC: R_f = 0.28 (5% MeOH/1% TEA/ DCM) [UV].

^{1}H-NMR (DMSO, 400 MHz) δ = 9.97 (s, 1H, NH), 9.23 (s, 1H, NH), 8.57 (d, J = 5.2 Hz, 1H, H-2‘‘), 8.39 (d, J = 8.4 Hz, 1H, H-10‘‘‘), 8.00 (d, J = 8.6 Hz, 2H, H-3‘‘), 7.93 (d, J = 8.4 Hz, 1H, H-7‘‘‘), 7.75 (s, 1H, NH), 7.72 (t, J = 8.0 Hz, 1H, H-8‘‘‘), 7.61 (d, J = 8.8 Hz, 2H, H-3‘), 7.57 (t, J =

8.4 Hz, 1H, H-9‘“), 7.47 (d, J = 8.4 Hz, 2H, H-2“), 7.19 (d, J = 5.2 Hz, 1H, H-3‘“), 7.03 (d, J = 8.9 Hz, 2H, H-2‘), 6.85 (t, J = 7.9 Hz, 1H, H-5), 6.33 (s, 1H, H-2), 6.22 (d, J = 7.8 Hz, 1H, H-6), 6.04 (d, J = 7.9 Hz, 1H, H-4), 4.95 (s, 2H, NH_2).
^{13}C-NMR (DMSO, 101 MHz) δ = 164.29, 149.43, 144.53, 139.74, 131.45, 129.46, 129.35, 128.98, 128.87, 124.98, 122.29, 121.64, 120.36, 119.76, 117.37, 106.00, 104.84, 101.74.
HR-MS m/z calcd. for $C_{28}H_{24}N_5O^+$ $(M + 1)^+$ 446.1975, found 446.2001.

***N*-(4-((3-Amino-5-methylphenyl)amino)phenyl)-4-(quinolin-4-ylamino)benzamide (43)**

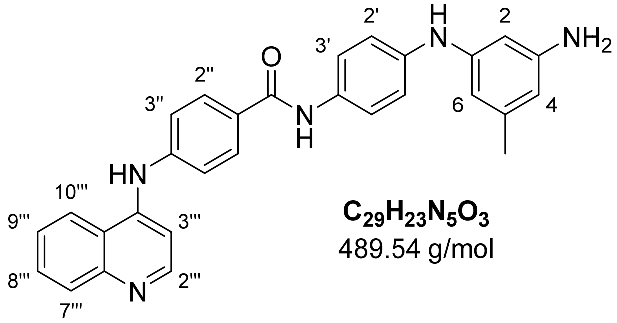

$C_{29}H_{23}N_5O_3$
489.54 g/mol

Following general procedure C, **87** (149 mg, 300 µmol, 1.00 eq), 10 wt% Pd/C (36.0 mg, 330 µmol, 1.10 eq), and ammonium formate (189 mg, 3.00 mmol, 10.0 eq) in 20 mL EtOH were stirred at room temperature overnight. The reaction mixture was filtrated, washed with EtOAc and concentrated. The crude product was purified by silica gel column chromatography [2% MeOH → 3% MeOH/1% TEA/DCM] to obtain **43** (67.0 mg, 144 µmol, 48%) as yellowish solid.
TLC: R_f = 0.33 (5% MeOH/1% TEA/ DCM) [UV].
^{1}H-NMR (DMSO, 400 MHz) δ = 9.97 (s, 1H, NH), 9.22 (s, 1H, NH), 8.58 (d, J = 5.3 Hz, 1H, H-2‘“), 8.39 (d, J = 8.4 Hz, 1H, H-10‘“), 8.00 (d, J = 8.6 Hz, 2H, H-3“), 7.93 (d, J = 8.4 Hz, 1H, H-7‘“), 7.73 (t, J = 7.6 Hz, 1H, H-8‘“), 7.68 (s, 1H, NH), 7.59 (d, J = 8.9 Hz, 2H, H-3‘), 7.56 (t, J = 8.4 Hz, 1H, H-9‘“) 7.47 (d, J = 8.3 Hz, 2H, H-2“), 7.19 (d, J = 5.2 Hz, 1H, H-3‘“), 7.02 (d, J = 8.9 Hz, 2H, H-2‘), 6.15 (s, 1H, H-2), 6.05 (s, 1H, H-6*), 5.89 (s, 1H, H-4*), 4.84 (s, 2H, NH_2), 2.09 (s, 3H, CH_3).
^{13}C-NMR (DMSO, 101 MHz) δ = 164.30, 150.70, 149.24, 149.01, 146.39, 144.46, 139.84, 138.26, 131.38, 129.44, 129.25, 128.98, 128.85, 124.97, 122.28, 121.64, 120.38, 119.74, 117.47, 106.94, 105.82, 103.56, 99.27, 21.43.
HR-MS m/z calcd. for $C_{29}H_{24}N_5O_3^+$ $(M + 1)^+$ 460.2131, found 460.2157.

***N*^{1}-(Pyridin-2-yl)benzene-1,4-diamine (88)**

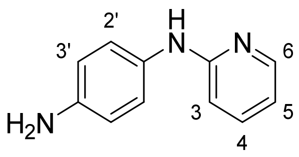

$C_{11}H_{11}N_3$
185.23 g/mol

Following general procedure C, **67** (506 mg, 2.35 mmol, 1.00 eq), 10 wt% Pd/C (300 mg, 2.82 mmol, 1.20 eq), and ammonium formate (1.48 g, 23.5 mmol, 10.0 eq) in 20 mL EtOH were stirred at room temperature for three hours. The reaction mixture was filtrated, washed with EtOAc and concentrated to obtain **88** (445 mg, 2.35 mmol, 100%) as solid.

^{1}H-NMR (DMSO, 400 MHz) δ = 8.37 (s, 1H, NH), 8.08 – 7.98 (m, 1H, H-6), 7.46 – 7.34 (m, 1H, H-4), 7.20 (d, J = 8.7 Hz, 2H, H-2'), 6.62 (d, J = 8.4 Hz, 1H, H-3), 6.59 – 6.55 (m, 1H, H-5), 6.53 (d, J = 8.7 Hz, 2H, H-3').

^{13}C-NMR (DMSO, 101 MHz) δ = 157.09, 147.42, 143.51, 136.83, 130.60, 121.58, 114.24, 112.67, 108.70, 40.15, 39.99, 39.94, 39.78, 39.73, 39.52, 39.31, 39.10, 38.89.

3-((4-Aminophenyl)amino)phenol (89)

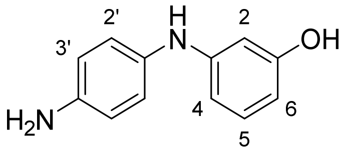

$C_{12}H_{12}N_2O$
200.24 g/mol

Following general procedure C, **68** (704 mg, 3.06 mmol, 1.00 eq), 10 wt% Pd/C (390 mg, 3.67 mmol, 1.20 eq), and ammonium formate (1.92 g, 30.6 mmol, 10.0 eq) in 20 mL EtOH were stirred at room temperature overnight. The reaction mixture was filtrated, washed with EtOAc and concentrated. The crude product was further purified by precipitation with isopropanol/ water to obtain **89** (250 mg, 1.25 mmol, 40%) as brown solid.

^{1}H-NMR (DMSO, 400 MHz) δ = 8.93 (s, 1H, OH), 7.32 (s, 1H, NH), 6.85 (t, J = 8.2 Hz, 1H, H-5), 6.80 (d, J = 8.6 Hz, 2H, H-2'), 6.52 (d, J = 8.5 Hz, 2H, H-3'), 6.24 – 6.13 (m, 2H, H-2, H-4), 6.05 – 5.91 (m, 1H, H-6), 4.74 (s, 2H, NH_2).

^{13}C-NMR (DMSO, 101 MHz) δ = 158.12, 148.05, 143.83, 131.65, 129.52, 122.83, 114.63, 105.18, 104.46, 100.41.

General procedure D: ester hydrolysis

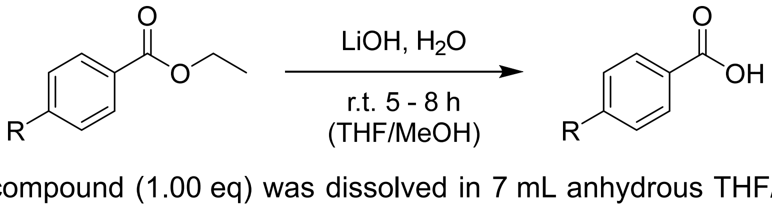

Ethyl benzoate compound (1.00 eq) was dissolved in 7 mL anhydrous THF/MeOH (1:1 v/v). Lithium hydroxide (10.0 eq) and 8.5 mL water were added, and the reaction mixture was stirred at room temperature for 5 – 8 hours. The mixture was acidified with 1 M hydrochloric acid below pH 4. The obtained precipitate was filtered, washed with cold water and dried at 50 °C under reduced pressure.

4-(Quinolin-4-ylamino)benzoic acid (90)

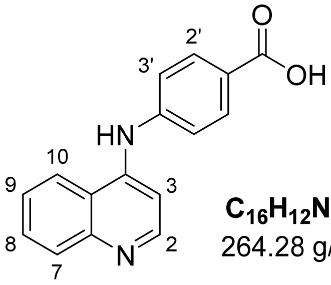

$C_{16}H_{12}N_2O_2$
264.28 g/mol

Following general procedure D, a mixture of ethyl 4-(quinolin-4-ylamino)benzoate (**50**) (669 mg, 2.29 mmol, 1.00 eq), LiOH (548 mg, 22.9 mmol, 10.0 eq) in 20 mL THF/MeOH (1:1 v/v) and 23 mL water were stirred at room temperature for five hours. Precipitation with 1 M hydrochloric acid solution afforded product **90** as a yellowish solid (571 mg, 2.15 mmol, 94%).
TLC: R_f = 0.05 (1% MeOH/1%TEA/DCM) [UV].
^{1}H-NMR (DMSO-d$_6$, 400 MHz) δ = 11.02 (br.s, 1H, OH), 8.81 (d, J = 8.49 Hz, 1H, H-2), 8.59 (d, J = 6.9 Hz, 1H, H-10), 8.11 (d, J = 8.52 Hz, 2H, H-2'), 8.10 (d, J = 8.41 Hz, 1H, H-7), 8.04 (t, J = 8.41 Hz, 1H, H-8), 7.83 (t, J = 7.70 Hz, 1H, H-9), 7.63 (d, J = 8.52 Hz, 2H, H-3'), 7.06 (d, J = 6.9 Hz, 1H, H-3).
These data are in accordance with previous literature reports.[3]

4-(Naphthalen-1-ylamino)benzoic acid (91)

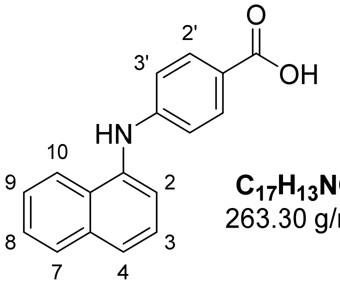

$C_{17}H_{13}NO_2$
263.30 g/mol

Following general procedure D, a mixture of ethyl 4-(quinazolin-4-ylamino)benzoate (**70**) (596 mg, 2.04 mmol, 1.00 eq), LiOH (487 mg, 20.4 mmol, 10.0 eq) in 16 mL THF/MeOH (1:1 v/v) and 23 mL water were stirred at room temperature for five hours. Precipitation with 1 M hydrochloric acid solution afforded product **91** as a white solid (430 mg, 1.63 mmol, 80%).
^{1}H-NMR (DMSO-d$_6$, 400 MHz) δ = 9.18 (s, 1H, NH), 8.48 (d, *J* = 7.5 Hz, 1H, H-10), 8.36 (d, *J* = 7.3 Hz, 1H, H-7), 8.17 (d, *J* = 8.7 Hz, 2H, H-2'), 8.13 (d, *J* = 7.9 Hz, 1H, H-4), 7.99 – 7.89 (m, 3H, H-3, H-8, H-9), 7.87 (d, *J* = 7.5, 1H, H-2), 7.34 (d, *J* = 8.7 Hz, 2H, H-3').
These data are in accordance with previous literature reports.[6]

4-(Quinazolin-4-ylamino)benzoic acid (92)

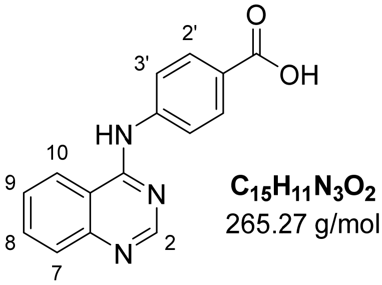

$C_{15}H_{11}N_3O_2$
265.27 g/mol

Following general procedure D, a mixture of ethyl 4-(quinazolin-4-ylamino)benzoate (**71**) (530 mg, 1.81 mmol, 1.00 eq), LiOH (433 mg, 18.1 mmol, 10.0 eq) in 12 mL THF/MeOH (1:1 v/v) and 10 mL water were stirred at room temperature for five hours. Precipitation with 1 M hydrochloric acid solution afforded product **92** as a white solid (460 mg, 1.73 mmol, 96%).

^{1}H-NMR (DMSO-d$_6$, 400 MHz) δ = 11.94 (s, 1H, OH), 9.08 (d, *J* = 8.4 Hz, 1H, H-2), 9.00 (s, 1H, NH), 8.12 (t, *J* = 7.1 Hz, 1H, H-10), 8.07 – 8.01 (m, 3H, H-10, H-2'), 7.96 (d, *J* = 7.7 Hz, 2H, H-3'), 7.87 (t, *J* = 7.7 Hz, 1H, H-9).

^{13}C-NMR (DMSO, 101 MHz) δ = 166.68, 159.82, 151.13, 140.88, 136.26, 129.89, 128.65, 128.22, 124.93, 124.11, 120.22, 113.77.

4-((3a,7a-Dihydro-1H-pyrrolo[2,3-b]pyridin-4-yl)amino)benzoic acid (93)

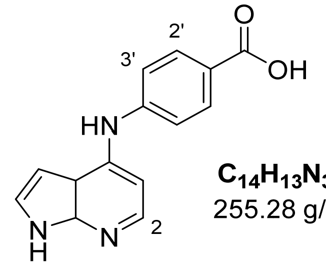

$C_{14}H_{13}N_3O_2$
255.28 g/mol

Following general procedure D, a mixture of ethyl ethyl 4-((3a,7a-dihydro-1H-pyrrolo[2,3-b]pyridin-4-yl)amino)benzoate (**72**) (259 mg, 921 µmol, 1.00 eq), LiOH (216 mg, 9.03 mmol, 10.0 eq) in 8 mL THF/MeOH (1:1 v/v) and 8 mL water were stirred at room temperature for five hours. Precipitation with 1 M hydrochloric acid solution afforded product **93** as a white solid (201 mg, 79.0 µmol, 88%).

^{1}H-NMR (DMSO, 400 MHz) δ = 12.61 (s, 1H, NH), 10.66 (s, 1H, NH), 8.08 (d, *J* = 6.9 Hz, 1H, H-2), 8.04 (d, *J* = 8.5 Hz, 2H, H-2'), 7.54 (d, *J* = 8.6 Hz, 2H, H-3'), 7.42 (dd, *J* = 3.6, 2.3 Hz, 1H, H-8), 7.06 – 7.01 (m, 1H, H-3), 6.94 (d, *J* = 6.9 Hz, 1H, H-9).

^{13}C-NMR (DMSO, 101 MHz) δ = 166.70, 149.26, 142.37, 138.12, 134.97, 130.85, 127.38, 124.45, 122.73, 122.64, 110.22, 101.16, 98.85.

General procedure E: amide coupling

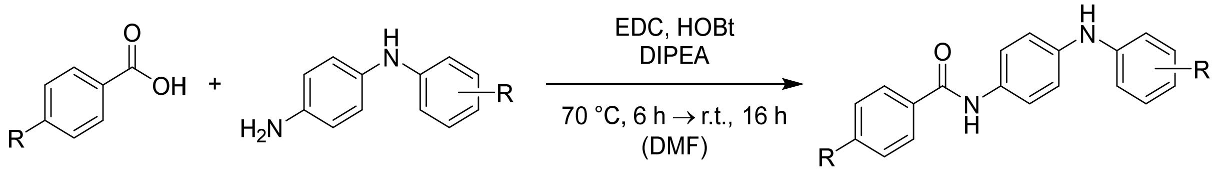

Benzoic acid (1.00 eq) and aryl amine (1.00 eq) were dissolved in 5 mL anhydrous DMF. DIPEA (2.50 eq), EDC hydrochloride (2.00 eq) and HOBt (1.20 eq) were added, and the reaction mixture was stirred at 70 °C for six hours and at room temperature overnight. The solvent was removed under reduced pressure and crude product was purified by silica gel column chromatography. Unless otherwise stated, final compounds were further purified by precipitation in an isopropanol/water mixture. Therefore, the product was dissolved in isopropanol (1 - 2 mL) under heat exposure and subsequently placed on ice. The pure product was precipitated by the addition of 15 – 20 mL water. The suspension was centrifuged (20 min, 4000 rpm, 4 °C), washed once with 10 mL water and finally dried under reduced pressure.

_N_-(4-((2-Amino-6-methylpyrimidin-4-yl)amino)phenyl)-4-(quinolin-4-ylamino)benzamide (1)

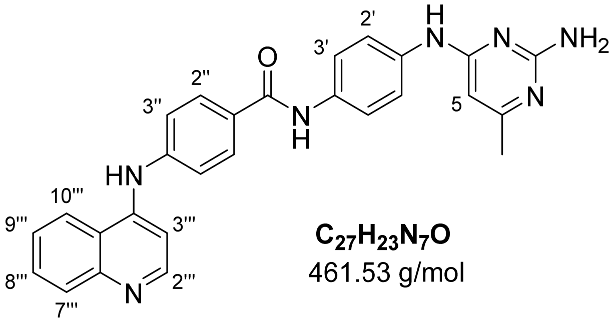

Following general procedure E, **90** (49.0 mg, 185 µmol, 1.00 eq), **73** (40.0 mg, 185 µmol, 1.00 eq), EDC hydrochloride (71.0 mg, 370 µmol, 2.00 eq), HOBt (30.0 mg, 222 µmol, 1.20 eq), and TEA (85.0 µL, 62.5 mg, 463 µmol, 2.50 eq) in 2 mL DMF were stirred at 70 °C for six hours and at room temperature overnight. The crude product was purified by silica gel column chromatography [2% MeOH/1% TEA/DCM → 10% MeOH/1% TEA/DCM] to yield **1** (42.0 mg, 90.7 µmol, 49%) as a light orange solid.

^{1}H-NMR (DMSO, 400 MHz) δ = 10.08 (s, 1H, NH), 9.26 (s, 1H, NH), 8.98 (s, 1H, NH), 8.57 (d, J = 5.22 Hz, 1H, N-2‘“), 8.37 (d, J = 8.30 Hz, 1H, H-10‘“), 8.00 (d, J = 8.71 Hz, 2H, H-3“), 7.93 (d, J = 8.5 Hz, 1H, H-7‘“), 7.74 (t, J = 7.35 Hz, 1H, H-8‘“), 7.66 (s, 4H, H-2‘, H-3‘), 7.58 (t, J = 7.35 Hz, 1H, H-9‘), 7.47 (d, J = 8.71 Hz, 2H, H-2“), 7.20 (d, J = 5.22 Hz, 1H, H-3‘“), 6.11 (s, 2H, NH_2), 5.86 (s, 1H, H-5), 2.08 (s, 3H, CH_3).

HR-MS m/z calcd. for $C_{27}H_{24}N_7O^+$ $(M + 1)^+$ 462.2023, found 462.2045.

These data are in accordance with previous literature reports.[3]

N-(4-((2-Aminopyrimidin-4-yl)amino)phenyl)-4-(quinolin-4-ylamino)benzamide (26)

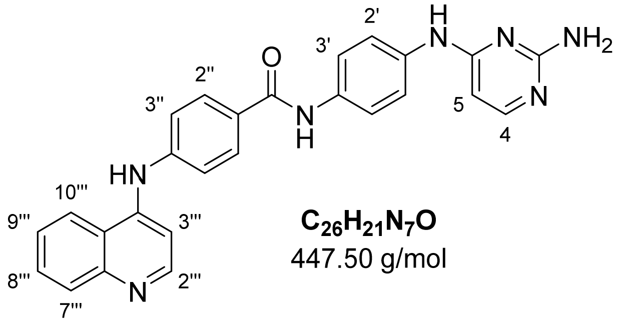

$C_{26}H_{21}N_7O$
447.50 g/mol

Following general procedure E, **90** (92.0 mg, 347 µmol, 1.00 eq), **74** (70.0 mg, 347 µmol, 1.00 eq), TEA (120 µL, 88.0 mg, 868 µmol, 2.50 eq), EDC hydrochloride (133 mg, 694 µmol, 2.00 eq), and HOBt (56.0 mg, 416 µmol,1.20 eq) in 4 mL DMF were heated to 70°C for 6 hours and at room temperature overnight. The crude product was purified by silica gel column chromatography [1% MeOH/1% TEA/DCM → 10% MeOH/1% TEA/DCM] and precipitated with isopropanol (2 mL) and water (15 mL) to yield **26** (42.0 mg, 90.7 µmol, 49%) as a light yellow solid.

TLC: R_f = 0.37 (10% MeOH/1% TEA/ DCM) [UV].

^{1}H-NMR (DMSO, 400 MHz) δ= 10.06 (s, 1H, NH), 9.23 (s, 1H, NH), 9.05 (s, 1H, NH), 8.58 (d, J = 5.2 Hz, 1H, H-2‘‘‘), 8.38 (d, J = 8.4 Hz, 1H, H-10‘‘‘), 8.01 (d, J = 8.3 Hz, 2H, H-3‘‘), 7.93 (d, J = 8.3 Hz, 1H, H-7‘‘‘), 7.79 (d, J = 5.7 Hz, 1H, H-4), 7.74 (t, J = 7.7 Hz, 1H, H-8‘‘‘), 7.69 (s, 4H, H-3‘, H-2‘), 7.58 (t, J = 7.6 Hz, 1H, H-9‘‘‘), 7.48 (d, J = 8.3 Hz, 2H, H-2‘‘), 7.20 (d, J = 5.2 Hz, 1H, H-3‘‘‘), 6.18 (s, 2H, NH_2), 5.98 (d, J = 5.7 Hz, 1H, H-5).

^{13}C-NMR (DMSO, 101 MHz) δ = 164.52, 163.04, 160.79, 156.20, 150.76, 149.08, 146.33, 144.22, 136.38, 133.37, 129.46, 129.31, 129.08, 128.72, 125.02, 122.30, 120.82, 120.41, 119.69, 119.66, 103.67, 96.44.

HR-MS m/z calcd. for $C_{26}H_{22}N_7O^+$ $(M + 1)^+$ 448.1880, found 448.1884.

4-(Quinolin-4-ylamino)-*N*-(4-((5-(trifluoromethyl)pyridin-2-yl)amino)phenyl)benzamide (27)

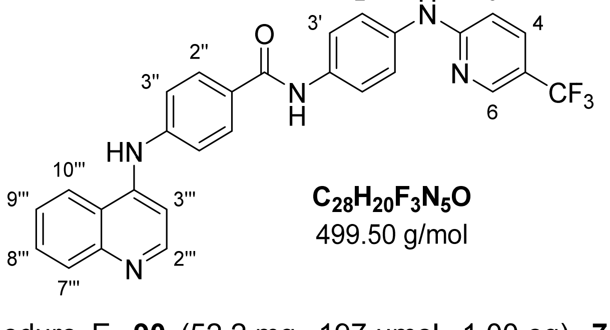

$C_{28}H_{20}F_3N_5O$
499.50 g/mol

Following general procedure E, **90** (52.2 mg, 197 µmol, 1.00 eq), **75** (50.0 mg, 197 µmol, 1.00 eq), TEA (68.0 µL, 50.0 mg, 493 µmol, 2.50 eq), EDC hydrochloride (76.0 mg, 394 µmol, 2.00 eq), and HOBt (32.0 mg, 236 µmol,1.20 eq) in 3 mL DMF were heated to 70°C for 6 hours

and at room temperature overnight. The crude product was purified by silica gel column chromatography [1% MeOH/1% TEA/DCM → 4% MeOH/1% TEA/DCM] and precipitated with isopropanol (2 mL) and water (15 mL) to yield **27** (7.00 mg, 14.1 µmol, 7%) as a light yellow solid.

TLC: R_f = 0.24 (5% MeOH/1% TEA/ DCM) [UV].

^{1}H-NMR (MeOD, 400 MHz) δ = 8.49 (d, J = 5.7 Hz, 1H, H-2“), 8.38 (s, 1H, H-6), 8.35 (s, 1H, H-10“‘), 8.04 (d, J = 8.6 Hz, 2H, H-3“), 7.94 (dd, J = 8.5, 1.2 Hz, 1H, H-7“‘), 7.85 – 7.77 (m, 1H, H-8“‘), 7.73 (dd, J = 8.9, 2.6 Hz, 1H, H-4), 7.69 – 7.58 (m, 5H, H-2‘, H-3‘, H-9“‘), 7.53 (d, J = 8.7 Hz, 2H, H-2“), 7.23 (d, J = 5.7 Hz, 1H, H-3“‘), 6.87 (d, J = 8.9 Hz, 1H, H-3).

^{13}C-NMR (MeOD, 101 MHz) δ = 168.11, 160.19, 151.09, 150.12, 148.18, 146.55, 145.18, 138.46, 135.46, 135.00, 132.15, 131.80, 130.50, 127.95, 127.35, 123.30, 123.28, 122.82, 121.83, 121.63, 111.04, 104.11.

HR-MS m/z calcd. for $C_{28}H_{21}F_3N_5O^+$ (M + 1)$^+$ 500.1693, found 500.1694.

4-(Quinolin-4-ylamino)-*N*-(4-((4-(trifluoromethyl)phenyl)amino)phenyl)benzamide (28)

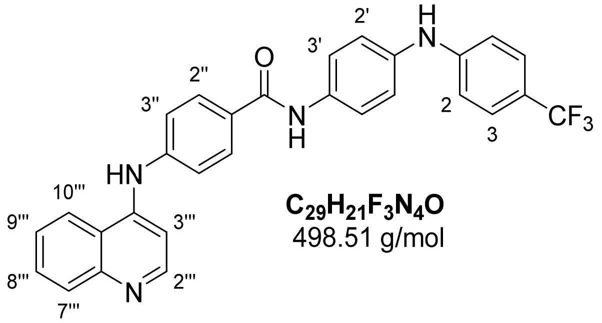

$C_{29}H_{21}F_3N_4O$
498.51 g/mol

Following general procedure E, **90** (73.0 mg, 277 µmol, 1.00 eq), **76** (70.0 mg, 277 µmol, 1.00 eq), TEA (95.0 µL, 70.0 mg, 690 µmol, 2.50 eq), EDC hydrochloride (106 mg, 554 µmol, 2.00 eq), and HOBt (45.0 mg, 330 µmol, 1.20 eq) in 3 mL DMF were heated to 70°C for 4 hours and at room temperature overnight. The crude product was purified by silica gel column chromatography [1% MeOH/1% TEA/ DCM → 4% MeOH/1% TEA/ DCM] and precipitated with isopropanol (2 mL) and water (15 mL) to yield **28** (11.0 mg, 22.1 µmol, 8%) as a light yellow solid.

TLC: R_f = 0.33 (5% MeOH/1% TEA/ DCM) [UV].

^{1}H-NMR (MeOD, 400 MHz) δ = 8.49 (d, J = 5.7 Hz, 1H, H-2“‘), 8.37 (d, J = 8.5 Hz, 1H, H-10“‘), 8.04 (d, J = 8.7 Hz, 2H, H-3“), 7.94 (d, J = 8.5 Hz, 1H, H-7“‘), 7.87 – 7.77 (m, 1H, H-8“‘), 7.74 – 7.59 (m, 3H, H-3, H-9“‘), 7.53 (d, J = 8.6 Hz, 2H, H-2“), 7.44 (d, J = 8.5 Hz, 2H, H-3‘), 7.26 – 7.16 (m, 3H, H-2, H-3“‘), 7.10 (d, J = 8.6 Hz, 2H, H-2‘).

^{13}C-NMR (MeOD, 101 MHz) δ = 158.41, 141.54, 140.27, 139.93, 138.27, 135.40, 130.50, 124.74, 122.55, 122.16, 120.81, 118.09, 118.02, 117.98, 117.70, 114.24, 113.63, 113.21, 111.87, 111.69, 106.11, 94.35.

HR-MS m/z calcd. for $C_{29}H_{22}F_3N_4O^+$ $(M + 1)^+$ 499.1740, found 499.1743.

4-(Quinolin-4-ylamino)-*N*-(4-((2-(trifluoromethyl)phenyl)amino)phenyl)benzamide (29)

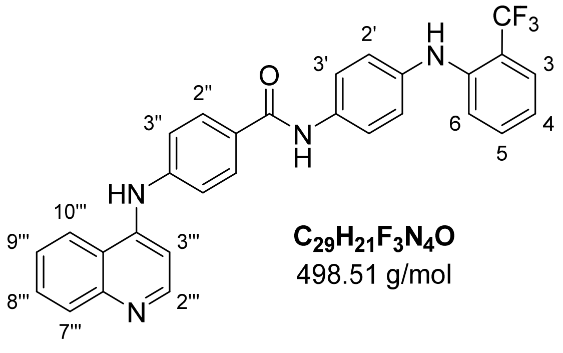

Following general procedure E, **90** (73.0 mg, 277 µmol, 1.00 eq), **77** (70.0 mg, 277 µmol, 1.00 eq), DIPEA (117 µL, 89.0 mg, 690 µmol, 2.50 eq), EDC hydrochloride (106 mg, 554 µmol, 2.00 eq), and HOBt (45.0 mg, 330 µmol, 1.20 eq) in 3 mL DMF were heated to 70°C for 6 hours and at room temperature overnight. The crude product was purified by silica gel column chromatography [1% MeOH → 2% MeOH/1% TEA/ DCM] and precipitated with isopropanol (2 mL) and water (15 mL) to yield **29** (96.0 mg, 216 µmol, 78%) as a light yellow solid.

TLC: R_f = 0.32 (5% MeOH/1% TEA/ DCM) [UV].

^{1}H-NMR (DMSO, 400 MHz) δ = 10.09 (s, 1H, NH), 9.25 (s, 1H, NH), 8.58 (d, J = 5.2 Hz, 1H, H-2‘‘‘), 8.39 (d, J = 8.5 Hz, 1H, H-10‘‘‘), 8.02 (d, J = 8.7 Hz, 2H, H-3‘‘), 7.94 (d, J = 8.4 Hz, 1H, H-7‘‘‘), 7.74 (t, J = 8.5, 1H, H-9‘‘‘), 7.70 (d, J = 8.8 Hz, 2H, H-3‘), 7.64 – 7.54 (m, 2H, H-H-8‘‘‘, H-4*), 7.48 (d, J = 8.7 Hz, 2H, H-2‘‘), 7.46 (d, 1H, H-3), 7.38 (s, 1H, NH), 7.23 (d, J = 8.5 Hz, 1H, H-6), 7.21 (d, J = 5.2 Hz, 1H, H-3‘‘‘), 7.09 (d, J = 8.9 Hz, 2H, H-2‘), 7.02 (t, J = 7.6 Hz, 1H, H-5*).

^{13}C-NMR (DMSO, 101 MHz) δ = 164.51, 150.67, 148.97, 146.42, 142.83, 138.66, 133.62, 133.31, 129.49, 129.07, 128.68, 126.81, 125.86, 125.02, 123.15, 122.32, 121.53, 121.43, 120.31, 120.11, 119.72, 119.62, 117.75, 117.46, 103.62.

HR-MS m/z calcd. for $C_{29}H_{22}F_3N_4O^+$ $(M + 1)^+$ 499.1740, found 499.1739.

4-(Quinolin-4-ylamino)-*N*-(4-(*p*-tolylamino)phenyl)benzamide (30)

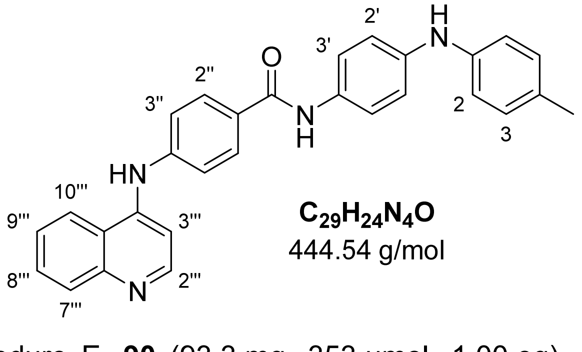

Following general procedure E, **90** (93.3 mg, 353 µmol, 1.00 eq), **78** (70.0 mg, 353 µmol, 1.00 eq), DIPEA (150 µL, 114 mg, 880 µmol, 2.50 eq), EDC hydrochloride (135 mg, 706 µmol,

2.00 eq), and HOBt (56.8 mg, 330 µmol, 1.20 eq) in 4 mL DMF were heated to 70°C for 6 hours and at room temperature overnight. The crude product was purified by silica gel column chromatography [0.5% MeOH → 1% MeOH/1% TEA/ DCM] and precipitated with isopropanol (2 mL) and water (15 mL) to yield **30** (62.0 mg, 137 µmol, 39%) as a light yellow solid.

TLC: R_f = 0.33 (5% MeOH/1% TEA/ DCM) [UV].

^{1}H-NMR (DMSO, 400 MHz) δ = 10.03 (s, 1H, NH), 8.58 (d, J = 5.5 Hz, 1H, H-2‘‘‘), 8.45 (d, J = 8.6 Hz, 1H, H-10‘‘‘), 8.03 (d, J = 8.7 Hz, 2H, H-3‘‘), 7.99 – 7.88 (m, 2H, NH, H-7‘‘‘), 7.87 – 7.73 (m, 1H, H-8‘‘‘), 7.69 – 7.57 (m, 3H, H-3‘, H-9‘‘‘), 7.50 (d, J = 8.7 Hz, 2H, H-2‘‘), 7.16 (d, J = 5.5 Hz, 1H, H-3‘‘‘), 7.04 (d, J = 8.4 Hz, 2H, H-3), 7.02 (d, J = 8.7 Hz, 2H, H-2‘), 6.96 (d, J = 8.4 Hz, 2H, H-2), 2.22 (s, 3H, CH_3).

^{13}C-NMR (DMSO, 101 MHz) δ = 164.26, 149.46, 147.73, 143.48, 141.27, 139.90, 131.43, 130.20, 129.58, 129.05, 128.09, 127.81, 125.36, 122.52, 121.81, 120.52, 119.93, 116.72, 116.61, 103.05, 20.28.

HR-MS m/z calcd. for $C_{29}H_{25}N_4O^+$ $(M + 1)^+$ 445.2020, found 445.2029.

N-(4-(Phenylamino)phenyl)-4-(quinolin-4-ylamino)benzamide (31)

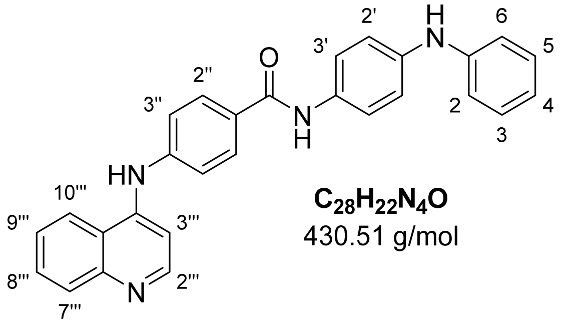

$C_{28}H_{22}N_4O$
430.51 g/mol

Following general procedure E, **90** (100 mg, 380 µmol, 1.00 eq), **79** (70.0 mg, 380 µmol, 1.00 eq), DIPEA (161 µL, 123 mg, 950 µmol, 2.50 eq), EDC hydrochloride (146 mg, 760 µmol, 2.00 eq), and HOBt (62.0 mg, 460 µmol, 1.20 eq) in 4 mL DMF were heated to 70°C for 6 hours and at room temperature overnight. The crude product was purified by silica gel column chromatography [1% MeOH/1% TEA/ DCM] and precipitated with isopropanol (2 mL) and water (15 mL) to yield **31** (127 mg, 296 µmol, 78%) as a white solid.

TLC: R_f = 0.32 (5% MeOH/1% TEA/ DCM) [UV].

^{1}H-NMR (DMSO, 400 MHz) δ = 10.03 (s, 1H, NH), 9.24 (s, 1H, NH), 8.58 (d, J = 5.2 Hz, 1H, H-2‘‘‘), 8.39 (d, J = 8.5, 1.4 Hz, 1H, H-10‘‘‘), 8.08 (s, 1H, NH), 8.01 (d, J = 8.7 Hz, 2H, H-3‘‘), 7.93 (d, J = 8.5 Hz, 1H, H-7‘‘‘), 7.74 (t, J = 8.3 Hz, 1H, H-8‘‘‘), 7.65 (d, J = 8.9 Hz, 2H, H-3‘), 7.58 (t, J = 7.6 Hz, 1H, H-9‘‘‘), 7.48 (d, J = 8.7 Hz, 2H, H-2‘‘), 7.23 (d, J = 8.7 Hz, 1H, H-3‘‘‘), 7.20 (d, J = 7.5 Hz, 2H, H-2, H-6), 7.08 (d, J = 8.9 Hz, 2H, H-2‘), 7.04 (d, J = 7.5 Hz, 2H, H-3, H-5), 6.78 (t, J = 7.3 Hz, 1H, H-4).

^{13}C-NMR (DMSO, 101 MHz) δ = 164.38, 150.70, 144.03, 139.09, 132.09, 129.48, 129.16, 129.03, 128.80, 125.01, 122.31, 121.73, 120.39, 119.75, 119.05, 117.57, 115.88, 103.58.

HR-MS m/z calcd. for $C_{28}H_{23}N_4O^+$ $(M + 1)^+$ 431.1866, found 431.1869.

***N*-(4-((4-Methoxyphenyl)amino)phenyl)-4-(quinolin-4-ylamino)benzamide (32)**

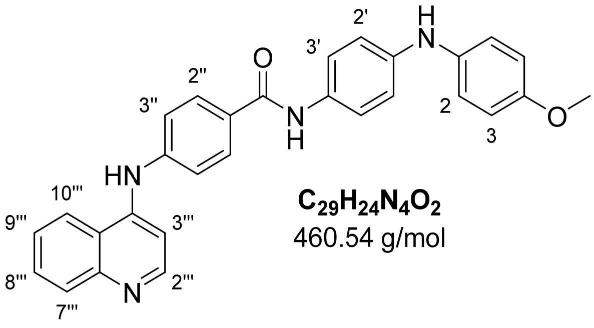

Following general procedure E, **90** (86.0 mg, 326 µmol, 1.00 eq), **80** (70.0 mg, 326 µmol, 1.00 eq), DIPEA (138 µL, 105 mg, 815 µmol, 2.50 eq), EDC hydrochloride (125 mg, 654 µmol, 2.00 eq), and HOBt (53.0 mg, 390 µmol, 1.20 eq) in 4 mL DMF were heated to 70°C for 6 hours and at room temperature overnight. The crude product was purified by silica gel column chromatography [1% MeOH/1% TEA/ DCM] and precipitated with isopropanol (2 mL) and water (15 mL) to yield **32** (82.0 mg, 179 µmol, 55%) as a white solid.

TLC: R_f = 0.29 (5% MeOH/1% TEA/ DCM) [UV].

^{1}H-NMR (DMSO, 400 MHz) δ = 9.96 (s, 1H, NH), 9.23 (s, 1H, NH), 8.57 (d, J = 5.2 Hz, 1H, H-2‘‘‘), 8.39 (d, J = 8.5, 1H, H-10‘‘‘), 8.00 (d, J = 8.6 Hz, 2H, H-3‘‘), 7.93 (d, J = 8.4 Hz, 1H, H-7‘‘‘), 7.78 (s, 1H, NH), 7.73 (t, J = 7.6 Hz, 1H, H-8‘‘), 7.65 – 7.53 (m, 3H, H-3‘, H-9‘‘‘), 7.47 (d, J = 8.6 Hz, 2H, H-2‘‘), 7.19 (d, J = 5.1 Hz, 1H, H-3‘‘‘), 7.03 (d, J = 9.0 Hz, 2H, H-2), 6.94 (d, J = 9.0 Hz, 2H, H-2‘), 6.86 (d, J = 8.9 Hz, 1H, H-3), 3.71 (s, 1H, CH_3).

^{13}C-NMR (DMSO, 101 MHz) δ = 164.27, 153.41, 150.73, 149.04, 146.41, 144.06, 141.00, 136.81, 130.84, 129.47, 129.27, 128.99, 128.88, 125.00, 122.31, 121.94, 120.40, 119.76, 119.40, 115.41, 114.58, 103.56, 55.24.

HR-MS m/z calcd. for $C_{29}H_{25}N_4O_2^+$ $(M + 1)^+$ 461.1972, found 461.1973.

***N*-(4-((4-Fluorophenyl)amino)phenyl)-4-(quinolin-4-ylamino)benzamide (33)**

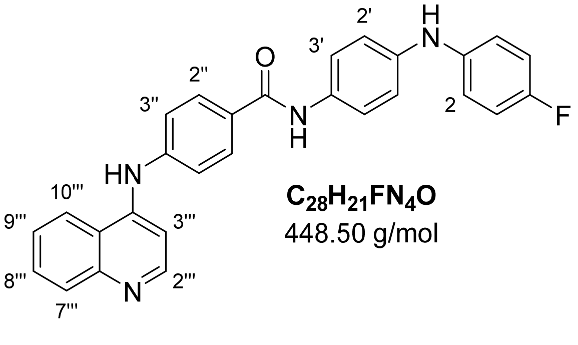

Following general procedure E, **90** (91.0 mg, 346 µmol, 1.00 eq), **81** (70.0 mg, 346 µmol, 1.00 eq), DIPEA (148 µL, 112 mg, 870 µmol, 2.50 eq), EDC hydrochloride (133 mg, 692 µmol, 2.00 eq), and HOBt (57.0 mg, 420 µmol, 1.20 eq) in 4 mL DMF were heated to 70°C for 6 hours and at room temperature overnight. The crude product was purified by silica gel column chromatography [1% MeOH/1% TEA/ DCM] and precipitated with isopropanol (2 mL) and water (15 mL) to yield **33** (101 mg, 225 µmol, 65%) as a yellow solid.

TLC: R_f = 0.37 (5% MeOH/1% TEA/ DCM) [UV].

^{1}H-NMR (DMSO, 400 MHz) δ = 10.14 (s, 1H, NH), 8.62 (d, J = 8.6 Hz, 1H, H-10'''), 8.58 (d, J = 6.0 Hz, 1H, H-2'''), 8.09 (d, J = 5.4 Hz, 2H, H-3''), 8.07 (s, 1H, NH), 8.01 (dd, J = 8.5, 1.3 Hz, 1H, H-7'''), 7.89 (t, J = 8.3 Hz, 1H, H-8'''), 7.70 (t, J = 7.8 Hz, 1H, H-9'''), 7.65 (d, J = 9.0 Hz, 2H, H-3'), 7.56 (d, J = 8.6 Hz, 2H, H-2''), 7.09 (d, J = 6.0 Hz, 1H, H-3'''), 7.07 – 7.02 (m, 6H, H-2', H-2, H-3).

^{13}C-NMR (DMSO, 101 MHz) δ = 164.21, 157.20, 154.86, 150.38, 146.97, 143.84, 142.19, 140.36, 140.34, 139.75, 131.73, 131.68, 131.03, 129.16, 126.08, 124.94, 123.04, 122.12, 121.87, 118.99, 117.91, 117.84, 116.82, 115.77, 115.54, 102.01.

HR-MS m/z calcd. for $C_{28}H_{22}FN_4O^+$ (M + 1)$^+$ 449.1772, found 449.1777.

***N*-(4-(Pyridin-2-ylamino)phenyl)-4-(quinolin-4-ylamino)benzamide (34)**

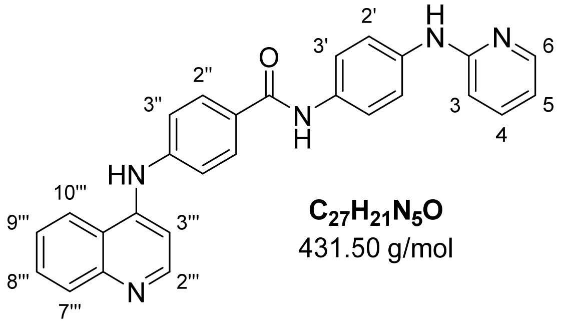

$C_{27}H_{21}N_5O$
431.50 g/mol

Following general procedure E, **90** (100 mg, 377µmol, 1.00 eq), **88** (70.0 mg, 377 µmol, 1.00 eq), DIPEA (160 µL, 121 mg, 940 µmol, 2.50 eq), EDC hydrochloride (145 mg, 754 µmol, 2.00 eq), and HOBt (61.0 mg, 452 µmol, 1.20 eq) in 4 mL DMF were heated to 70°C for 6 hours and at room temperature overnight. The crude product was purified by silica gel column chromatography [1% MeOH → 5% MeOH/1% TEA/ DCM] and precipitated with isopropanol (2 mL) and water (15 mL) to yield **34** (73.0 mg, 170 µmol, 45%) as a white solid.

TLC: R_f = 0.23 (5% MeOH/1% TEA/ DCM) [UV].

^{1}H-NMR (DMSO, 400 MHz) δ = 10.04 (s, 1H, NH), 9.23 (s, 1H, NH), 8.97 (s, 1H, NH), 8.58 (d, J = 5.2 Hz, 1H, H-2'''), 8.39 (d, J = 8.4 Hz, 1H, H-10'''), 8.14 (dd, J = 5.1, 2.0 Hz, 1H, H-6), 8.02 (d, J = 8.5 Hz, 2H, H-3''), 7.93 (d, J = 8.3 Hz, 1H, H-7'''), 7.73 (t, J = 8.3, 1H, H-8'''), 7.66 (d, J = 2.8 Hz, 4H, H-2', H-3'), 7.61 – 7.51 (m, 2H, H-4, H-9'''), 7.48 (d, J = 8.4 Hz, 2H, H-2'), 7.21 (d, J = 5.2 Hz, 1H, H-3'''), 6.81 (d, J = 8.4 Hz, 1H, H-3), 6.75 – 6.67 (m, 1H, H-5).

^{13}C-NMR (DMSO, 101 MHz) δ = 164.40, 155.99, 150.76, 149.08, 147.28, 146.35, 144.15, 137.62, 137.15, 132.39, 129.45, 129.30, 129.05, 128.78, 125.01, 122.30, 121.11, 120.41, 119.71, 118.23, 113.94, 110.38, 103.63, 40.15, 39.99, 39.94, 39.73, 39.52, 39.31, 39.10, 38.89.
HR-MS m/z calcd. for $C_{27}H_{22}N_5O^+$ $(M + 1)^+$ 432.1819, found 432.1820.

N-(4-((6-Aminopyridin-2-yl)amino)phenyl)-4-(quinolin-4-ylamino)benzamide (35)

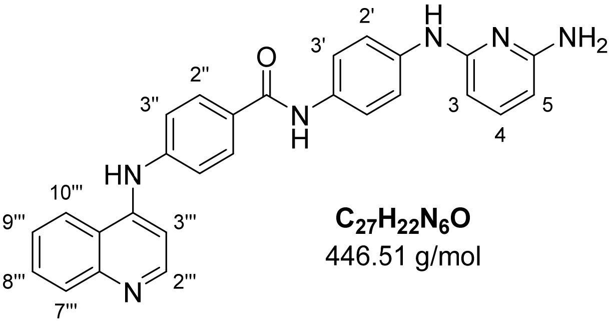

$C_{27}H_{22}N_6O$
446.51 g/mol

Following general procedure E, **90** (198 mg, 750 µmol, 1.00 eq), **82** (150 mg, 750 µmol, 1.00 eq), DIPEA (318 µL, 242 mg, 1.87 mmol, 2.50 eq), EDC hydrochloride (287 mg, 1.50 mmol, 2.00 eq), and HOBt (121 mg, 900 µmol, 1.20 eq) in 6 mL DMF were heated to 70°C for 6 hours and at room temperature overnight. The crude product was purified by silica gel column chromatography [0.5% MeOH → 5% MeOH/1% TEA/ DCM] and precipitated with isopropanol (2 mL) and water (15 mL) to yield **35** (89.0 mg, 203 µmol, 27%) as a light brown solid.
TLC: R_f = 0.14 (5% MeOH/1% TEA/ DCM) [UV].
^{1}H-NMR (DMSO, 400 MHz) δ = 9.99 (s, 1H, NH), 9.23 (s, 1H, NH), 8.58 (d, J = 5.2 Hz, 1H, H-2“‘), 8.50 (s, 1H, NH), 8.38 (d, J = 8.6 Hz, 1H, H-10“‘), 8.01 (d, J = 8.7 Hz, 2H, H-3“), 7.93 (d, J = 8.3 Hz, 1H, H-7“‘), 7.74 (t, J = 7.0 Hz, 1H, H-9“‘), 7.67 – 7.52 (m, 5H, H-2‘, H-3‘, H-8“‘), 7.48 (d, J = 8.3 Hz, 2H, H-2“), 7.26 – 7.13 (m, 2H, H-4, H-3“‘), 5.98 (d, J = 7.8 Hz, 1H, H-3), 5.82 (d, J = 7.8 Hz, 1H, H-5), 5.65 (s, 2H, NH_2).
^{13}C-NMR (DMSO, 101 MHz) δ = 164.37, 158.45, 155.10, 150.77, 149.07, 146.40, 144.09, 138.42, 138.25, 131.69, 129.48, 129.30, 129.03, 128.91, 125.03, 122.31, 121.05, 120.41, 119.76, 117.93, 103.60, 97.11, 96.96.

N-(4-((2-Aminopyridin-4-yl)amino)phenyl)-4-(quinolin-4-ylamino)benzamide (36)

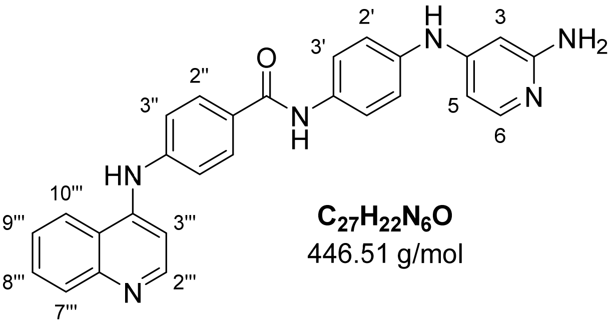

$C_{27}H_{22}N_6O$
446.51 g/mol

Following general procedure E, **90** (92.0 mg, 350 µmol, 1.00 eq), **83** (70.0 mg, 350 µmol, 1.00 eq), DIPEA (152 µL, 113 mg, 875 µmol, 2.50 eq), EDC hydrochloride (137 mg, 700 µmol, 2.00 eq), and HOBt (57.0 mg, 420 µmol, 1.20 eq) in 4 mL DMF were heated to 70°C for 6 hours and at room temperature overnight. The crude product was purified by silica gel column chromatography [3% MeOH/1% TEA/ DCM] and precipitated with isopropanol (2 mL) and water (15 mL) to yield **36** (65.0 mg, 147 µmol, 42%) as a light yellow solid.

TLC: R_f = 0.38 (10% MeOH/1% TEA/ DCM) [UV].

^{1}H-NMR (DMSO, 400 MHz) δ = 10.10 (s, 1H, NH), 9.24 (s, 1H, NH), 8.58 (d, J = 5.2 Hz, 1H, H-2‘‘‘), 8.38 (d, J = 8.5 Hz, 1H, H-10‘‘‘), 8.30 (s, 1H, NH), 8.01 (d, J = 8.7 Hz, 2H, H-3‘‘), 7.93 (d, J = 8.3 Hz, 1H, H-7‘‘‘), 7.73 (t, 1H, H-8‘‘‘), 7.72 (d, J = 8.9 Hz, 2H, H-3‘), 7.59 (d, 1H, H-6), 7.57 (t, 1H, H-9‘‘‘), 7.48 (d, J = 8.3 Hz, 2H, H-2‘‘), 7.21 (d, J = 5.2 Hz, 1H, H-3‘‘‘), 7.13 (d, J = 8.9 Hz, 2H, H-2‘), 6.12 (dd, J = 5.8, 2.0 Hz, 1H, H-5), 6.04 (d, J = 2.0 Hz, 1H, H-3), 5.52 (s, 2H, NH_2).

^{13}C-NMR (DMSO, 101 MHz) δ = 164.57, 160.72, 151.44, 150.76, 149.07, 148.07, 146.33, 144.27, 136.86, 133.78, 129.47, 129.30, 129.10, 128.64, 125.02, 122.31, 121.38, 120.61, 120.43, 119.68, 103.69, 101.15, 90.31.

HR-MS m/z calcd. for $C_{27}H_{23}N_6O^+$ (M + 1)$^+$ 447.1928, found 447.1958.

***N*-(4-((3-Hydroxyphenyl)amino)phenyl)-4-(quinolin-4-ylamino)benzamide (40)**

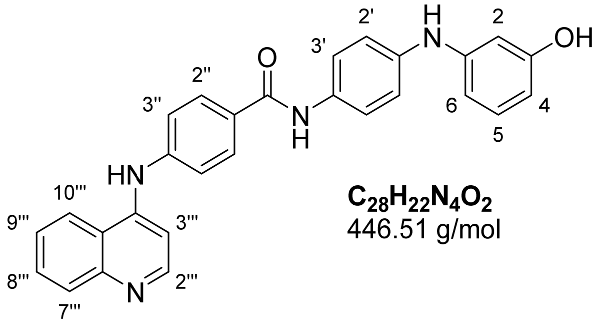

$C_{28}H_{22}N_4O_2$
446.51 g/mol

Following general procedure E, **90** (91.0 mg, 340 µmol, 1.00 eq), **89** (69.0 mg, 340 µmol, 1.00 eq), DIPEA (148 µL, 110 mg, 850 µmol, 2.50 eq), EDC hydrochloride (130 mg, 680 µmol, 2.00 eq), and HOBt (55.0 mg, 408 µmol, 1.20 eq) in 4 mL DMF were heated to 70°C for six hours and at room temperature overnight. The crude product was purified by silica gel column chromatography [2% MeOH → 5% MeOH/1% TEA/ DCM] and precipitated with isopropanol (2 mL) and water (15 mL) to yield **40** (50.0 mg, 112 µmol, 33%) as light yellow crystals.

TLC: R_f = 0.22 (5% MeOH/1% TEA/ DCM) [UV].

^{1}H-NMR (MeOD, 400 MHz) δ = 8.45 (d, J = 5.6 Hz, 1H, H-2‘‘‘), 8.27 (d, J = 8.4 Hz, 1H, H-10‘‘‘), 7.96 (d, J = 8.6 Hz, 1H, H-3‘), 7.91 (d, J = 8.5 Hz, 1H, H-7‘‘‘), 7.71 (t, 1H, H-8‘‘‘), 7.57 – 7.48 (m, 3H, H-3‘, H-9‘‘‘), 7.44 (d, 2H, J = 8.51 Hz, H-2‘‘), 7.22 – 7.16 (m, 1H, H-3‘), 7.08 (d, J = 8.8

Hz, 2H, H-2‘), 7.00 (t, J = 8.0 Hz, 1H, H-5), 6.60 – 6.56 (m, 1H, H-2), 6.53 (dd, J = 8.0, 2.1 Hz, 1H, H-6), 6.29 (dd, J = 7.7, 1.7 Hz, 1H, H-4).

^{13}C-NMR (MeOD, 101 MHz) δ = 167.92, 159.26, 151.24, 149.70, 149.39, 146.71, 145.54, 142.06, 132.35, 131.07, 130.91, 130.80, 130.72, 130.26, 130.13, 129.13, 126.67, 123.83, 122.97, 122.81, 121.85, 121.76, 121.61, 119.12, 109.65, 108.09, 104.63, 104.32.

HR-MS m/z calcd. for $C_{28}H_{23}N_4O_2^+$ (M + 1)$^+$ 447.1816, found 447.1843.

***tert*-Butyl (*tert*-butoxycarbonyl)(2-fluoro-5-((4-(4-(quinolin-4ylamino)benzamido)phenyl) amino)phenyl)carbamate (94)**

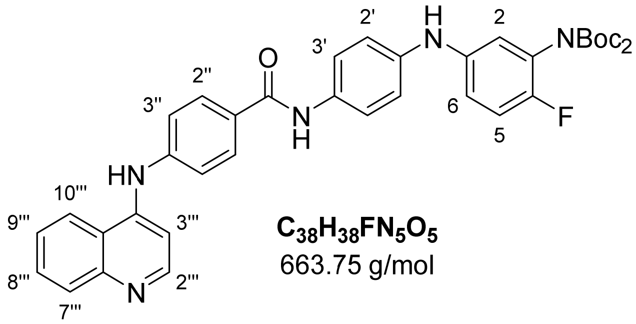

$C_{38}H_{38}FN_5O_5$
663.75 g/mol

Following general procedure E, **90** (53.0 mg, 198 µmol, 1.00 eq), **84** (83.0 mg, 198 µmol, 1.00 eq), DIPEA (86.0 µL, 64.0 mg, 495 µmol, 2.50 eq), EDC hydrochloride (76.0 mg, 396 µmol, 2.00 eq), and HOBt (32.0 mg, 238 µmol, 1.20 eq) in 4 mL DMF wer heated to 70°C for six hours and at room temperature overnight. The crude product was purified by silica gel column chromatography [2% MeOH/1% TEA/ DCM] and precipitated with isopropanol (2 mL) and water (15 mL) to yield **94** (82.0 mg, 124 µmol, 63%) as light yellow crystals.

TLC: R_f = 0.23 (5% MeOH/1% TEA/ DCM) [UV].

^{1}H-NMR (MeOD, 400 MHz) δ = 8.49 (d, J = 5.4 Hz, 1H, H-2‘‘‘), 8.31 (dd, J = 8.7, 1.3 Hz, 1H, H-10‘‘‘), 8.00 (d, J = 8.7 Hz, 1H, H-3‘‘), 7.94 (dd, J = 8.1, 1.0 Hz, 1H, H-7‘‘‘), 7.75 (ddd, J = 8.4, 6.8, 1.3 Hz, 1H, H-8‘‘‘), 7.62 – 7.54 (m, 3H, H-3‘, H-9‘‘‘), 7.50 (d, J = 8.7 Hz, 2H, H-2‘‘), 7.24 (d, J = 5.5 Hz, 1H, H-3‘‘‘), 7.12 – 6.96 (m, 4H, H-5, H-6, H-2‘), 6.95 – 6.88 (m, 1H, H-2), 1.43 (s, 18H, Boc_2).

^{13}C-NMR (MeOD, 101 MHz) δ = 167.95, 154.42, 152.55, 152.03, 151.19, 149.65, 149.60, 145.61, 142.21, 142.03, 132.78, 131.18, 130.82, 130.17, 129.08, 128.57, 128.42, 126.74, 123.95, 122.87, 121.89, 119.55, 119.45, 118.56, 118.32, 117.09, 116.88, 104.34, 84.43, 49.71, 49.64, 49.50, 49.43, 49.21, 49.00, 48.79, 48.57, 48.36, 28.07.

***tert*-Butyl (*tert*-butoxycarbonyl)(3-fluoro-5-((4-(4-(quinolin-4-ylamino)benzamido)phenyl)amino)phenyl)carbamate (95)**

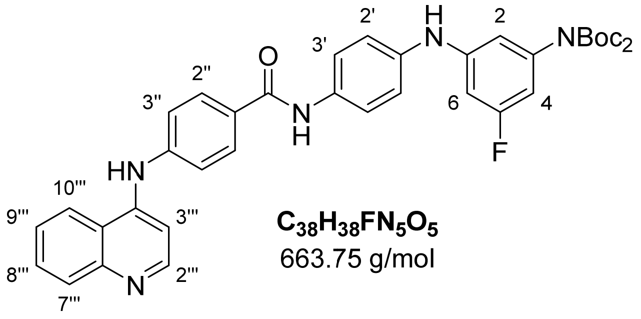

$C_{38}H_{38}FN_5O_5$
663.75 g/mol

Compound **63** (161 mg, 380 μmol, 1.00 eq) was stirred with 10 wt% Pd/C (49.0 mg, 460 μmol, 1.20 eq), and ammonium formate (240 mg, 3.80 mmol, 10.0 eq) in 10 mL EtOH overnight. After filtration and washing with EtOAc, the crude intermediate was directly subjected to amide coupling using general procedure F. To a solution of **90** (70.0 mg, 265 μmol, 1.00 eq) and the crude intermediate (84.0 mg, 265 μmol, 1.00 eq) in 4 mL DMF were added NEt_3 (92.0 μL, 67.0 mg, 663 μmol, 2.50 eq), EDC hydrochloride (101 mg, 530 μmol, 2.00 eq), and HOBt (44.0 mg, 318 μmol, 1.20 eq). The mixture was heated to 70°C for six hours and left at room temperature overnight. The crude product was purified by silica gel column chromatography [2% MeOH/1% TEA/ DCM] and precipitated with isopropanol (2 mL) and water (15 mL) to yield **95** (80.0 mg, 121 μmol, 52% over two steps) as light yellow crystals.

^{1}H-NMR (DMSO, 400 MHz) δ =10.09 (s, 1H, NH), 9.23 (s, 1H, NH), 8.58 (d, *J* = 5.2 Hz, 1H, H-2'''), 8.44 (s, 1H, NH), 8.38 (d, *J* = 8.4 Hz, 1H, H-10'''), 8.01 (d, *J* = 8.6 Hz, 2H, H-3''), 7.93 (d, *J* = 8.4 Hz, 1H, H-7'''), 7.72 (m, 3H, H-3', H-8'''), 7.58 (t, *J* = 7.6 Hz, 1H, H-9'''), 7.48 (d, *J* = 8.5 Hz, 2H, H-2''), 7.21 (d, *J* = 5.1 Hz, 1H, H-3'''), 7.11 (d, *J* = 8.9 Hz, 2H, H-2'), 6.70 (dt, *J* = 11.4, 2.2 Hz, 1H, H-6), 6.57 (s, 1H, H-4), 6.46 (dt, *J* = 9.4, 2.1 Hz, 1H, H-2), 1.42 (s, 18H, Boc_2).

***N*-(4-((2-Amino-6-(trifluoromethyl)pyrimidin-4-yl)amino)phenyl)-4-(quinolin-4-ylamino) benzamide (41)**

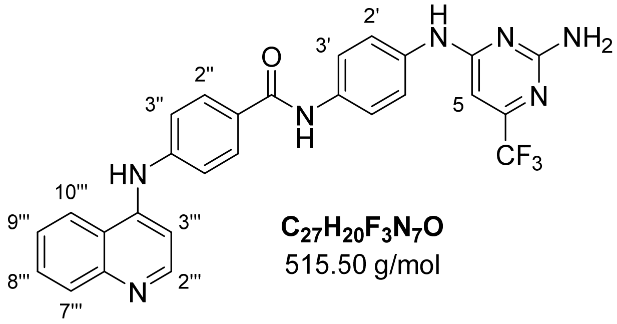

$C_{27}H_{20}F_3N_7O$
515.50 g/mol

Following general procedure E, **90** (69.0 mg, 260 μmol, 1.00 eq), **85** (70.0 mg, 260 μmol, 1.00 eq), DIPEA (113 μL, 84.0 mg, 650 μmol, 2.50 eq), EDC hydrochloride (100 mg, 520 μmol, 2.00 eq), and HOBt (42.0 mg, 310 μmol, 1.20 eq) in 4 mL DMF were heated to 70°C for six hours and at room temperature overnight. The crude product was purified by silica gel column

chromatography [3% MeOH → 5% MeOH/1% TEA/ DCM] and precipitated with isopropanol (2 mL) and water (15 mL) to yield **41** (24.0 mg, 46.8 µmol, 18%) as a light yellow solid.

^{1}H-NMR (DMSO, 400 MHz) δ = 10.20 (s, 1H, NH), 9.62 (s, 1H, NH), 8.59 (d, J = 5.8 Hz, 1H, H-2‘‘‘), 8.54 (d, J = 8.4 Hz, 1H, H-10‘‘‘), 8.07 (d, J = 8.3 Hz, 2H, H-3‘‘), 7.98 (d, J = 8.4 Hz, 1H, H-7‘‘‘), 7.85 (t, J = 7.7 Hz, 1H, H-8‘‘‘), 7.75 (s, 4H, H-2‘, H-3‘), 7.68 (t, J = 8.0 Hz, 1H, H-9‘‘‘), 7.55 (d, J = 8.4 Hz, 2H, H-2‘‘), 7.13 (d, J = 5.8 Hz, 1H, H-3‘‘‘), 6.83 (s, 2H, NH_2), 6.36 (s, 1H, H-5).

^{13}C-NMR (DMSO, 101 MHz) δ = 164.49, 163.48, 161.46, 147.96, 142.81, 135.49, 134.14, 131.11, 130.31, 129.20, 127.21, 126.05, 125.81, 124.43, 122.77, 121.44, 120.82, 120.18, 119.35, 119.09, 109.64, 102.47, 40.15, 39.99, 39.94, 39.73, 39.52, 39.31, 39.10, 38.89.

^{19}F-NMR (DMSO, 376 MHz) δ = -69.88.

HR-MS m/z calcd. for $C_{27}H_{21}F_3N_7O^+$ (M + 1)$^+$ 516.1754, found 516.1791.

***N*-(4-((2-Aminopyrimidin-4-yl)amino)phenyl)-4-(quinazolin-4-ylamino)benzamide (47)**

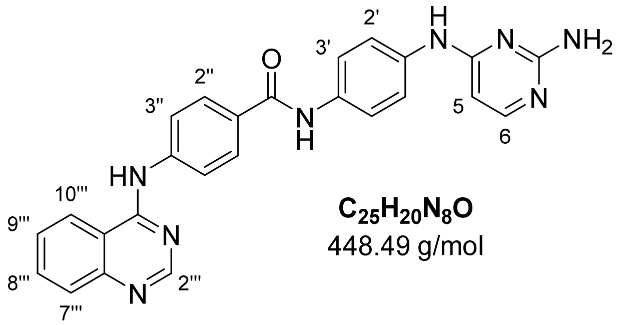

$C_{25}H_{20}N_8O$
448.49 g/mol

Following general procedure E, **92** (210 mg, 790 µmol, 1.00 eq), **74** (170 mg, 790 µmol, 1.00 eq), DIPEA (346 µL, 256 mg, 1.98 mmol, 2.50 eq), EDC hydrochloride (302 mg, 1.58 mmol, 2.00 eq), and HOBt (128 mg, 948 µmol, 1.20 eq) in 8 mL DMF were heated to 70°C for six hours and at room temperature overnight. The crude product was purified by silica gel column chromatography [5% MeOH → 10% MeOH/1% TEA/ DCM] and precipitated with isopropanol (2 mL) and water (15 mL) to yield **47** (136 mg, 300 µmol, 38%) as a light yellow solid.

^{1}H-NMR (DMSO, 400 MHz) δ = 10.08 (s, 1H, NH), 10.00 (s, 1H, NH), 9.05 (s, 1H, NH), 8.70 (s, 1H, H-2‘‘‘), 8.62 (d, J = 8.3 Hz, 1H, H-10‘‘‘), 8.09 (d, J = 8.8 Hz, 2H, H-3‘‘), 8.01 (d, J = 8.8 Hz, 2H, H-2‘‘), 7.90 (t, J = 7.6 Hz, 1H, H-8‘‘‘), 7.84 (d, J = 8.3 Hz, 1H, H-7‘‘‘), 7.79 (d, J = 5.7 Hz, 1H, H-6), 7.71 – 7.67 (m, 1H, H-9‘‘‘), 7.69 (s, 4H, H-2‘, H-3‘), 6.17 (s, 2H, NH_2), 5.99 (d, J = 5.8 Hz, 1H, H-5).

^{13}C-NMR (DMSO, 101 MHz) δ = 164.64, 163.02, 160.78, 157.56, 156.16, 154.31, 149.79, 142.21, 136.39, 133.33, 133.22, 129.58, 128.13, 127.91, 126.48, 123.03, 121.07, 120.79, 119.65, 115.26, 96.42.

HR-MS m/z calcd. for $C_{25}H_{21}N_8O^+$ (M + 1)$^+$ 449.1833, found 449.1855.

N-(4-((2-(Methylamino)pyrimidin-4-yl)amino)phenyl)-4-(quinolin-4-ylamino)benzamide (42)

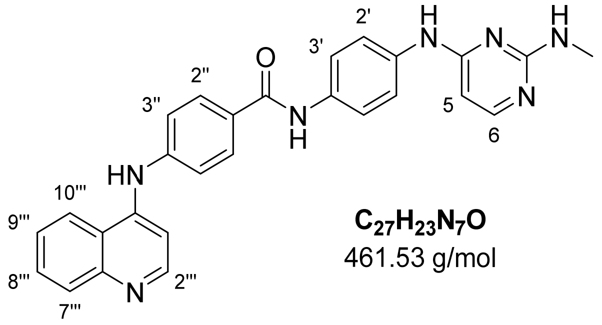

$C_{27}H_{23}N_7O$
461.53 g/mol

Following general procedure E, **90** (109 mg, 410 µmol, 1.00 eq), **96** (89.0 mg, 410 µmol, 1.00 eq), DIPEA (179 µL, 133 mg, 1.03 mmol, 2.50 eq), EDC hydrochloride (157 mg, 820 µmol, 2.00 eq), and HOBt (67.0 mg, 492 µmol, 1.20 eq) in 4 mL DMF were heated to 70°C for six hours and at room temperature overnight. The crude product was purified by silica gel column chromatography [2% MeOH → 7% MeOH/1% TEA/ DCM] and precipitated with isopropanol (2 mL) and water (15 mL) to yield **42** (100 mg, 217 µmol, 53%) as light yellow solid.

^{1}H-NMR (DMSO, 400 MHz) δ = 10.06 (s, 1H, NH), 9.23 (s, 1H, NH), 9.10 (s, 1H, NH), 8.58 (d, J = 5.2 Hz, 1H, H-2‘‘‘), 8.39 (d, J = 8.4 Hz, 1H, H-10‘‘‘), 8.01 (d, J = 8.4 Hz, 2H, H-3‘‘), 7.93 (d, J = 8.4 Hz, 1H, H-7‘‘‘), 7.83 (d, J = 5.7 Hz, 1H, H-6), 7.77 – 7.71 (m, 1H, H-9‘‘‘), 7.70 (s, 4H, H-2‘, H-3‘), 7.64 – 7.53 (m, 1H, H-8‘‘‘), 7.48 (d, J = 8.3 Hz, 2H, H-2‘‘), 7.20 (d, J = 5.2 Hz, 1H, H-3‘‘‘), 6.62 (d, J = 5.2 Hz, 1H, NH), 5.98 (d, J = 5.7 Hz, 1H, H-5), 2.80 (d, J = 4.7 Hz, 3H, CH3).

^{13}C-NMR (DMSO, 101 MHz) δ = 164.50, 162.49, 160.47, 150.66, 148.99, 146.38, 144.25, 136.39, 133.30, 129.44, 129.21, 129.05, 128.71, 124.98, 122.29, 120.82, 120.41, 119.69, 119.54, 103.63, 27.92.

HR-MS m/z calcd. for $C_{27}H_{24}N_7O^+$ $(M + 1)^+$ 462.2037, found 462.2062.

N-(4-((3-Nitrophenyl)amino)phenyl)-4-(quinolin-4-ylamino)benzamide (86)

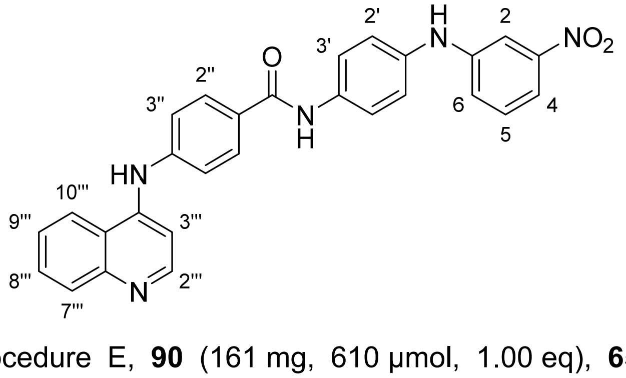

Following general procedure E, **90** (161 mg, 610 µmol, 1.00 eq), **65** (140 mg, 610 µmol, 1.00 eq), DIPEA (266 µL, 197 mg, 1.53 mmol, 2.50 eq), EDC hydrochloride (233 mg, 1.22 µmol, 2.00 eq), and HOBt (99.0 mg, 730 µmol, 1.20 eq) in 4 mL DMF were heated to 70°C for six hours and at room temperature overnight. The crude product was purified by silica gel

column chromatography [1% MeOH → 3% MeOH/1% TEA/ DCM] to yield **86** (208 mg, 439 µmol, 72%) as dark red solid.

TLC: R_f = 0.47 (10% MeOH/1% TEA/ DCM) [UV].

^{1}H-NMR (DMSO, 400 MHz) δ = 10.15 (s, 1H, NH), 9.35 (s, 1H, NH), 8.70 (s, 1H, NH), 8.58 (d, J = 5.3 Hz, 1H, H-2‘‘‘), 8.42 (d, J = 8.4 Hz, 1H, H-10‘‘‘), 8.03 (d, J = 8.7 Hz, 2H, H-3‘‘), 7.94 (d, J = 8.5 Hz, 1H, H-7‘‘‘), 7.81 – 7.70 (m, 4H, H-3‘, H-8‘‘‘,H-2*), 7.64 – 7.57 (m, 1H, H-9‘‘‘), 7.57 – 7.53 (m, 1H, H-6*), 7.50 (d, J = 8.8 Hz, 2H, H-2‘‘), 7.46 (t, J = 8.1 Hz, 1H, H-5*), 7.43 – 7.35 (m, 1H, H-4*), 7.22 – 7.14 (m, 3H, H-2‘, H-3‘‘‘).

^{13}C-NMR (DMSO, 101 MHz) δ = 164.54, 148.78, 146.09, 136.98, 134.02, 130.43, 129.60, 129.08, 128.74, 125.05, 122.41, 121.67, 120.76, 120.32, 119.87, 119.83, 112.51, 107.98, 103.53.

N-(4-((3-Methyl-5-nitrophenyl)amino)phenyl)-4-(quinolin-4-ylamino)benzamide (87)

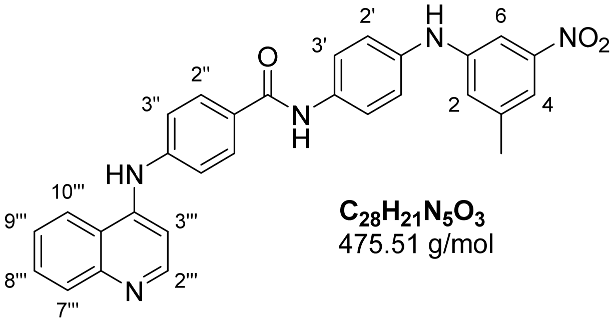

$C_{28}H_{21}N_5O_3$
475.51 g/mol

Following general procedure E, **90** (146 mg, 550 µmol, 1.00 eq), **66** (135 mg, 550 µmol, 1.00 eq), DIPEA (228 µL, 178 mg, 1.38 mmol, 2.50 eq), EDC hydrochloride (211 mg, 1.10 mmol, 2.00 eq), and HOBt (89.0 mg, 660 µmol, 1.20 eq) in 4 mL DMF were heated to 70°C for six hours and at room temperature overnight. The crude product was purified by silica gel column chromatography [1% MeOH → 3% MeOH/1% TEA/ DCM] to yield **87** (149 mg, 259 µmol, 47%) as a dark red solid.

TLC: R_f = 0.32 (5% MeOH/1% TEA/ DCM) [UV].

^{1}H-NMR (DMSO, 400 MHz) δ = 10.15 (s, 1H, NH), 8.60 (s, 1H, NH), 8.58 (d, J = 5.3 Hz, 1H, H-2‘‘‘), 8.44 (d, J = 8.5 Hz, 1H, H-10‘‘‘), 8.03 (d, J = 8.7 Hz, 2H, H-3‘‘), 7.94 (d, J = 8.5 Hz, 1H, H-7‘‘‘), 7.81 – 7.69 (m, 3H, H-3‘, H-8‘‘‘), 7.60 (t, J = 7.7 Hz, 1H, H-9‘‘‘), 7.56 (t, J = 2.2 Hz, 1H, H-6*), 7.50 (d, J = 8.7 Hz, 2H, H-2‘‘), 7.40 (s, 1H, H-4*), 7.21 – 7.15 (m, 3H, H-2‘, H-2*), 2.35 (s, 3H, CH_3).

^{13}C-NMR (DMSO, 101 MHz) δ = 164.53, 148.77, 145.91, 140.45, 137.11, 133.92, 129.09, 125.16, 122.46, 121.68, 121.07, 120.17, 120.08, 119.93, 113.17, 105.71, 103.37, 21.00.

N-(4-((2-Amino-6-methylpyrimidin-4-yl)amino)phenyl)-4-(naphthalen-1-ylamino)benzamide (44)

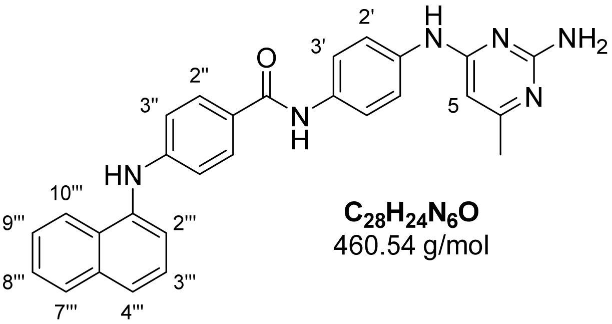

$C_{28}H_{24}N_6O$
460.54 g/mol

Following general procedure E, **70** (70.0 mg, 252 µmol, 1.00 eq), **73** (54.0 mg, 252 µmol, 1.00 eq), DIPEA (110 µL, 631 µmol, 2.50 eq), EDC hydrochloride (97.0 mg, 505 µmol, 2.00 eq), and HOBt (41.0 mg, 303 µmol, 1.20 eq) in 4 mL DMF were heated to 70°C for six hours and at room temperature overnight. The crude product was purified by silica gel column chromatography [5% MeOH/1% TEA/ DCM] to yield **44** (35.0 mg, 76.0 µmol, 30%) as light yellow solid.

^{1}H-NMR (DMSO, 600 MHz) δ = 9.84 (s, 1H, NH), 8.96 (s, 1H, NH), 8.68 (s, 1H, NH), 8.10 (d, *J* = 8.0 Hz, 1H, H-10’’’*), 7.95 (d, *J* = 9.6 Hz, 1H, H-7’’’*), 7.84 (d, *J* = 8.8 Hz, 2H, H-2’’), 7.69 (d, *J* = 8.1 Hz, 1H, H-4’’’), 7.67 – 7.60 (m, 4H, H-2’, H-3’), 7.58 – 7.48 (m, 3H, H-3’’’, H-8’’’, H-9’’’), 7.45 (d, *J* = 7.6 Hz, 1H, H-2’’’), 7.00 (d, *J* = 8.8 Hz, 2H, H-3’’), 6.16 (s, 2H, NH_2), 5.86 (s, 1H, H-5), 2.09 (s, 1H, CH3).

^{13}C-NMR (DMSO, 151 MHz) δ = 164.73, 162.59, 161.41, 148.89, 137.61, 136.14, 134.44, 133.64, 129.16, 128.28, 128.04, 128.00, 126.27, 126.14, 125.57, 124.44, 123.49, 123.47, 122.92, 120.71, 120.60, 119.81, 117.83, 114.19, 94.61, 23.29.

N-(4-((2-Amino-6-methylpyrimidin-4-yl)amino)phenyl)-4-(quinazolin-4-ylamino)benzamide (45)

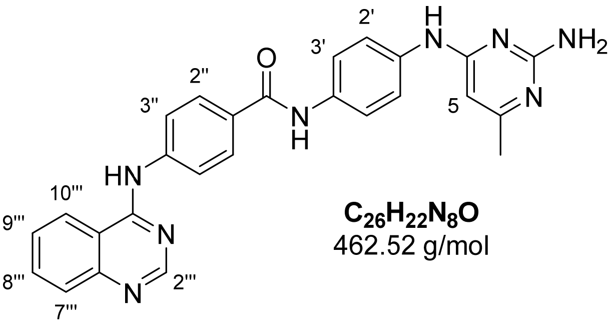

$C_{26}H_{22}N_8O$
462.52 g/mol

Following general procedure E, **92** (70.0 mg, 264 µmol, 1.00 eq), **73** (56.8 mg, 264 µmol, 1.00 eq), DIPEA (115 µL, 659 µmol, 2.50 eq), EDC hydrochloride (101 mg, 528 µmol, 2.00 eq), and HOBt (43.0 mg, 317 µmol, 1.20 eq) in 4 mL DMF were heated to 70°C for six hours and at room temperature overnight. The crude product was purified by silica gel column chromatography [5% MeOH/1% TEA/ DCM] and RP-HPLC (NUCLEODUR® C18 Pyramid,

buffer A: 50 mM ammonium acetate in water [pH 8.5], buffer B: 98% MeOH and 2% buffer A) to yield **45** (8.00 mg, 17.3 µmol, 7%) as light yellow solid.

^{1}H-NMR (DMSO, 600 MHz) δ = 10.08 (s, 1H, NH), 10.01 (s, 1H, NH), 8.95 (s, 1H, NH), 8.69 (s, 1H, H-2'''), 8.62 (dd, *J* = 8.6, 1.4 Hz, 1H, H-7'''), 8.09 (d, *J* = 8.8 Hz, 1H, H-2''), 8.01 (d, *J* = 8.8 Hz, 1H, H-3''), 7.91 (t, *J* = 7.0 Hz, 1H, H-9'''), 7.84 (d, *J* = 8.4 Hz, 1H, H-10'''), 7.73 – 7.58 (m, 5H, H-8''', H-2', H-2'), 6.13 (s, 2H, NH_2), 5.86 (s, 1H, H-5), 2.08 (s, 1H, CH_3).

^{13}C-NMR (DMSO, 151 MHz) δ = 164.77, 164.64, 162.83, 161.40, 157.58, 154.33, 149.81, 142.22, 136.59, 133.27, 133.22, 129.59, 128.15, 127.94, 126.52, 123.06, 121.09, 120.81, 119.69, 115.28, 94.60, 23.45.

HR-MS m/z calcd. for $C_{26}H_{23}N_8O^+$ $(M + 1)^+$ 463.1989, found 463.2008.

***N*-(4-((2-Amino-6-methylpyrimidin-4-yl)amino)phenyl)-4-((3a,7a-dihydro-1H-pyrrolo[2,3-b]pyridin-4-yl)amino)benzamide (46)**

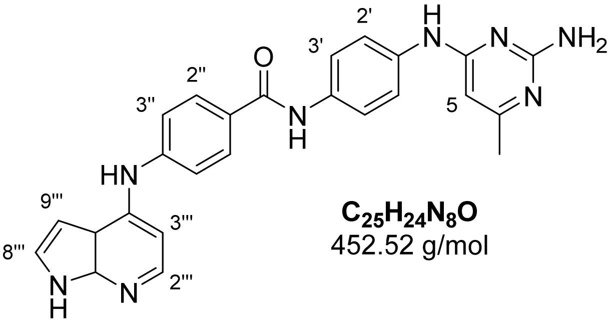

$C_{25}H_{24}N_8O$
452.52 g/mol

Following general procedure E, **93** (70.0 mg, 267 µmol, 1.00 eq), **73** (59.4 mg, 267 µmol, 1.00 eq), DIPEA (117 µL, 690 µmol, 2.50 eq), EDC hydrochloride (106 mg, 550 µmol, 2.00 eq), and HOBt (44.8 mg, 332 µmol, 1.20 eq) in 5 mL DMF were heated to 70°C for six hours and at room temperature overnight. The crude product was purified by silica gel column chromatography [5% MeOH/1% TEA/ DCM] to yield **46** (58.1 mg, 129 µmol, 47%) as light yellow solid.

^{1}H-NMR (DMSO, 400 MHz) δ = 11.44 (s, 1H, NH), 9.98 (s, 1H, NH), 8.96 (s, 1H, NH), 8.90 (s, 1H, NH), 8.00 (d, *J* = 5.4 Hz, 1H, H-2'''), 7.96 (d, *J* = 8.7 Hz, 2H, H-2''), 7.66 (d, *J* = 2.9 Hz, 4H, H-2', H-3'), 7.37 (d, *J* = 8.7 Hz, 2H, H-3''), 7.27 (dd, *J* = 3.4, 2.2 Hz, 1H, H-8'''), 6.86 (d, *J* = 5.4 Hz, 1H, H-3'''), 6.60 (dd, *J* = 3.5, 1.7 Hz, 1H, H-9'''), 6.09 (s, 2H, NH_2), 5.86 (s, 1H, H-5), 2.08 (s, 3H, CH_3).

^{13}C-NMR (DMSO, 101 MHz) δ = 164.90, 164.56, 162.91, 161.40, 149.95, 144.72, 143.80, 142.00, 136.46, 133.32, 128.93, 127.29, 122.77, 120.78, 119.70, 117.97, 109.69, 100.38, 98.31, 94.54, 23.49.

General procedure F: Boc-deprotection

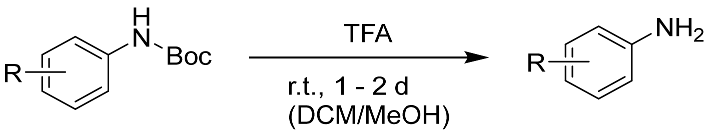

To a solution of boc-protected compound (1.00 eq) in 4 mL DCM and 1 mL methanol was added 2 mL trifluoroacetic acid. The reaction mixture was stirred at room temperature for two days. Completion of deprotection was monitored by TLC. The solvent was removed under reduced pressure and the residue was dissolved in EtOAc and sat. $NaHCO_3$ solution. The aqueous phase was alkalized with 10% aqueous NaOH solution and extracted with EtOAc (3 x 50 mL). The combined organic layer was dried over $MgSO_4$, filtered and concentrated. Each residue was purified by silica gel column chromatography and final compounds were further purified by precipitation as stated above.

_N_-(4-((3-Amino-5-fluorophenyl)amino)phenyl)-4-(quinolin-4-ylamino)benzamide (38)

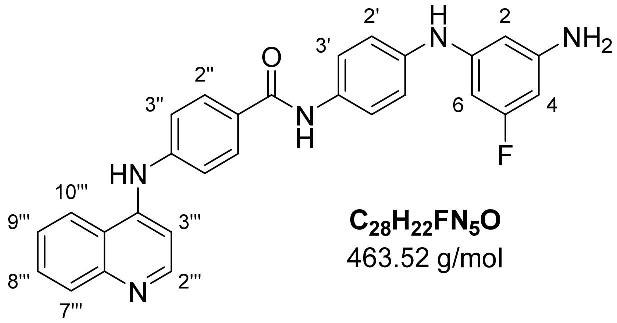

$C_{28}H_{22}FN_5O$
463.52 g/mol

Following general procedure F, **95** (64.0 mg, 96.4 µmol, 1.00 eq) and 2 mL TFA in DCM /MeOH (4 mL/1 mL) were stirred at room temperature for two days. The crude product was purified by silica gel column chromatography [3% MeOH/1% TEA/ DCM] and precipitated with isopropanol (2 mL) and water (15 mL) to yield deprotected **38** (29.0 mg, 55.0 µmol, 57%) as light yellow solid.

TLC: R_f = 0.31 (5% MeOH/1% TEA/ DCM) [UV].

^{1}H-NMR (MeOD, 400 MHz) δ = 8.49 (d, J = 5.5 Hz, 1H, H-2‘‘‘), 8.30 (d, J = 8.4 Hz, 1H, H-10‘‘‘), 7.99 (d, J = 8.6 Hz 2H, H-3‘‘), 7.93 (d, J = 8.5, 1H, H-7‘‘‘), 7.74 (t, J = 8.0, 1H, H-8‘‘‘), 7.61 – 7.51 (m, 3H, H-9‘‘‘, H-3‘), 7.49 (d, J = 7.6 Hz, 2H, H-2‘‘), 7.23 (d, J = 5.5 Hz, 1H, H-3‘‘‘), 7.11 (d, J = 8.8 Hz, 2H, H-2‘), 6.24 (t, J = 1.9 Hz, 1H, H-2*), 6.09 (dt, J = 11.4, 2.1 Hz, 1H, H-6*), 5.92 (dt, J = 10.9, 2.1 Hz, 1H, H-4*).

^{13}C-NMR (MeOD, 101 MHz) δ = 167.97, 167.33, 164.97, 151.42, 151.29, 149.78, 149.48, 147.91, 147.78, 145.63, 141.42, 132.96, 131.10, 130.77, 130.16, 129.18, 126.70, 123.75, 122.84, 121.90, 121.82, 120.01, 104.37, 99.46, 94.89, 94.64, 94.10, 93.85.

HR-MS m/z calcd. for $C_{28}H_{23}FN_5O^+$ $(M + 1)^+$ 464.1881, found 464.1914.

N-(4-((3-Amino-4-fluorophenyl)amino)phenyl)-4-(quinolin-4-ylamino)benzamide (37)

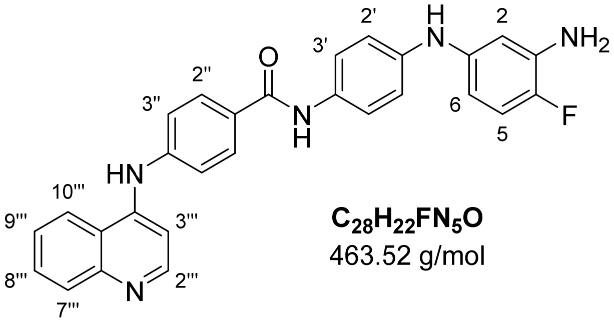

$C_{28}H_{22}FN_5O$
463.52 g/mol

Following general procedure F, 2 mL TFA was slowly added to a solution of **94** (82.0 mg, 123 µmol, 1.00 eq) in DCM/MeOH (4 mL/1 mL) and the reaction mixture was stirred at room temperature for two days. The reaction mixture was quenched with aqueous $NaHCO_3$ solution and extracted with EtOAc (3 x 50 mL). Purification by silica gel column chromatography [2% MeOH/1% TEA/ DCM] and precipitated with isopropanol (2 mL) and water (15 mL) afforded **37** (37 mg, 80.0 µmol, 65%) as yellowish solid.

TLC: R_f = 0.29 (5% MeOH/1% TEA/ DCM) [UV].

^{1}H-NMR (MeOD, 400 MHz) δ = 8.50 (d, J = 5.5 Hz, 1H, H-2‘‘‘), 8.33 (dd, J = 8.6, 1.3 Hz, 1H, H-10‘‘‘), 8.00 (d, J = 8.7 Hz, 2H, H-3‘‘), 7.94 (d, J = 9.0 Hz, 1H, H-7‘‘‘), 7.77 (ddd, J = 8.4, 6.8, 1.4 Hz, 1H, H-8‘‘‘), 7.59 (ddd, J = 8.3, 6.9, 1.3 Hz, 1H, H-9‘‘‘), 7.50 (dd, J = 8.8, 3.8 Hz, 4H, H-3‘, H-2‘‘), 7.24 (d, J = 5.5 Hz, 1H, H-3‘‘‘), 7.03 (d, J = 8.8 Hz, 2H, H-2‘), 6.83 (dd, J = 11.0, 8.7 Hz, 1H, H-5), 6.63 (dd, J = 7.9, 2.7 Hz, 1H, H-2), 6.37 (ddd, J = 8.7, 3.8, 2.7 Hz, 1H, H-6).

^{13}C-NMR (MeOD, 126 MHz) δ = 151.19, 149.68, 143.20, 141.76, 131.20, 130.97, 130.16, 129.07, 126.76, 123.96, 122.88, 121.97, 118.05, 116.20, 116.04, 108.93, 108.07, 104.33.

^{19}F-NMR (MeOD, 471 MHz, DMSO) δ = -146.58.

HR-MS m/z calcd. for $C_{28}H_{23}FN_5O^+$ $(M + 1)^+$ 464.1881, found 464.1919.

N^4-(4-Aminophenyl)-N^2-methylpyrimidine-2,4-diamine (96)

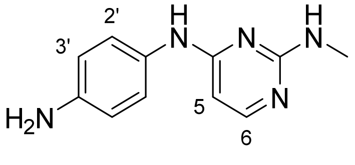

$C_{11}H_{13}N_5$
215.26 g/mol

Following general procedure F, 2 mL TFA was slowly added to a solution of **69** (588 mg, 1.86 mmol, 1.00 eq) in DCM/MeOH (4 mL/1 mL) and the reaction mixture was stirred at room temperature for two days. The reaction mixture was quenched with aqueous $NaHCO_3$ solution and extracted with EtOAc (3 x 50 mL). Purification by silica gel column chromatography [2% MeOH/1% TEA/ DCM] afforded **96** (94 mg, 428 µmol, 23%) as yellowish solid.

TLC: R_f = 0.35 (10% MeOH/1% TEA/ DCM) [UV].

^{1}H-NMR (DMSO, 400 MHz) δ = 8.60 (s, 1H, NH), 7.71 (d, J = 5.7 Hz, 1H, H-6), 7.22 (d, J = 8.2 Hz, 2H, H-2‘), 6.51 (d, J = 8.7 Hz, 2H, H-3‘), 6.42 (s, 1H, NH), 5.81 (d, J = 5.8 Hz, 1H, H-5), 4.89 (s, 2H, NH_2), 2.74 (d, J = 4.7 Hz, 3H, CH_3).
^{13}C-NMR (DMSO, 101 MHz) δ = 162.40, 161.01, 155.19, 144.22, 129.15, 122.34, 114.00, 27.86.

Synthesis of truncated SGI-1027 structures

***N*-(4-Aminophenyl)-4-(quinolin-4-ylamino)benzamide (22)**

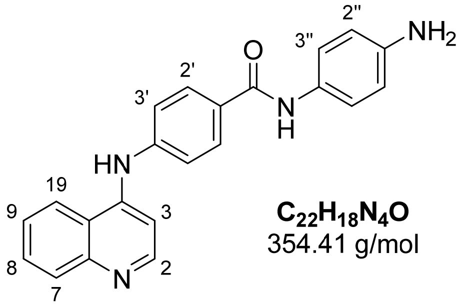

$C_{22}H_{18}N_4O$
354.41 g/mol

Following general procedure E, benzene-1,4-diamine (20.0 mg, 189 µmol, 1.00 eq), 4-(quinolin-4-ylamino)benzoic acid (**90**) (49.0 mg, 189 µmol, 1.00 eq), DIPEA (80.0 µL, 61.0 mg, 473 µmol, 2.50 eq), EDC hydrochloride (43.0 mg, 227 µmol, 1.20 eq) and HOBt (30.0 mg, 227 µmol, 1.20 eq) in 2 mL DMF were stirred at 70 °C for two hours. The crude product was purified by silica gel column chromatography [1% MeOH/1% TEA/ DCM → 10% MeOH/1% TEA / DCM]. Recrystallization in 50 mL isopropanol afforded the final compound **22** as a light yellow crystals (10.0 mg, 28.4 µmol, 15%).
TLC: R_f = 0.46 (5% MeOH/1%TEA/ DCM) [UV].
^{1}H-NMR (MeOD, 400 MHz) δ = 8.50 (d, *J* = 5.55 Hz, 1H, H-2), 8.34 (d, *J* = 8.40 Hz, 1H, H-10), 8.00 (d, *J* = 8.64 Hz, 2H, H-3‘), 7.94 (d, *J* = 8.0 Hz, 1H, H-7), 7.84 – 7.74 (m, 1H, H-8), 7.66 – 7.56 (m, 1H, H-9), 7.51 (d, *J* = 8.64 Hz, 2H, H-2‘), 7.39 (d, *J* = 8.69 Hz, 2H, H-3“), 7.23 (d, *J* = 5.55 Hz, 1H, H-3), 6.76 (d, *J* = 8.69 Hz, 2H, H-2“).
HR-MS m/z calcd. for $C_{22}H_{19}N_5O^+$ $(M + 1)^+$ 355.1559, found 355.1560.
These data are in accordance with previous literature reports. Literature spectra were recorded in deuterated DMSO.[4]

***N*-(4-((2-Amino-6-methylpyrimidin-4-yl)amino)phenyl)-4-nitrobenzamide (24)**

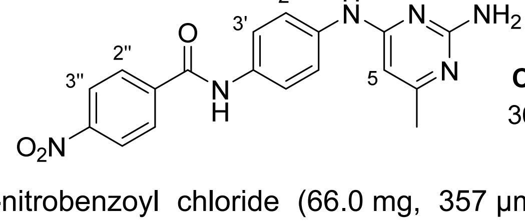

$C_{18}H_{16}N_6O_3$
364.37 g/mol

To a solution of 4-nitrobenzoyl chloride (66.0 mg, 357 µmol, 1.00 eq) and **73** (100 mg, 465 µmol, 1.30 eq) in 10 mL anhydrous DMF was added Cs_2CO_3 (348 mg, 1.07 mmol,

3.00 eq). The reaction mixture was stirred at room temperature for 4 hours. The solvent was removed under reduced pressure and the crude product was purified by silica gel column chromatography [1% MeOH/1% TEA/ DCM → 10% MeOH/1% TEA / DCM] and RP-HPLC (NUCLEODUR® C18, 50 mM ammonium acetate in water/ MeCN) to afford **24** (9.00 mg, 25.0 µmol, 7%) as orange solid.

^{1}H-NMR (DMSO, 500 MHz) δ = 10.46 (s,1H, NH), 8.98 (s, 1H, NH), 8.36 (d, J = 8.70 Hz, 2H, H-3”), 8.17 (d, J = 8.70 Hz, 2H, H-2“), 7.68 (dd, J = 9.05 Hz, 4H, H-2‘, H-3‘), 6.10 (s, 2H, NH_2), 5.86 (s, 1H, H-5), 2.08 (s, 3H, CH_3).

HR-MS m/z calcd. for $C_{18}H_{17}N_6O_3^+$ $(M + 1)^+$ 365.1362, found 365.1364.

These data are in accordance with previous literature reports.[3]

N-(4-((2-Amino-6-methylpyrimidin-4-yl)amino)phenyl)benzamide (23)

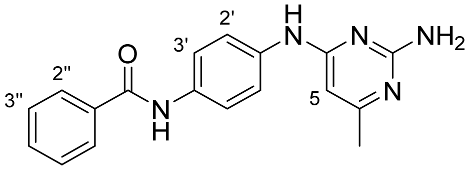

$C_{18}H_{17}N_5O$
319.37 g/mol

To a solution of *N*[4]-(4-aminophenyl)-6-methylpyrimidine-2,4-diamine (**73**) (100 mg, 465 µmol, 1.10 eq) in 10 mL anhydrous THF were added TEA (147 µL, 107 mg, 1.06 mmol, 2.50 eq) and benzoyl chloride (50.0 µL, 60.0 mg, 423 µmol, 1.00 eq). The reaction mixture was stirred at 80 °C for 2 hours and at room temperature overnight. The solvent was removed under reduced pressure and the crude product was purified by silica gel column chromatography [1% MeOH/1% TEA/ DCM → 5% MeOH/1% TEA/ DCM]. **23** (69.3 mg, 216 µmol, 51%) was obtained after further purification by extraction with EtOAc (3 x 50 mL) and sat. $NaHCO_3$ solution as light brown solid.

TLC: R_f = 0.33 (10% MeOH/1%TEA/ DCM) [UV].

^{1}H-NMR (DMSO, 400 MHz) δ = 10.14 (s, 1H, NH), 8.93 (s, 1H, NH), 7.94 (d, J = 6.7 Hz, 2H, H-2”), 7.66 (s, 4H, H-2’, H-3’), 7.60 – 7.41 (m, 3H, H-3”, H-4”), 6.11 (s, 2H, NH_2), 5.85 (s, 1H, H-5), 2.08 (s, 3H, CH_3).

^{13}C-NMR (DMSO, 101 MHz) δ = 165.14, 164.91, 162.90, 161.39, 136.75, 135.13, 133.02, 131.38, 128.35, 127.57, 120.85, 119.64, 94.59, 23.50.

HR-MS m/z calcd. for $C_{18}H_{18}N_5O^+$ $(M + 1)^+$ 320.1506, found 320.1512.

4-((4-((2-Amino-6-methylpyrimidin-4-yl)amino)phenyl)carbamoyl)benzenaminium (25)

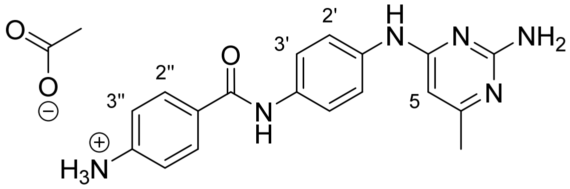

$C_{20}H_{22}N_6O_3$
394.44 g/mol

To a solution of **73** (100 mg, 465 µmol, 1.10 eq) in 10 mL anhydrous THF were added TEA (147 µL, 107 mg, 1.06 mmol, 2.50 eq) and 4-nitrobenzoyl chloride (78.0 mg, 423 µmol, 1.00 eq). The reaction mixture was stirred at 80 °C for 5 hours. The solvent was removed under reduced pressure and the residue was extracted with EtOAc (3 x 100 mL) and sat. $NaHCO_3$-solution. The combined organic layer was dried over $MgSO_4$, filtered and concentrated. In the second step, the 4-nitrobenzamide intermediate, 10 wt% Pd/C (45.0 mg, 423 µmol, 1.00 eq) and ammonium formate (243 mg, 3.85 mmol, 11.0 eq) were suspended in 10 mL EtOH and stirred at room temperature overnight. The suspension was filtered over Celite and washed with EtOAc. The filtrate was concentrated and purified via RP-HPLC (NUCLEODUR® C18, 50 mM ammonium acetate in water/ MeCN) to obtain **25** (24.0 mg, 59.2 µmol, 14%) as light yellow acetate salt.

^{1}H-NMR (DMSO, 400 MHz) δ = 9.65 (s, 1H, NH), 8.87 (s, 1H, NH), 7.70 (d, *J* = 8.7 Hz, 2H, H-2''), 7.67 – 7.49 (m, 4H, H-2', H-3'), 6.59 (d, *J* = 8.7 Hz, 2H, H-3''), 6.09 (s, 2H, NH_2), 5.84 (s, 1H, H-5), 5.71 (s, 2H, NH_2), 2.07 (s, 3H, CH_3), 1.90 (s, 3H, CH_3COO^-).

^{13}C-NMR (DMSO, 101 MHz) δ = 172.08, 164.97, 162.88, 161.44, 151.96, 136.02, 133.78, 129.21, 121.30, 120.62, 119.76, 112.54, 94.49, 23.46, 21.12.

HR-MS m/z calcd. for $C_{18}H_{19}N_6O^+$ $(M + 1)^+$ 335.1615, found 335.1615.

These data are in accordance with previous literature reports.[3]

4 Supplementary references

[1] Y. Yuan, F. M. Stumpf, L. A. Schlor, O. P. Schmidt, P. Saumer, L. B. Huber, M. Frese, E. Höllmüller, M. Scheffner, F. Stengel, K. Diederichs, A. Marx, *Nat. Commun.* **2023**, *14*.

[2] J. M. Pascal, P. J. O'Brien, A. E. Tomkinson, T. Ellenberger, *Nature* **2004**, *432*, 473-478.

[3] P. García-Domínguez, C. Dell'aversana, R. Alvarez, L. Altucci, A. R. Lera, *Bioorganic Med. Chem. Lett.* **2013**, *23*, 1631-1635.

[4] S. Valente, Y. Liu, M. Schnekenburger, C. Zwergel, S. Cosconati, C. Gros, M. Tardugno, D. Labella, C. Florean, S. Minden, H. Hashimoto, Y. Chang, X. Zhang, G. Kirsch, E. Novellino, P. B. Arimondo, E. Miele, E. Ferretti, A. Gulino, M. Diederich, X. Cheng, A. Mai, *J. Med. Chem.* **2014**, *57*, 701-713.

[5] M. Huang, Z. Yi, Y. Wan, X. Zhu, *Synthesis* **2018**, *50*, 3911-3920.

[6] R. Di Santo, R. Costi, G. Cuzzucoli Crucitti, L. Pescatori, F. Rosi, L. Scipione, D. Celona, M. Vertechy, O. Ghirardi, P. Piovesan, M. Marzi, S. Caccia, G. Guiso, F. Giorgi, P. Minetti, *J. Med. Chem.* **2012**, *55*, 8538-8548.

5 Appendix

Amino acid sequence of expressed proteins

<u>Expressed amino acid sequence from pET15b-Rlig1:</u>

MGSSHHHHHHSSGLVPR↓GSHMKRLGSVQRKMPCVFVTEVKEEPSSKREHQPFKVLATETVSHKALDADIYSAIP
TEKVDGTCCYVTTYKDQPYLWARLDRKPNKQAEKRFKNFLHSKENPKEFFWNVEEDFKPAPECWIPAKETEQING
NPVPDENGHIPGWVPVEKNNKQYCWHSSVVNYEFEIALVLKHHPDDSGLLEISAVPLSDLLEQTLELIGTNINGNPY
GLGSKKHPLHLLIPHGAFQIRNLPSLKHNDLVSWFEDCKEGKIEGIVWHCSDGCLIKVHRHHLGLCWPIPDTYM
NSRPVIINMNLNKCDSAFDIKCLFNHFLKIDNQKFVRLKDIIFDV*

<u>Expressed amino acid sequence from pET28a-hLig1:</u>

MQLSHHHHHHSSGENLYFQ↓GHMMQRSIMSFFHPKKEGKAKKPEKEASNSSRETEPPPKAALKEWNGVVSESD
SPVKRPGRKAARVLGSEGEEEDEALSPAKGQKPALDCSQVSPPRPATSPENNASLSDTSPMDSSPSGIPKRRTAR
KQLPKRTIQEVLEEQSEDEDREAKRKKEEEEEETPKESLTEAEVATEKEGEDGDQPTTPPKPLKTSKAETPTESVS
EPEVATKQELQEEEEQTKPPRRAPKTLSSFFTPRKPAVKKEVKEEEPGAPGKEGAAEGPLDPSGYNPAKNNYHPV
EDACWKPGQKVPYLAVARTFEKIEEVSARLRMVETLSNLLRSVVALSPPDLLPVLYLSLNHLGPPQQGLELGVGDG
VLLKAVAQATGRQLESVRAEAAEKGDVGLVAENSRSTQRLMLPPPPLTASGVFSKFRDIARLTGSASTAKKIDIIKGL
FVACRHSEARFIARSLSGRLRLGLAEQSVLAALSQAVSLTPPGQEFPPAMVDAGKGKTAEARKTWLEEQGMILKQ
TFCEVPDLDRIIPVLLEHGLERLPEHCKLSPGIPLKPMLAHPTRGISEVLKRFEEAAFTCEYKYDGQRAQIHALEGG
EVKIFSRNQEDNTGKYPDIISRIPKIKLPSVTSFILDTEAVAWDREKKQIQPFQVLTTRKRKEVDASEIQVQVCLYAFD
LIYLNGESLVREPLSRRRQLLRENFVETEGEFVFATSLDTKDIEQIAEFLEQSVKDSCEGLMVKTLDVDATYEIAKRS
HNWLKLKKDYLDGVGDTLDLVVIGAYLGRGKRAGRYGGFLLASYDEDSEELQAICKLGTGFSDEELEEHHQSLKA
LVLPSPRPYVRIDGAVIPDHWLDPSAVWEVKCADLSLSPIYPAARGLVDSDKGISLRFPRFIRVREDKQPEQATTSA
QVACLYRKQSQIQNQQGEDSGSDPEDTY*

Sequence of plasmids used in this study

<u>pET28a-hLig1</u>

TGGCGAATGGGACGCGCCCTGTAGCGGCGCATTAAGCGCGGCGGGTGTGGTGGTTACGCGCAGCGTGACC
GCTACACTTGCCAGCGCCCTAGCGCCCGCTCCTTTCGCTTTCTTCCCTTCCTTTCTCGCCACGTTCGCCGGC
TTTCCCCGTCAAGCTCTAAATCGGGGGCTCCCTTTAGGGTTCCGATTTAGTGCTTTACGGCACCTCGACCCCA
AAAAACTTGATTAGGGTGATGGTTCACGTAGTGGGCCATCGCCCTGATAGACGGTTTTTCGCCCTTTGACGTT
GGAGTCCACGTTCTTTAATAGTGGACTCTTGTTCCAAACTGGAACAACACTCAACCCTATCTCGGTCTATTCTT
TTGATTTATAAGGGATTTTGCCGATTTCGGCCTATTGGTTAAAAAATGAGCTGATTTAACAAAAATTTAACGCGAA
TTTTAACAAAATATTAACGCTTACAATTTAGGTGGCACTTTTCGGGGAAATGTGCGCGGAACCCCTATTTGTTTA
TTTTTCTAAATACATTCAAATATGTATCCGCTCATGAATTAATTCTTAGAAAAACTCATCGAGCATCAAATGAAACT
GCAATTTATTCATATCAGGATTATCAATACCATATTTTTGAAAAAGCCGTTTCTGTAATGAAGGAGAAAACTCACC

GAGGCAGTTCCATAGGATGGCAAGATCCTGGTATCGGTCTGCGATTCCGACTCGTCCAACATCAATACAACCT
ATTAATTTCCCCTCGTCAAAAATAAGGTTATCAAGTGAGAAATCACCATGAGTGACGACTGAATCCGGTGAGAA
TGGCAAAAGTTTATGCATTTCTTTCCAGACTTGCTCAACAGGCCAGCCATTACGCTCGTCATCAAAATCACTCG
CATCAACCAAACCGTTATTCATTCGTGATTGCGCCTGAGCGAGACGAAATACGCGATCGCTGTTAAAAGGACA
ATTACAAACAGGAATCGAATGCAACCGGCGCAGGAACACTGCCAGCGCATCAACAATATTTTCACCTGAATCA
GGATATTCTTCTAATACCTGGAATGCTGTTTTCCCGGGGATCGCAGTGGTGAGTAACCATGCATCATCAGGAGT
ACGGATAAAATGCTTGATGGTCGGAAGAGGCATAAATTCCGTCAGCCAGTTTAGTCTGACCATCTCATCTGTAA
CATCATTGGCAACGCTACCTTTGCCATGTTTCAGAAACAACTCTGGCGCATCGGGCTTCCCATACAATCGATAG
ATTGTCGCACCTGATTGCCCGACATTATCGCGAGCCCATTTATACCCATATAAATCAGCATCCATGTTGGAATTT
AATCGCGGCCTAGAGCAAGACGTTTCCCGTTGAATATGGCTCATAACACCCCTTGTATTACTGTTTATGTAAGC
AGACAGTTTTATTGTTCATGACCAAAATCCCTTAACGTGAGTTTTCGTTCCACTGAGCGTCAGACCCCGTAGAA
AAGATCAAAGGATCTTCTTGAGATCCTTTTTTTCTGCGCGTAATCTGCTGCTTGCAAACAAAAAAACCACCGCT
ACCAGCGGTGGTTTGTTTGCCGGATCAAGAGCTACCAACTCTTTTTCCGAAGGTAACTGGCTTCAGCAGAGC
GCAGATACCAAATACTGTCCTTCTAGTGTAGCCGTAGTTAGGCCACCACTTCAAGAACTCTGTAGCACCGCCTA
CATACCTCGCTCTGCTAATCCTGTTACCAGTGGCTGCTGCCAGTGGCGATAAGTCGTGTCTTACCGGGTTGGA
CTCAAGACGATAGTTACCGGATAAGGCGCAGCGGTCGGGCTGAACGGGGGGTTCGTGCACACAGCCCAGCT
TGGAGCGAACGACCTACACCGAACTGAGATACCTACAGCGTGAGCTATTAGAAAGCGCCACGCTTCCCGAAG
GGAGAAAGGCGGACAGGTATCCGGTAAGCGGCAGGGTCGGAACAGGAGAGCGCACGAGGGAGCTTCCAGG
GGGAAACGCCTGGTATCTTTATAGTCCTGTCGGGTTTCGCCACCTCTGACTTGAGCGTCGATTTTTGTGATGC
TCGTCAGGGGGGCGGAGCCTATGGAAAAACGCCAGCAACGCGGCCTTTTTACGGTTCCTGGCCTTTTGCTG
GCCTTTTGCTCACATGTTCTTTCCTGCGTTATCCCCTGATTCTGTGGATAACCGTATTACCGCCTTTGAGTGAG
CTGATACCGCTCGCCGCAGCCGAACGACCGAGCGCAGCGAGTCAGTGAGCGAGGAAGCGGAAGAGCGCCT
GATGCGGTATTTTCTCCTTACGCATCTGTGCGGTATTTCACACCGCAATGGTGCACTCTCAGTACAATCTGCTC
TGATGCCGCATAGTTAAGCCAGTATACACTCCGCTATCGCTACGTGACTGGGTCATGGCTGCGCCCCGACACC
CGCCAACACCCGCTGACGCGCCCTGACGGGCTTGTCTGCTCCCGGCATCCGCTTACAGACAAGCTGTGACC
GTCTCCGGGAGCTGCATGTGTCAGAGGTTTTCACCGTCATCACCGAAACGCGCGAGGCAGCTGCGGTAAAG
CTCATCAGCGTGGTCGTGAAGCGATTCACAGATGTCTGCCTGTTCATCCGCGTCCAGCTCGTTGAGTTTCTCC
AGAAGCGTTAATGTCTGGCTTCTGATAAAGCGGGCCATGTTAAGGGCGGTTTTTTCCTGTTTGGTCACTGATG
CCTCCGTGTAAGGGGGATTTCTGTTCATGGGGGTAATGATACCGATGAAACGAGAGAGGATGCTCACGATACG
GGTTACTGATGATGAACATGCCCGGTTACTGGAACGTTGTGAGGGTAAACAACTGGCGGTATGGATGCGGCG
GGACCAGAGAAAAATCACTCAGGGTCAATGCCAGCGCTTCGTTAATACAGATGTAGGTGTTCCACAGGGTAGC
CAGCAGCATCCTGCGATGCAGATCCGGAACATAATGGTGCAGGGCGCTGACTTCCGCGTTTCCAGACTTTAC
GAAACACGGAAACCGAAGACCATTCATGTTGTTGCTCAGGTCGCAGACGTTTTGCAGCAGCAGTCGCTTCAC
GTTCGCTCGCGTATCGGTGATTCATTCTGCTAACCAGTAAGGCAACCCCGCCAGCCTAGCCGGGTCCTCAAC
GACAGGAGCACGATCATGCGCACCCGTGGGGCCGCCATGCCGGCGATAATGGCCTGCTTCTCGCCGAAACG
TTTGGTGGCGGGACCAGTGACGAAGGCTTGAGCGAGGGCGTGCAAGATTCCGAATACCGCAAGCGACAGGC
CGATCATCGTCGCGCTCCAGCGAAAGCGGTCCTCGCCGAAAATGACCCAGAGCGCTGCCGGCACCTGTCCT
ACGAGTTGCATGATAAAGAAGACAGTCATAAGTGCGGCGACGATAGTCATGCCCCGCGCCCACCGGAAGGAG
CTGACTGGGTTGAAGGCTCTCAAGGGCATCGGTCGAGATCCCGGTGCCTAATGAGTGAGCTAACTTACATTAA
TTGCGTTGCGCTCACTGCCCGCTTTCCAGTCGGGAAACCTGTCGTGCCAGCTGCATTAATGAATCGGCCAAC
GCGCGGGGAGAGGCGGTTTGCGTATTGGGCGCCAGGGTGGTTTTTCTTTTCACCAGTGAGACGGGCAACAG
CTGATTGCCCTTCACCGCCTGGCCCTGAGAGAGTTGCAGCAAGCGGTCCACGCTGGTTTGCCCCAGCAGGC
GAAAATCCTGTTTGATGGTGGTTAACGGCGGGATATAACATGAGCTGTCTTCGGTATCGTCGTATCCCACTACC
GAGATATCCGCACCAACGCGCAGCCCGGACTCGGTAATGGCGCGCATTGCGCCCAGCGCCATCTGATCGTT
GGCAACCAGCATCGCAGTGGGAACGATGCCCTAATTCAGCATTTGCATGGTTTGTTGAAAACCGGACATGGC
ACTCCAGTCGCCTTCCCGTTCCGCTATCGGCTGAATTTGATTGCGAGTGAGATATTTATGCCAGCCAGCCAGA
CGCAGACGCGCCGAGACAGAACTTAATGGGCCCGCTAACAGCGCGATTTGCTGGTGACCCAATGCGACCAG
ATGCTCCACGCCCAGTCGCGTACCGTCTTCATGGGAGAAAATAATACTGTTGATGGGTGTCTGGTCAGAGACA
TCAAGAAATAACGCCGGAACATTAGTGCAGGCAGCTTCCACAGCAATGGCATCCTGGTCATCCAGCGGATAGT
TAATGATCAGCCCACTGACGCGTTGCGCGAGAAGATTGTGCACCGCCGCTTTACAGGCTTCGACGCCGCTTC
GTTCTACCATCGACACCACCACGCTGGCACCCAGTTGATCGGCGCGAGATTTAATCGCCGCGACAATTTGCG
ACGGCGCGTGCAGGGCCAGACTGGAGGTGGCAACGCCAATCAGCAACGACTGTTTGCCCGCCAGTTGTTGT
GCCACGCGGTTGGGAATGTAATTCAGCTCCGCCATCGCCGCTTCCACTTTTTCCCGCGTTTTCGCAGAAACG
TGGCTGGCCTGGTTCACCACGCGGGAAACGGTCTGATAAGAGACACCGGCATACTCTGCGACATCGTATAAC
GTTACTGGTTTCACATTCACCACCCTGAATTGACTCTCTTCCGGGCGCTATCATGCCATACCGCGAAAGGTTTT
GCGCCATTCGATGGTGTCCGGGATCTCGACGCTCTCCCTTATGCGACTCCTGCATTAGGAAGCAGCCCAGTA
GTAGGTTGAGGCCGTTGAGCACCGCCGCCGCAAGGAATGGTGCATGCAAGGAGATGGCGCCCAACAGTCCC
CCGGCCACGGGGCCTGCCACCATACCCACGCCGAAACAAGCGCTCATGAGCCCGAAGTGGCGAGCCCGAT
CTTCCCCATCGGTGATGTCGGCGATATAGGCGCCAGCAACCGCACCTGTGGCGCCGGTGATGCCGGCCACG

ATGCGTCCGGCGTAGAGGATCGAGATCTCGATCCCGCGAAATTAATACGACTCACTATAGGGAGAGGAATTGT
GAGCGGATAACAATTCCCCTCTAGAAATAATTTTGTTTAACTTTAAGAAGGAGAGCAGCTATGCAGCTTAGCCAT
CATCATCATCATCACAGCAGCGGCGAAAACCTGTATTTTCAGGGCCATATGATGCAACGCTCGATTATGAGCTT
CTTTCACCCTAAAAAAGAGGGGAAAGCTAAAAAGCCAGAGAAGGAAGCATCTAACTCTTCACGTGAGACTGA
GCCGCCTCCCAAGGCTGCGTTGAAAGAGTGGAATGGCGTTGTAAGTGAAAGTGACTCTCCCGTCAAACGCC
CCGGGCGCAAGGCTGCACGTGTATTAGGTTCAGAAGGAGAGGAGGAAGACGAAGCCTTGTCGCCTGCTAAA
GGGCAAAAGCCCGCGCTTGACTGCTCTCAAGTGTCCCCTCCTCGTCCTGCGACTAGCCCAGAGAATAATGCA
AGTCTGTCCGATACCAGTCCTATGGATTCGTCCCCTAGCGGGATCCCAAAGCGCCGTACTGCACGTAAGCAG
CTTCCCAAACGTACCATTCAGGAAGTCCTTGAAGAGCAATCGGAAGATGAGGATCGTGAAGCAAAACGCAAA
AAGGAGGAGGAGGAAGAAGAAACTCCGAAGGAGAGCCTGACTGAAGCTGAGGTCGCAACCGAGAAGGAGG
GTGAGGATGGCGATCAGCCAACAACGCCACCGAAGCCACTGAAGACCTCAAAGGCAGAGACGCCAACCGAG
AGCGTGAGCGAACCCGAGGTTGCTACAAAGCAAGAGTTGCAGGAGGAGGAAGAGCAGACTAAACCCCCACG
TCGCGCACCGAAAACATTATCGTCCTTCTTTACCCCGCGTAAACCCGCAGTGAAAAAGGAGGTCAAGGAAGA
AGAGCCGGGCGCACCAGGTAAGGAAGGTGCAGCGGAAGGACCTCTTGATCCAAGTGGGTATAACCCCGCAA
AAAATAACTATCACCCCGTAGAGGATGCCTGTTGGAAGCCGGGGCAGAAAGTCCCCTACTTAGCGGTCGCTC
GTACGTTCGAAAAAATTGAAGAAGTATCGGCGCGTCTGCGTATGGTGGAGACACTTTCCAATCTGCTGCGCTC
TGTTGTTGCCCTGTCGCCTCCCGATTTGTTGCCAGTTTTGTACCTGAGCTTGAACCACCTGGGCCCTCCTCAA
CAAGGCTTAGAATTGGGGGTAGGCGATGGTGTATTGTTGAAAGCAGTAGCGCAAGCCACGGGCCGTCAACTG
GAGTCCGTTCGTGCAGAGGCAGCGGAAAAGGGTGATGTAGGGTTAGTCGCTGAAAACAGCCGCAGTACGCA
GCGTTTGATGTTGCCCCCGCCACCCCTGACGGCCTCCGGAGTGTTCTCAAAGTTCCGCGATATCGCCCGTTT
AACCGGGTCAGCATCTACAGCGAAAAAGATTGACATCATCAAAGGCCTGTTCGTTGCTTGTCGCCACAGCGA
GGCTCGTTTTATTGCGCGCTCTTTATCCGGGCGCCTTCGTCTGGGCTTAGCCGAACAGTCAGTGCTTGCAGC
ATTGTCACAGGCCGTTTCGCTTACTCCCCCAGGGCAGGAGTTCCCCCCTGCCATGGTAGACGCTGGCAAAG
GGAAAACTGCGGAGGCCCGTAAGACATGGTTGGAGGAGCAAGGTATGATCCTTAAGCAGACCTTTTGCGAGG
TCCCAGACCTGGACCGCATCATCCCTGTGTTGCTGGAGCATGGCTTAGAACGCCTTCCGGAGCATTGCAAGC
TGTCCCCTGGGATTCCGTTAAAGCCGATGTTAGCCCATCCTACCCGTGGTATTTCAGAGGTTCTGAAGCGCTT
CGAAGAGGCTGCCTTCACATGTGAATATAAATACGACGGGCAACGCGCACAAATCCACGCTTTGGAGGGGGG
CGAGGTCAAGATCTTTTCGCGTAATCAAGAGGACAACACGGGCAAATACCCCGACATTATTTCGCGCATCCCT
AAAATTAAACTGCCCAGTGTTACGAGCTTCATTTTAGATACAGAAGCGGTGGCTTGGGACCGCGAGAAAAAGC
AAATTCAGCCCTTCCAGGTCTTGACCACGCGTAAACGTAAGGAGGTTGACGCGTCGGAAATCCAAGTACAAG
TCTGTCTGTACGCCTTCGATTTAATCTATTTAAATGGTGAAAGTTTGGTGCGTGAACCATTATCCCGCCGCCGT
CAGCTGCTTCGTGAAAACTTCGTTGAAACCGAGGGCGAATTTGTTTTTGCGACTTCACTGGATACAAAAGACA
TCGAACAAATCGCGGAGTTCCTTGAGCAATCCGTCAAAGACAGTTGCGAGGGACTGATGGTCAAGACCTTAG
ACGTTGATGCCACTTATGAGATCGCTAAACGCTCCCATAATTGGTTGAAACTGAAAAAGGACTACCTTGATGGC
GTGGGTGATACACTTGACCTTGTGGTCATCGGAGCCTATCTGGGACGTGGTAAGCGCGCAGGACGCTACGGT
GGTTTCTTGTTAGCCTCCTATGACGAGGATAGTGAGGAATTGCAGGCCATTTGTAAGCTGGGTACGGGGTTCT
CTGACGAAGAGTTGGAGGAACACCACCAATCACTTAAAGCATTGGTCTTGCCCAGTCCTCGTCCCTACGTCC
GTATCGACGGGGCAGTAATTCCGGATCACTGGCTTGACCCTAGCGCGGTCTGGGAAGTTAAATGCGCTGATC
TGTCCTTAAGTCCAATCTATCCCGCGGCACGCGGGCTTGTCGATTCTGACAAAGGGATCTCGCTTCGTTTTCC
CCGTTTCATCCGTGTGCGCGAGGACAAACAACCCGAACAAGCCACGACGAGTGCACAGGTGGCGTGCCTGT
ACCGCAAACAATCGCAGATTCAGAATCAGCAAGGCGAAGATTCAGGGAGCGACCCGGAGGACACGTATTAAT
GAGGATCCCTCGAGCACCACCACCACCACCACTGAGATCCGGCTGCTAACAAAGCCCGAAAGGAAGCTGAG
TTGGCTGCTGCCACCGCTGAGCAATAACTAGCATAACCCCTTGGGGCCTCTAAACGGGTCTTGAGGGGTTTTT
TGCTGAAAGGAGGAACTATATCCGGAT

NMR Spectra

tert-Butyl (tert-butoxycarbonyl)(5-chloro-2-fluorophenyl)carbamate & *tert*-butyl (5-chloro-2-fluorophenyl)carbamate (48)

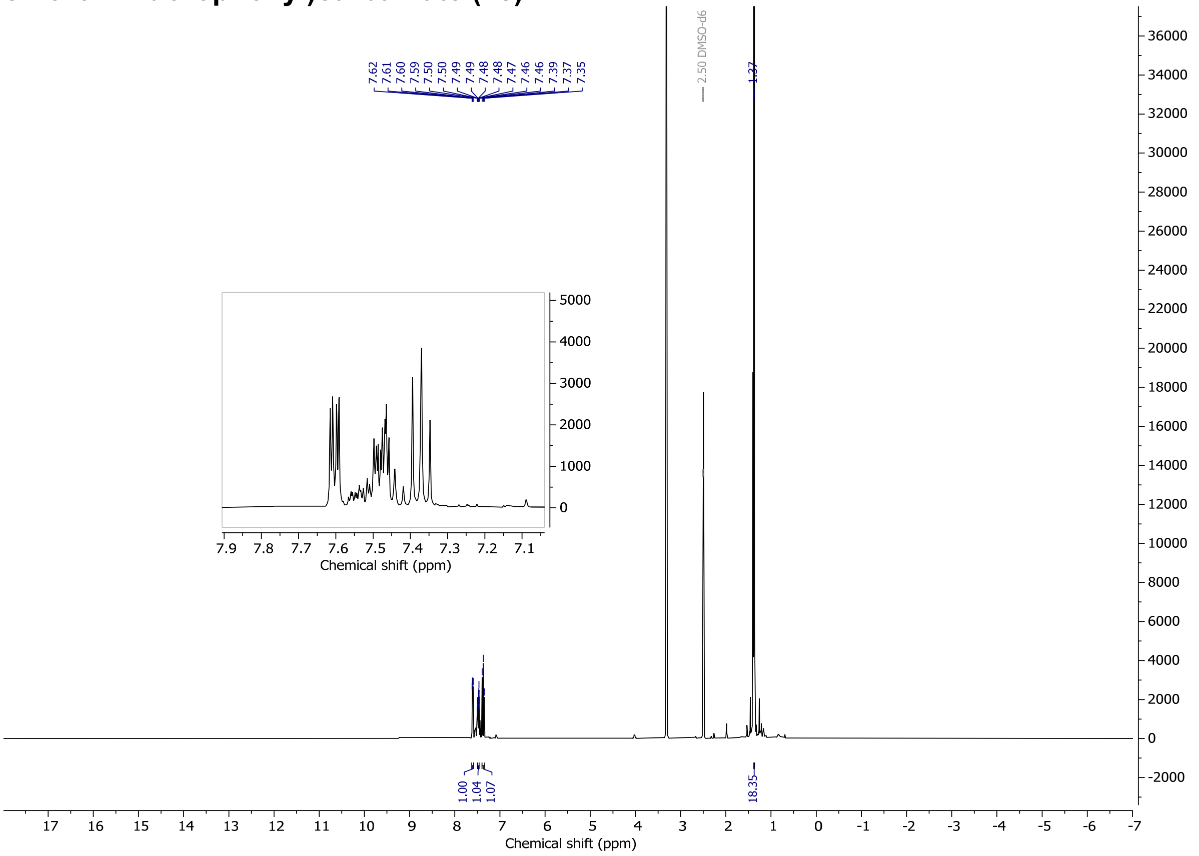

tert-Butyl (*tert*-butoxycarbonyl)(5-chloro-3-fluorophenyl)carbamate & *tert*-butyl (5-chloro-3-fluorophenyl)carbamate (49)

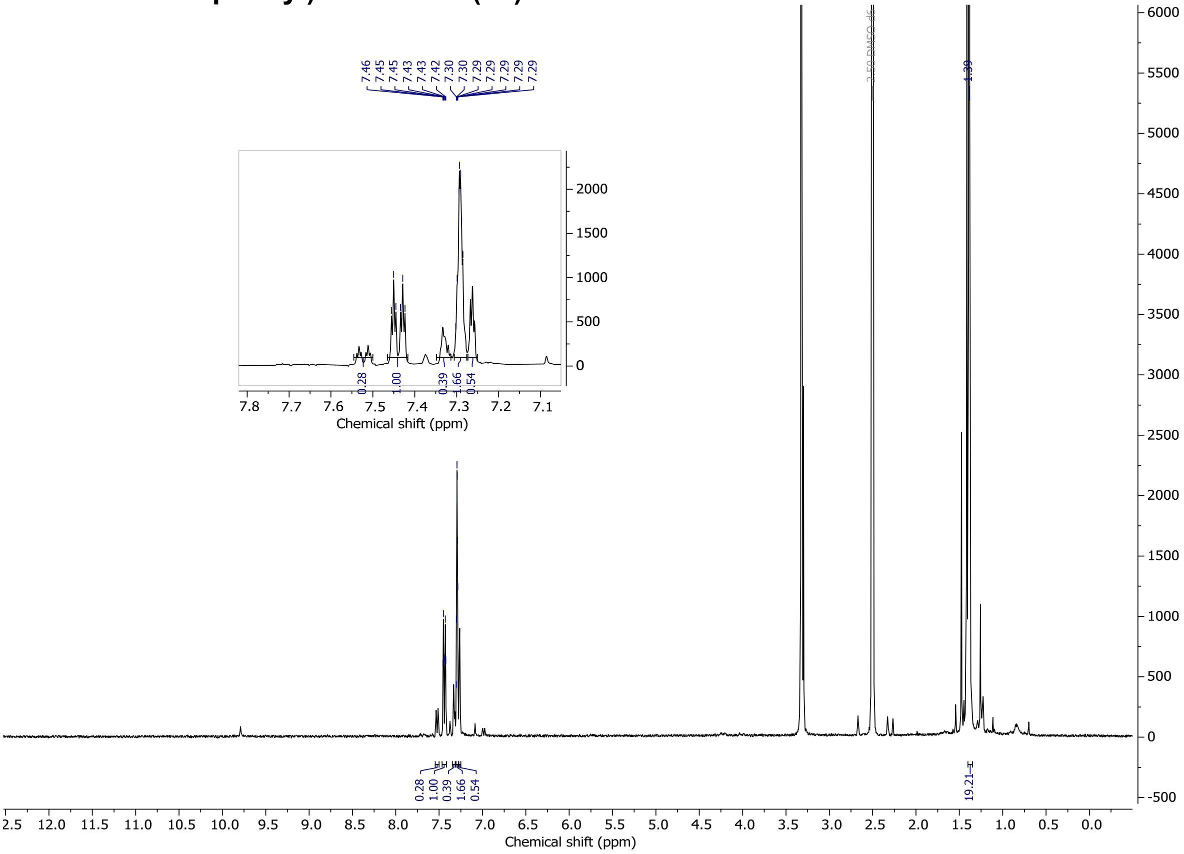

Ethyl 4-(quinolin-4-ylamino)benzoate (50)

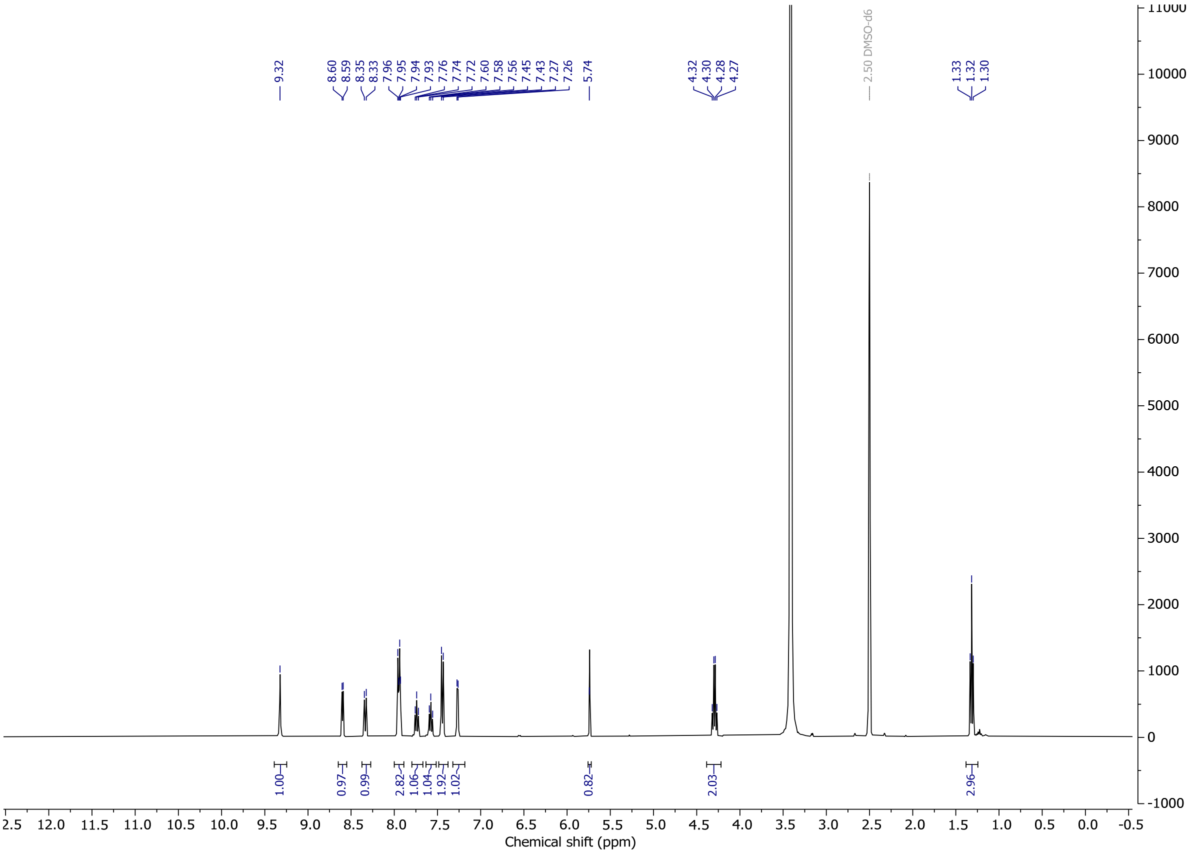

6-Methyl-*N*4-(4-nitrophenyl)pyrimidine-2,4-diamine (51)

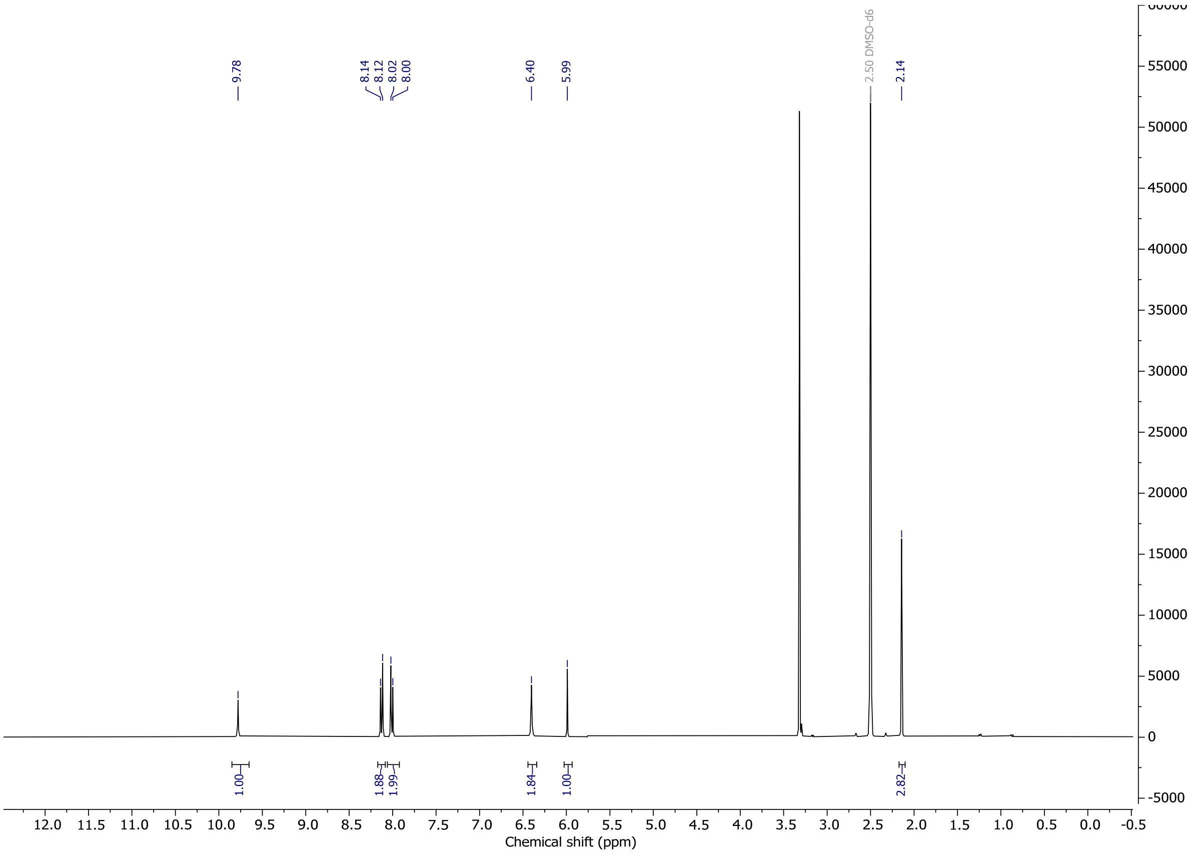

*N*4-(4-Nitrophenyl)pyrimidine-2,4-diamine (52)

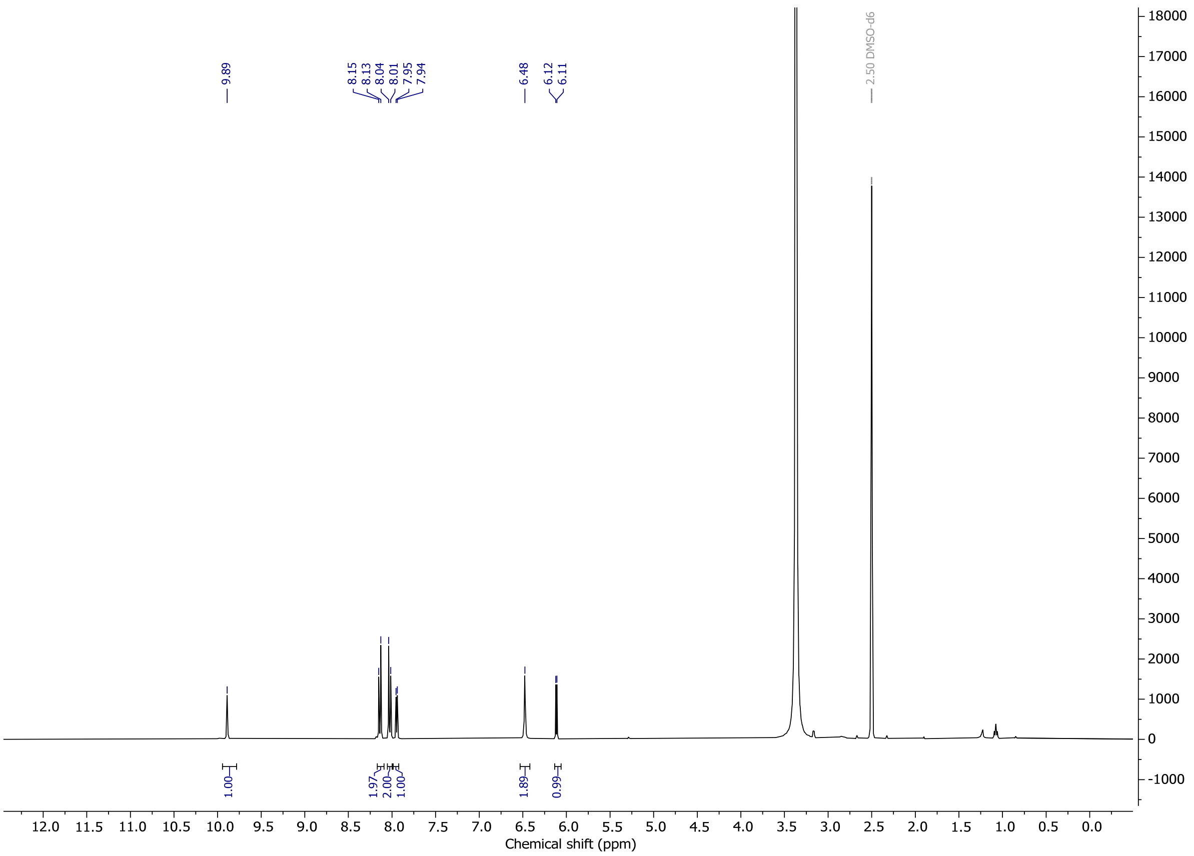

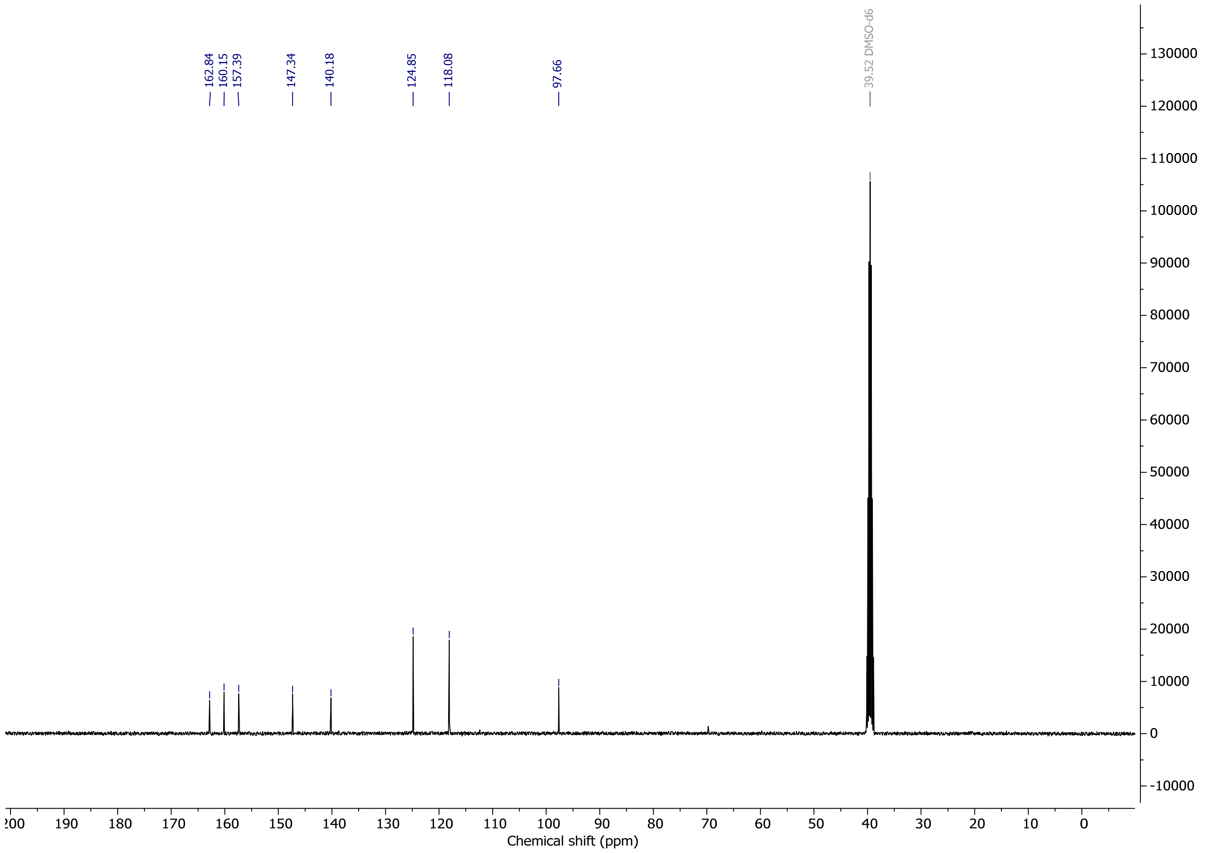

N-(4-Nitrophenyl)-5-(trifluoromethyl)pyridin-2-amine (53)

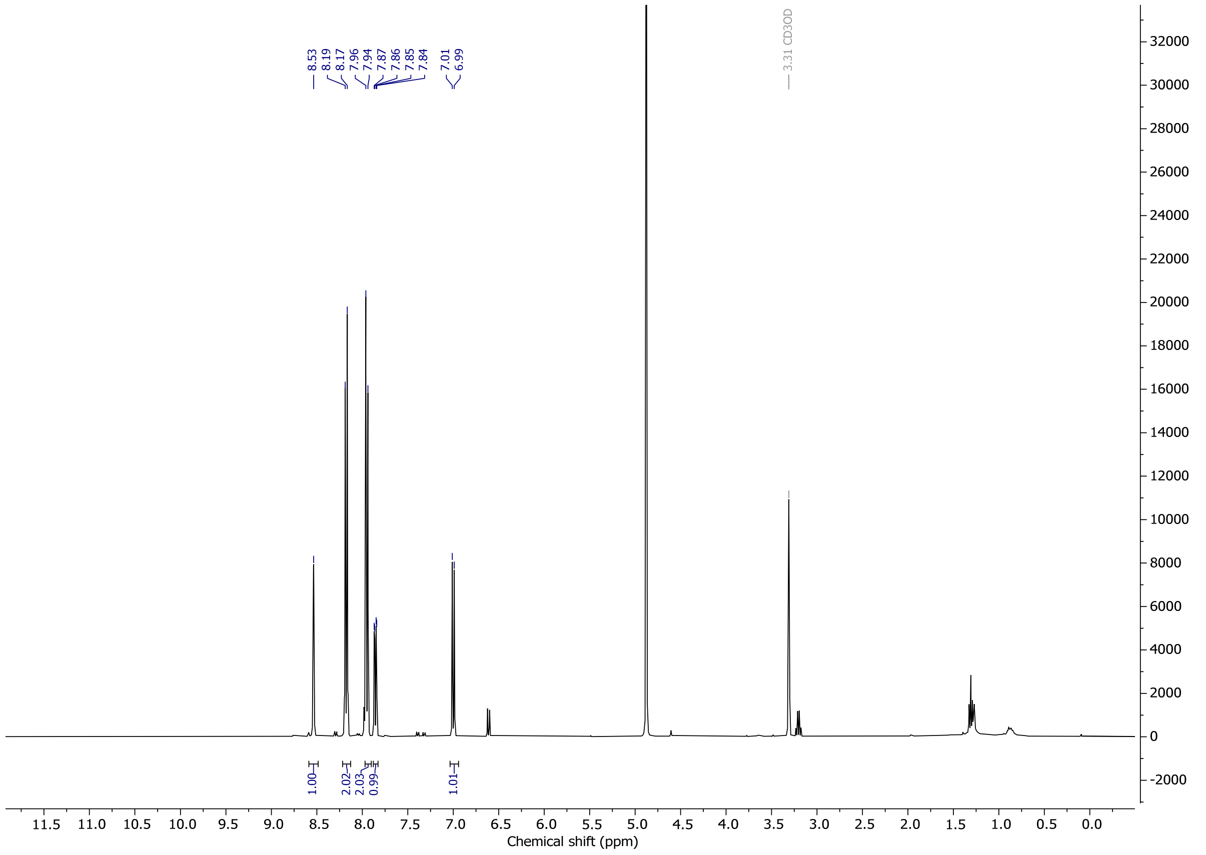

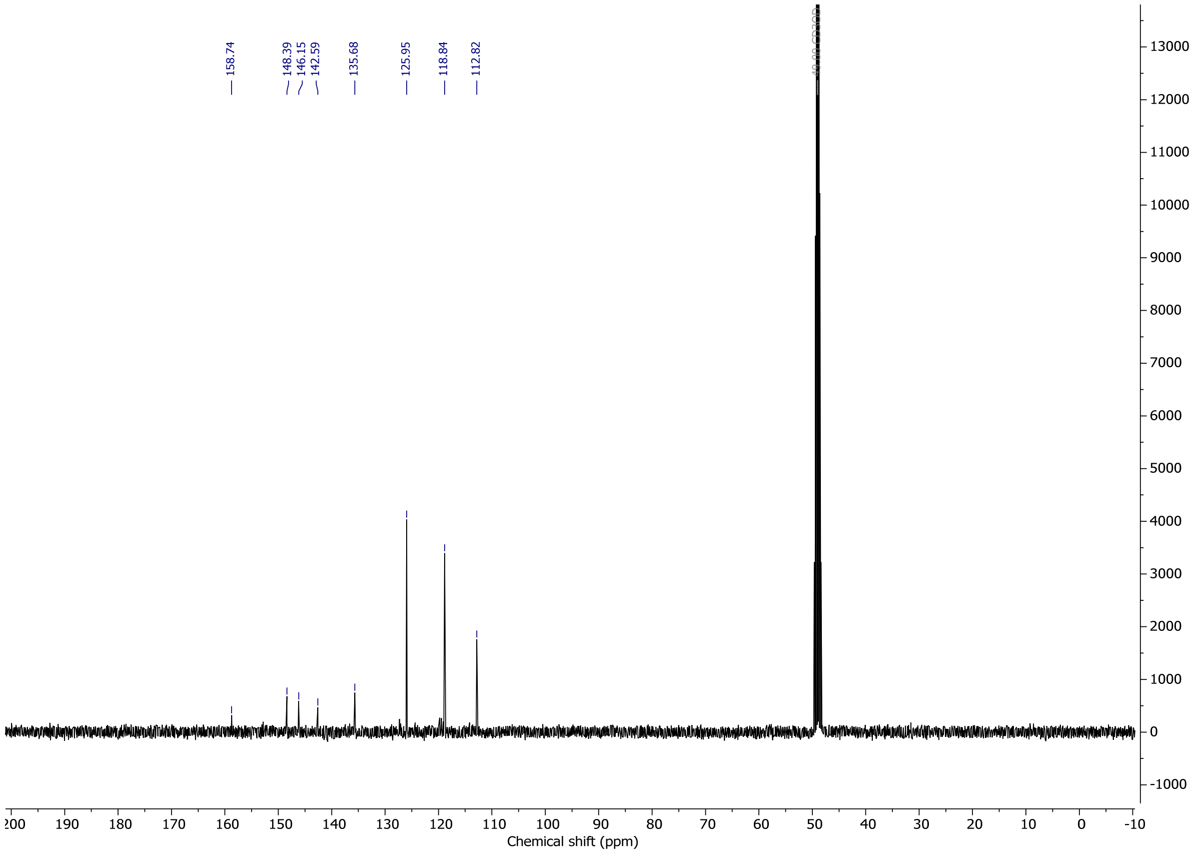

4-Nitro-*N*-(4-(trifluoromethyl)phenyl)aniline (54)

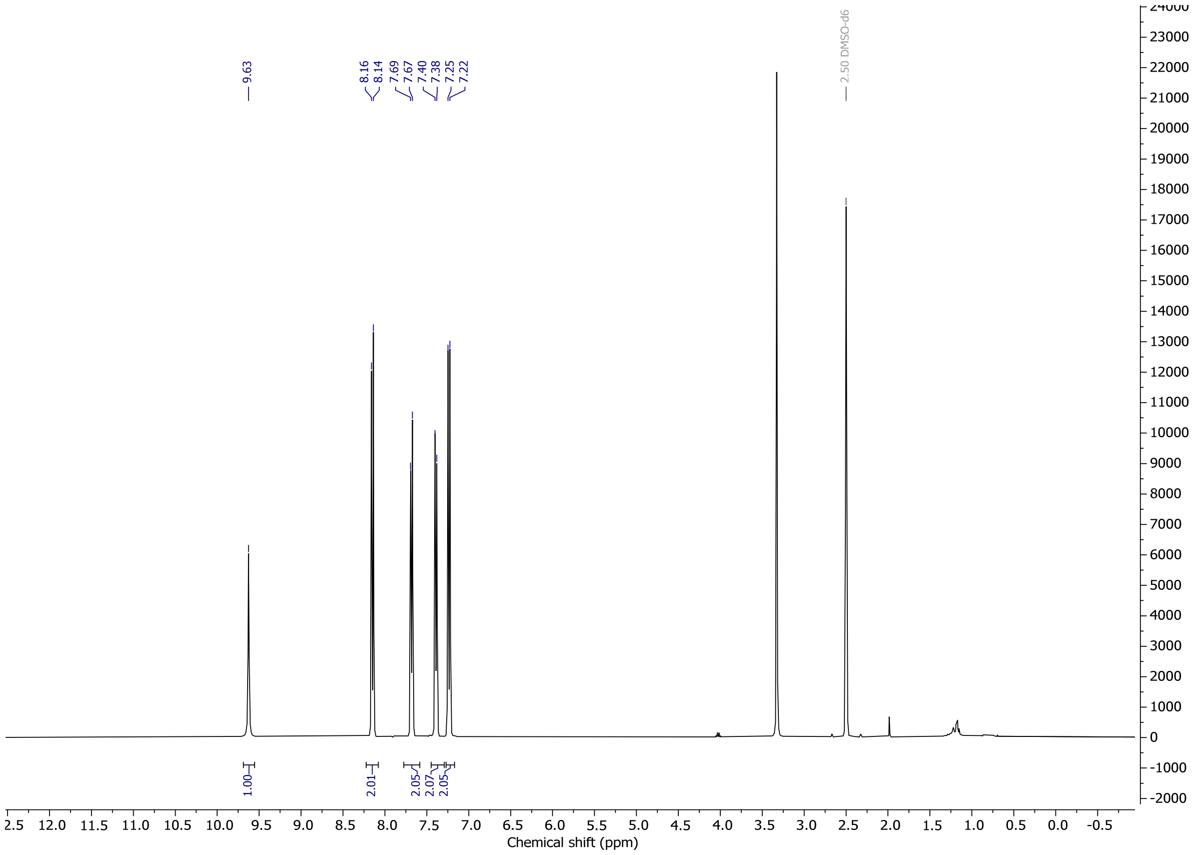

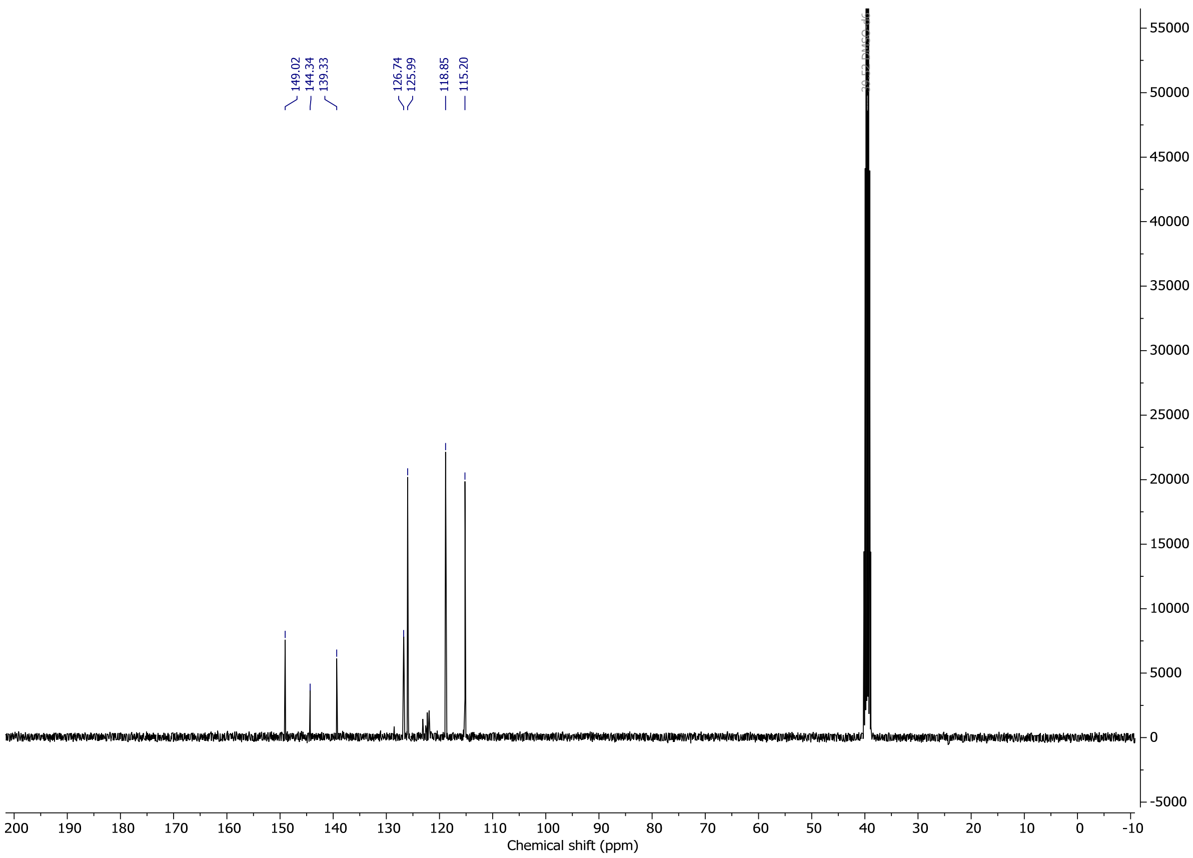

N-(4-Nitrophenyl)-2-(trifluoromethyl)aniline (55)

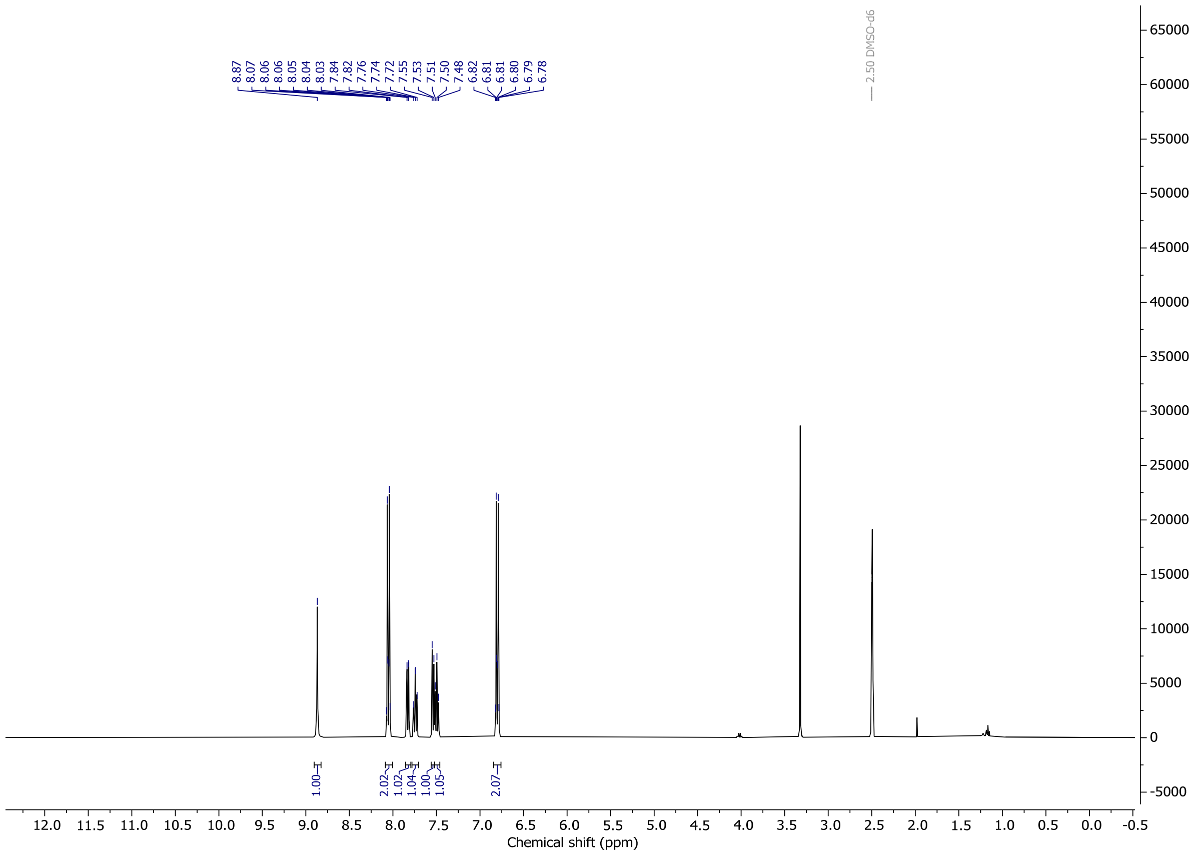

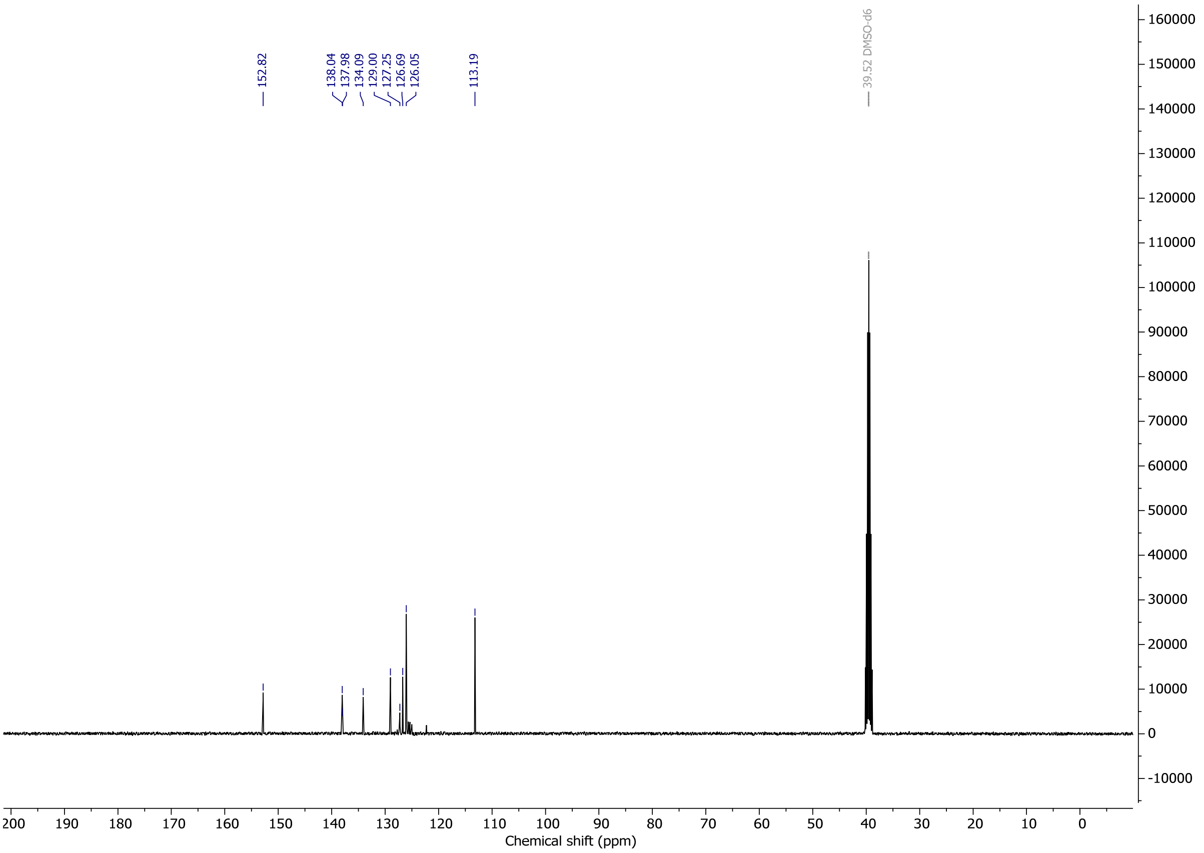

4-Methyl-*N*-(4-nitrophenyl)aniline (56)

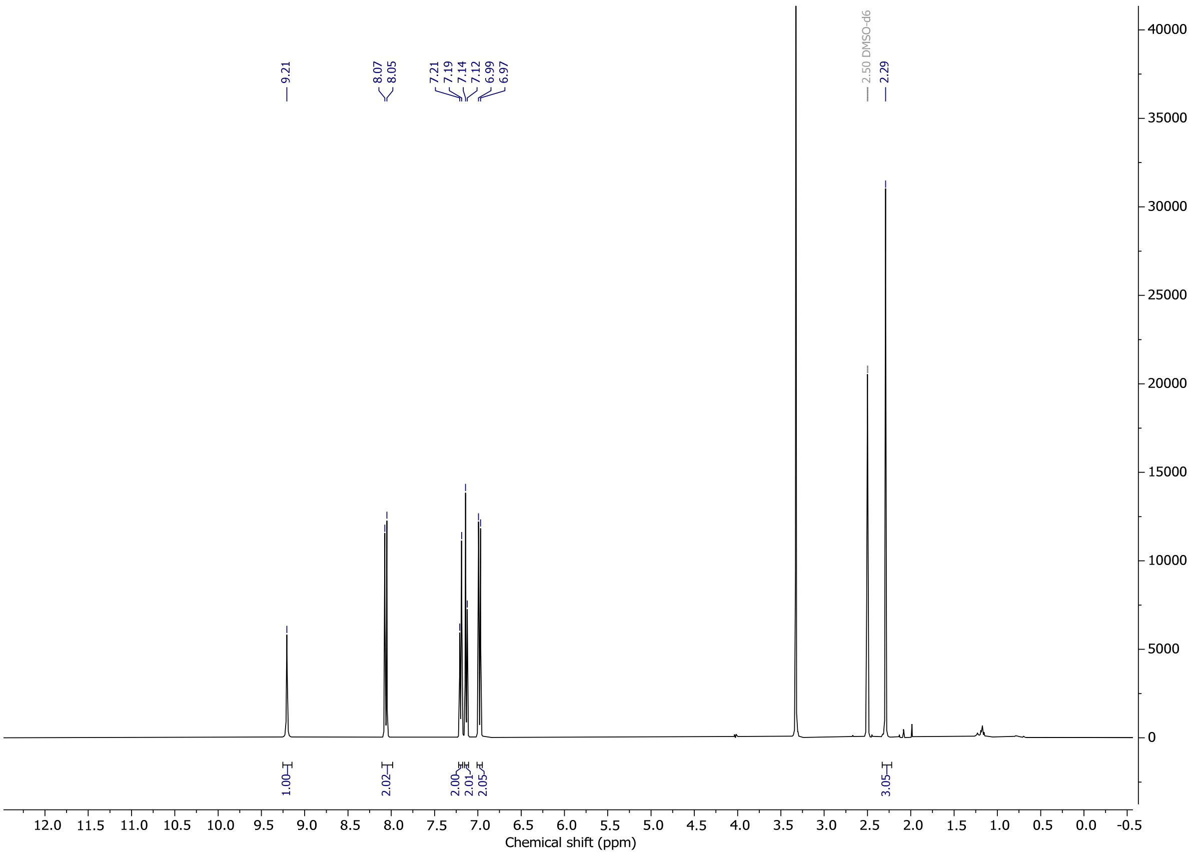

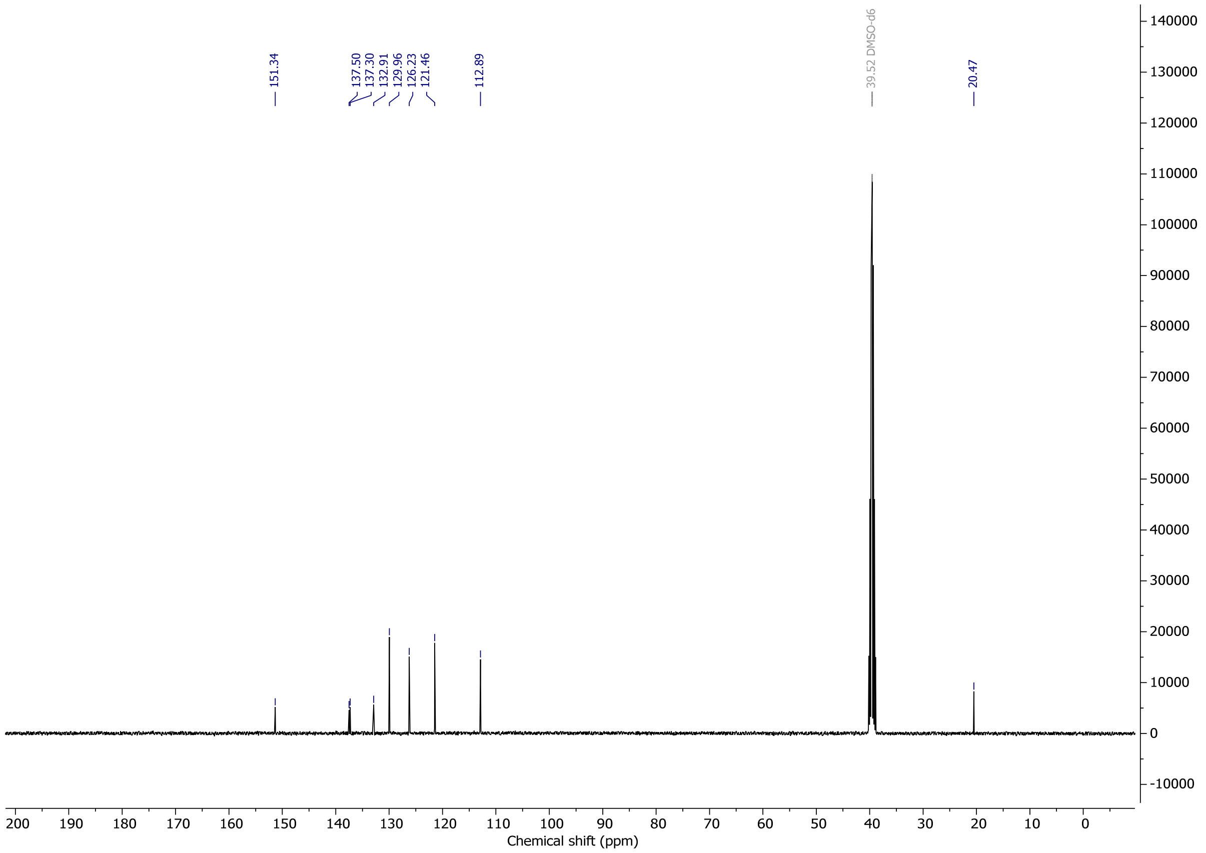

4-Nitro-*N*-phenylaniline (57)

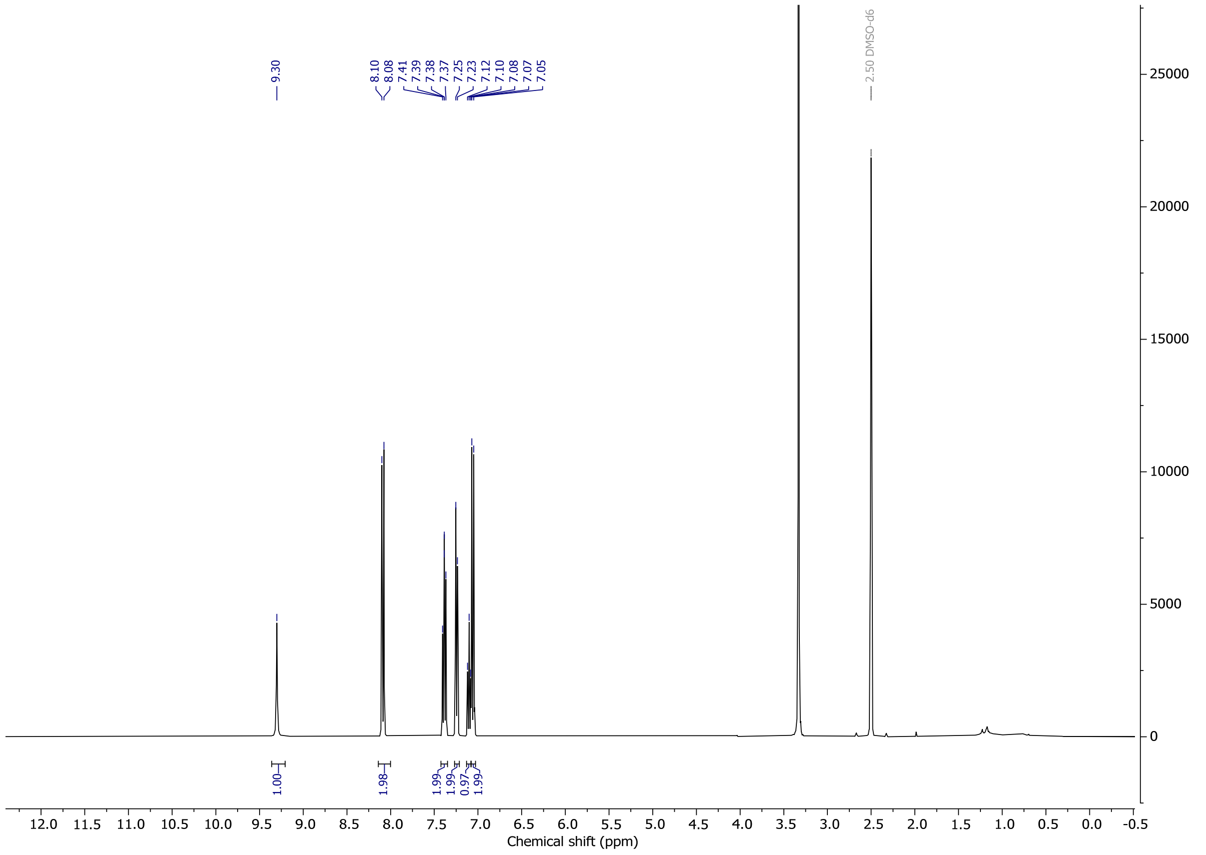

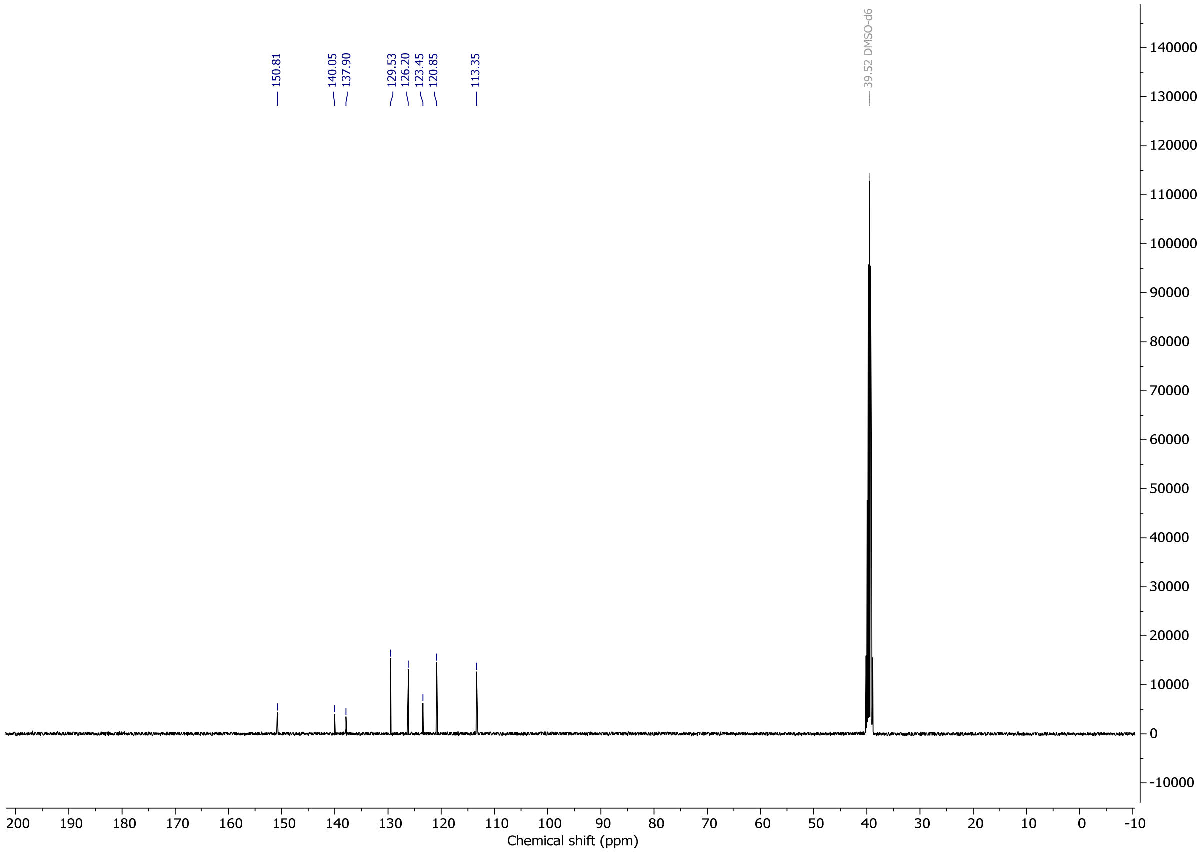

4-Methoxy-*N*-(4-nitrophenyl)aniline (58)

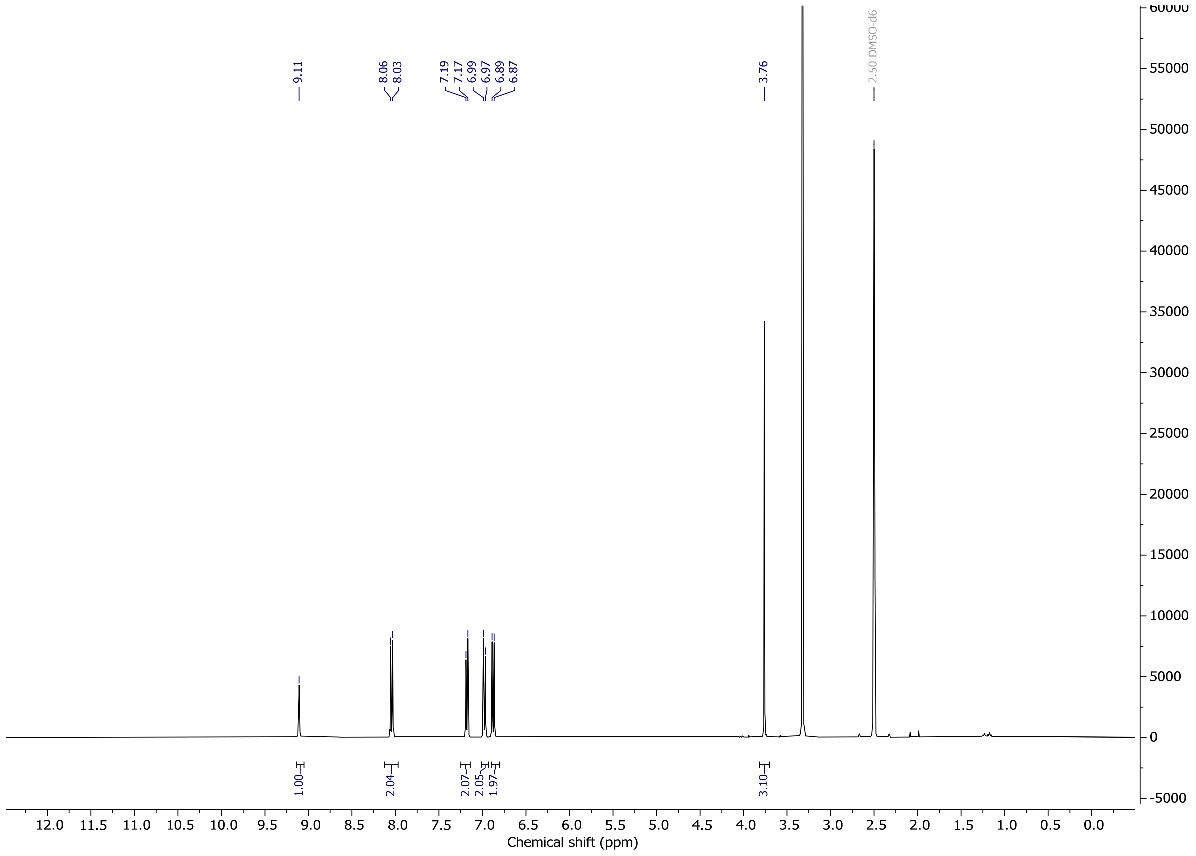

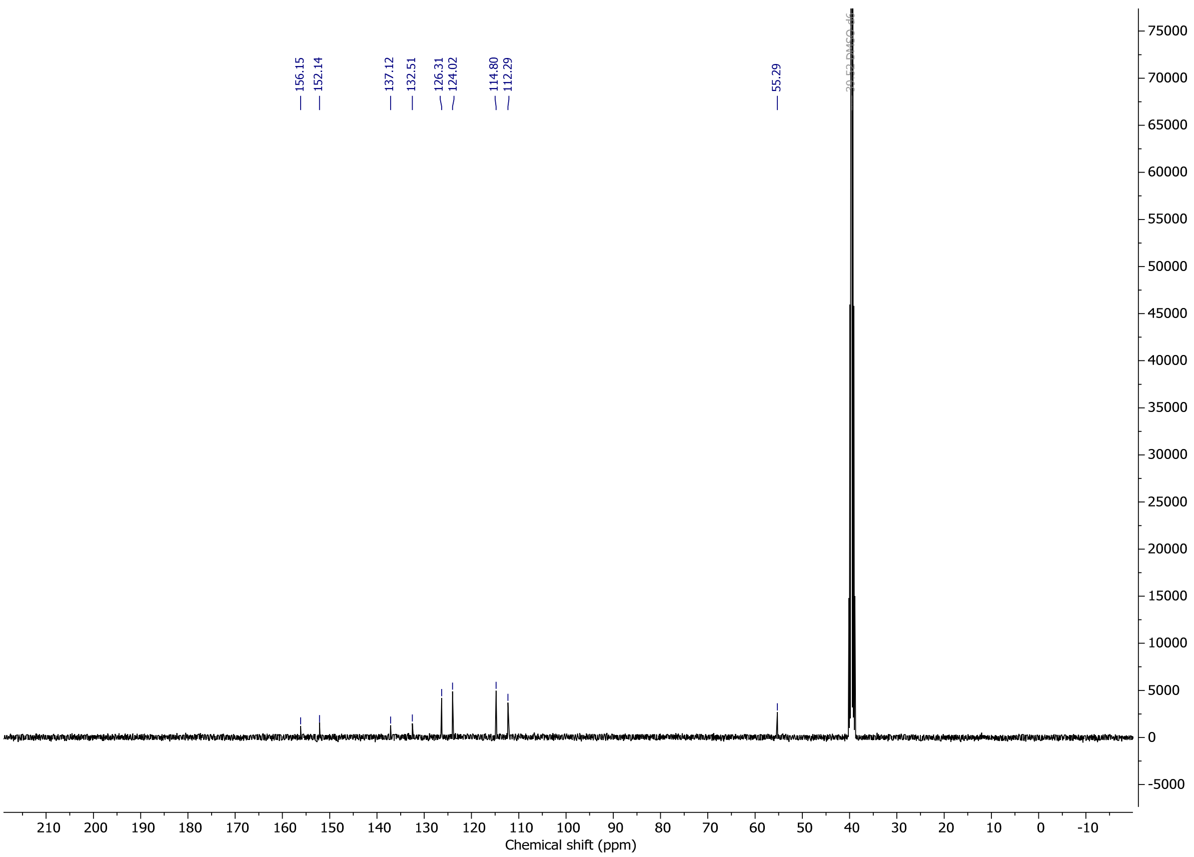

4-Fluoro-*N*-(4-nitrophenyl)aniline (59)

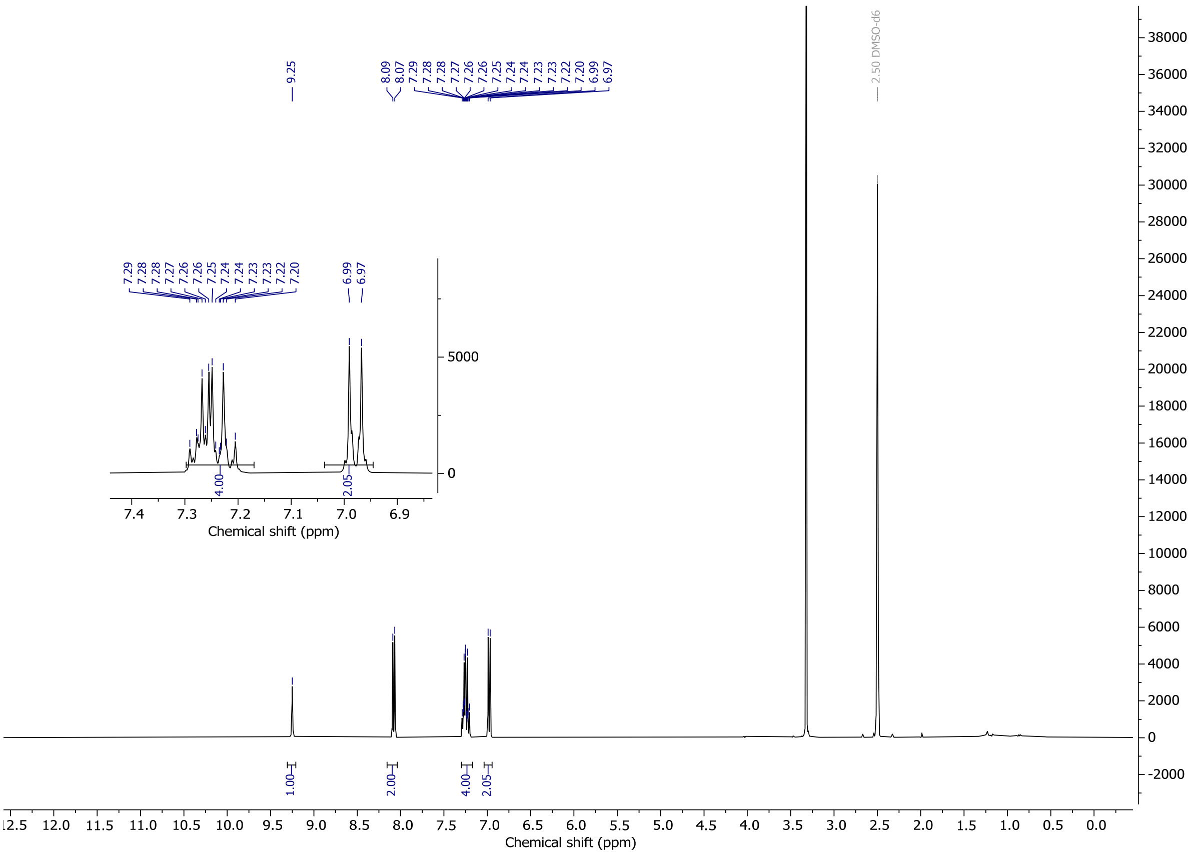

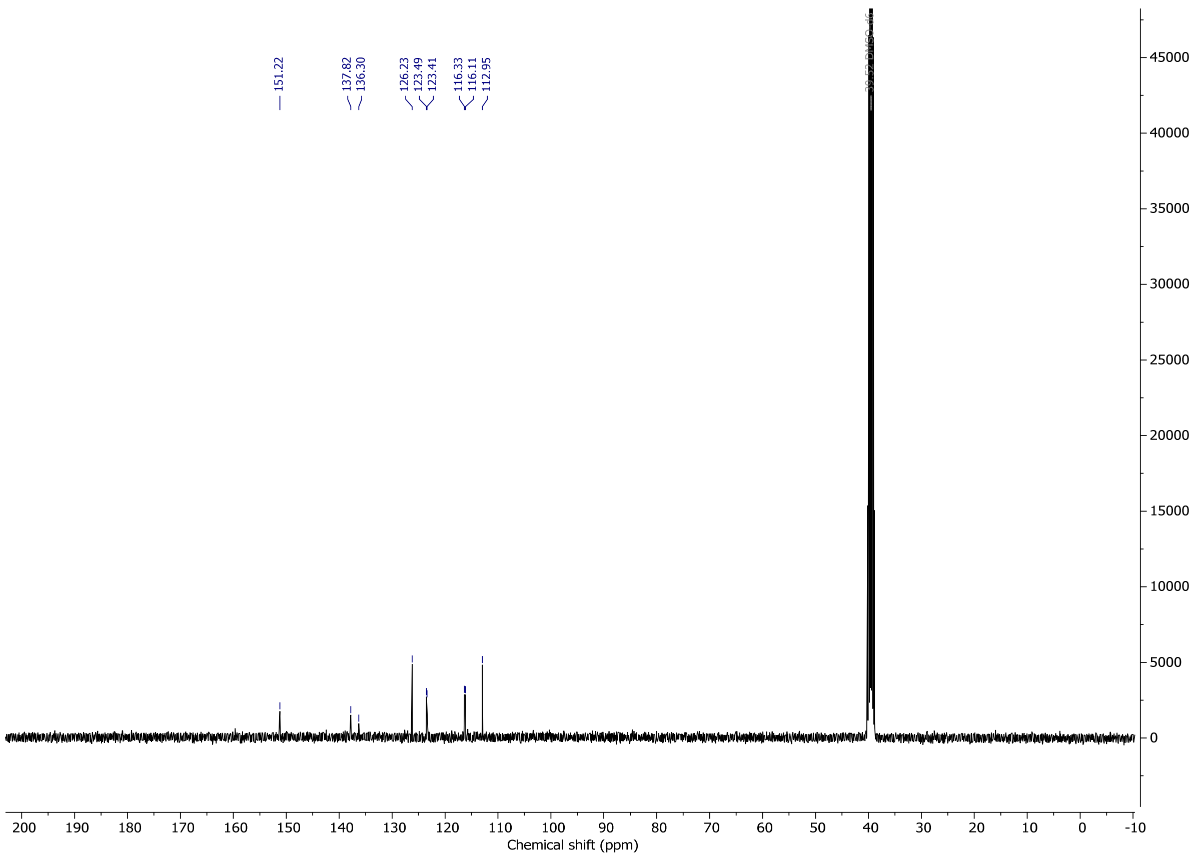

N[2]-(4-Nitrophenyl)pyridine-2,6-diamine (60)

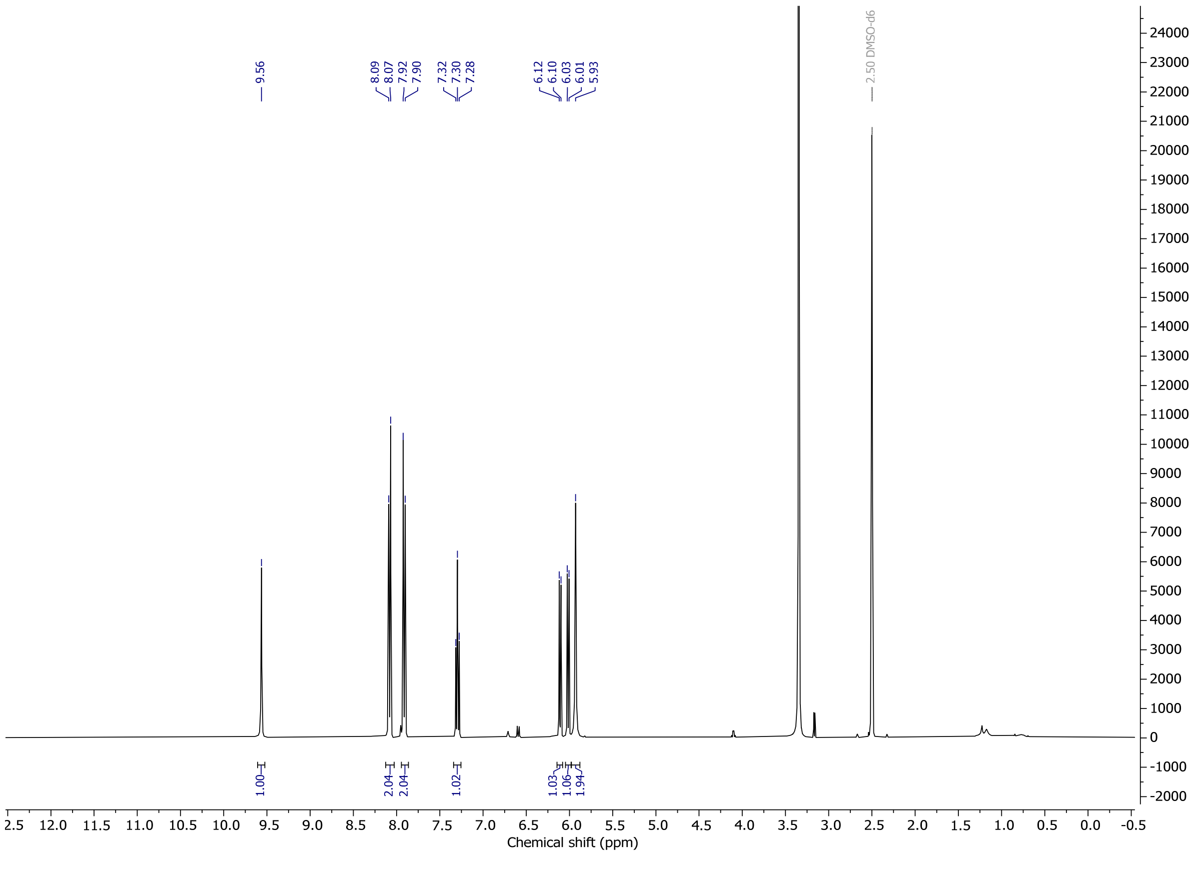

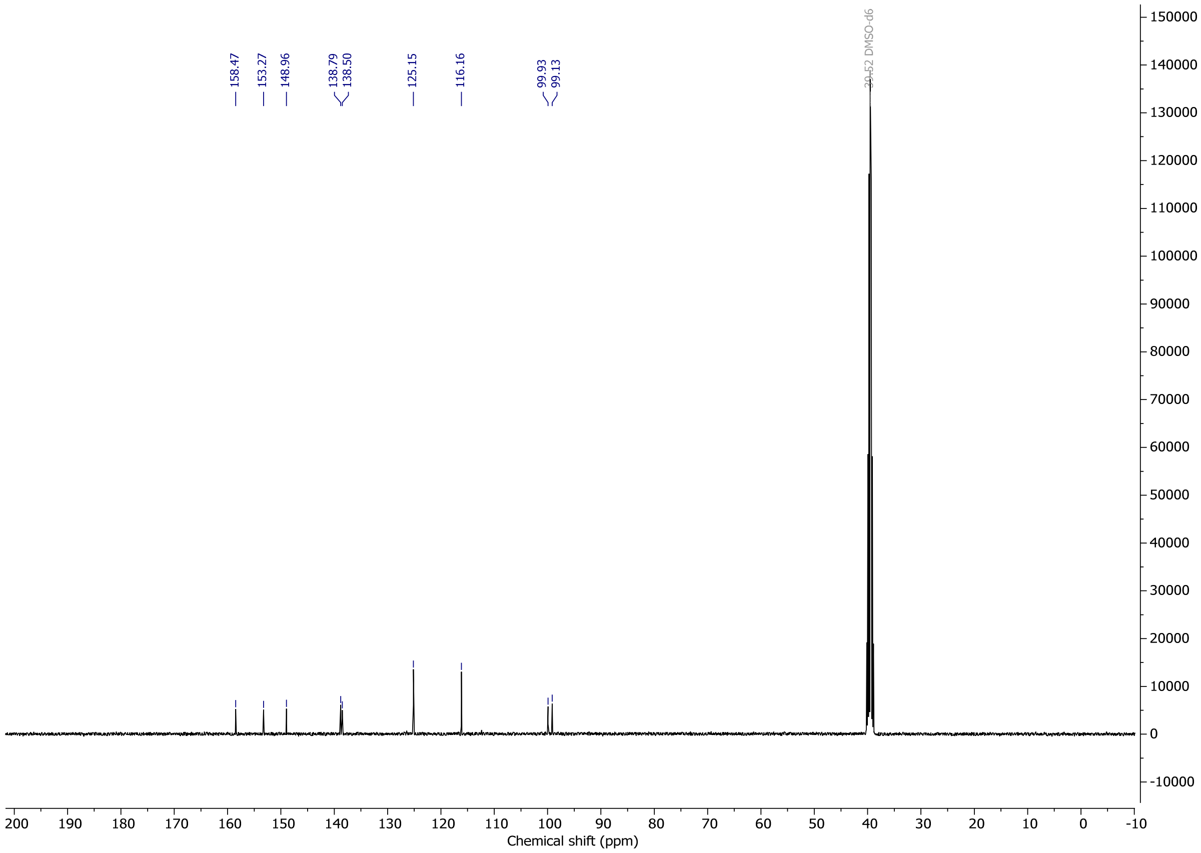

N^4-(4-Nitrophenyl)pyridine-2,4-diamine (61)

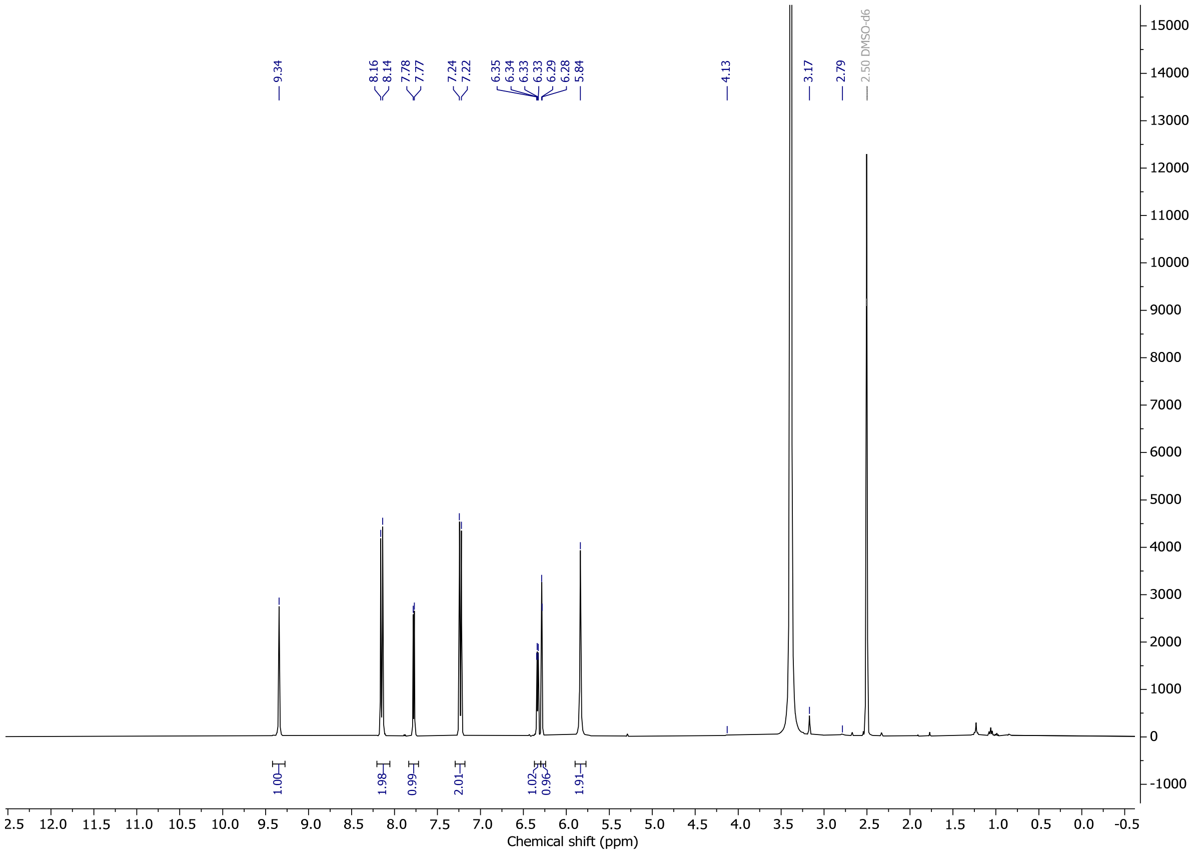

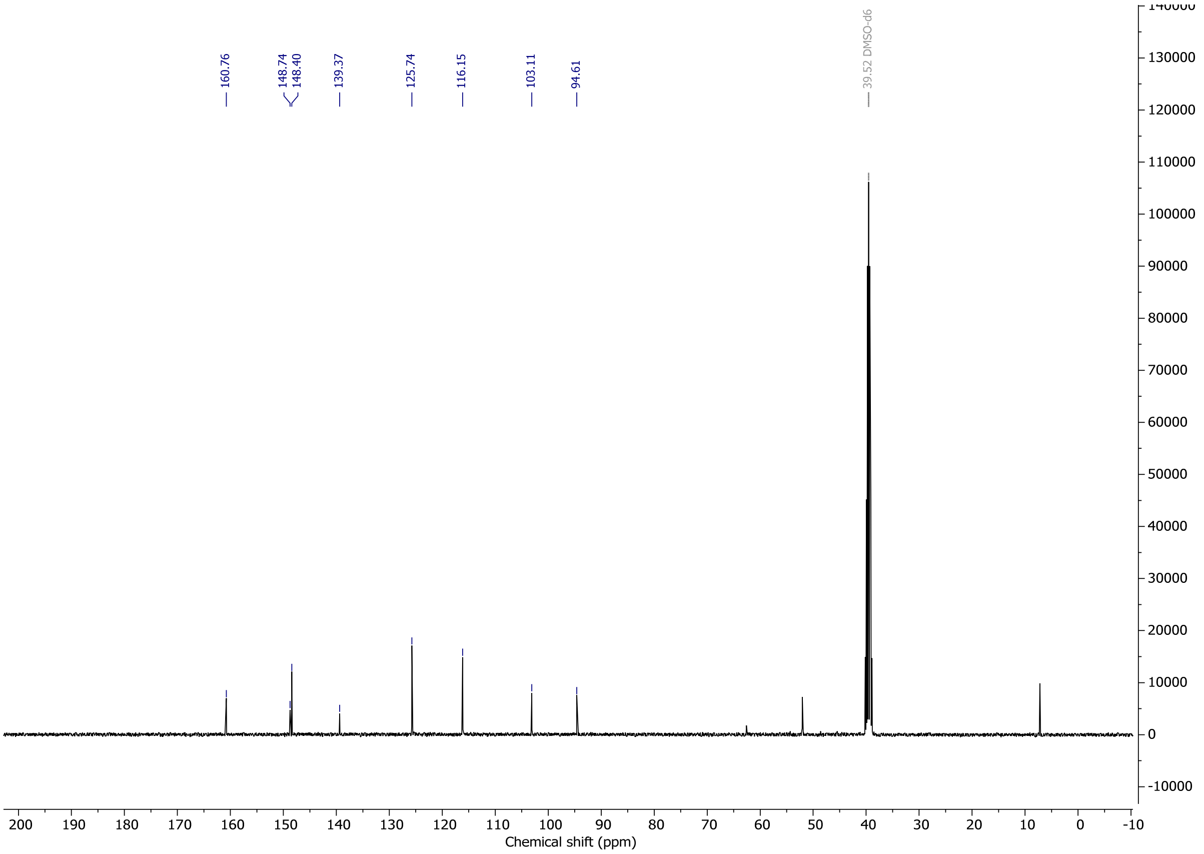

tert-Butyl (*tert*-butoxycarbonyl)(2-fluoro-5-((4-nitrophenyl)amino)phenyl)carbamate (62)

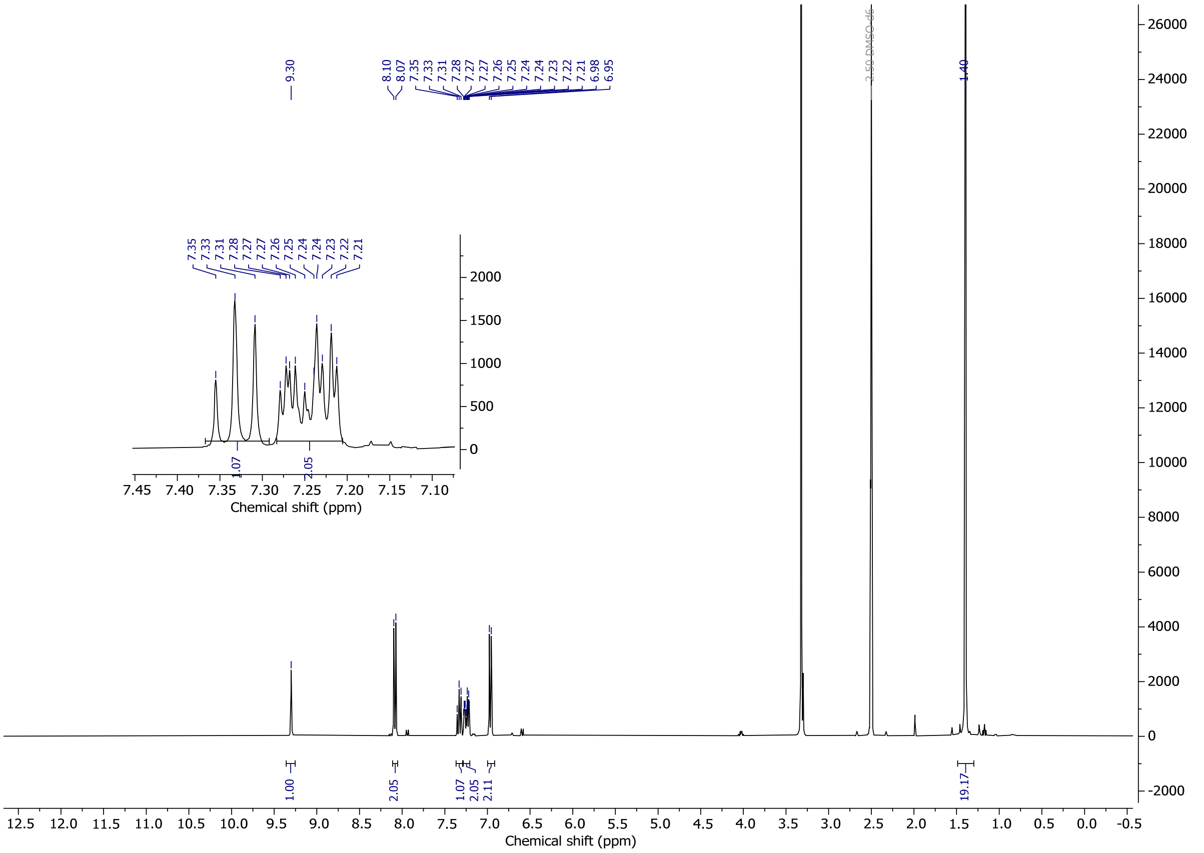

tert-Butyl (*tert*-butoxycarbonyl)(3-fluoro-5-((4-nitrophenyl)amino)phenyl)carbamate & *tert*-butyl (3-fluoro-5-((4-nitrophenyl)amino)phenyl)carbamate (63)

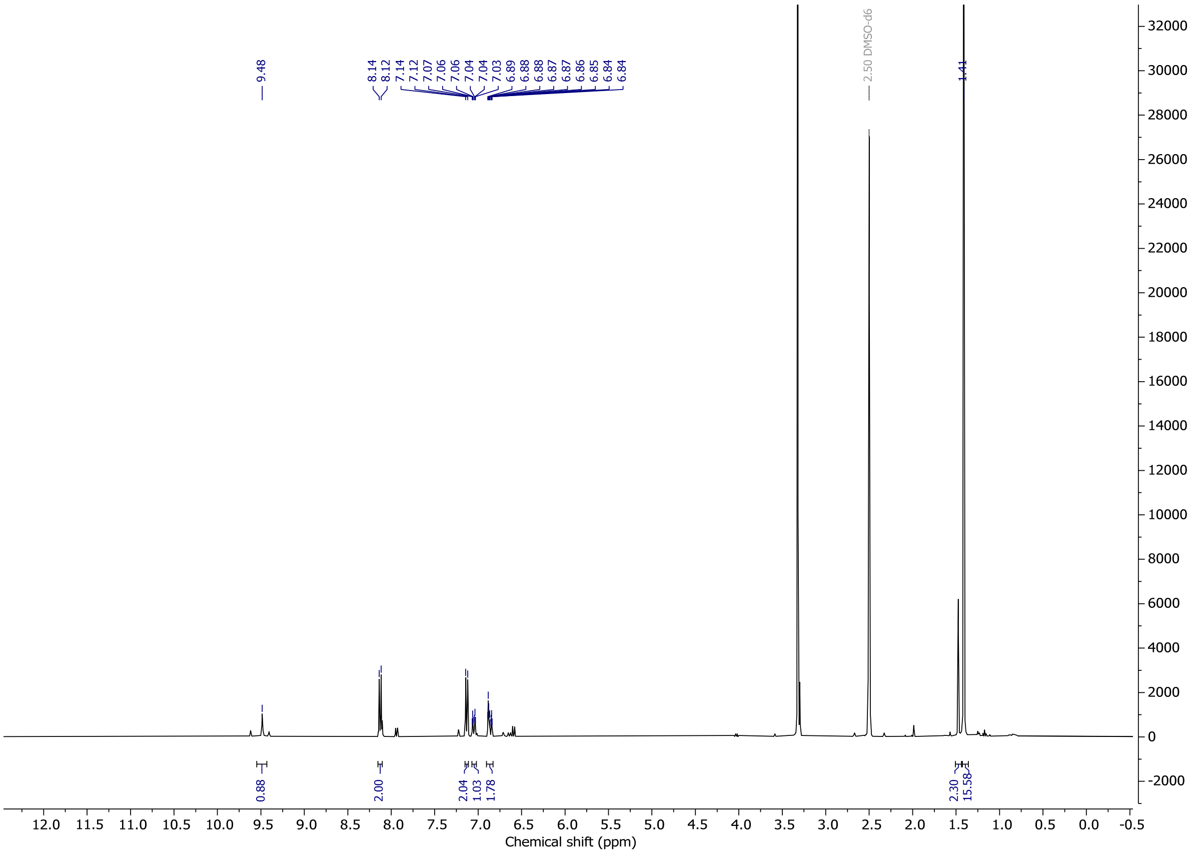

N^4-(4-Nitrophenyl)-6-(trifluoromethyl)pyrimidine-2,4-diamine (64)

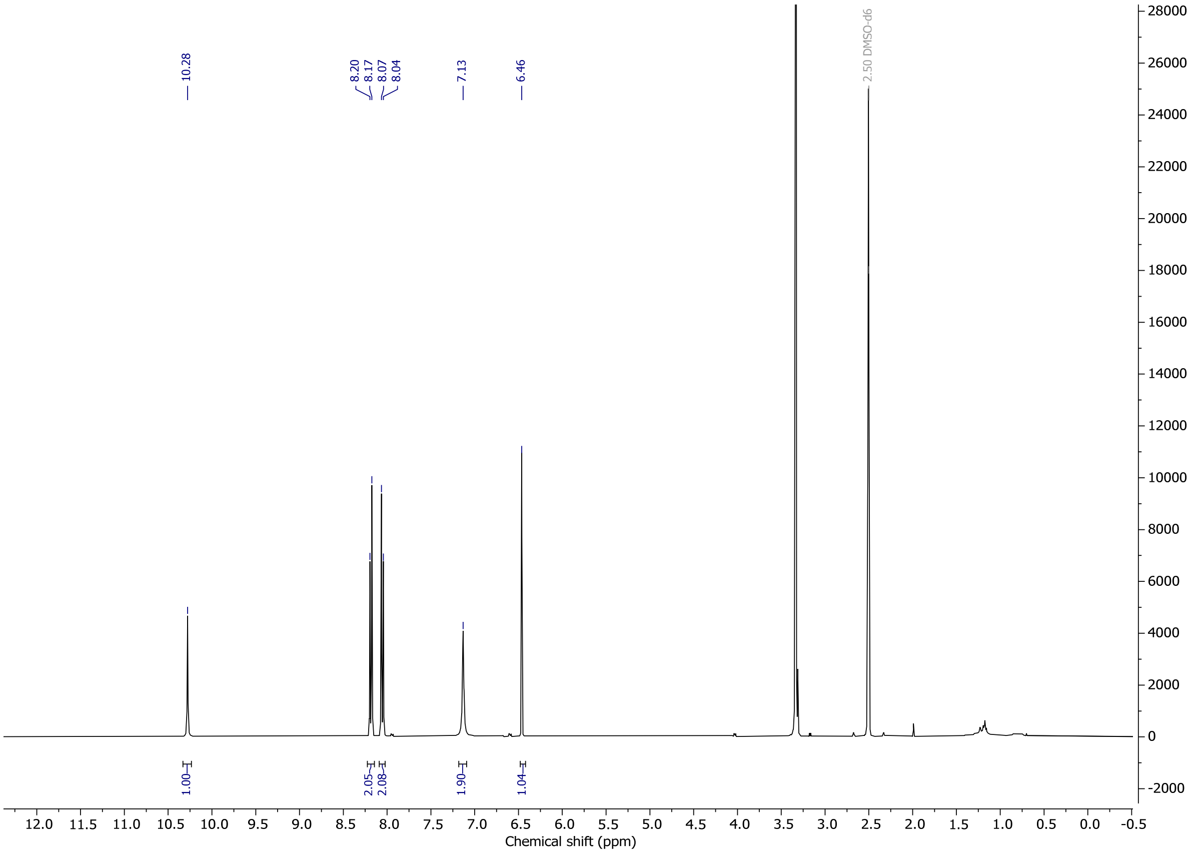

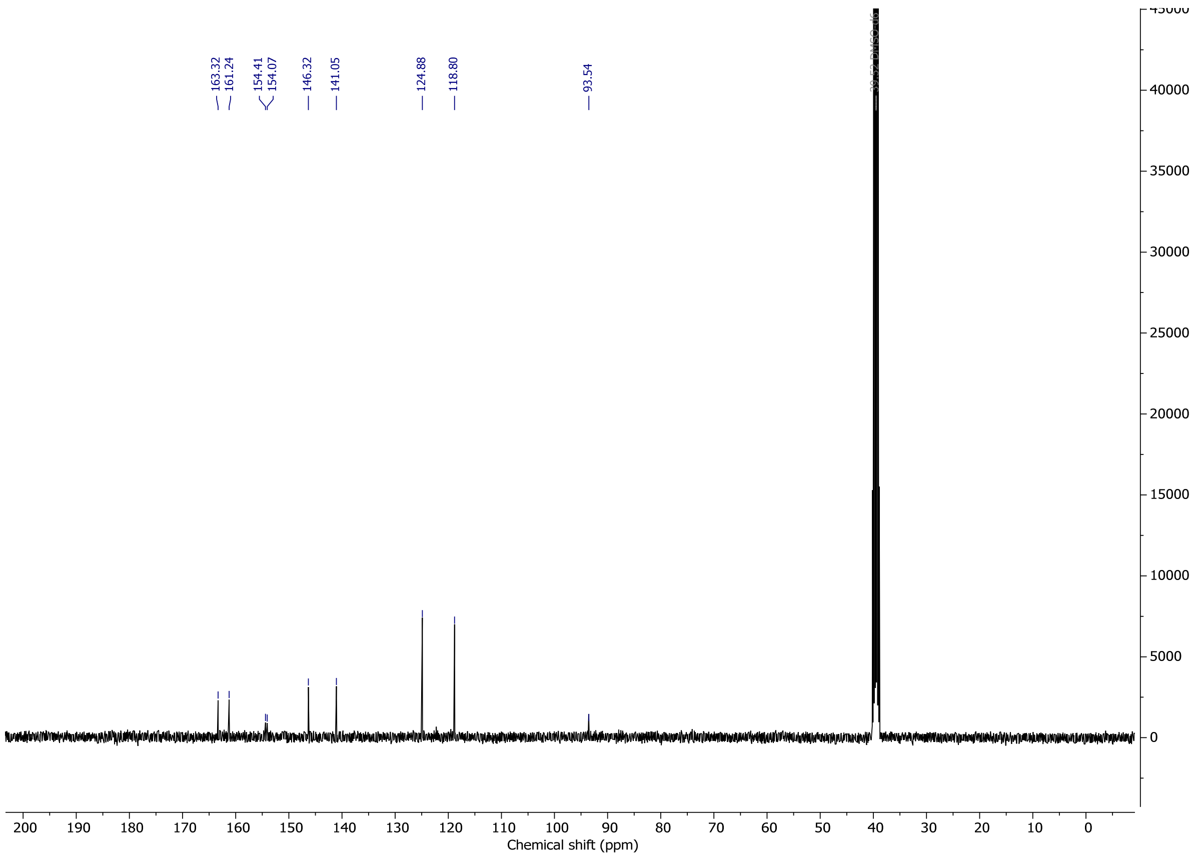

N^1-(3-Nitrophenyl)benzene-1,4-diamine (65)

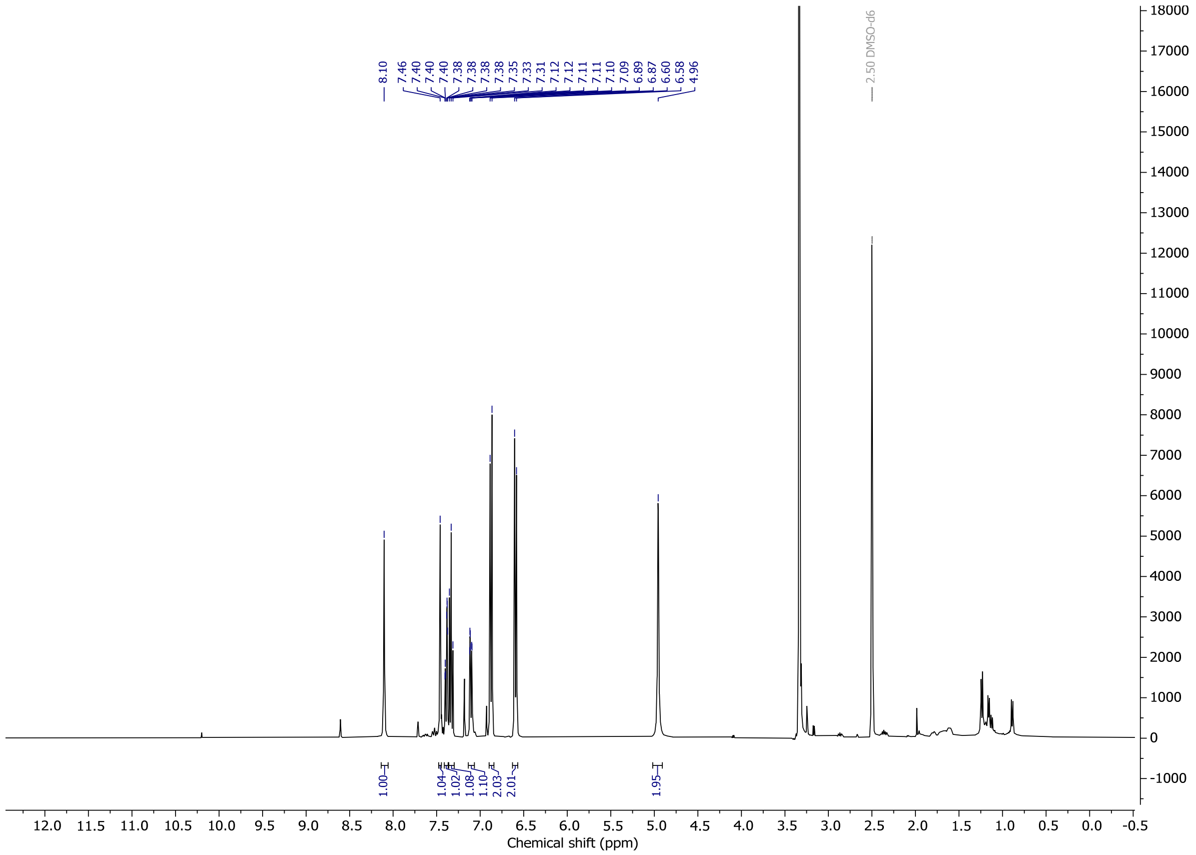

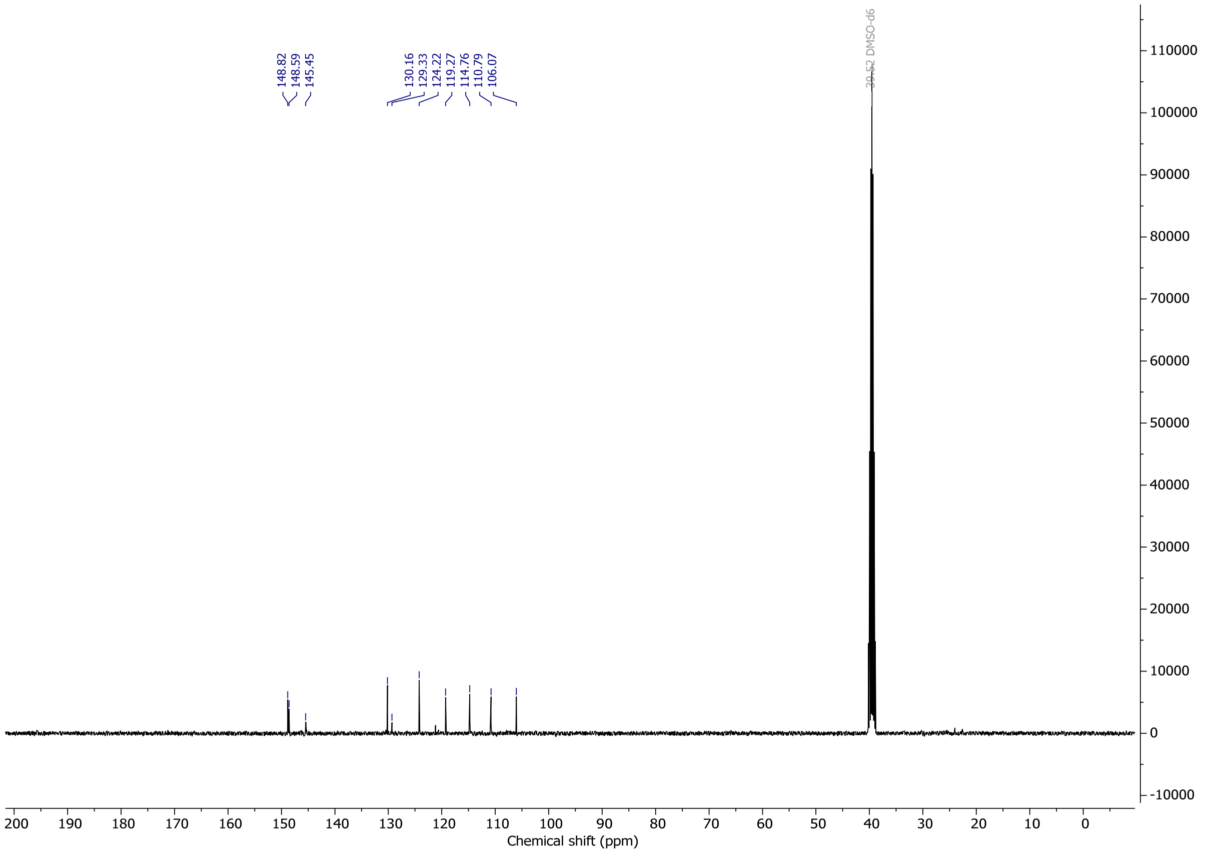

N^1-(3-Methyl-5-nitrophenyl)benzene-1,4-diamine (66)

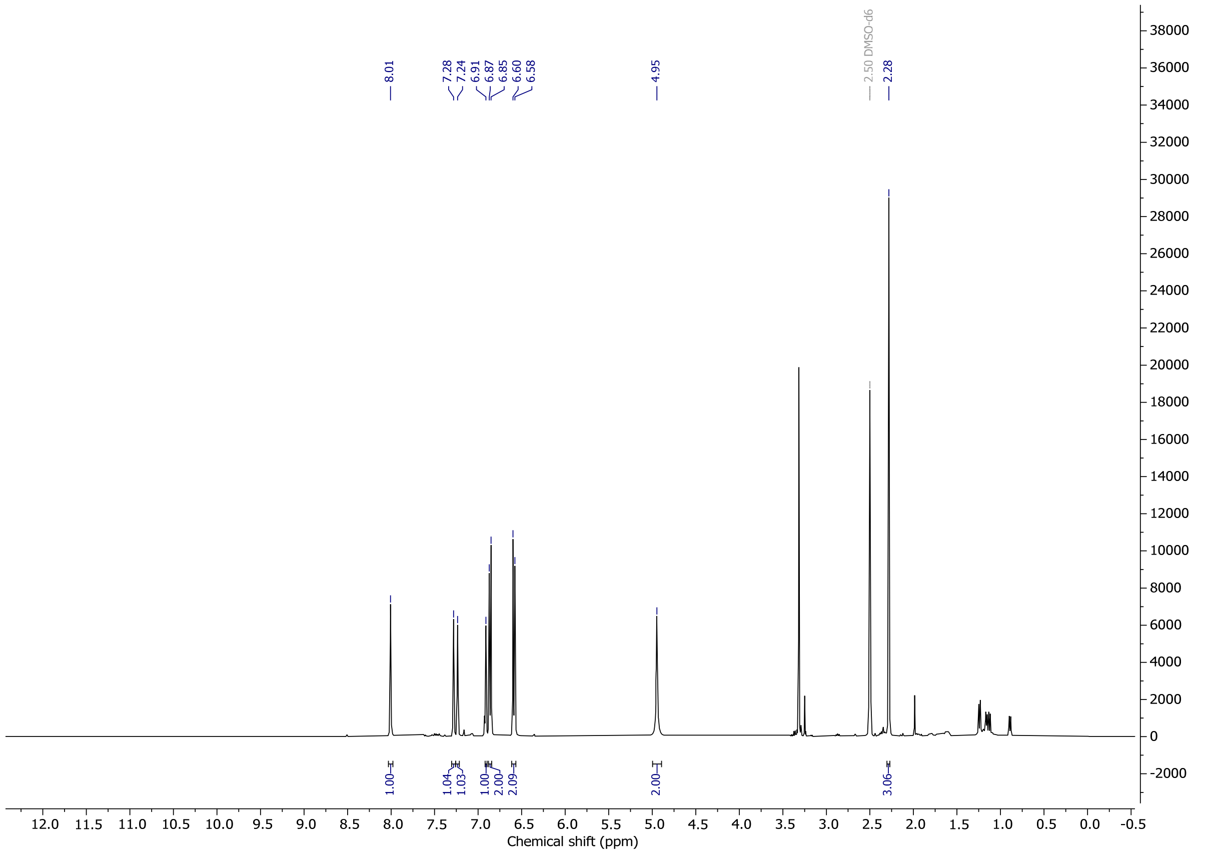

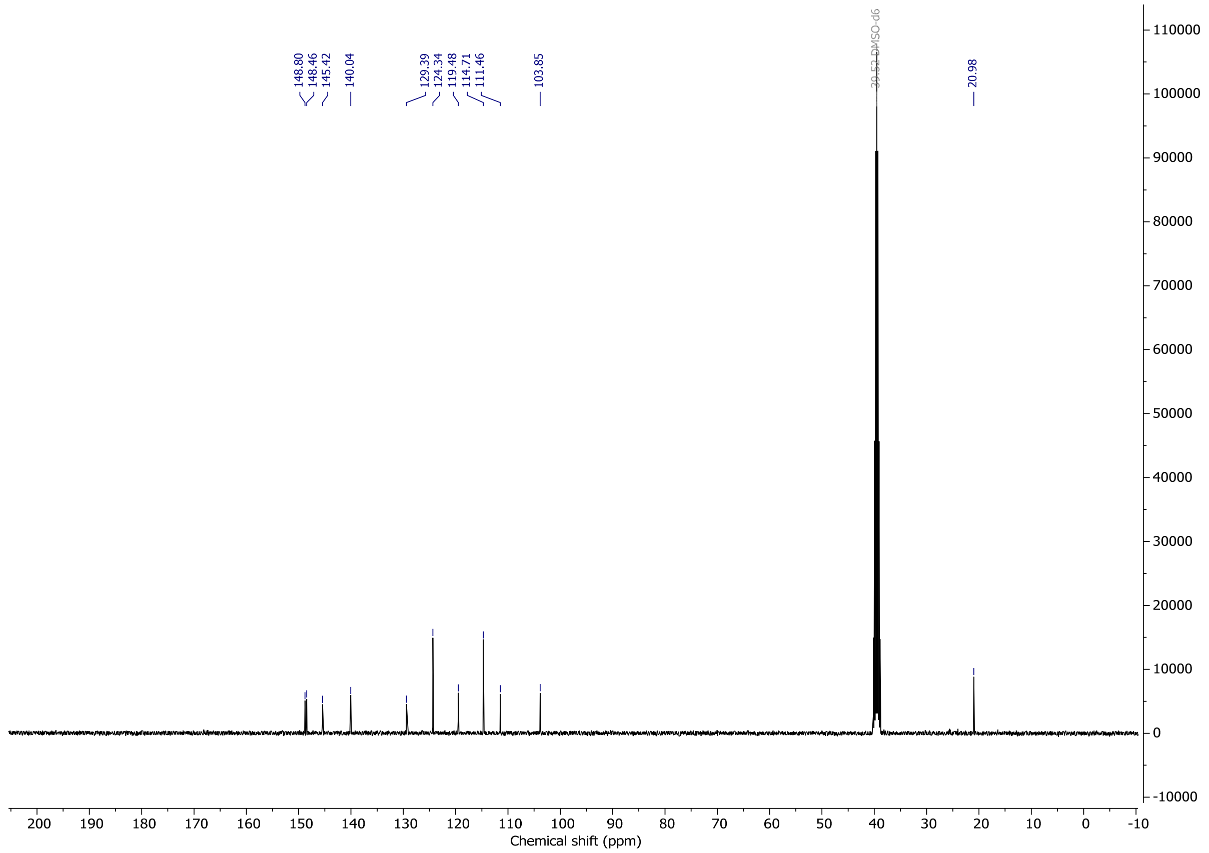

N-(4-Nitrophenyl)pyridin-2-amine (67)

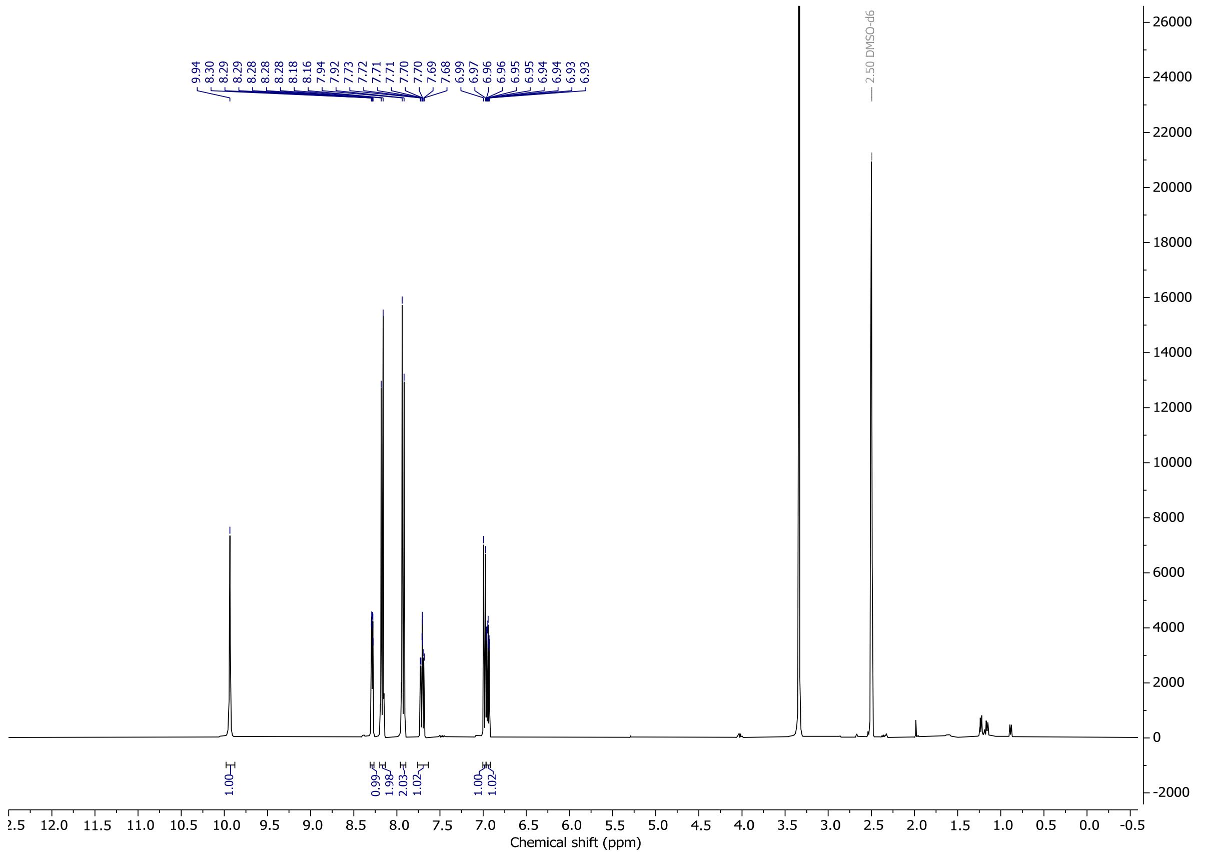

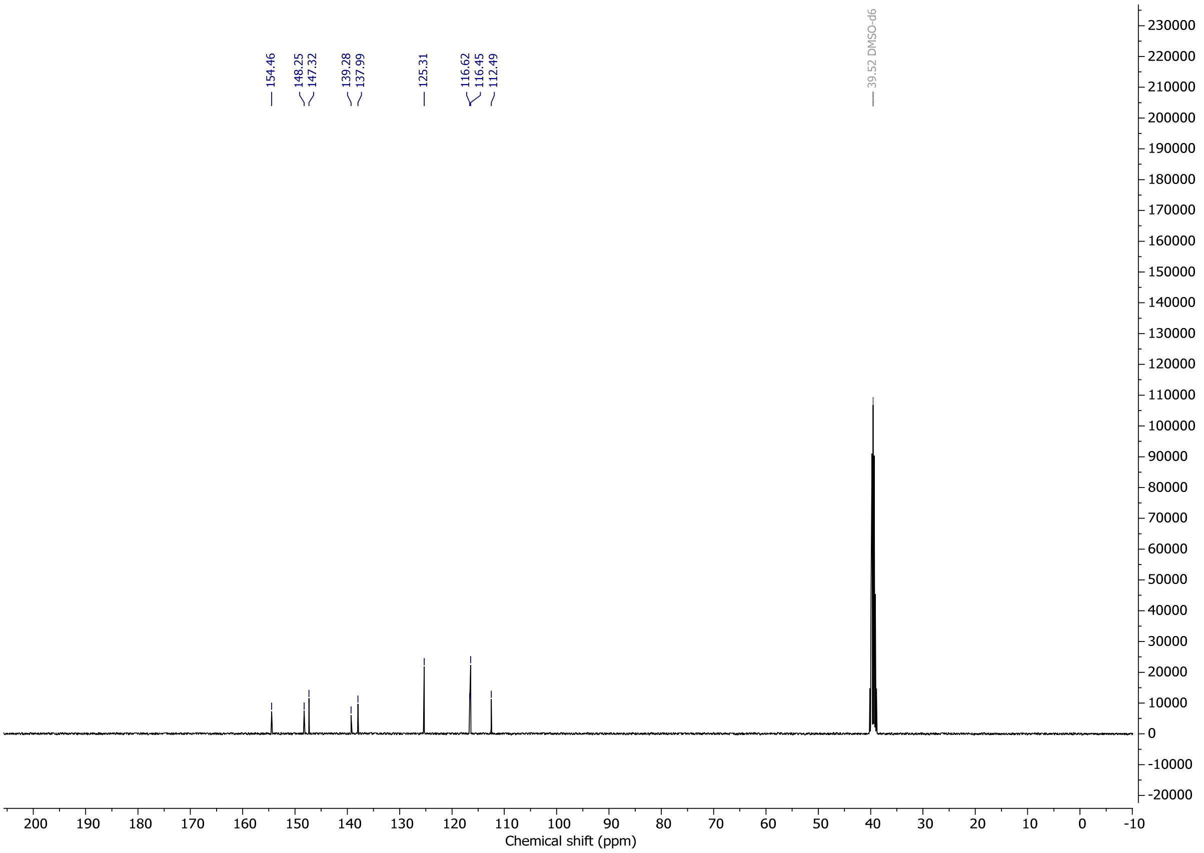

3-((4-Nitrophenyl)amino)phenol (68)

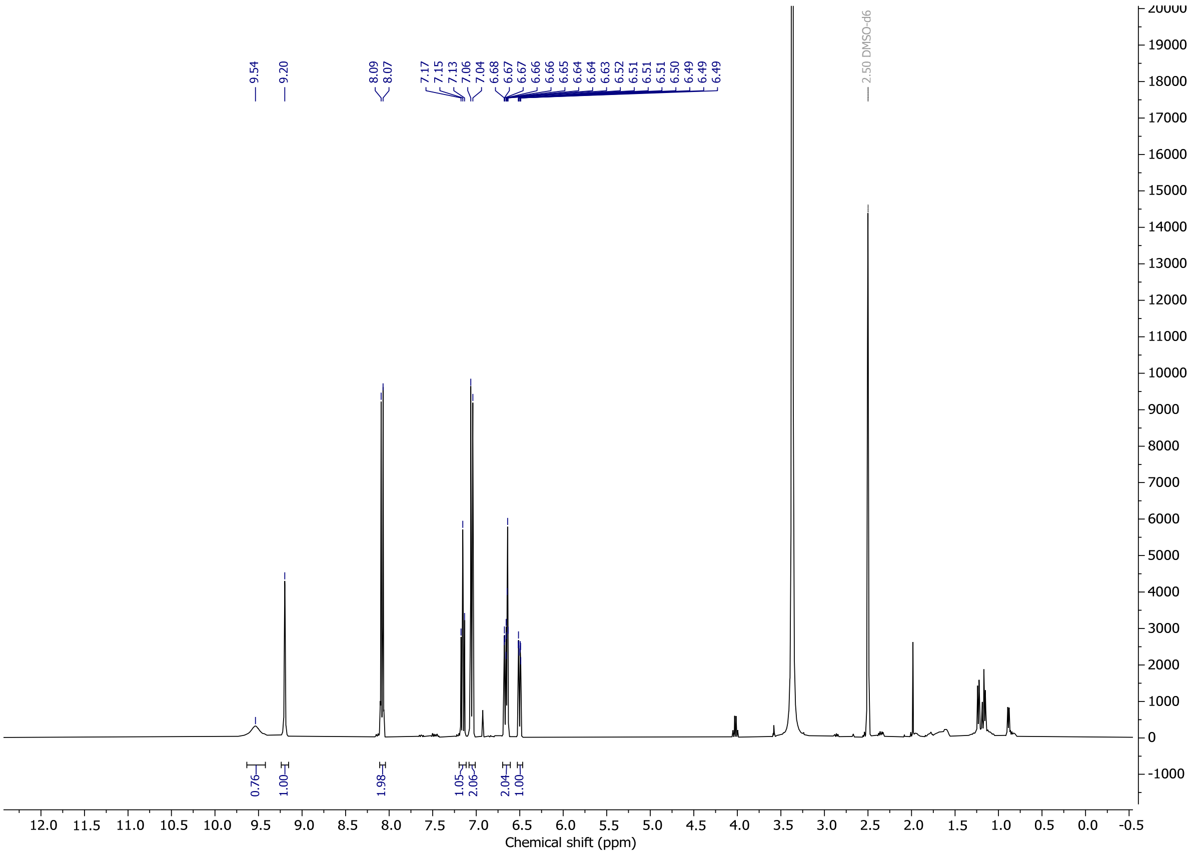

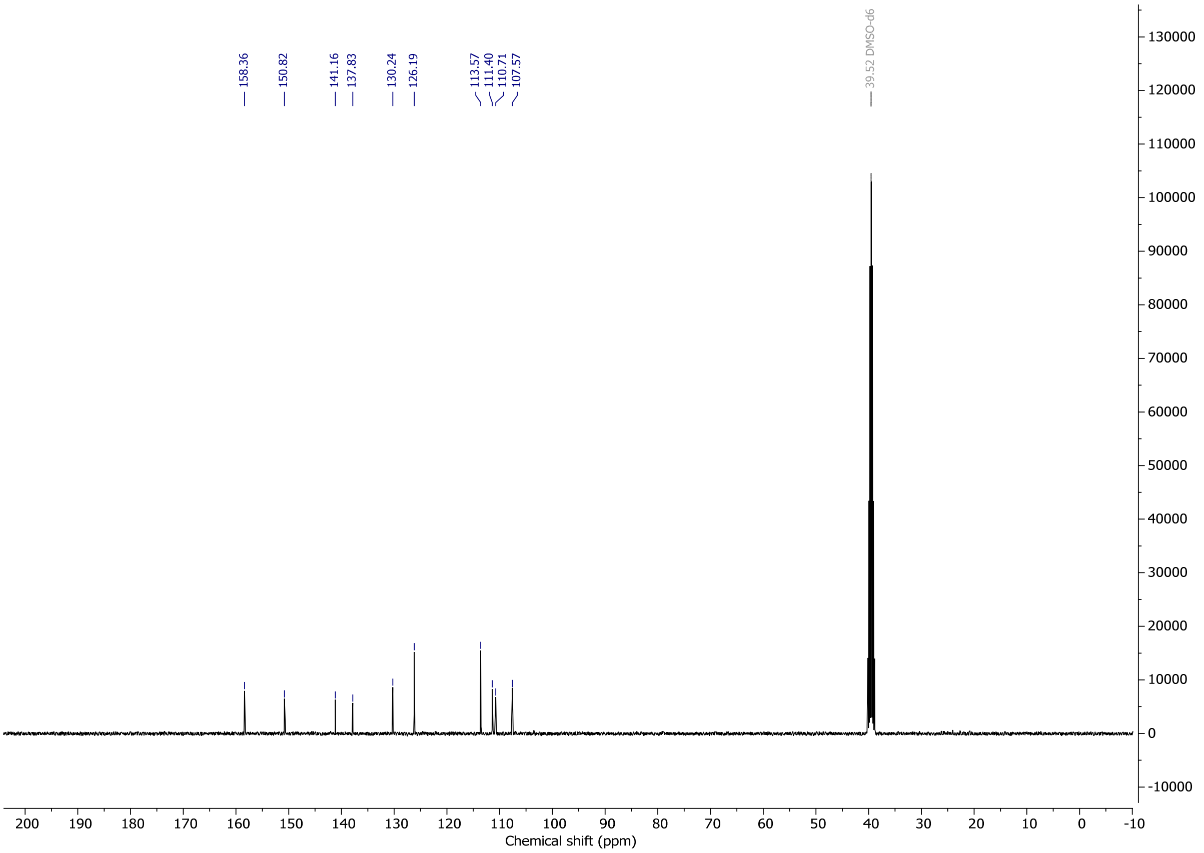

tert-Butyl (4-((2-(ethylamino)pyrimidin-4-yl)amino)phenyl)carbamate (69)

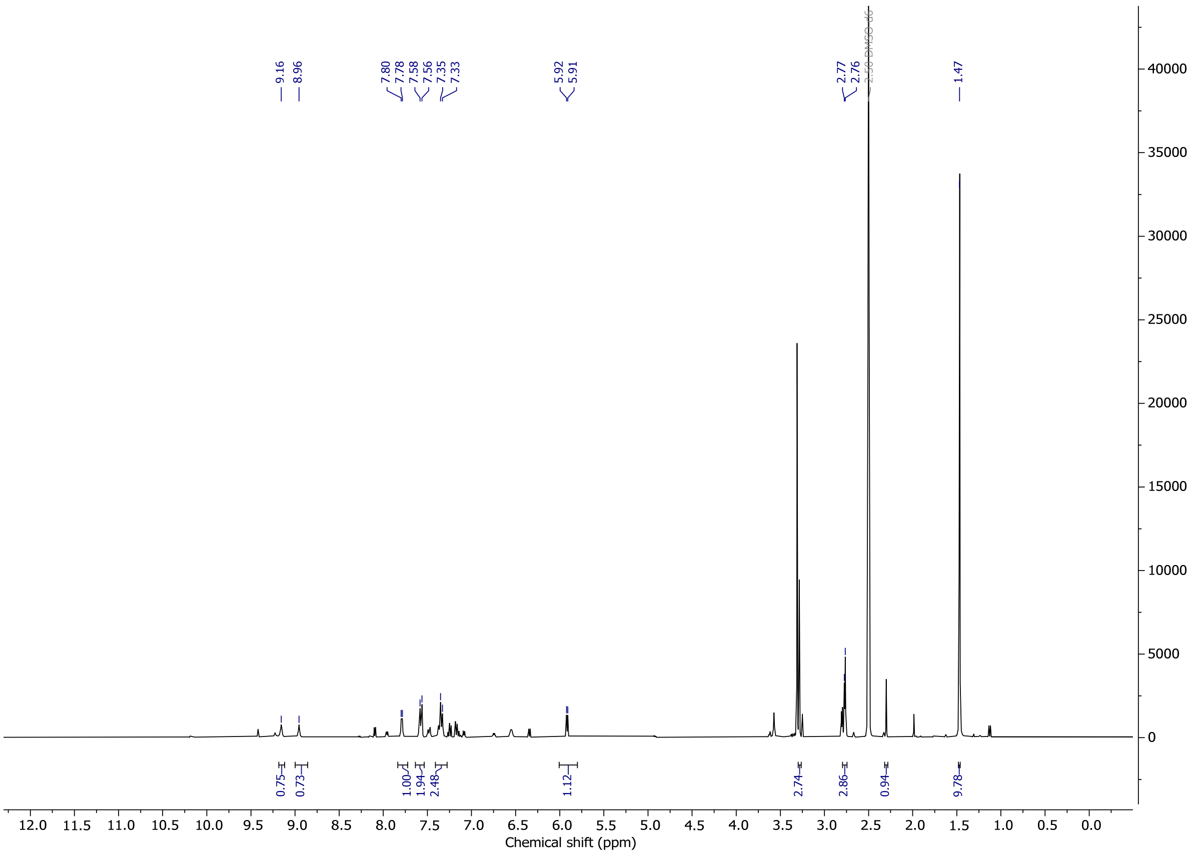

Ethyl 4-(naphthalen-1-ylamino)benzoate (70)

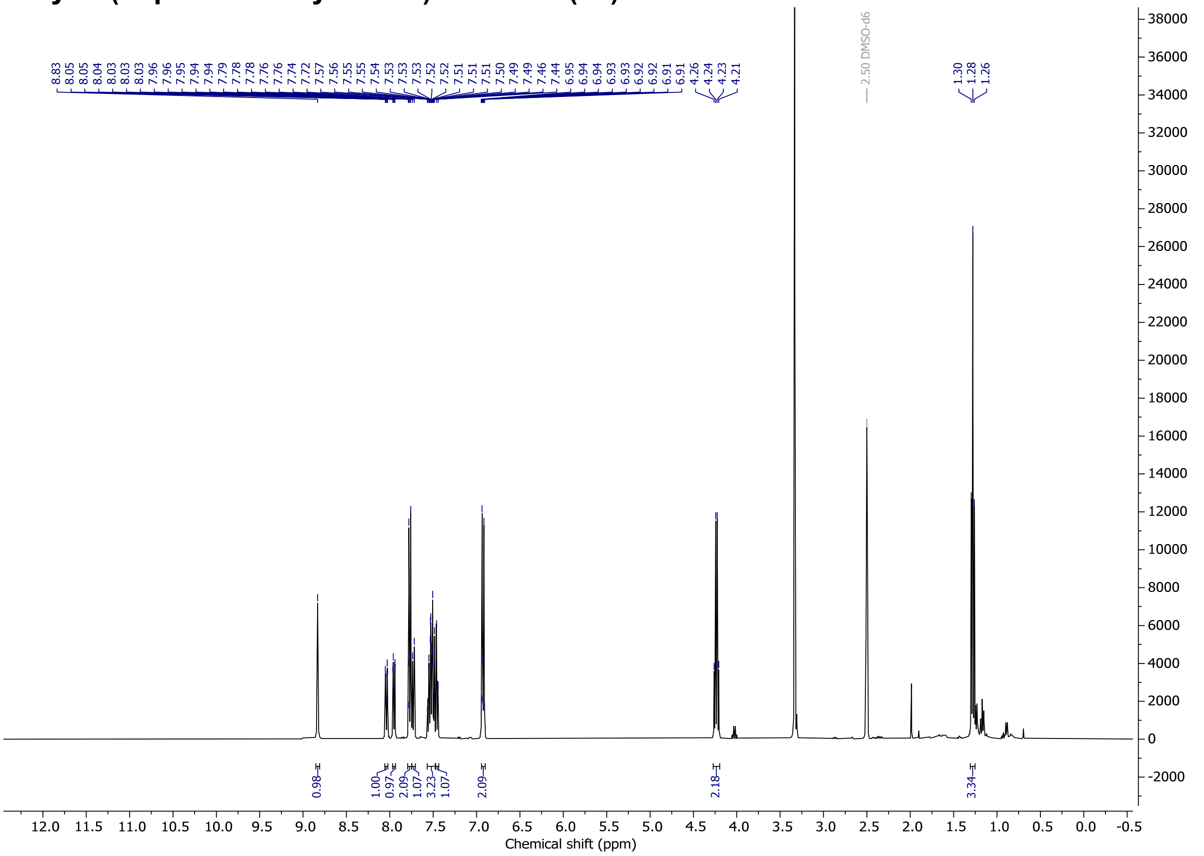

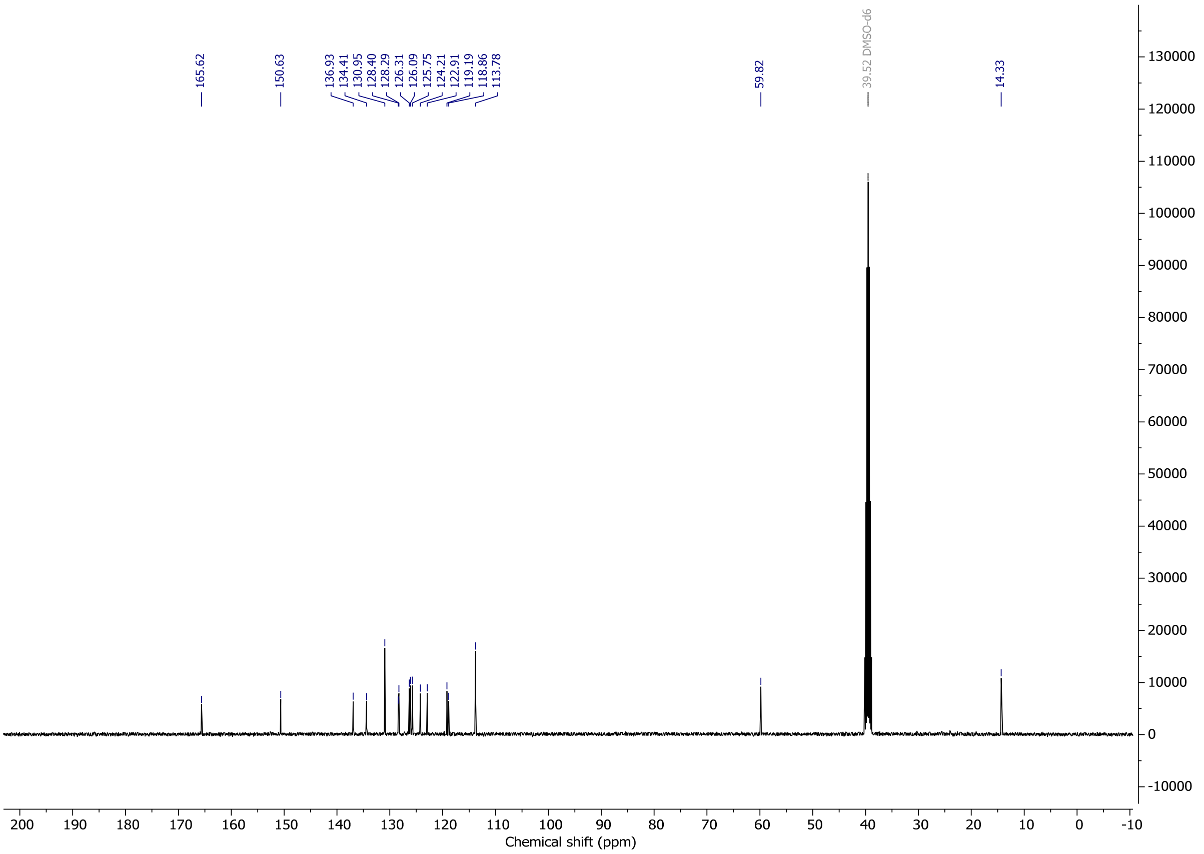

Ethyl 4-(quinazolin-4-ylamino)benzoate (71)

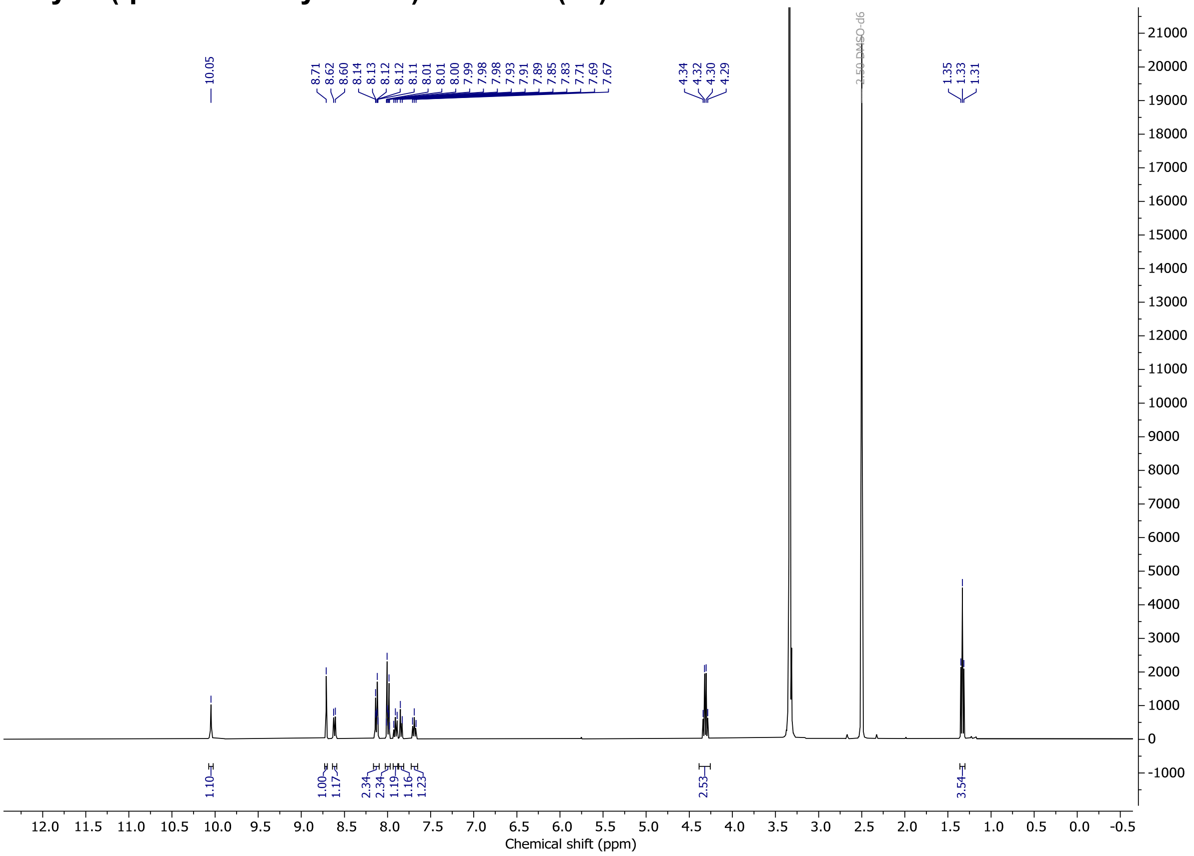

Ethyl 4-((3a,7a-dihydro-1H-pyrrolo[2,3-b]pyridin-4-yl)amino)benzoate (72)

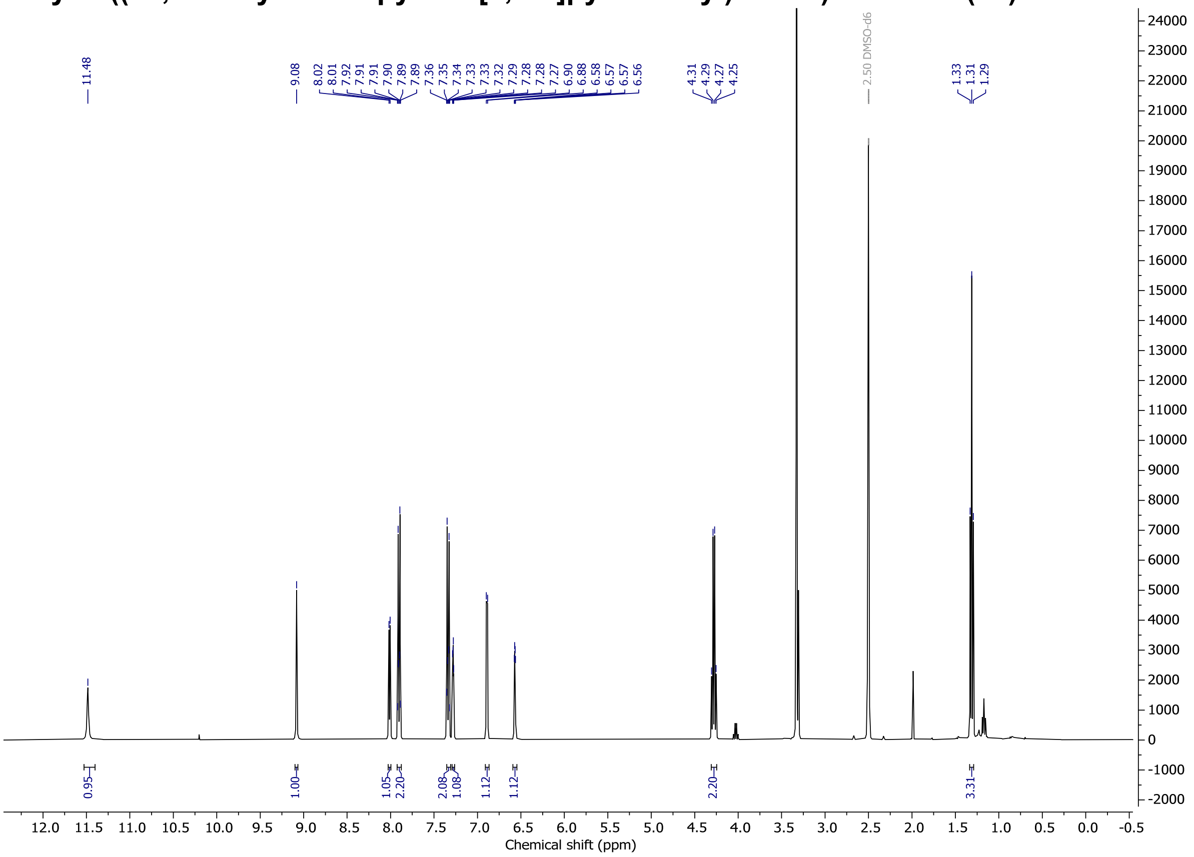

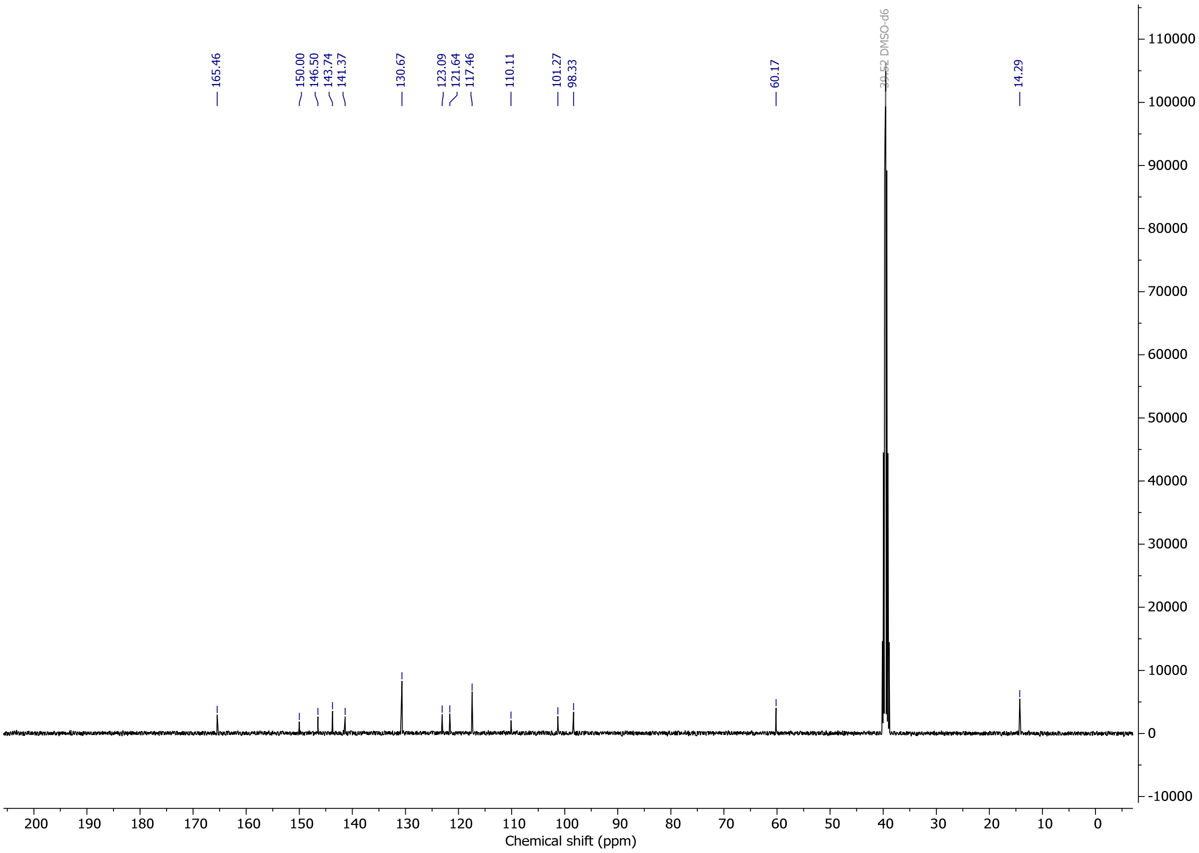

N^4-(4-Aminophenyl)-6-methylpyrimidine-2,4-diamine (73)

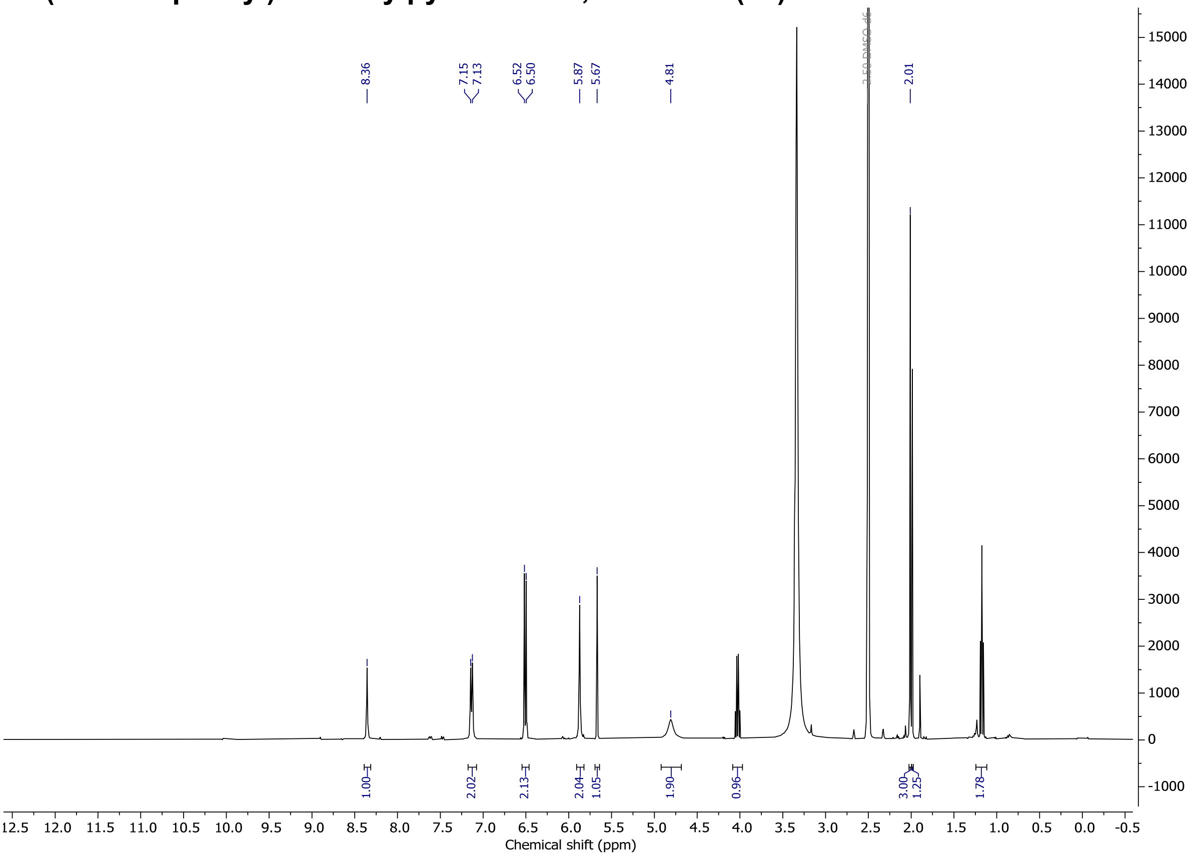

N^4-(4-Aminophenyl)pyrimidine-2,4-diamine (74)

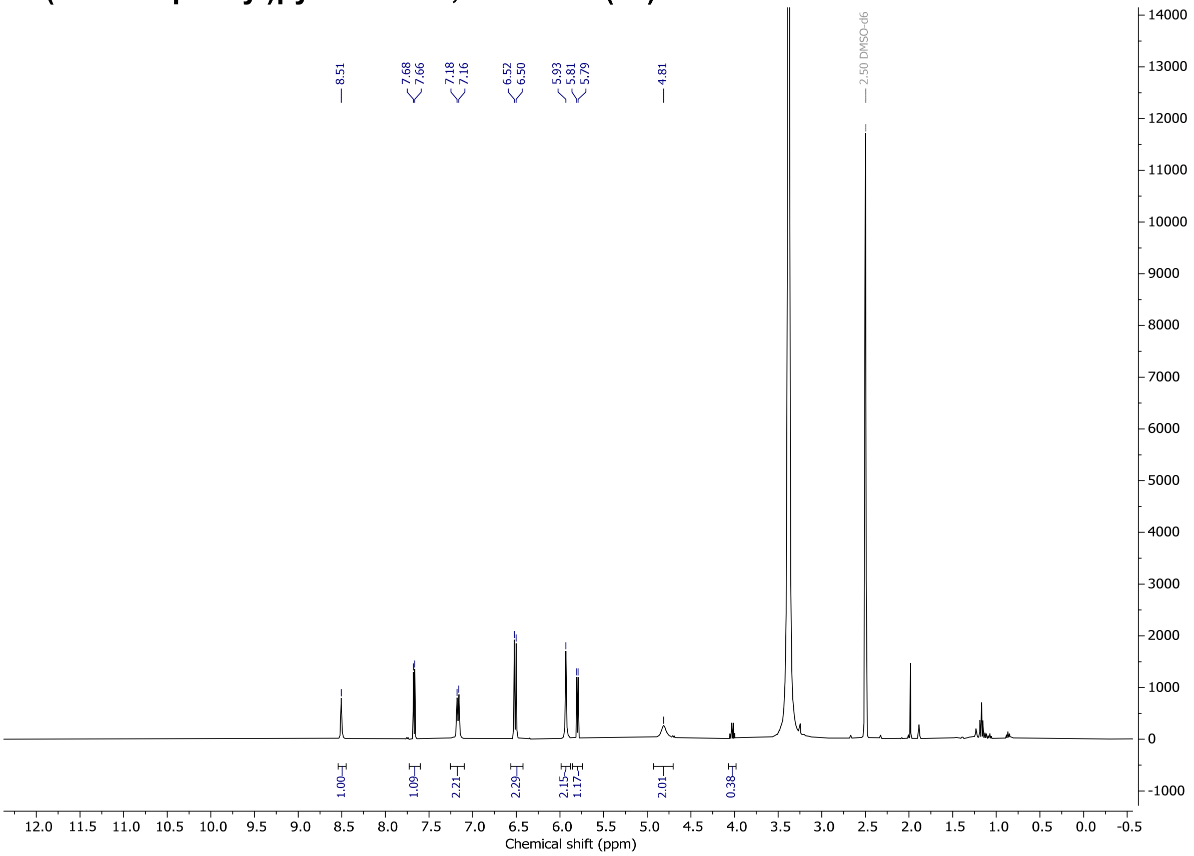

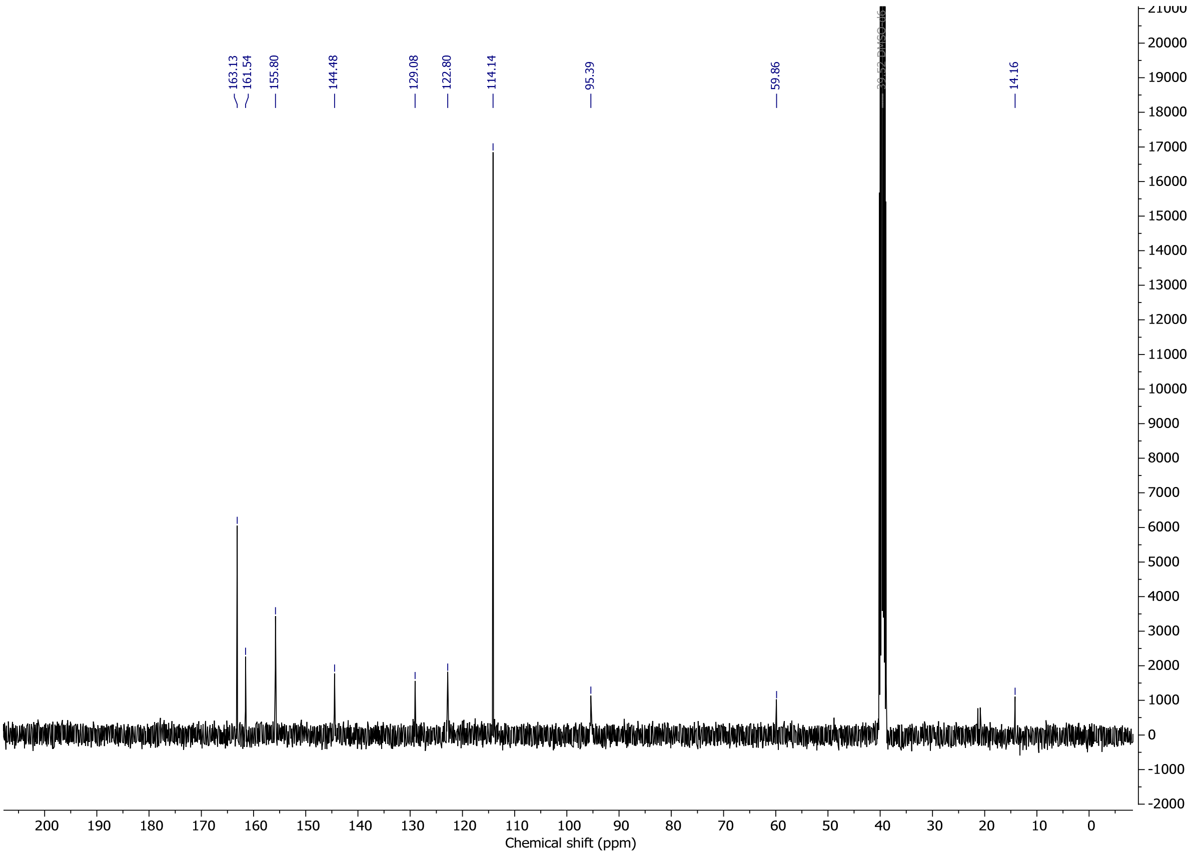

N^1-(5-(Trifluoromethyl)pyridin-2-yl)benzene-1,4-diamine (75)

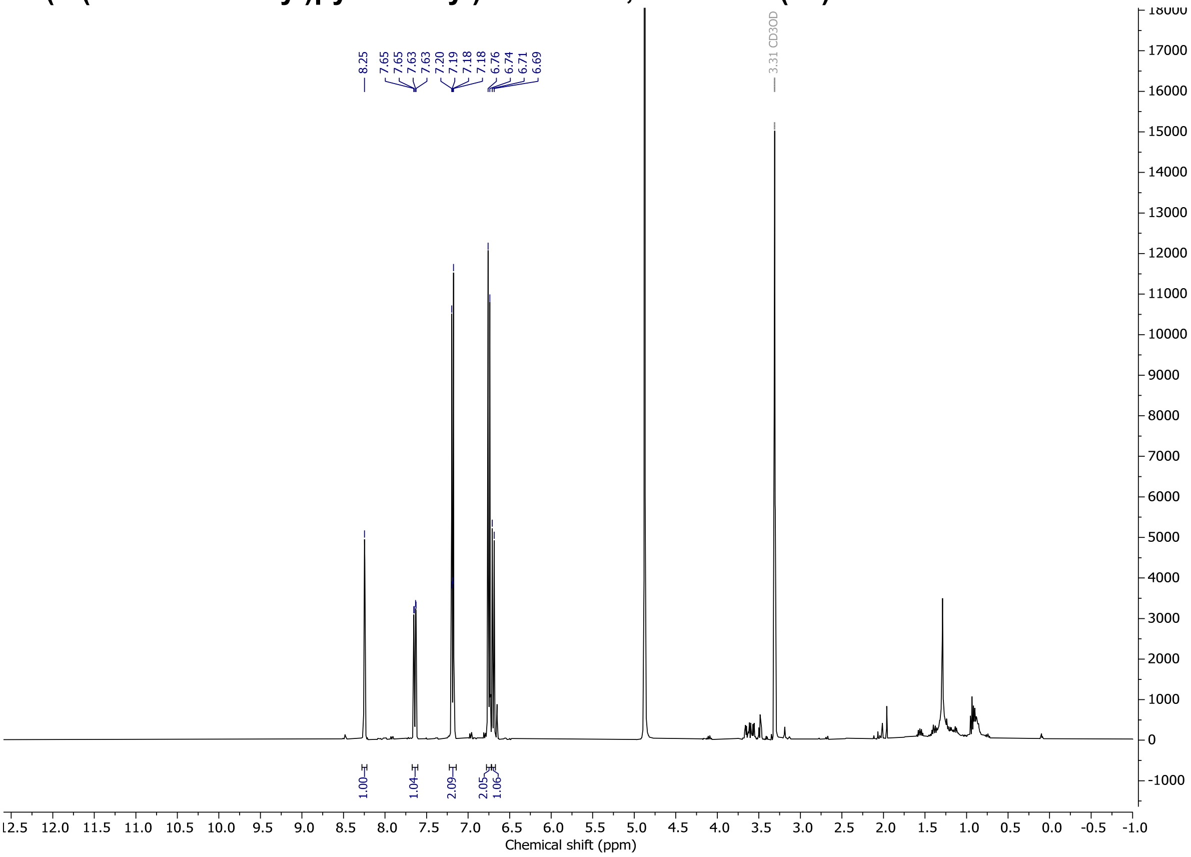

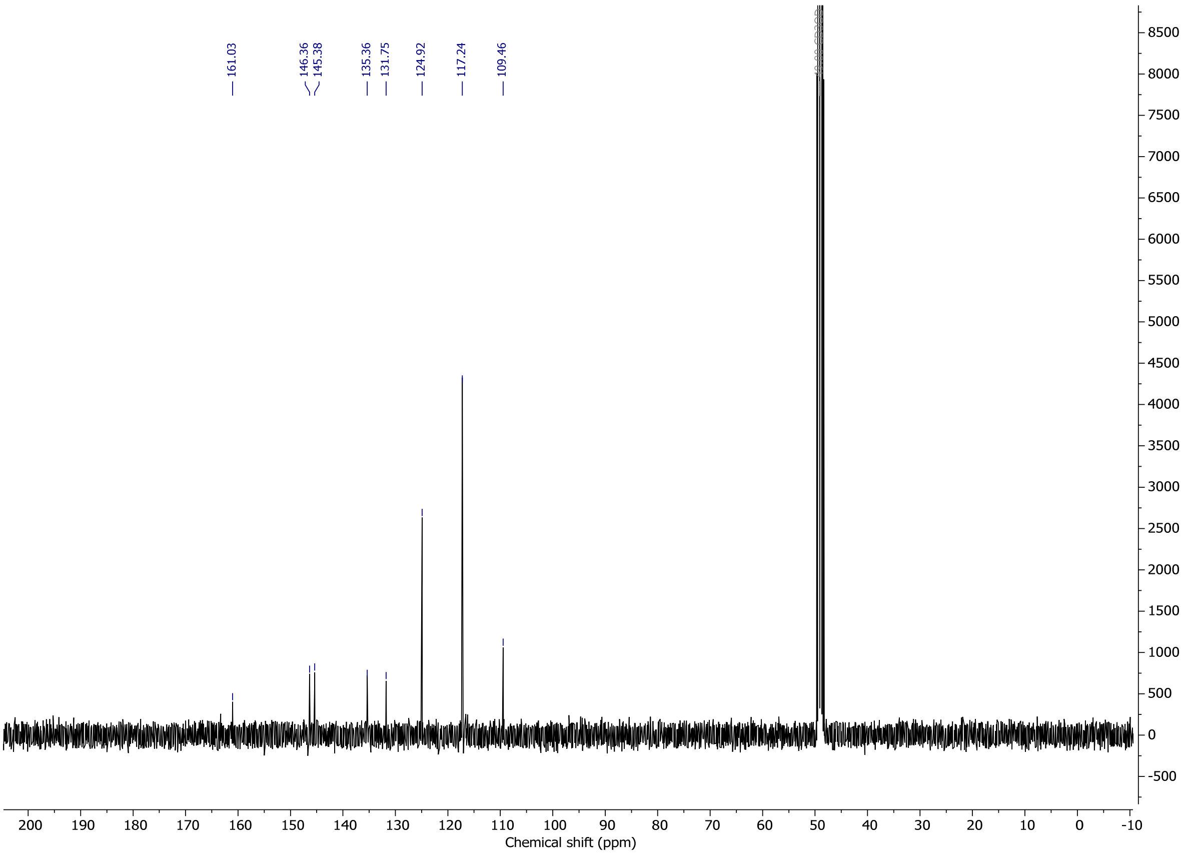

N^1-(4-(Trifluoromethyl)phenyl)benzene-1,4-diamine (76)

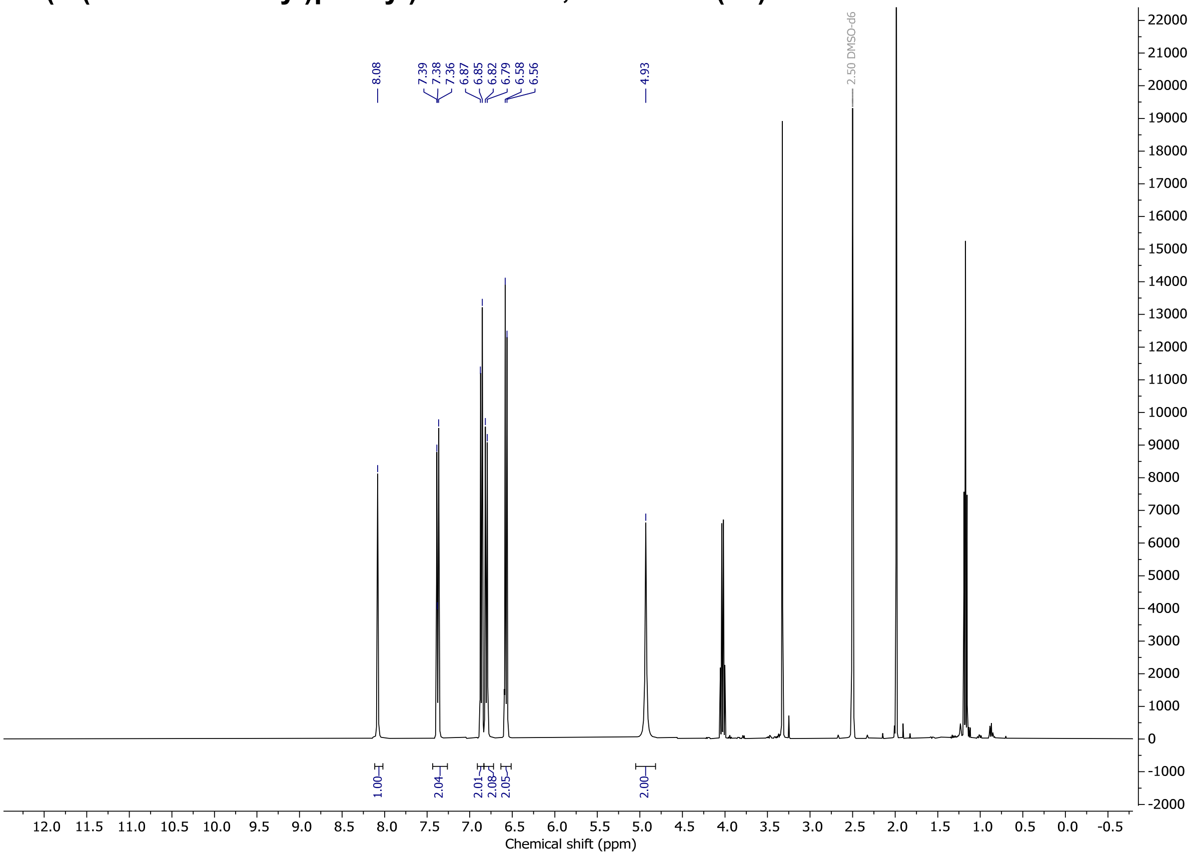

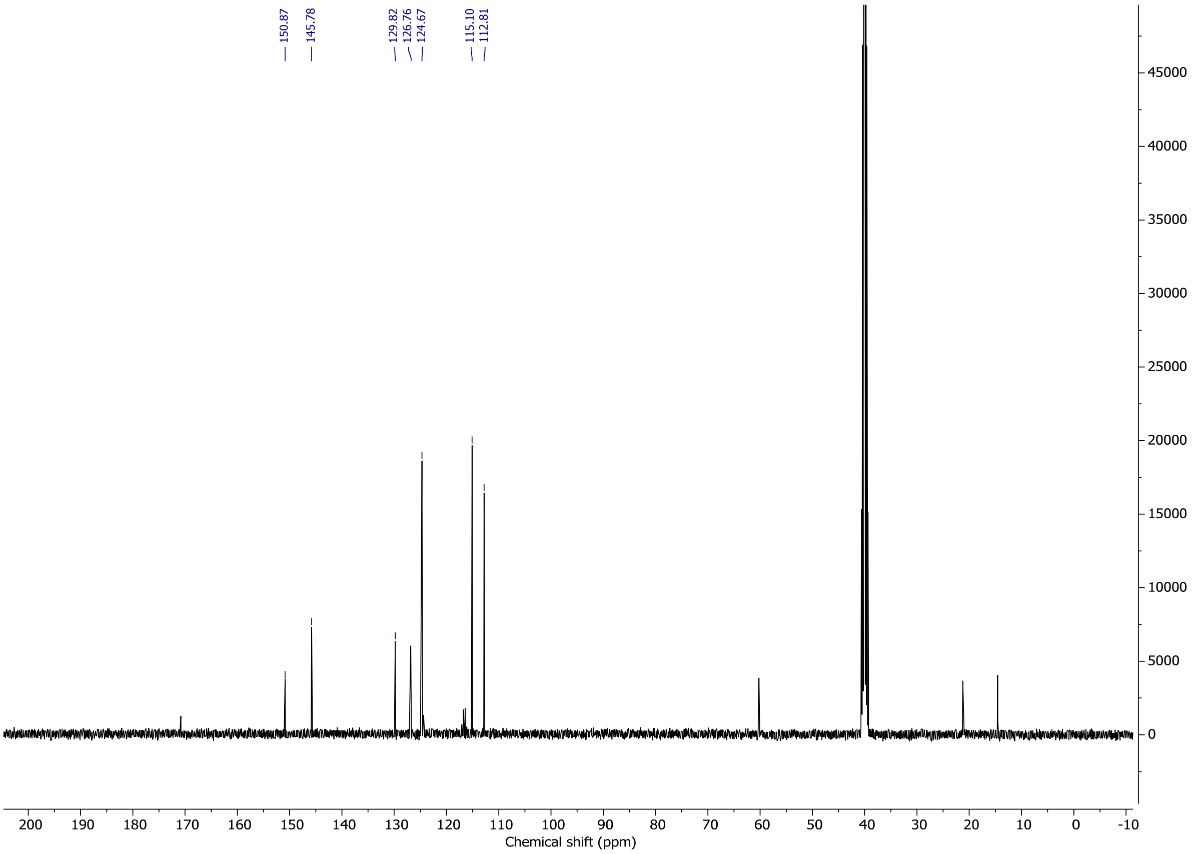

N^1-(2-(Trifluoromethyl)phenyl)benzene-1,4-diamine (77)

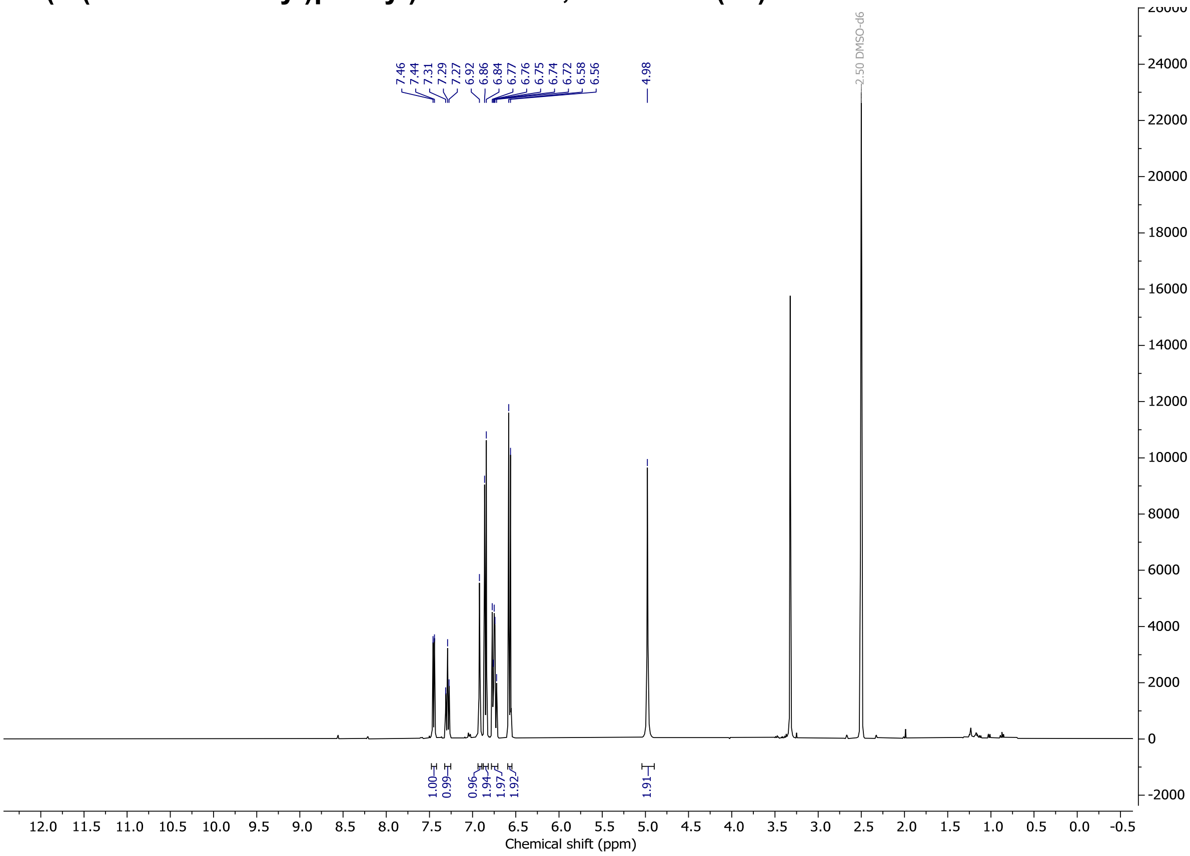

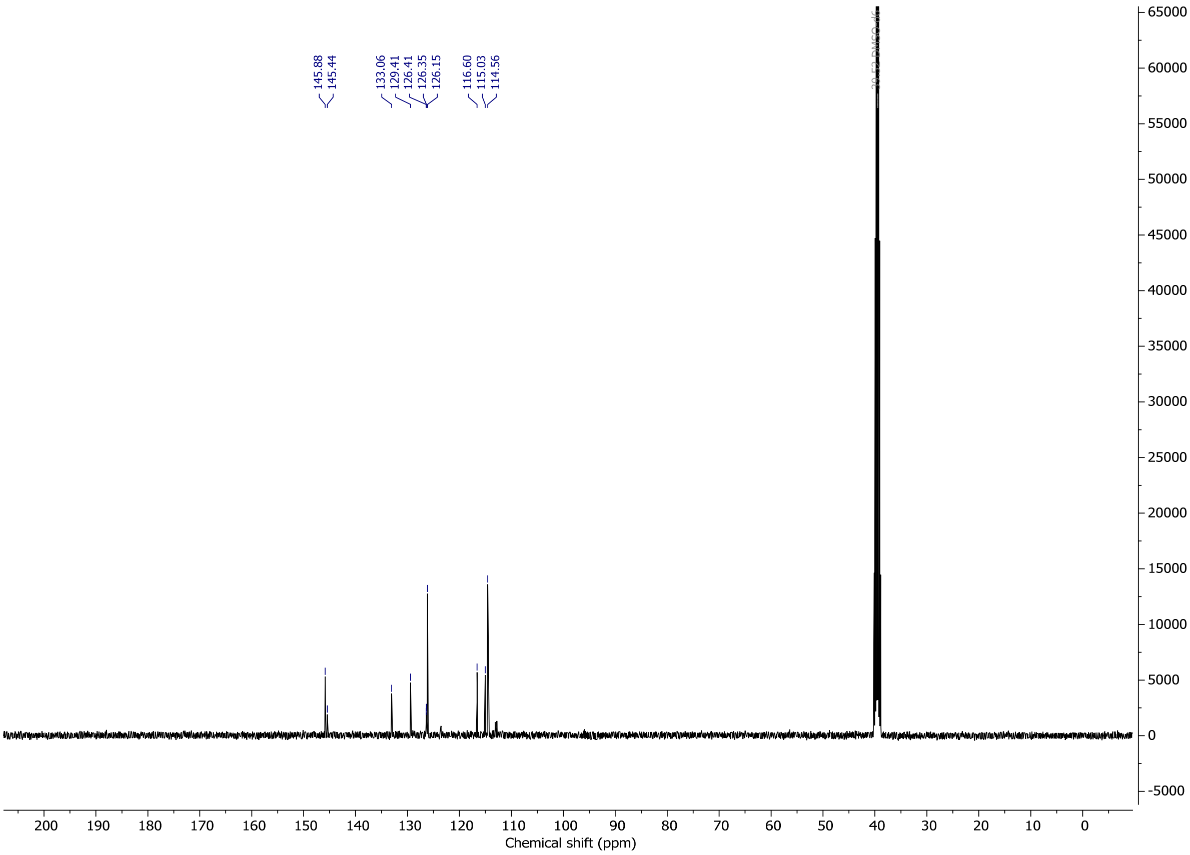

N^1-(*p*-tolyl)benzene-1,4-diamine (78)

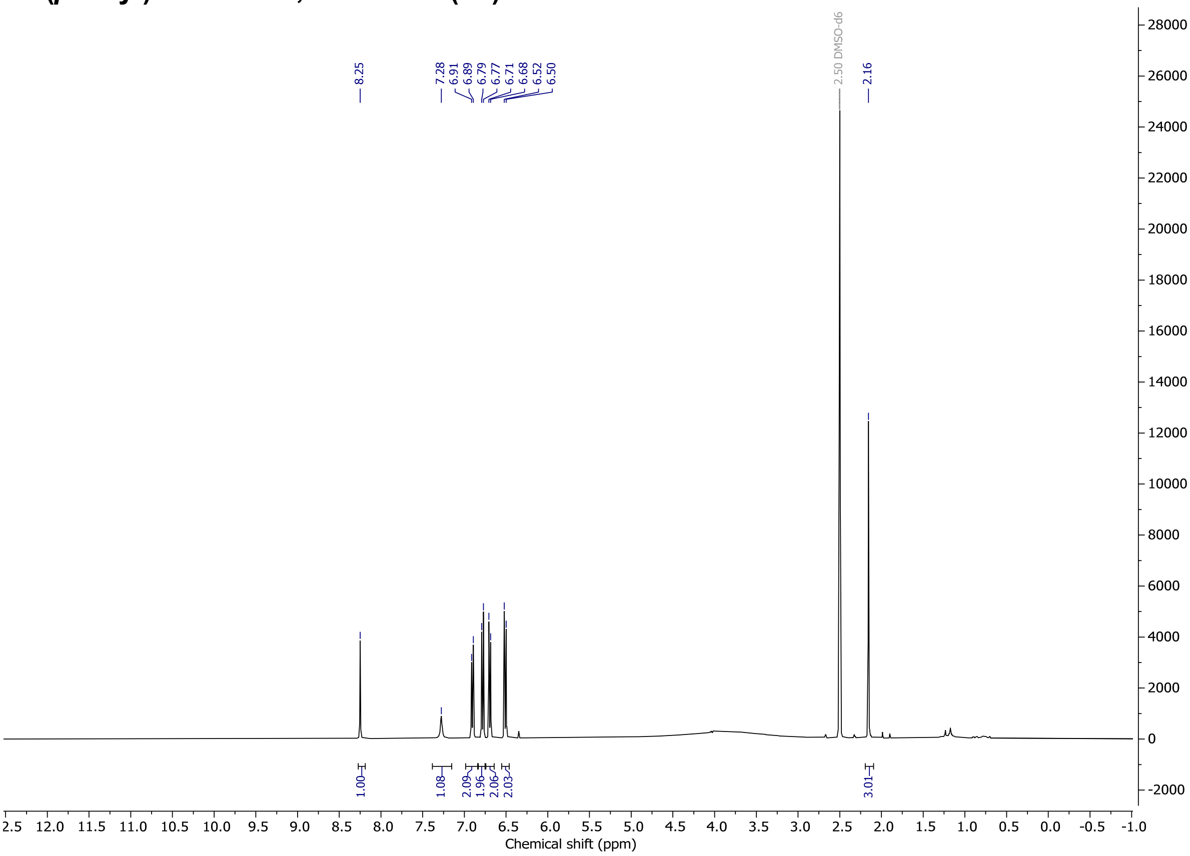

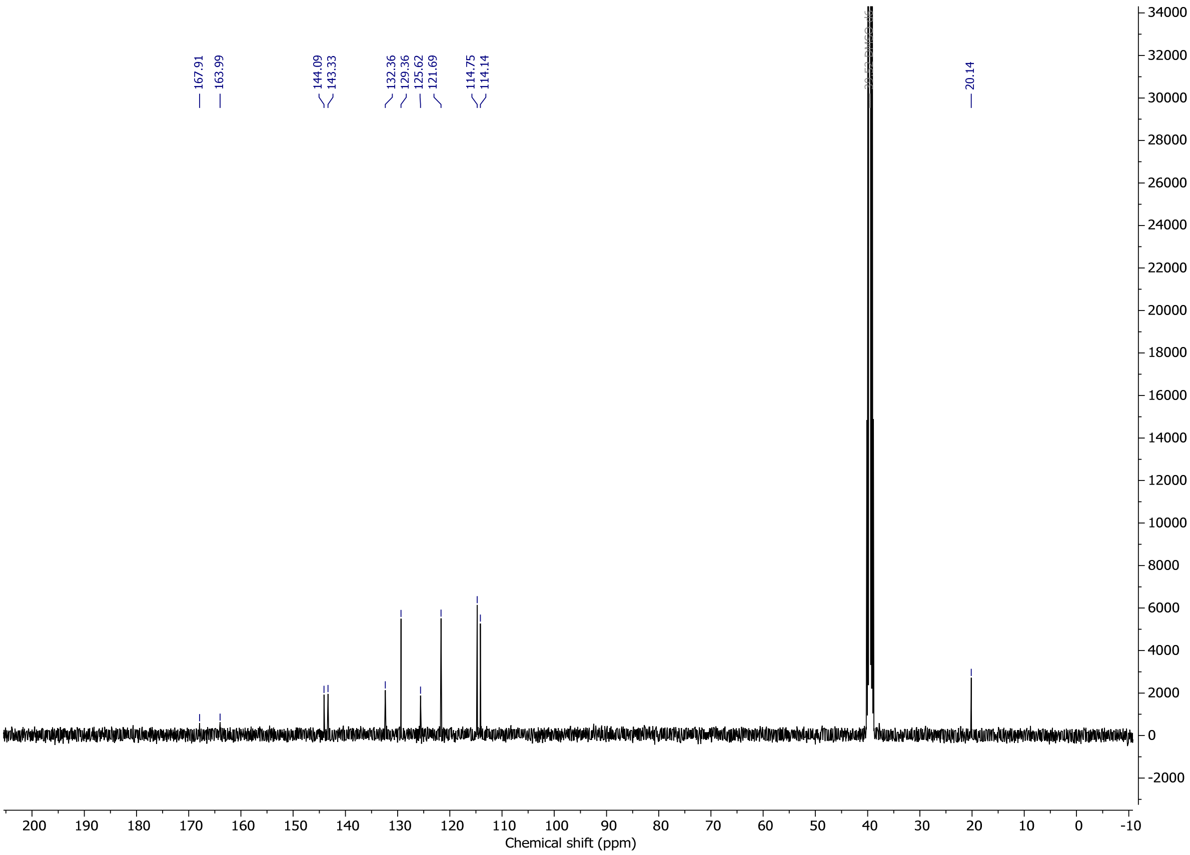

N[1]-Phenylbenzene-1,4-diamine (79)

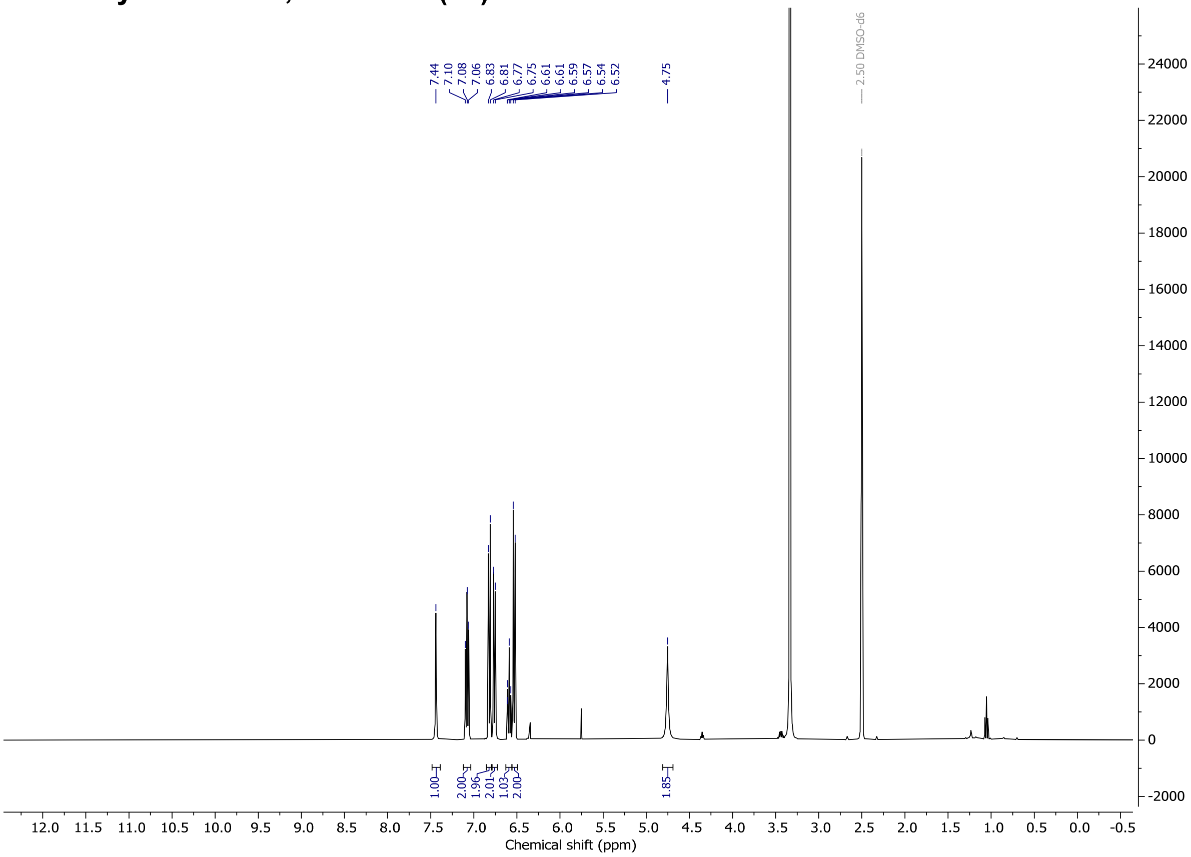

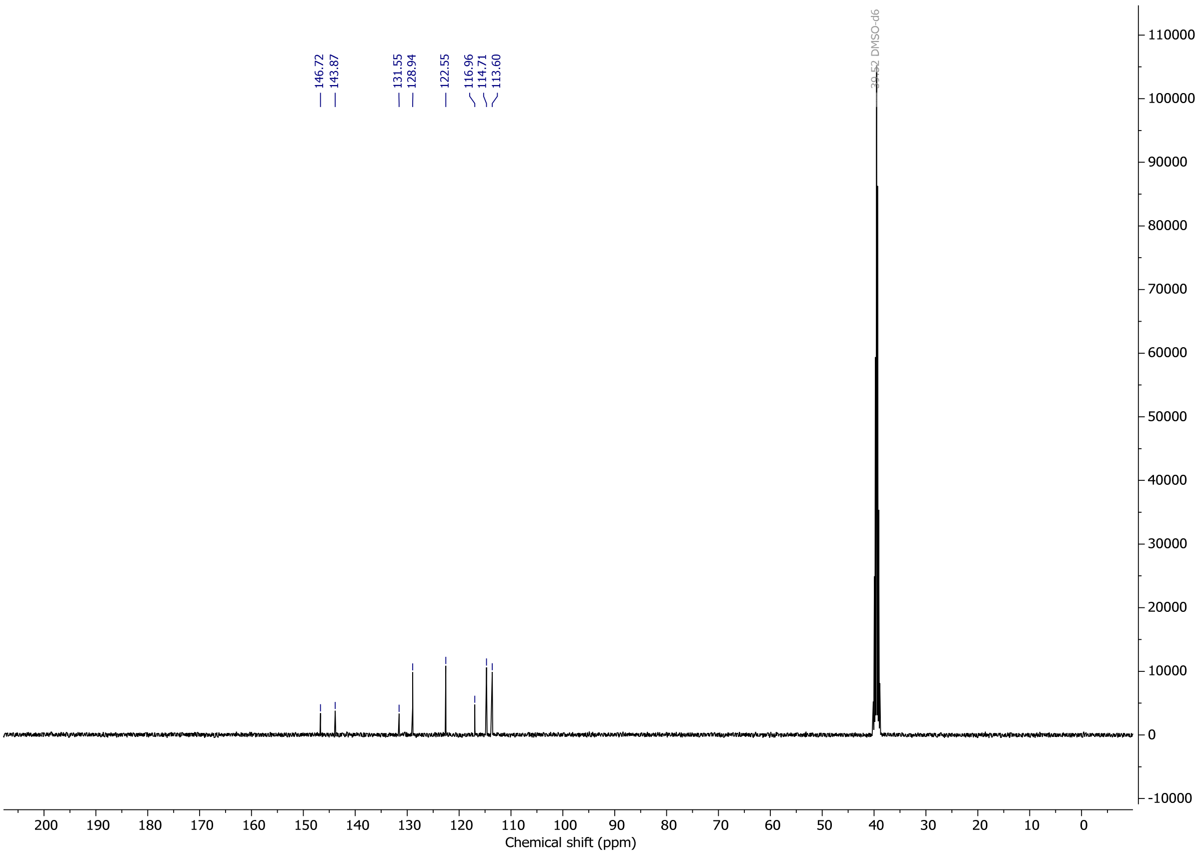

N^1-(4-Methoxyphenyl)benzene-1,4-diamine (80)

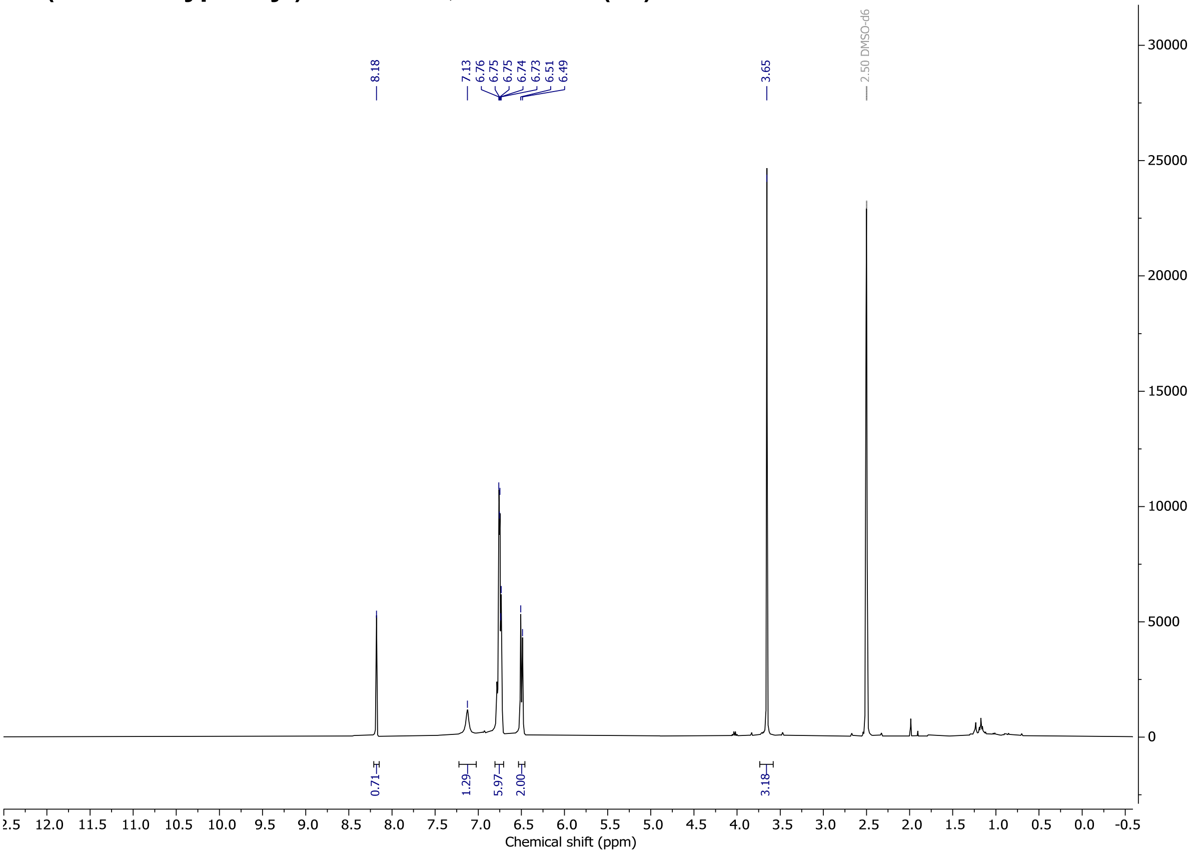

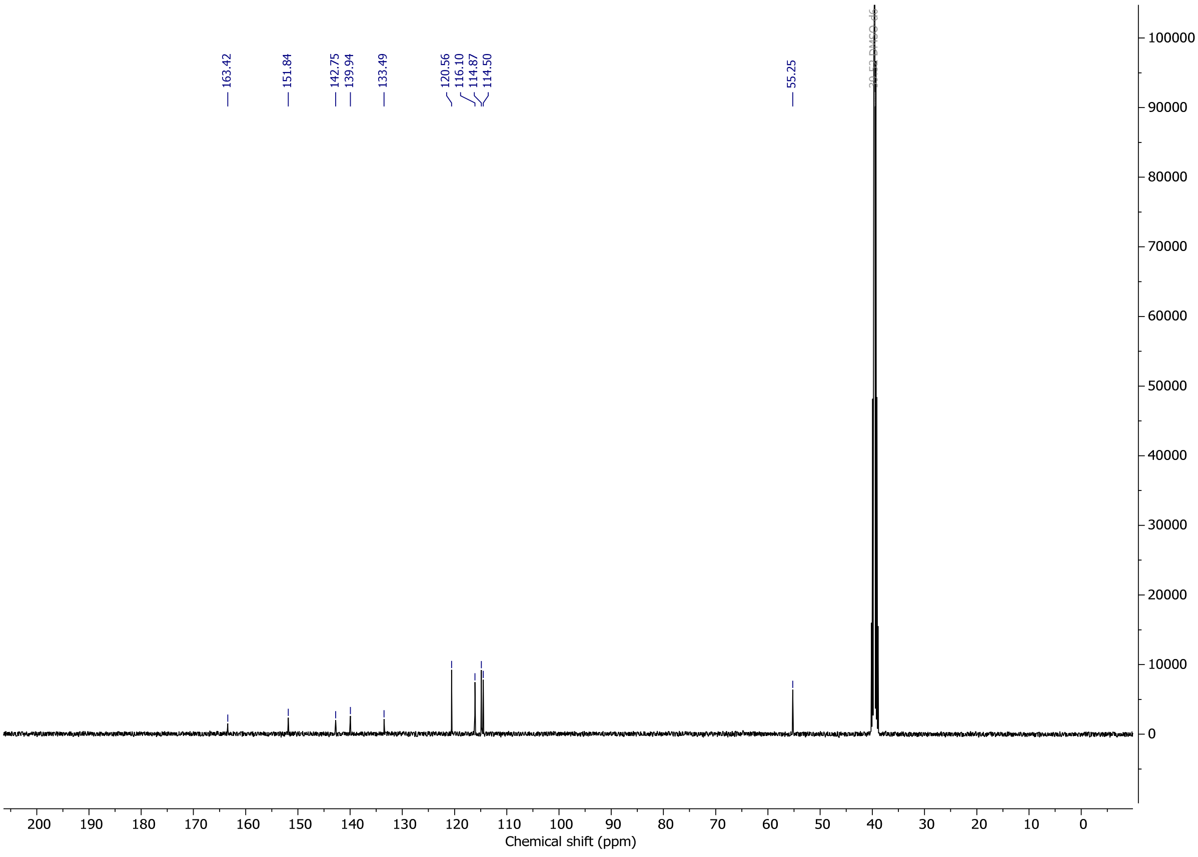

N[1]-(4-Fluorophenyl)benzene-1,4-diamine (81)

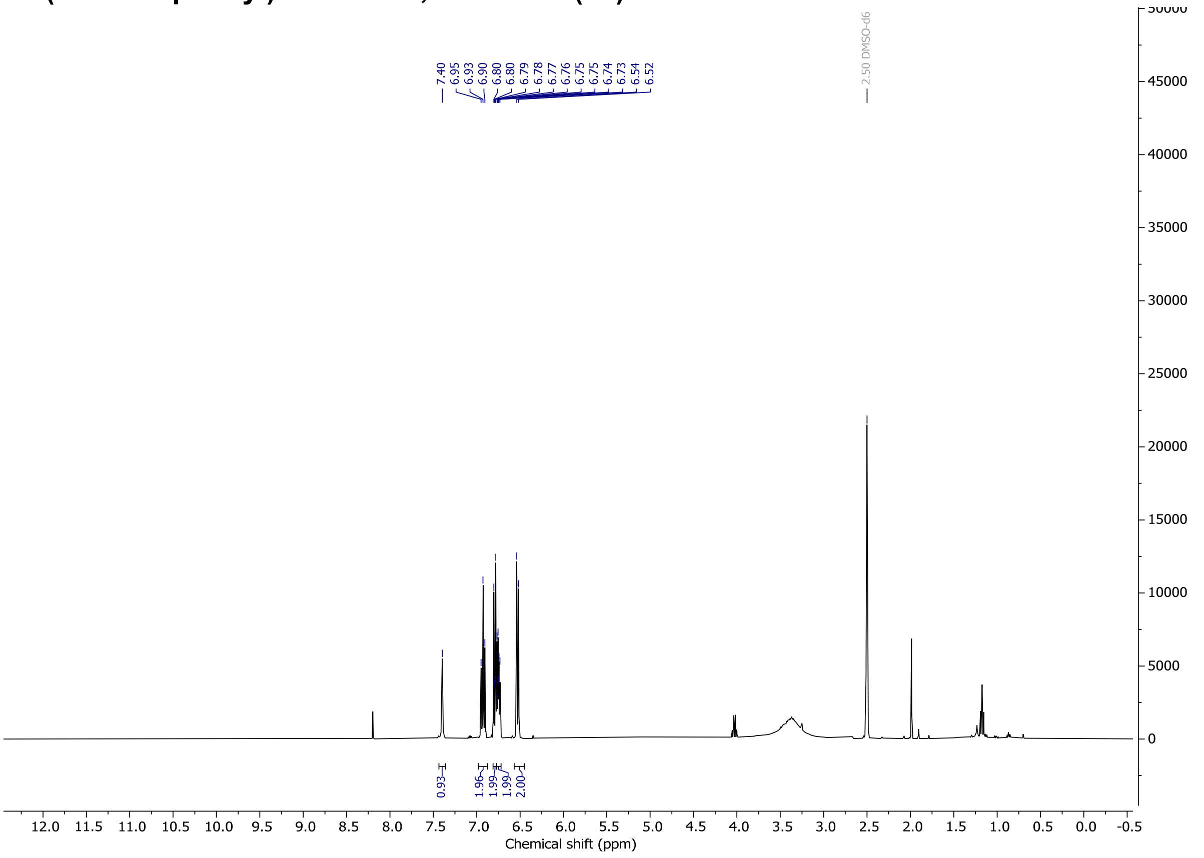

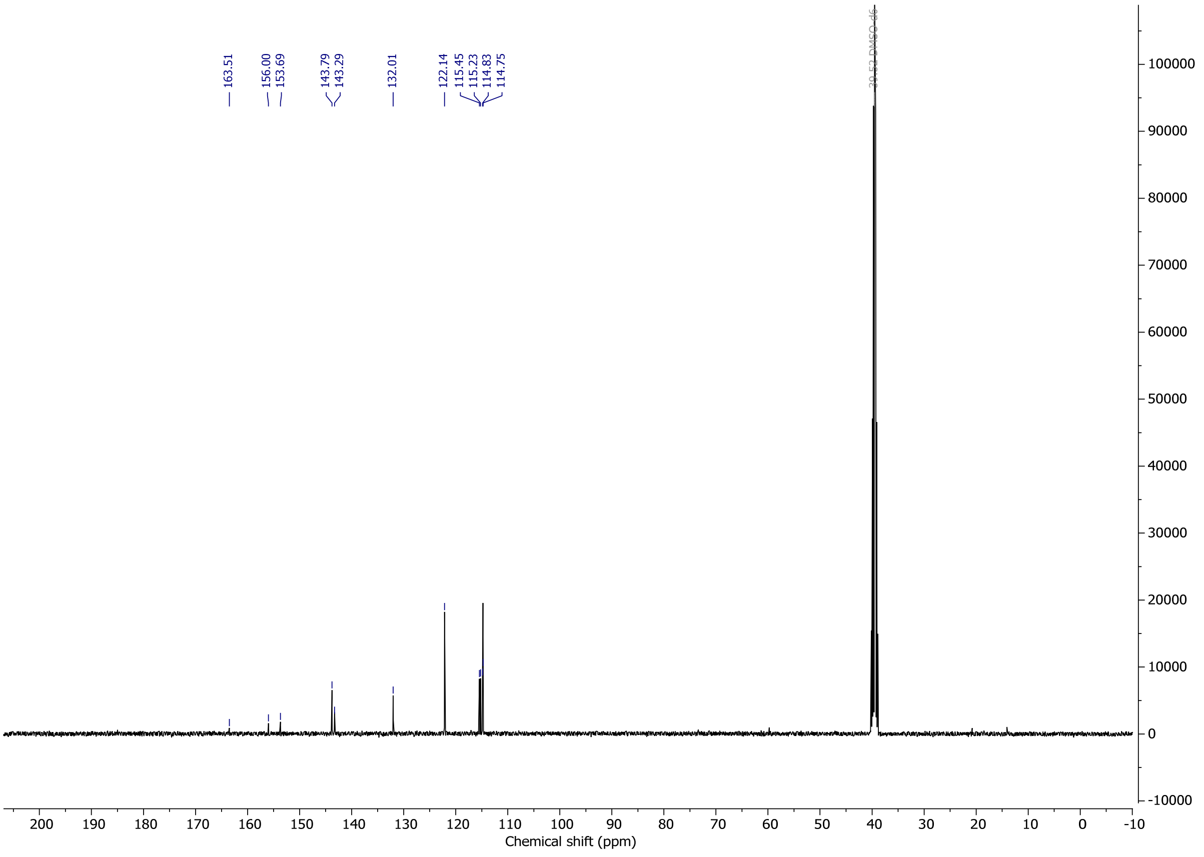

N^2-(4-Aminophenyl)pyridine-2,6-diamine (82)

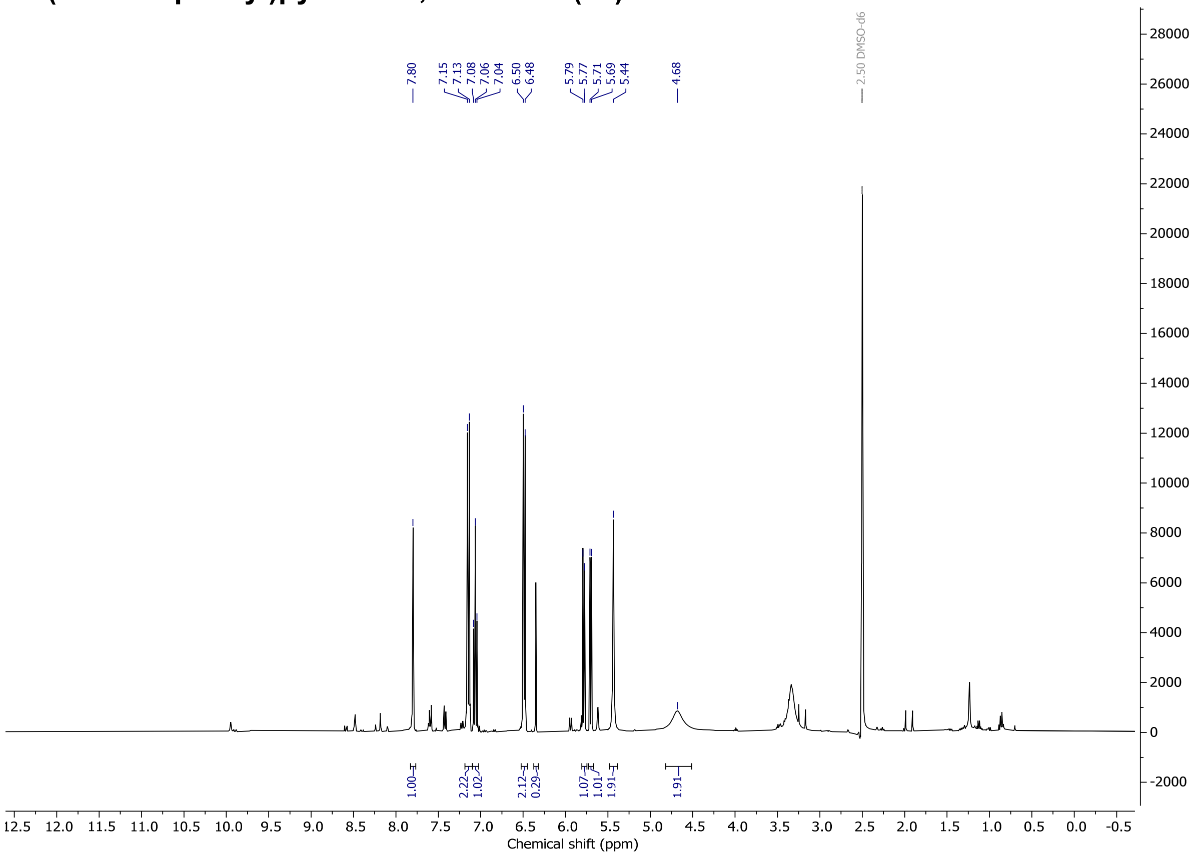

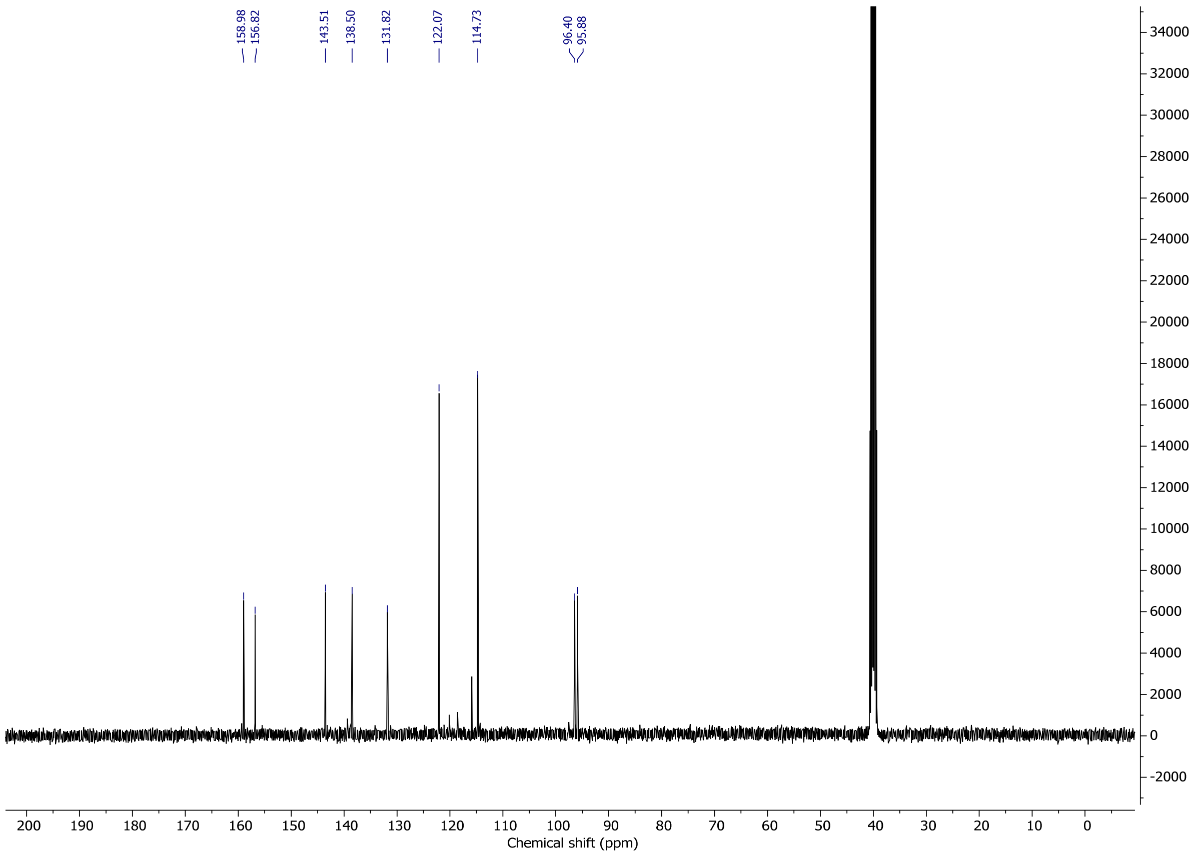

N^4-(4-Aminophenyl)pyridine-2,4-diamine (83)

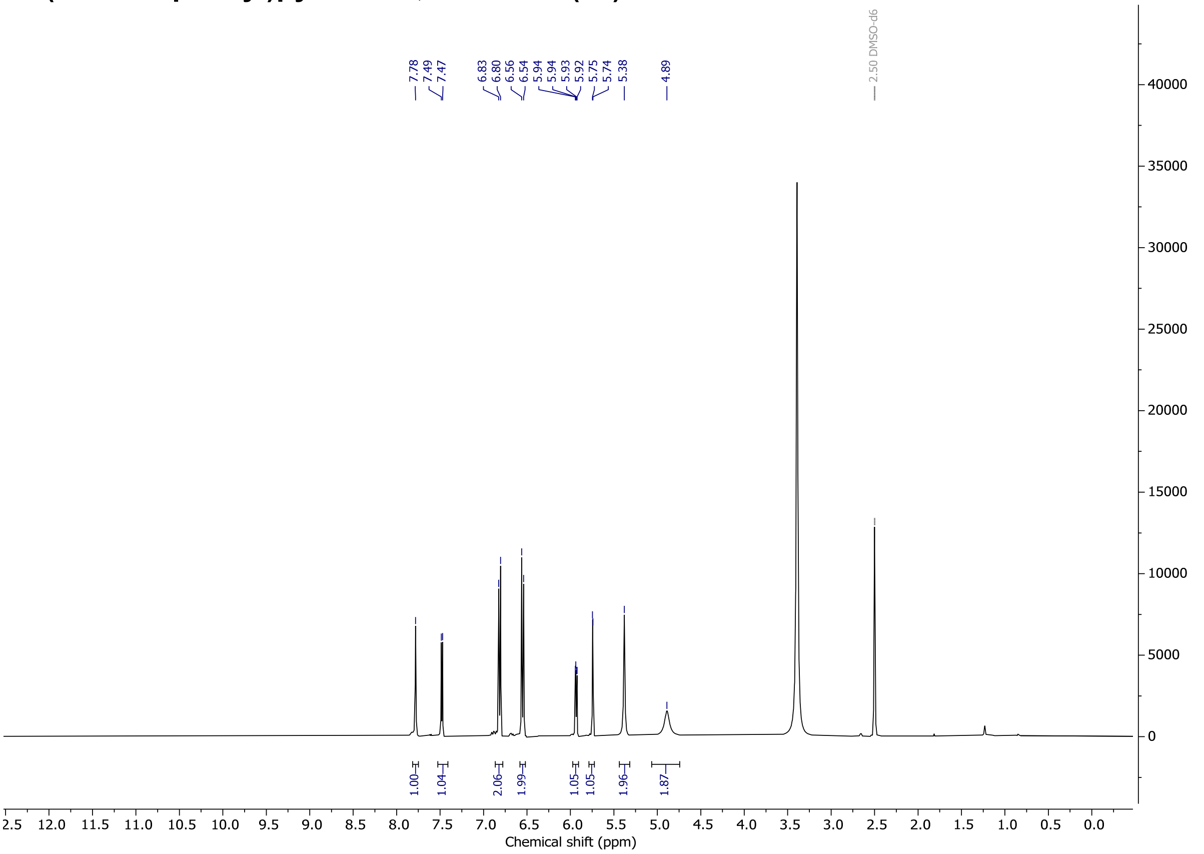

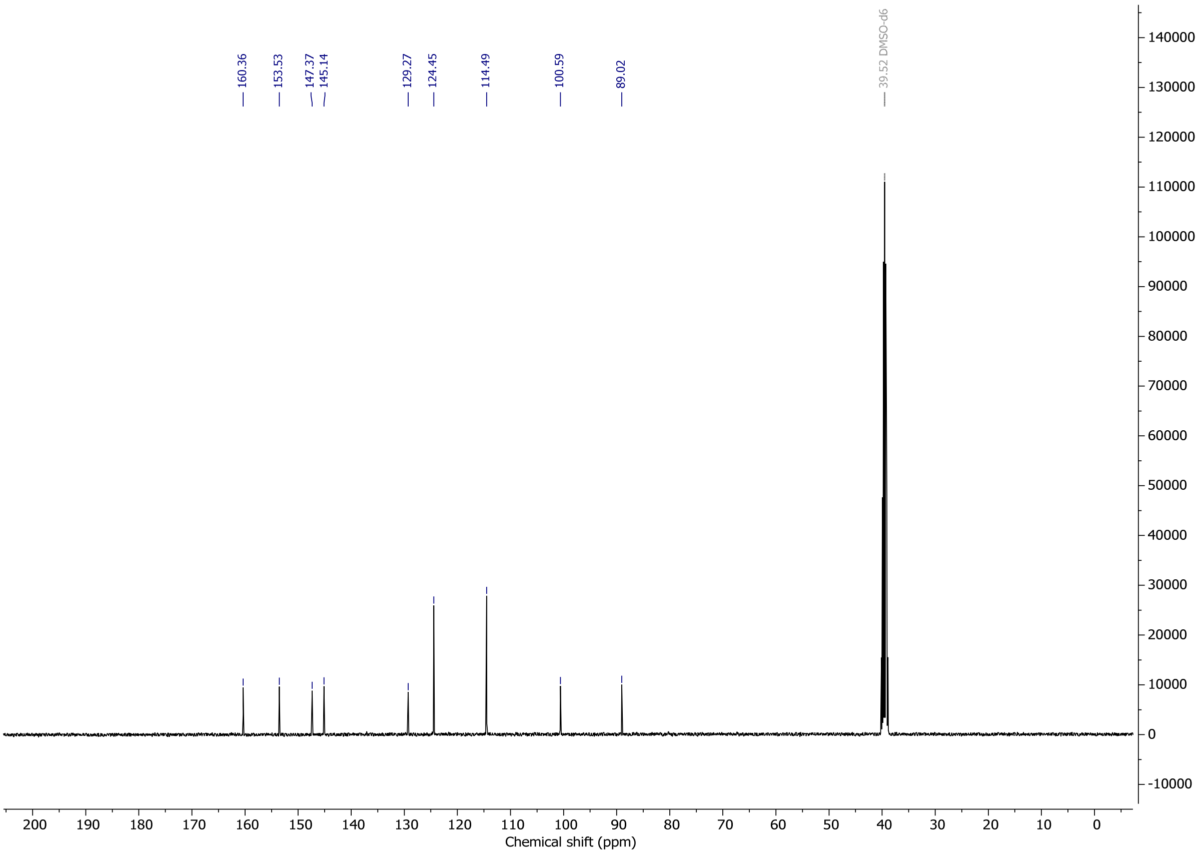

tert-Butyl (5-((4-aminophenyl)amino)-2-fluorophenyl)(tert-butoxycarbonyl)carbamate & *tert*-butyl (5-((4-aminophenyl)amino)-2-fluorophenyl)carbamate (84)

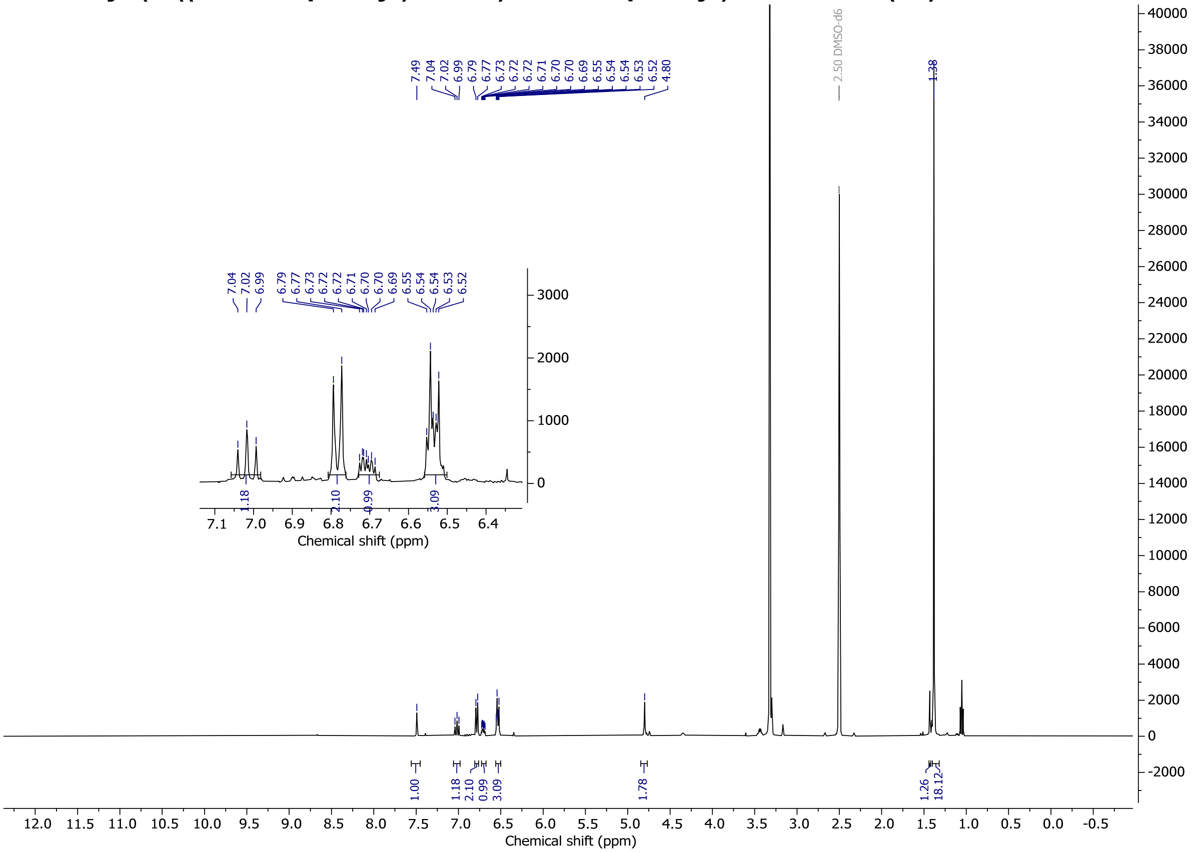

N^4-(4-Aminophenyl)-6-(trifluoromethyl)pyrimidine-2,4-diamine (85)

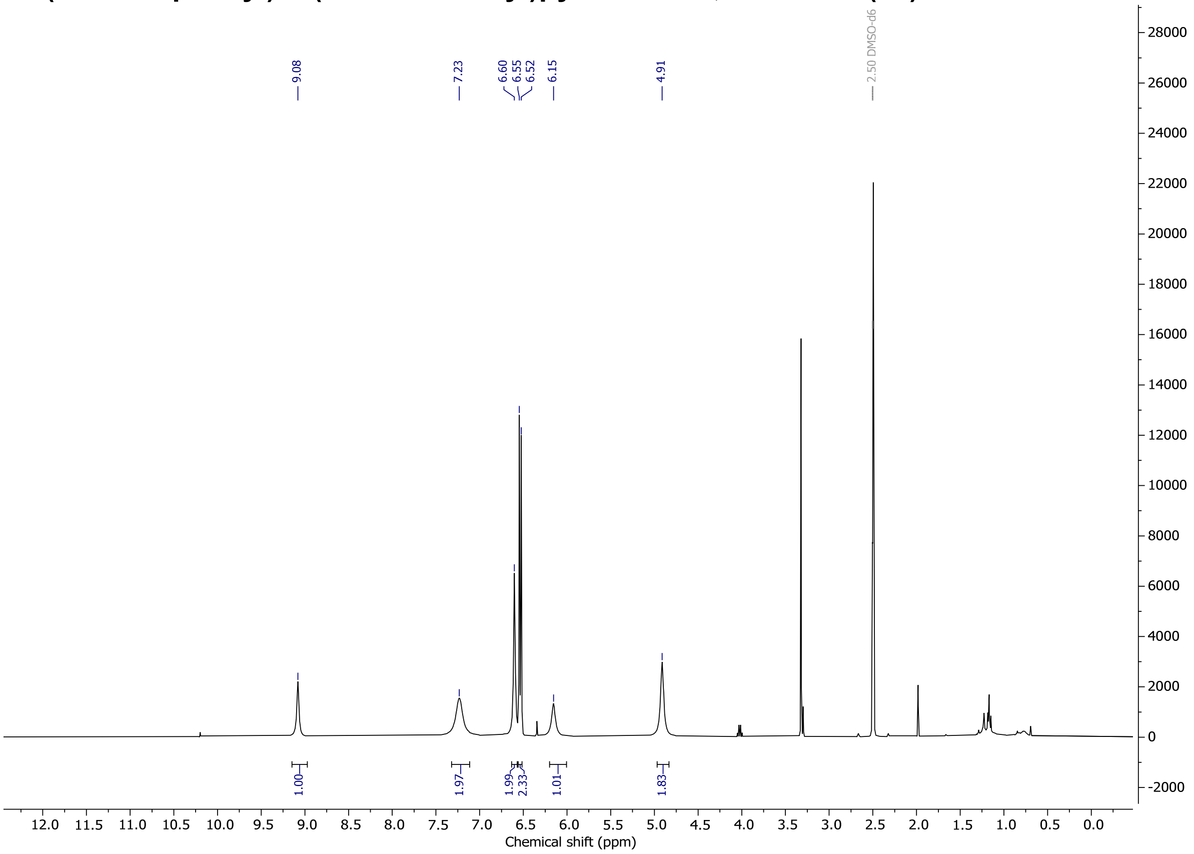

N-(4-((3-Aminophenyl)amino)phenyl)-4-(quinolin-4-ylamino)benzamide (39)

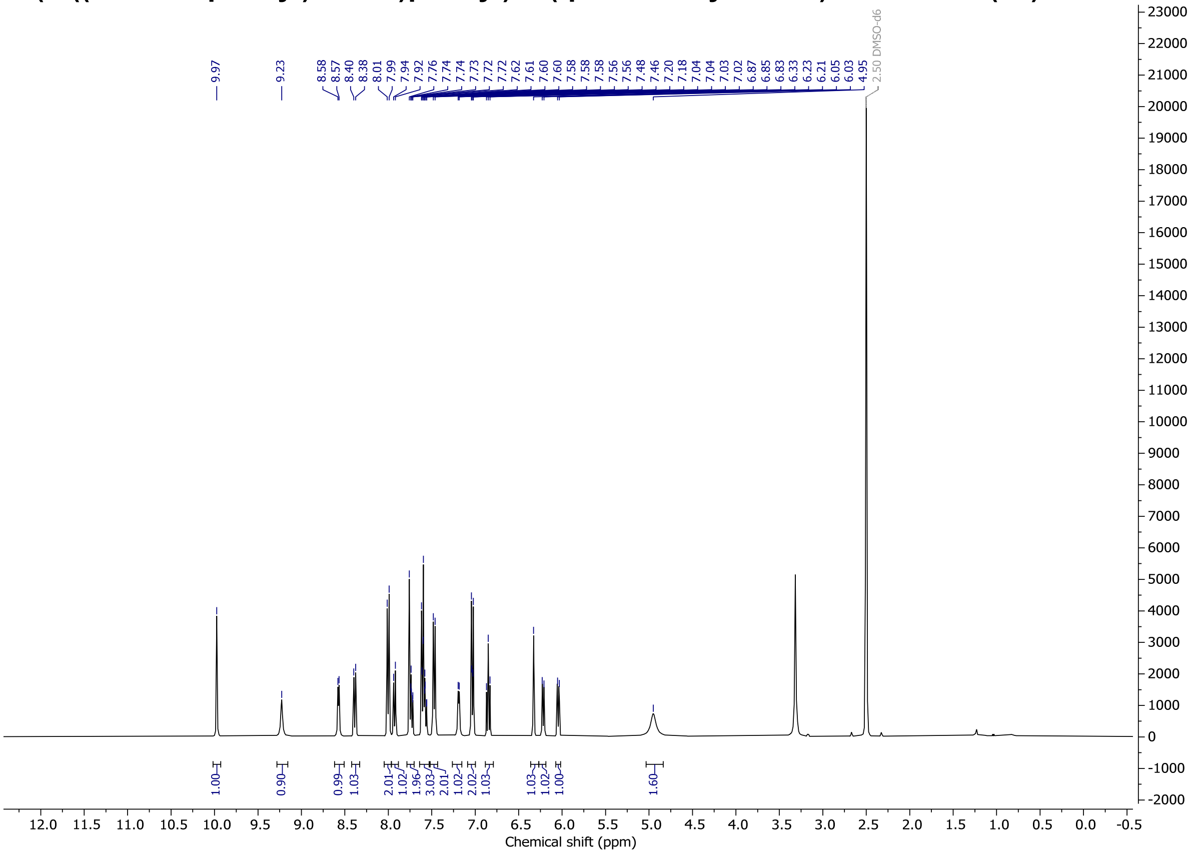

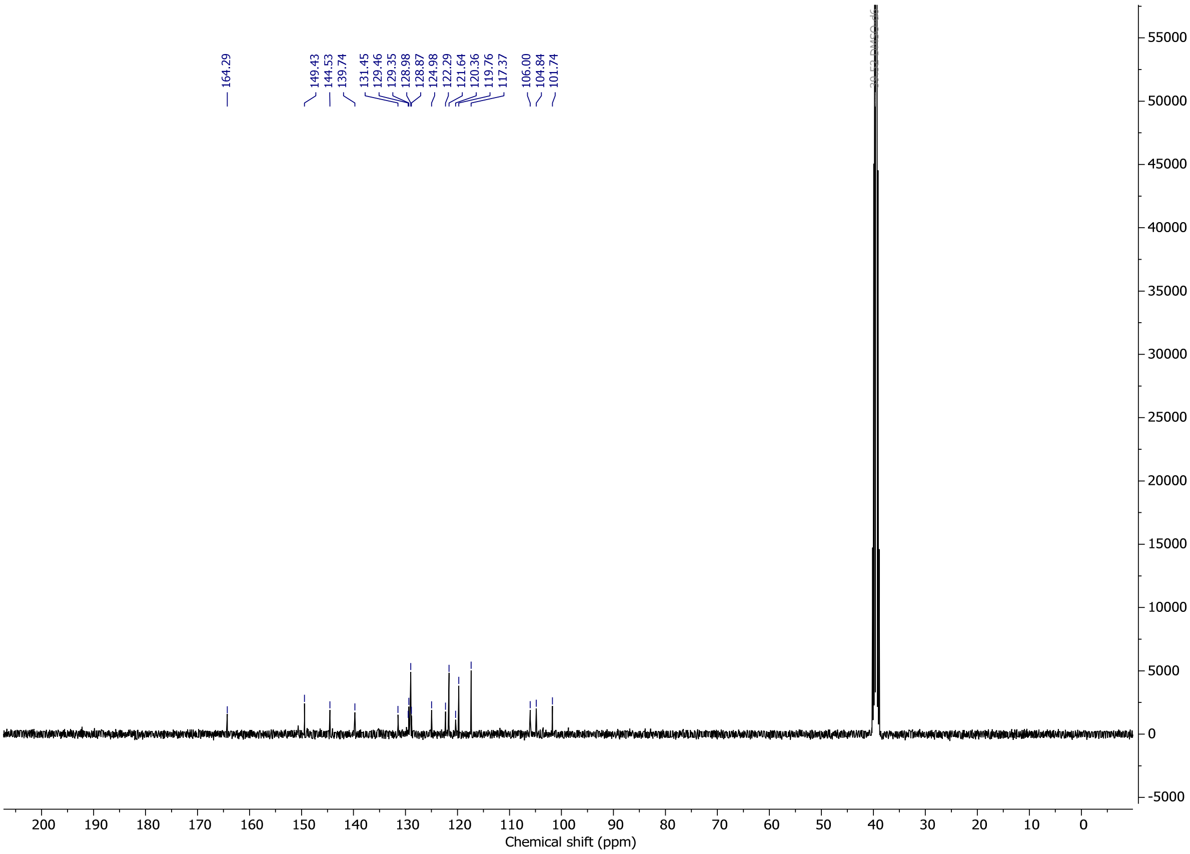

N-(4-((3-Amino-5-methylphenyl)amino)phenyl)-4-(quinolin-4-ylamino)benzamide (43)

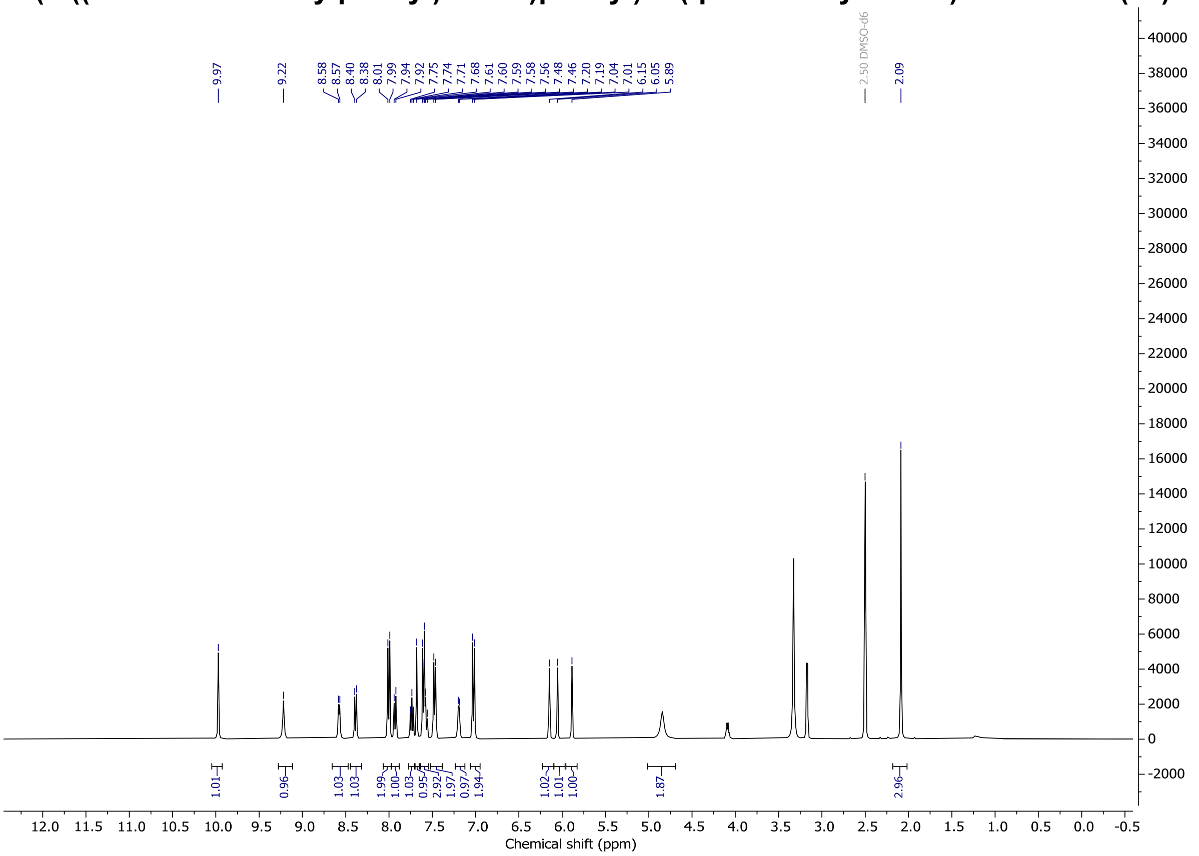

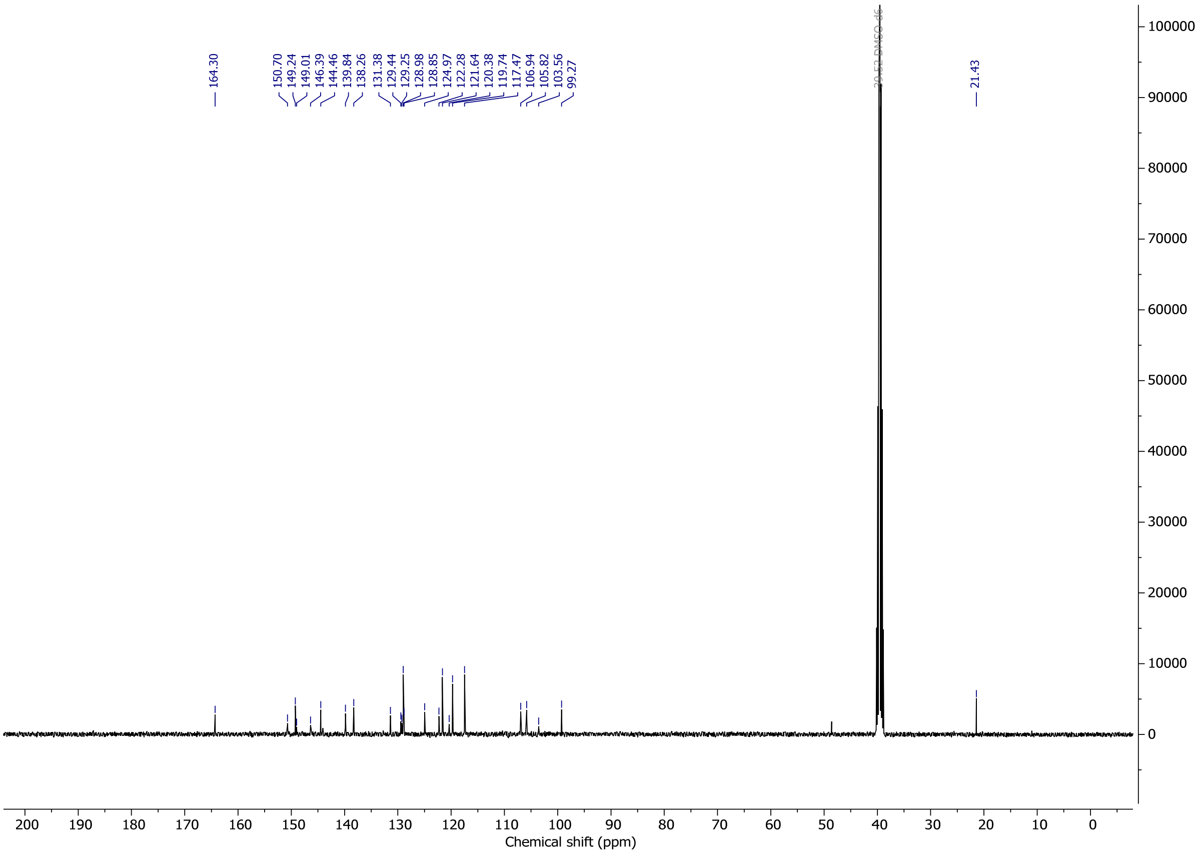

N[1]-(Pyridin-2-yl)benzene-1,4-diamine (88)

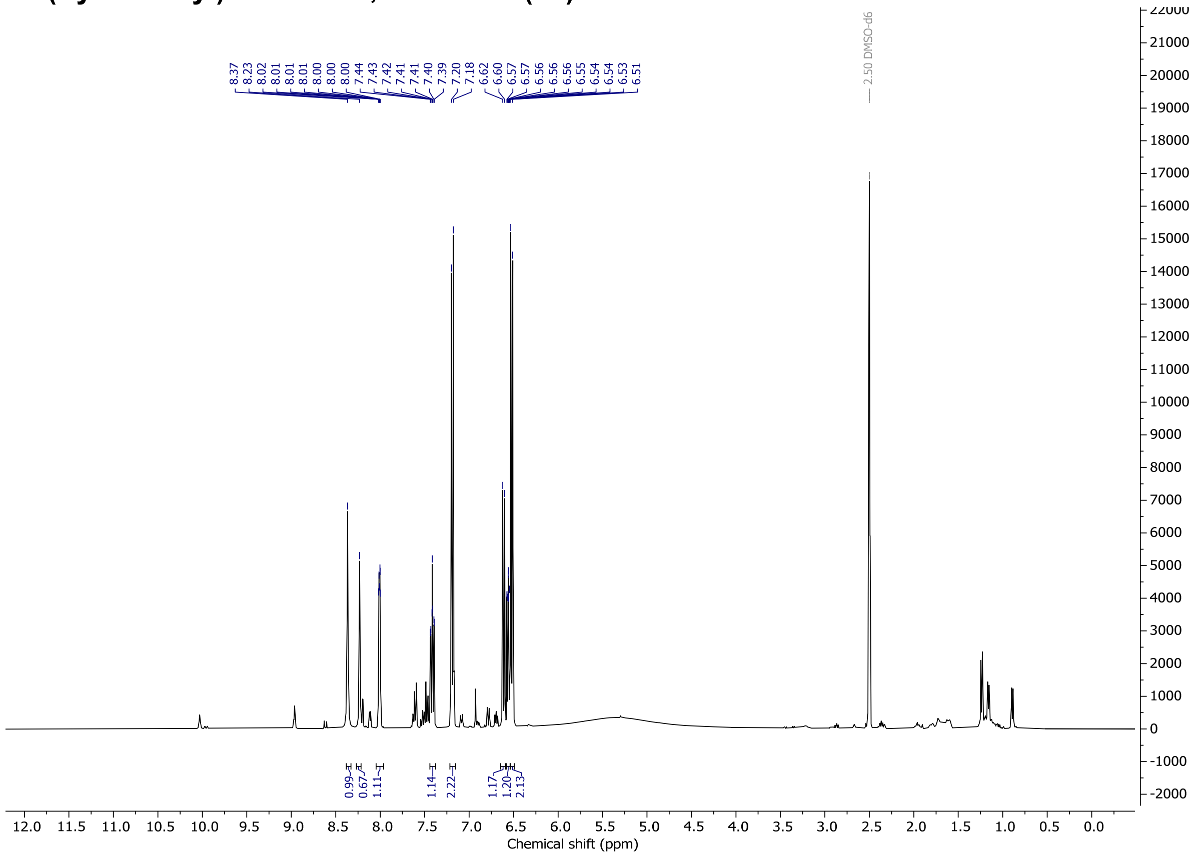

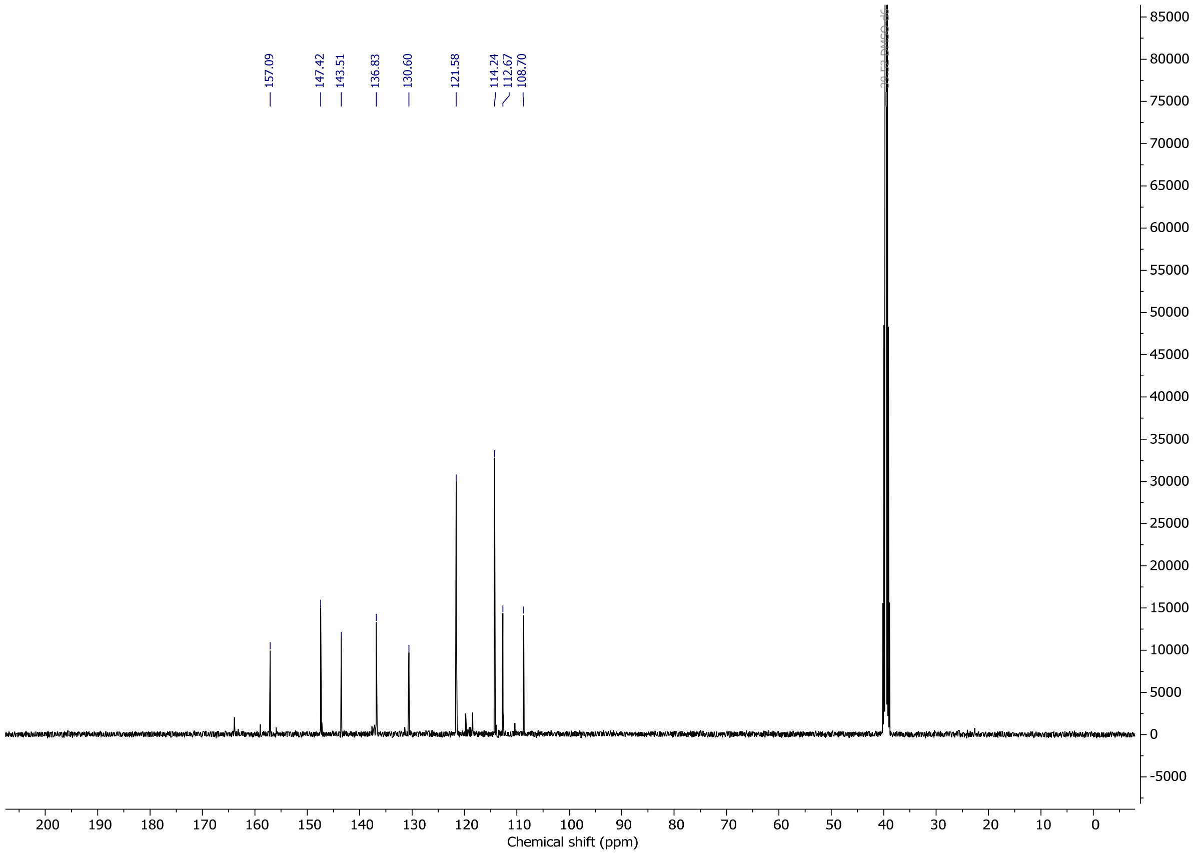

3-((4-Aminophenyl)amino)phenol (89)

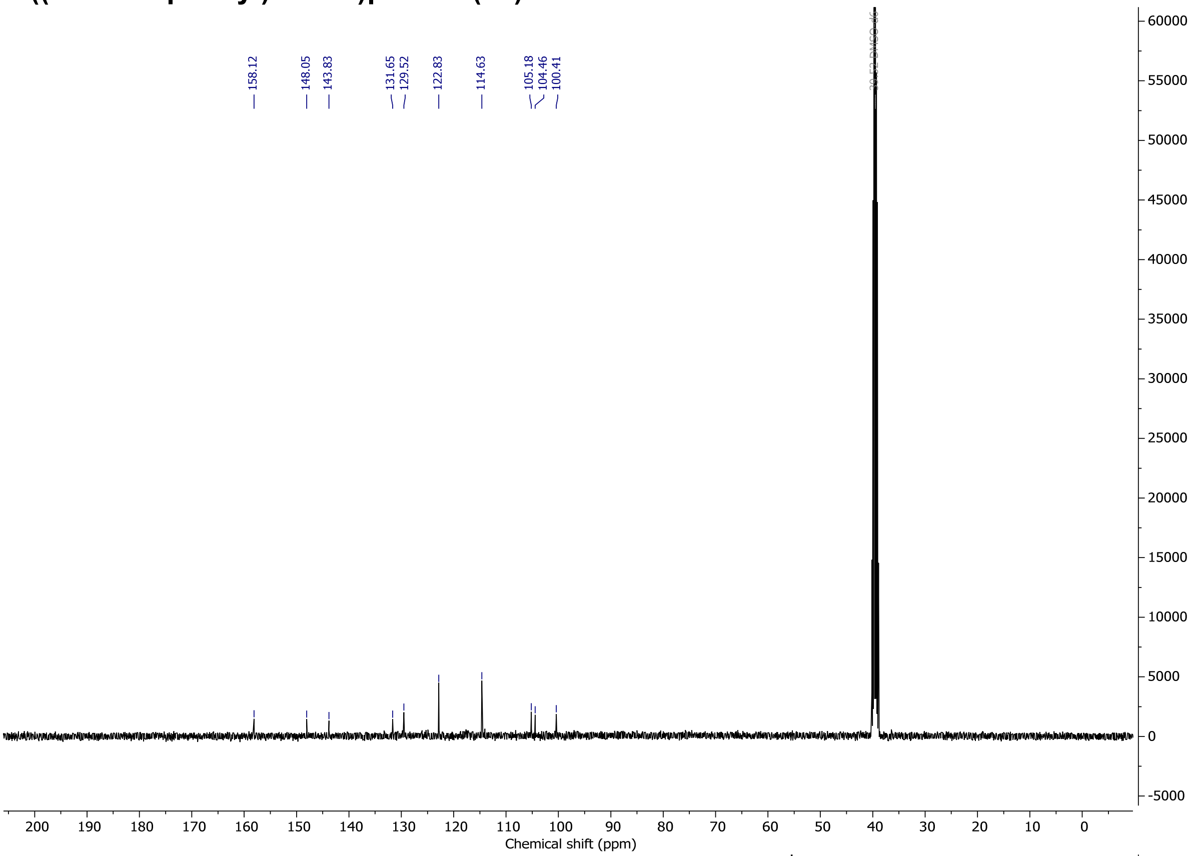

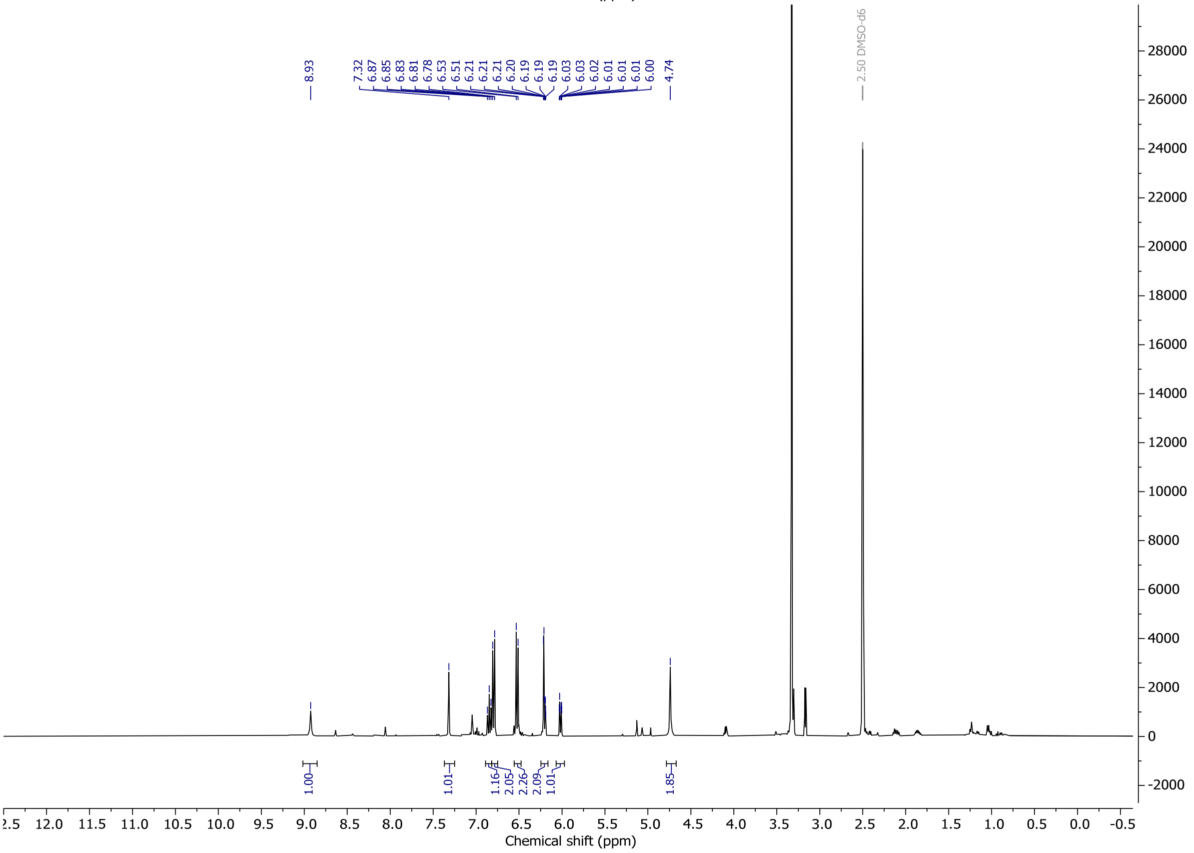

4-(Quinolin-4-ylamino)benzoic acid (90)

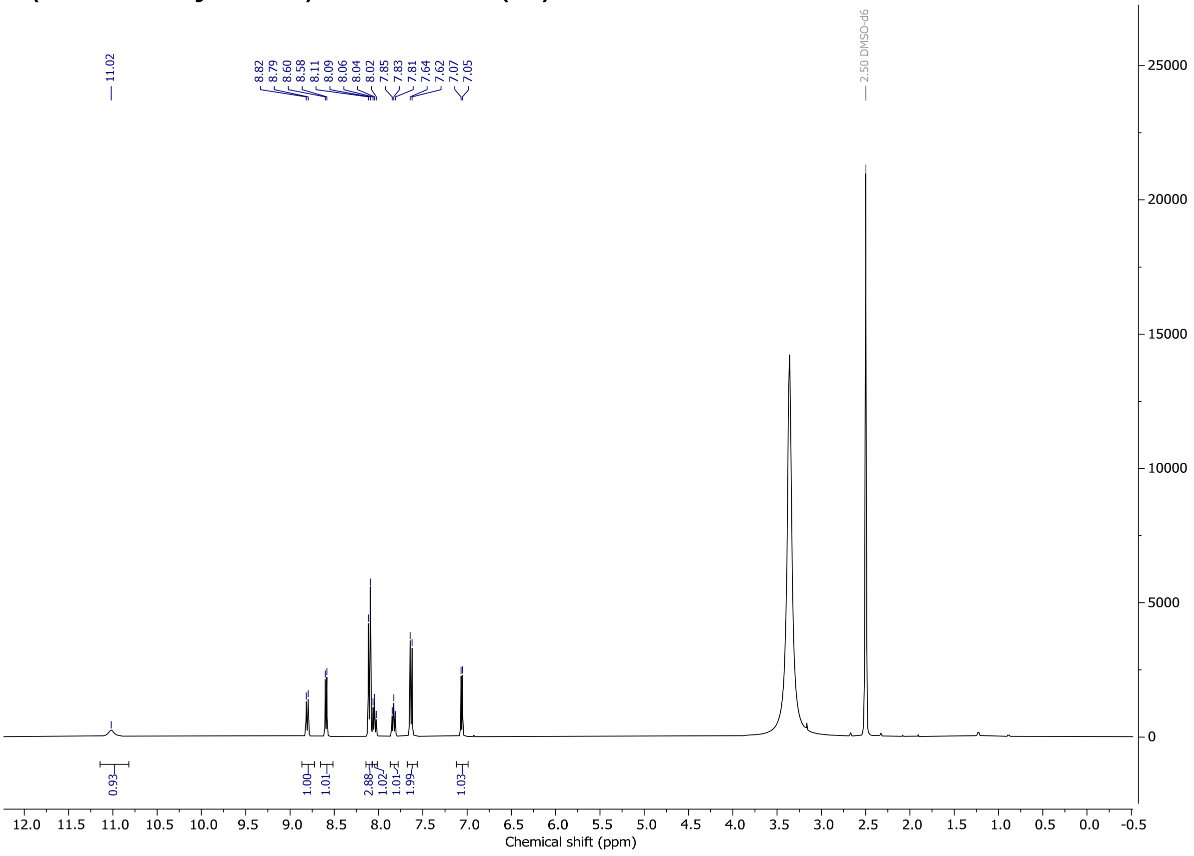

4-(Naphthalen-1-ylamino)benzoic acid (91)

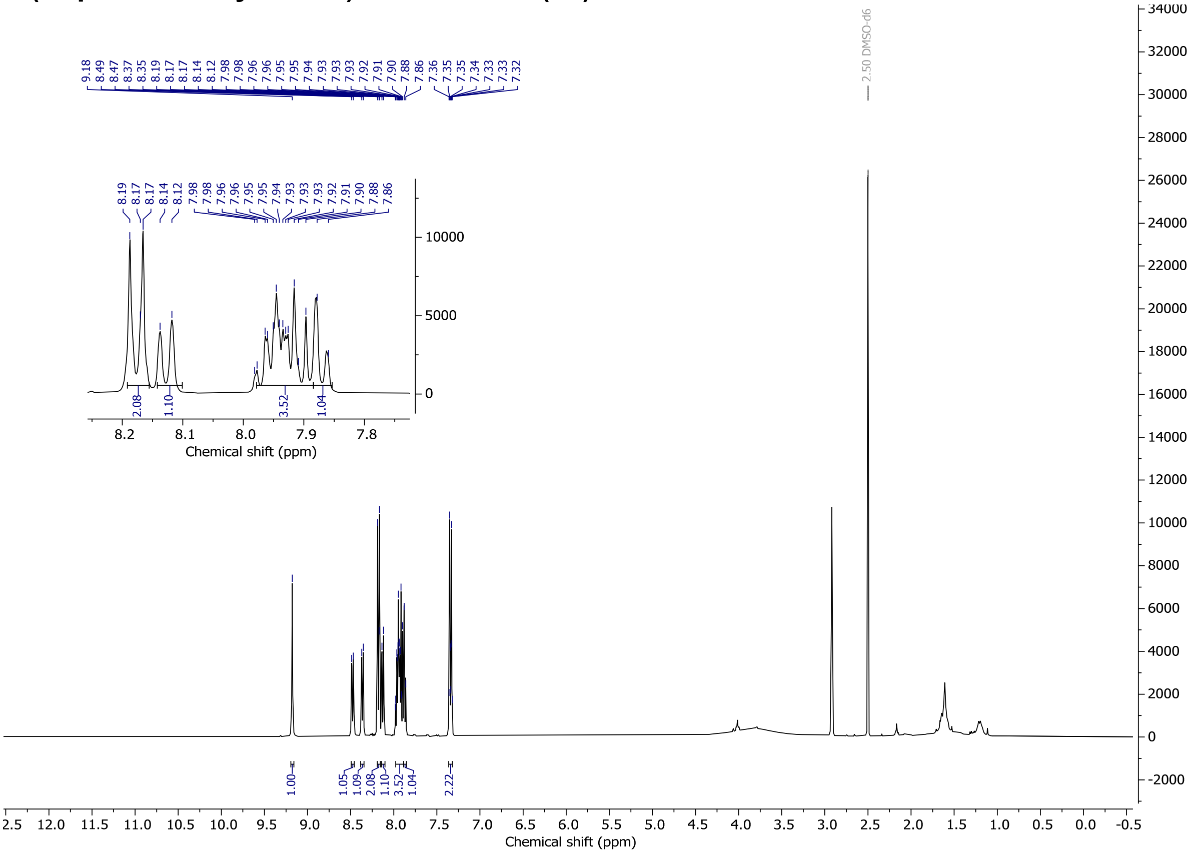

4-(Quinazolin-4-ylamino)benzoic acid (92)

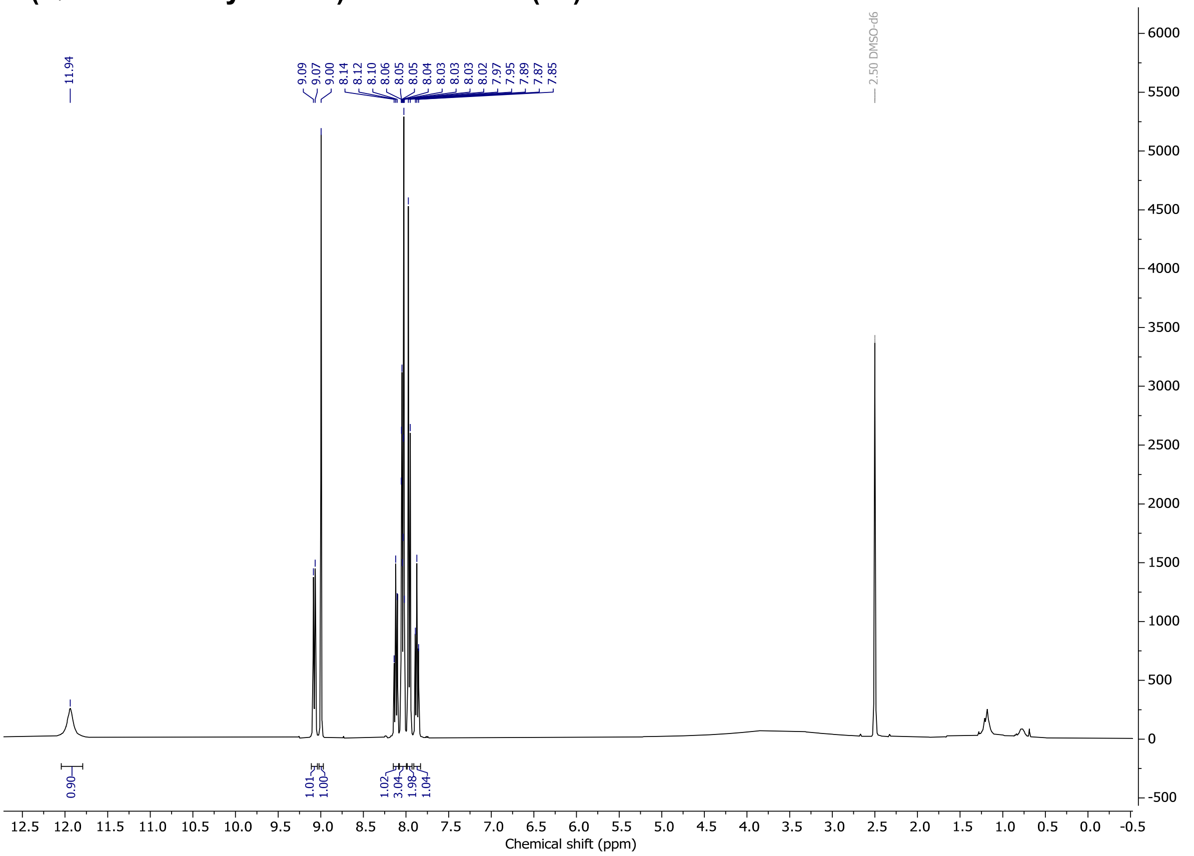

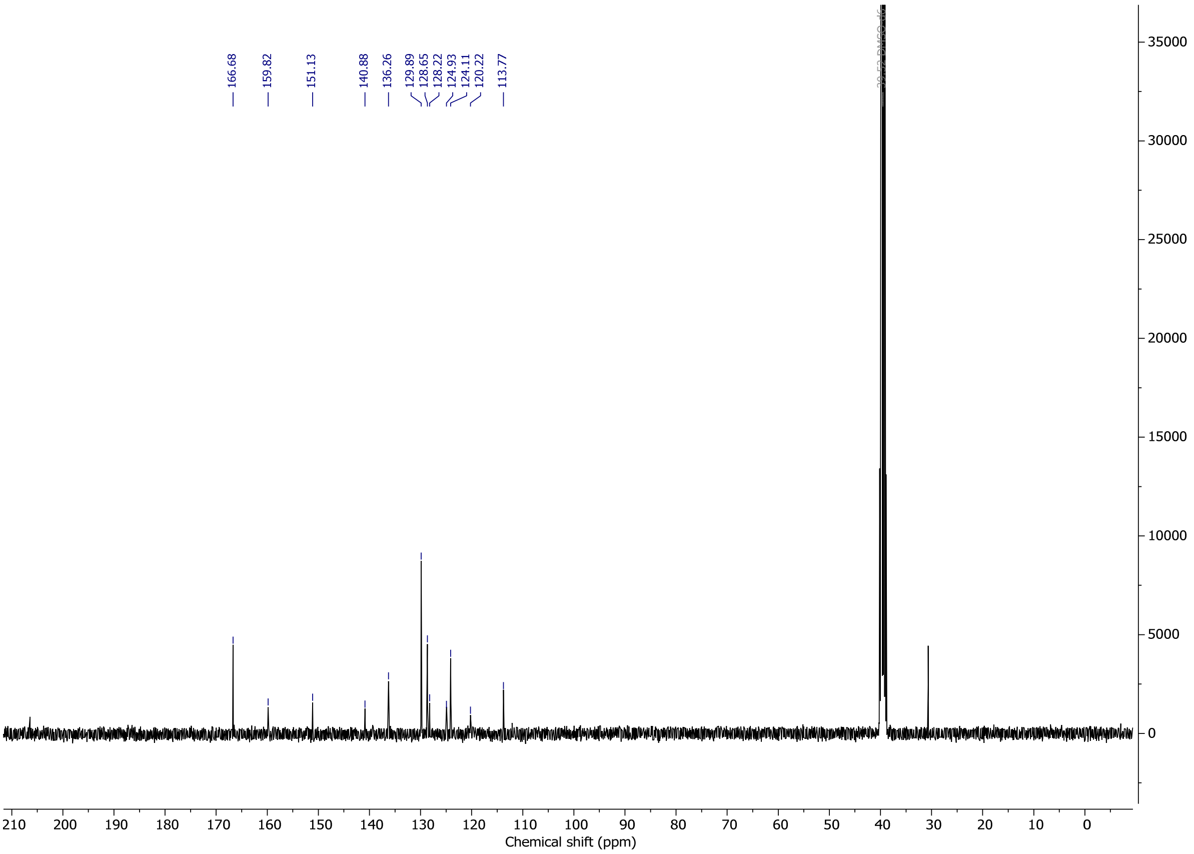

4-((3a,7a-Dihydro-1H-pyrrolo[2,3-b]pyridin-4-yl)amino)benzoic acid (93)

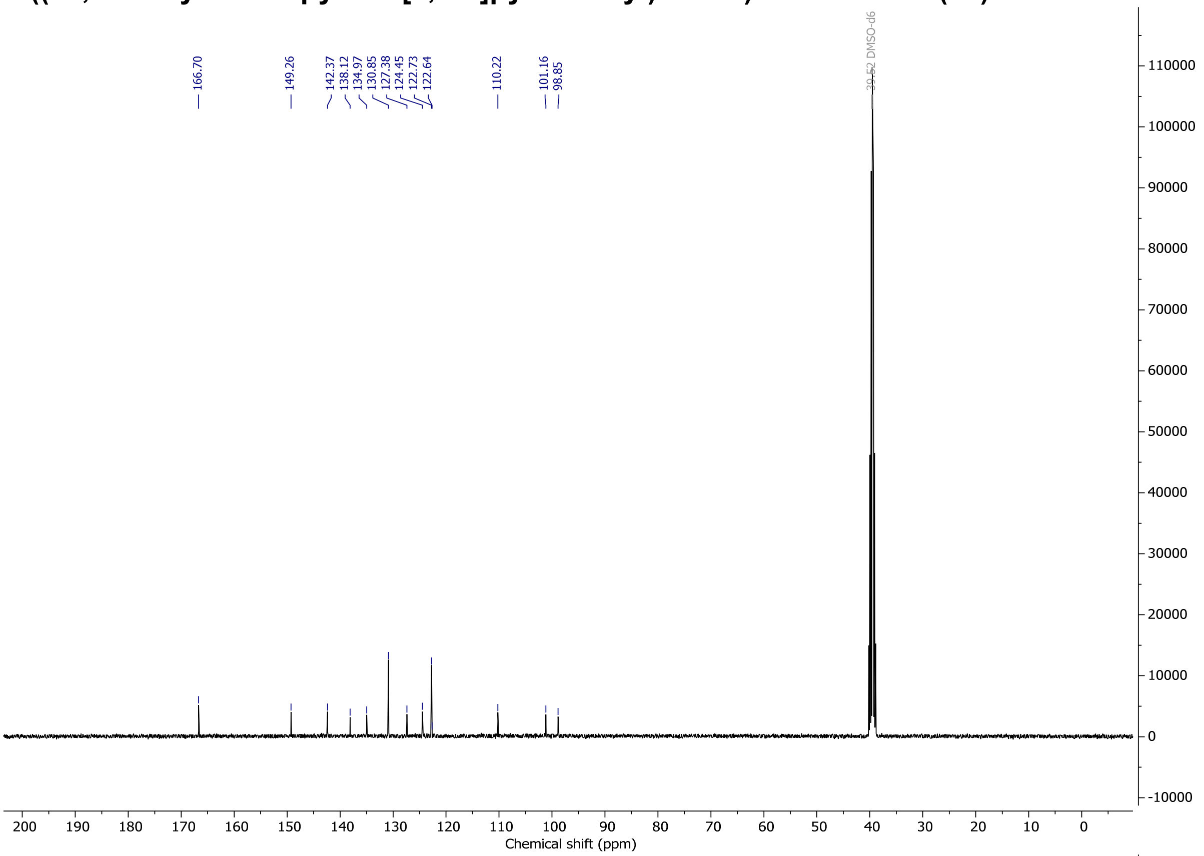

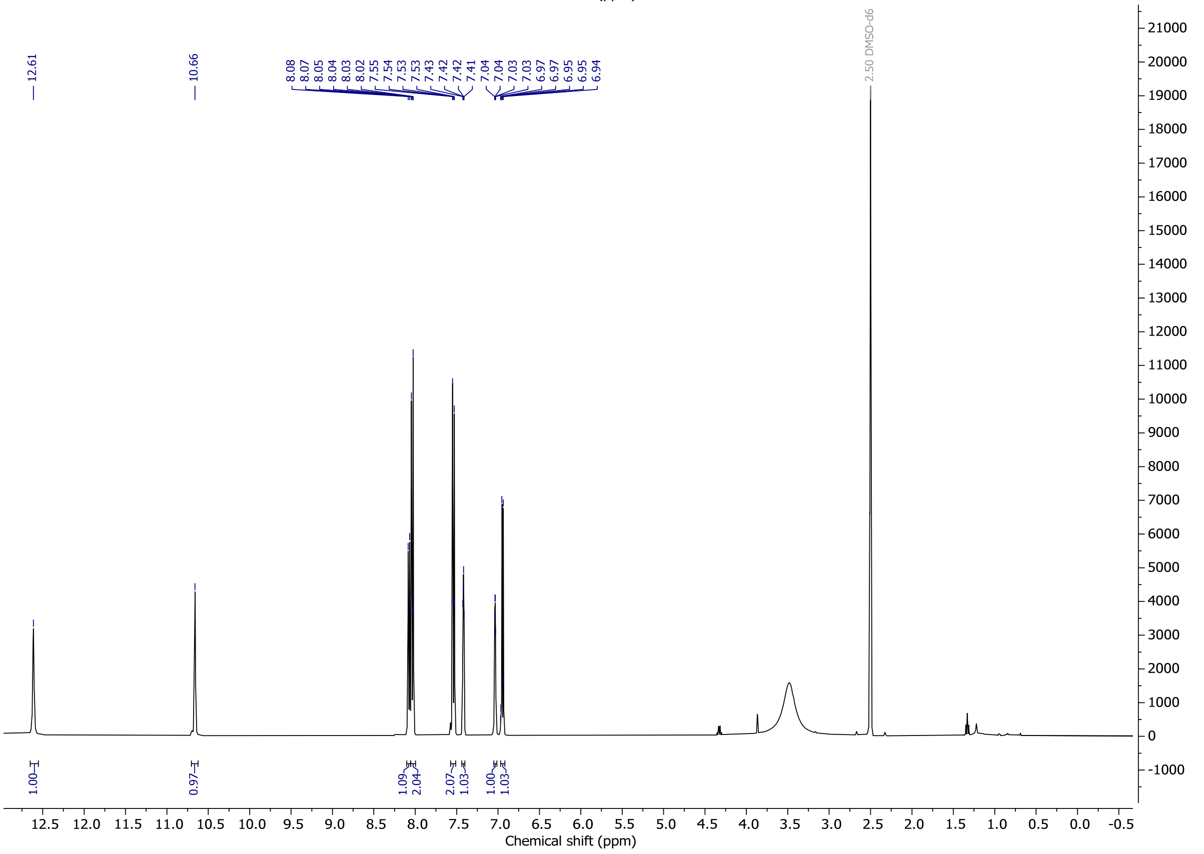

N-(4-((2-Amino-6-methylpyrimidin-4-yl)amino)phenyl)-4-(quinolin-4-ylamino)benzamide (1)

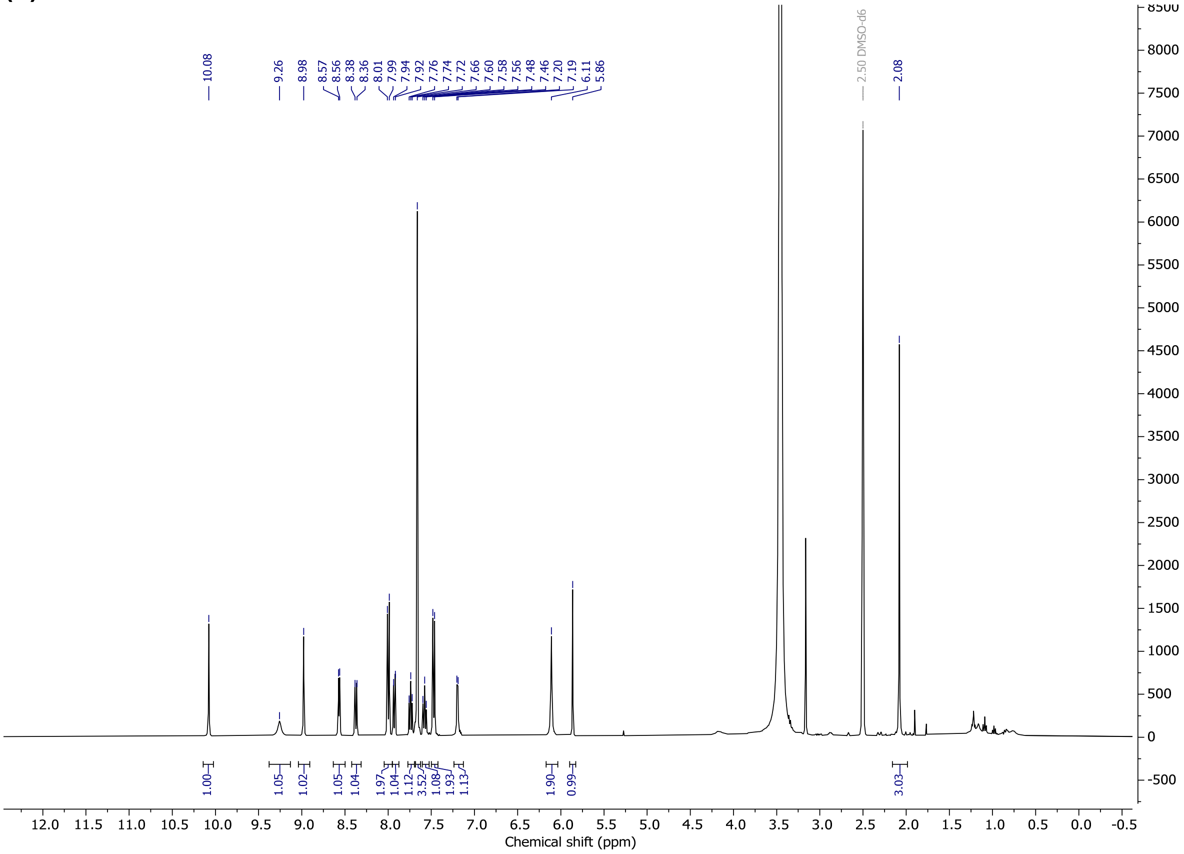

N-(4-((2-Aminopyrimidin-4-yl)amino)phenyl)-4-(quinolin-4-ylamino)benzamide (26)

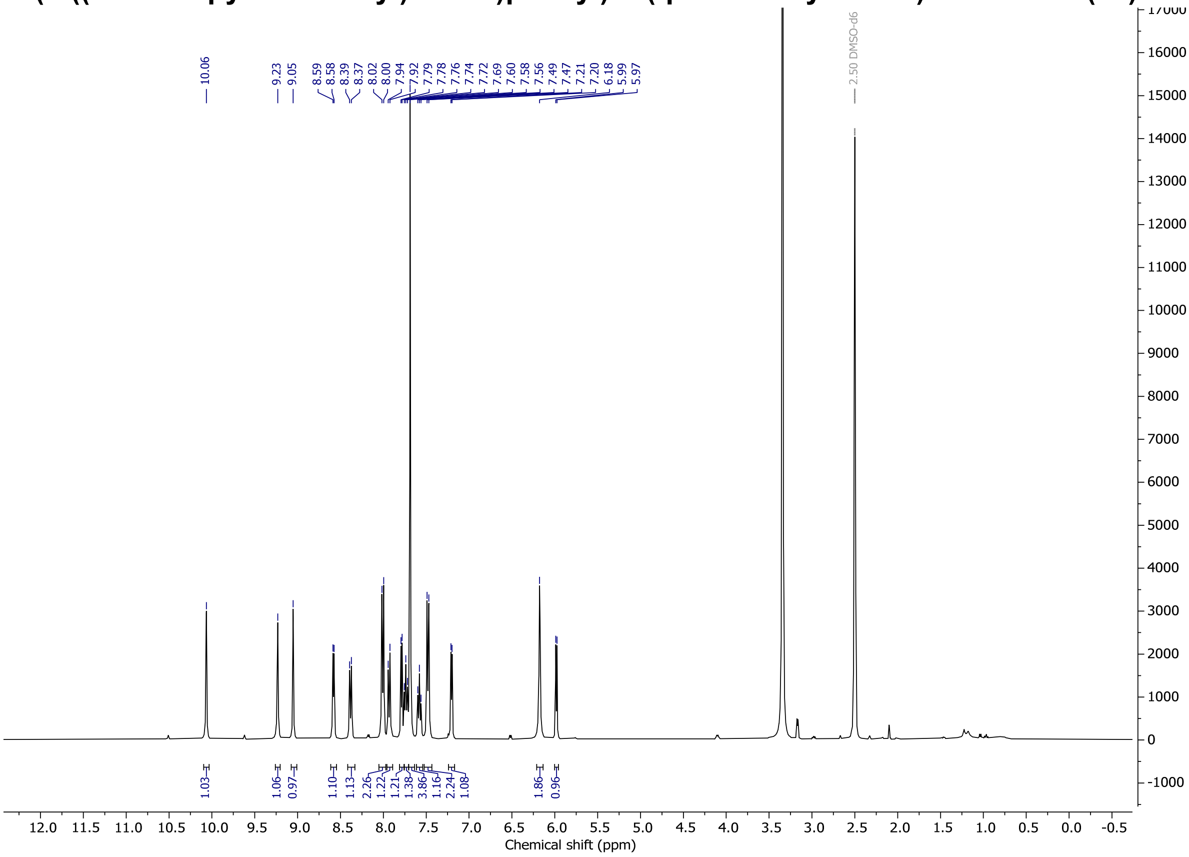

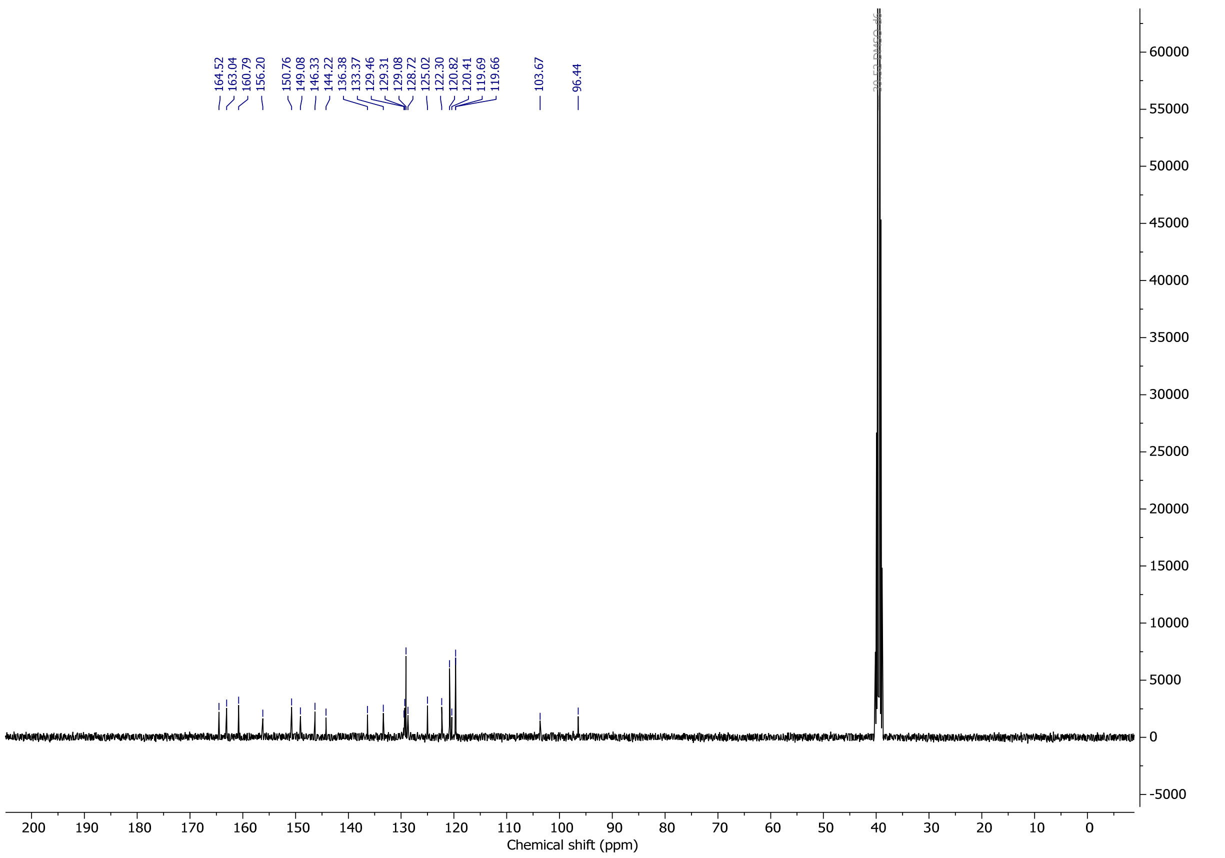

4-(Quinolin-4-ylamino)-*N*-(4-((5-(trifluoromethyl)pyridin-2-yl)amino)phenyl)benzamide (27)

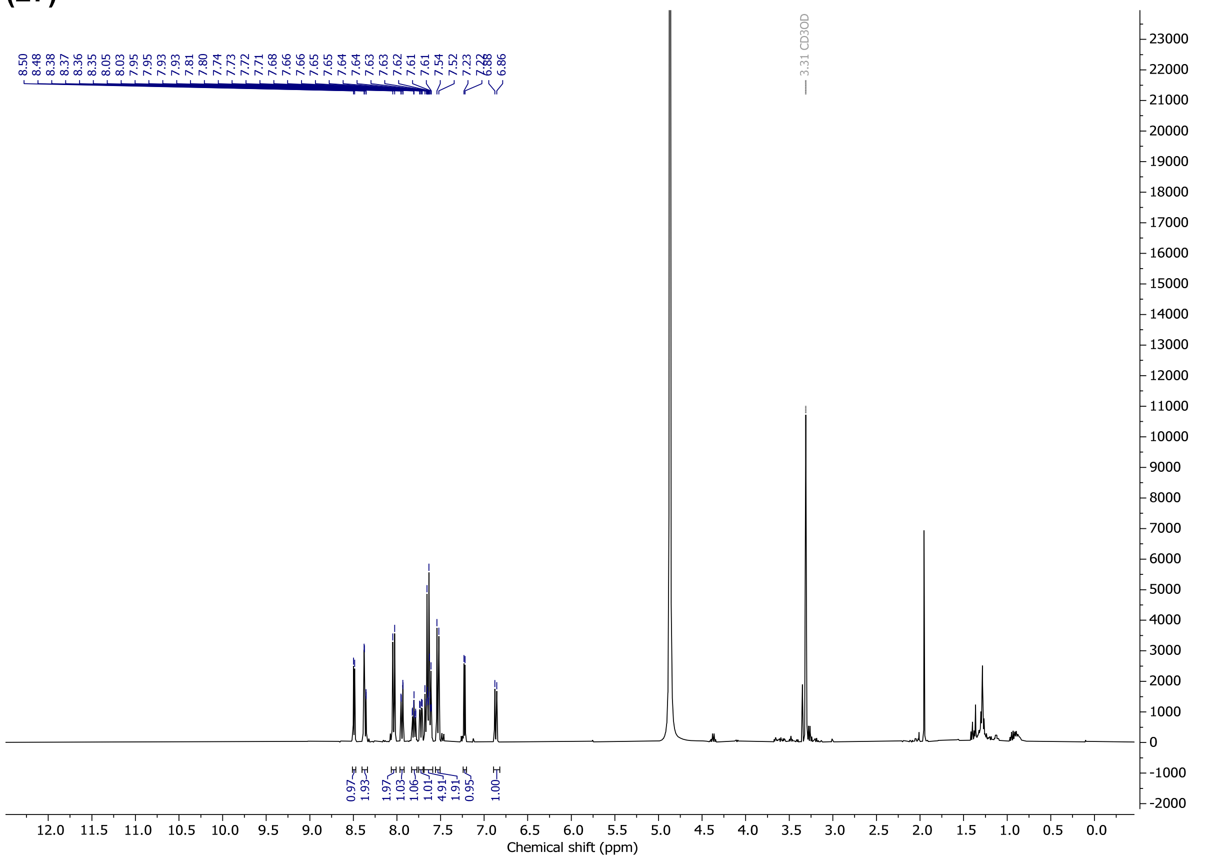

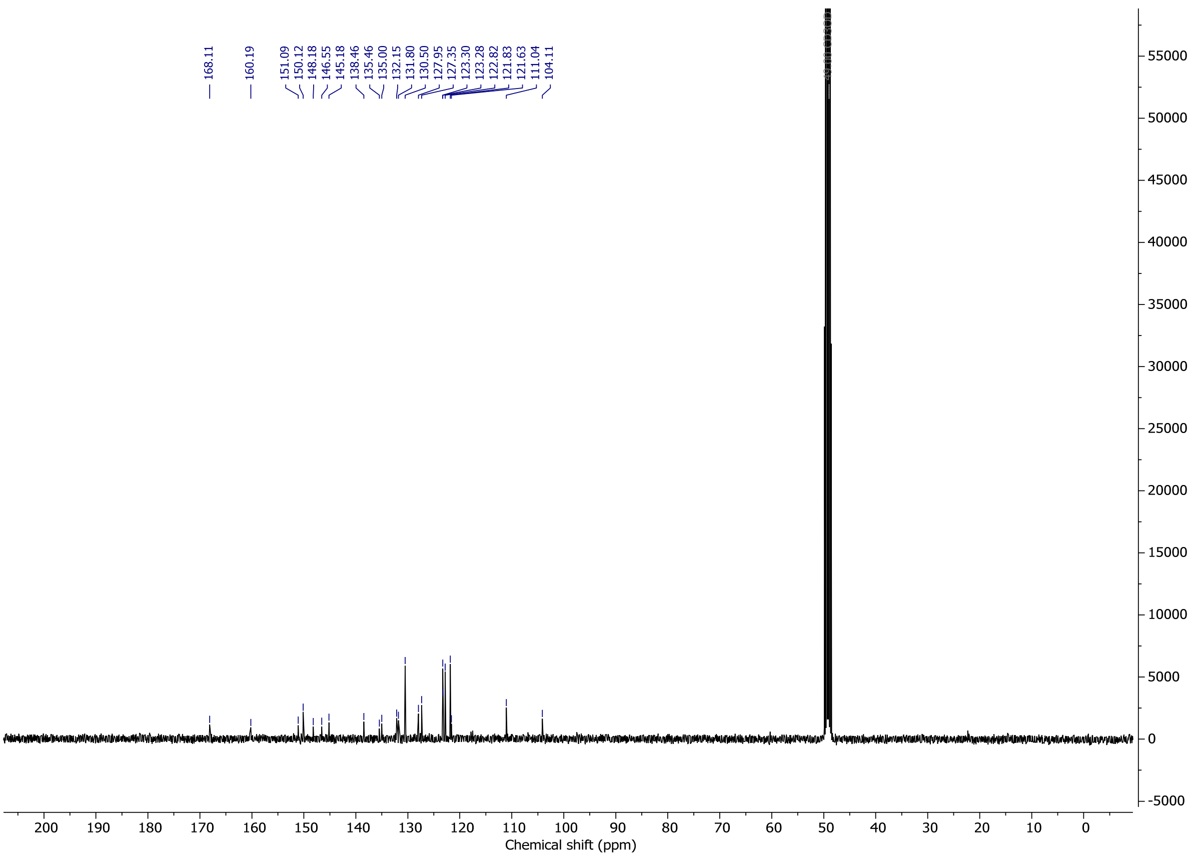

4-(Quinolin-4-ylamino)-*N*-(4-((4-(trifluoromethyl)phenyl)amino)phenyl)benzamide (28)

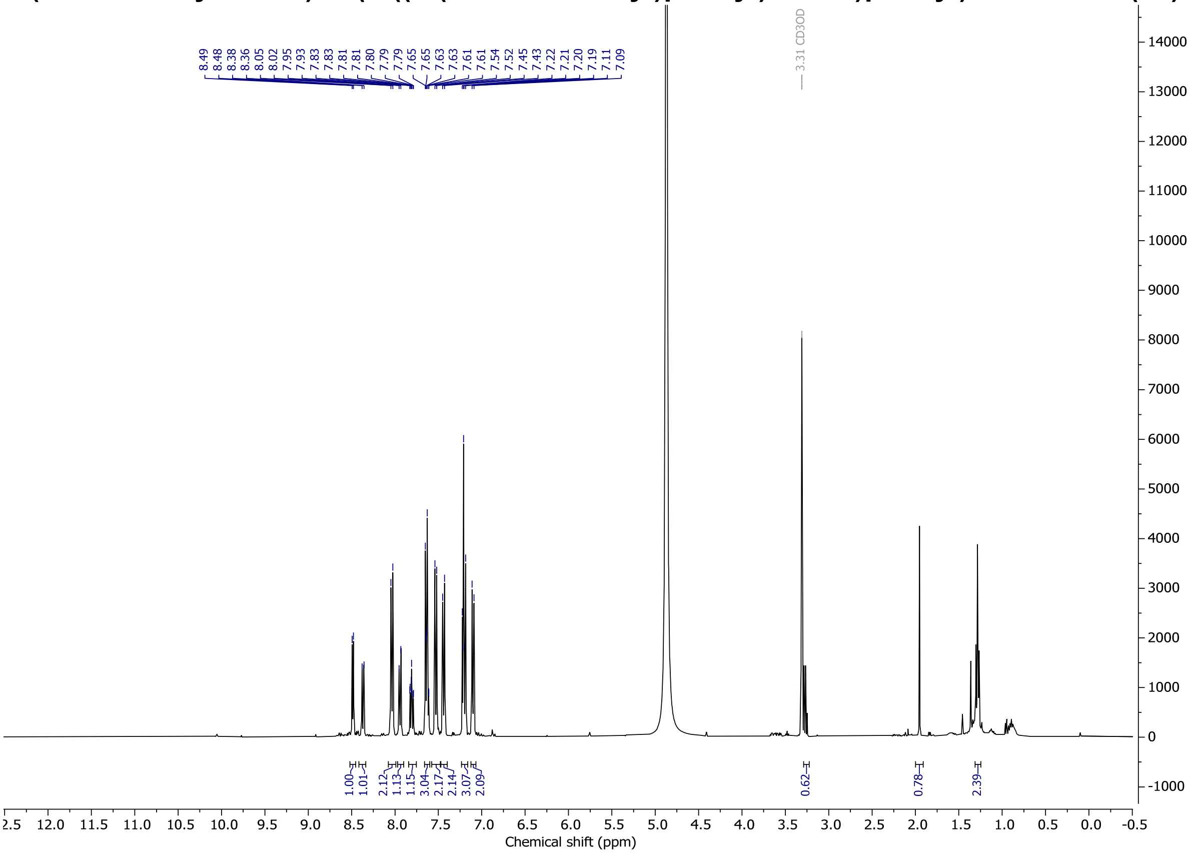

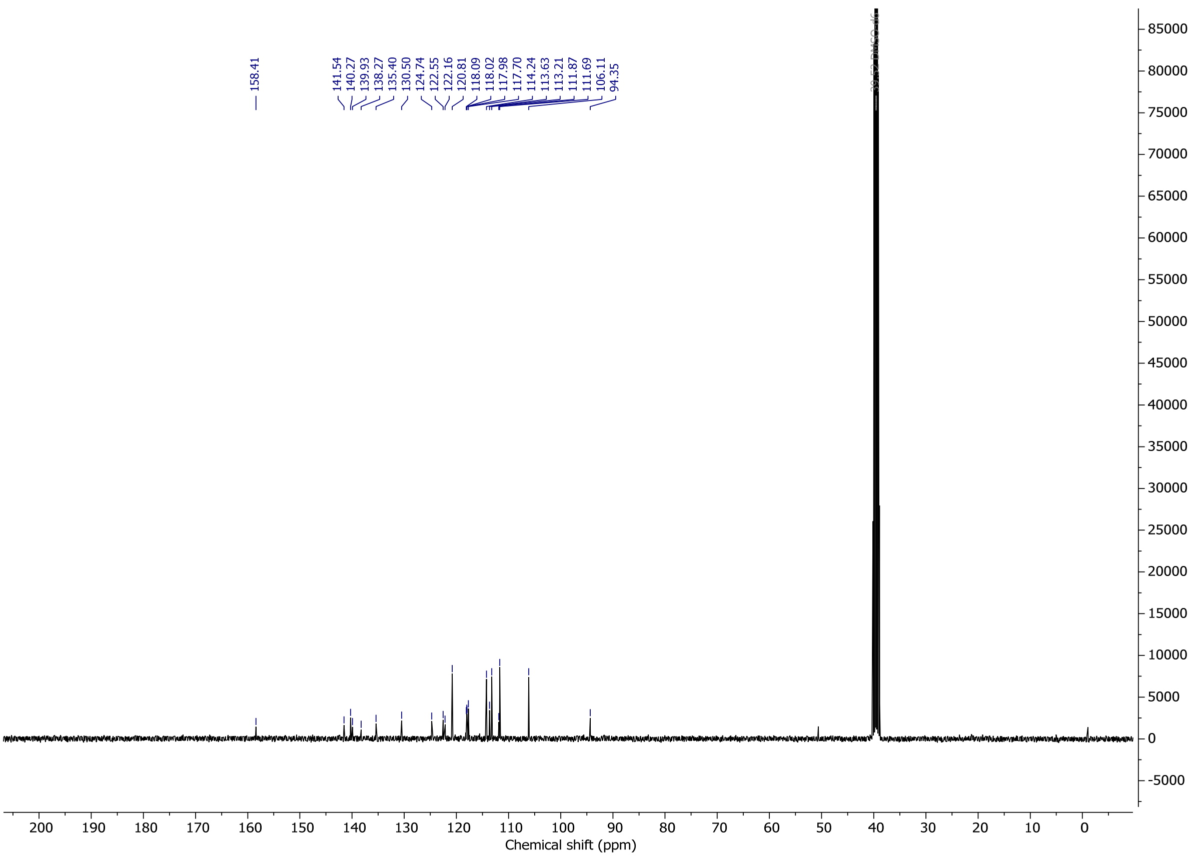

4-(Quinolin-4-ylamino)-*N*-(4-((2-(trifluoromethyl)phenyl)amino)phenyl)benzamide (29)

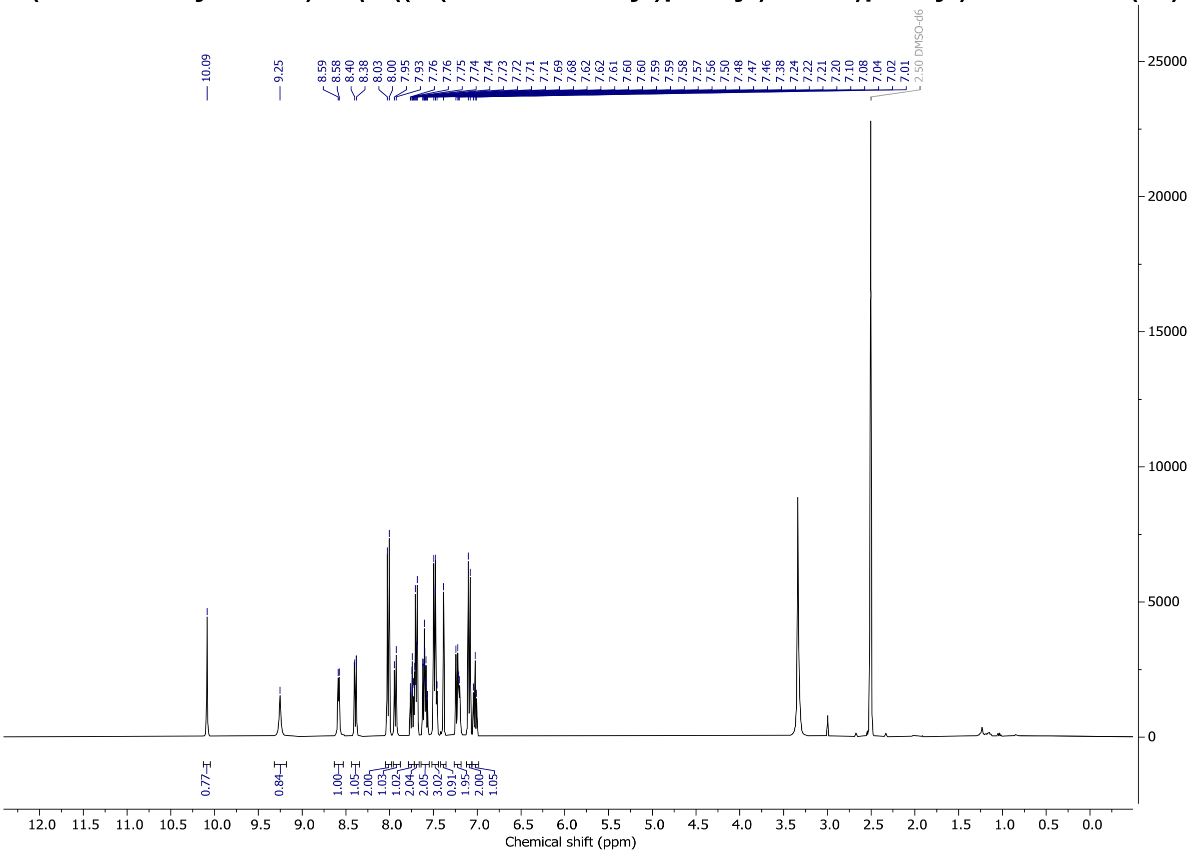

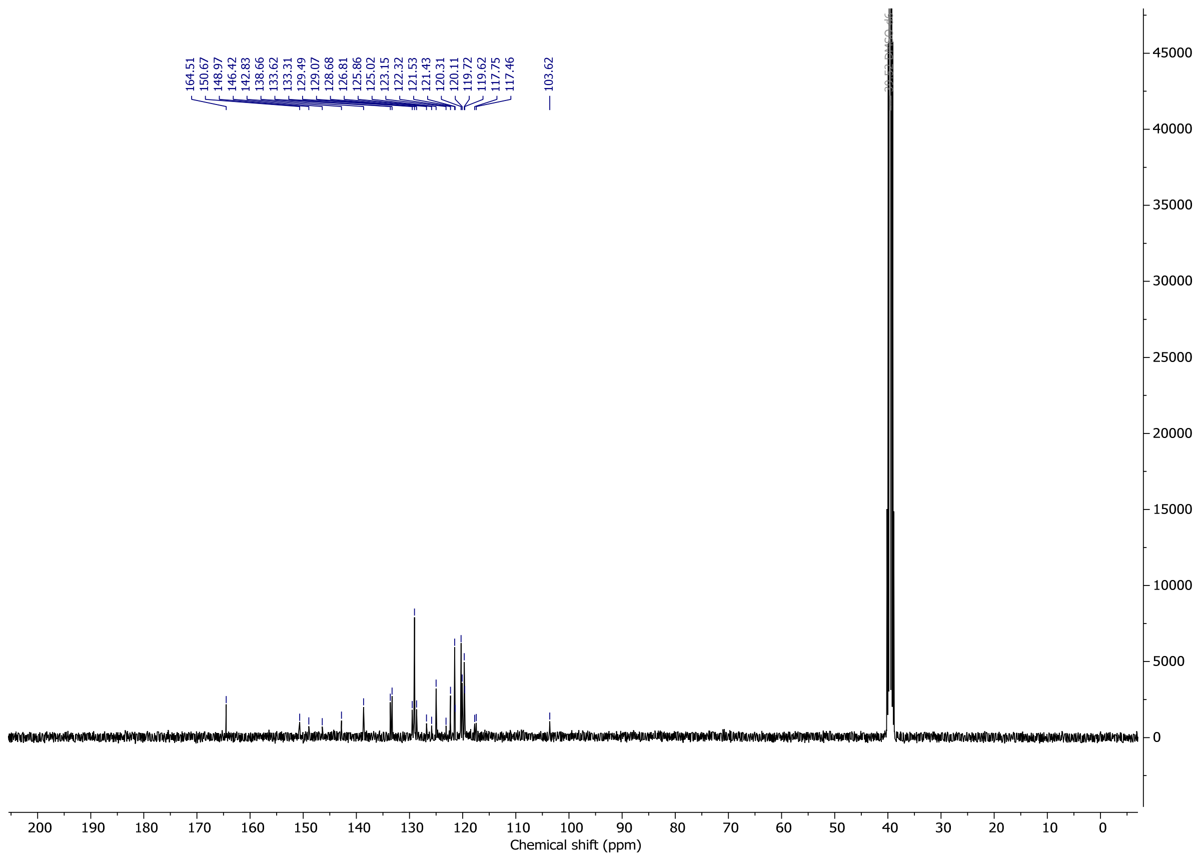

4-(Quinolin-4-ylamino)-*N*-(4-(*p*-tolylamino)phenyl)benzamide (30)

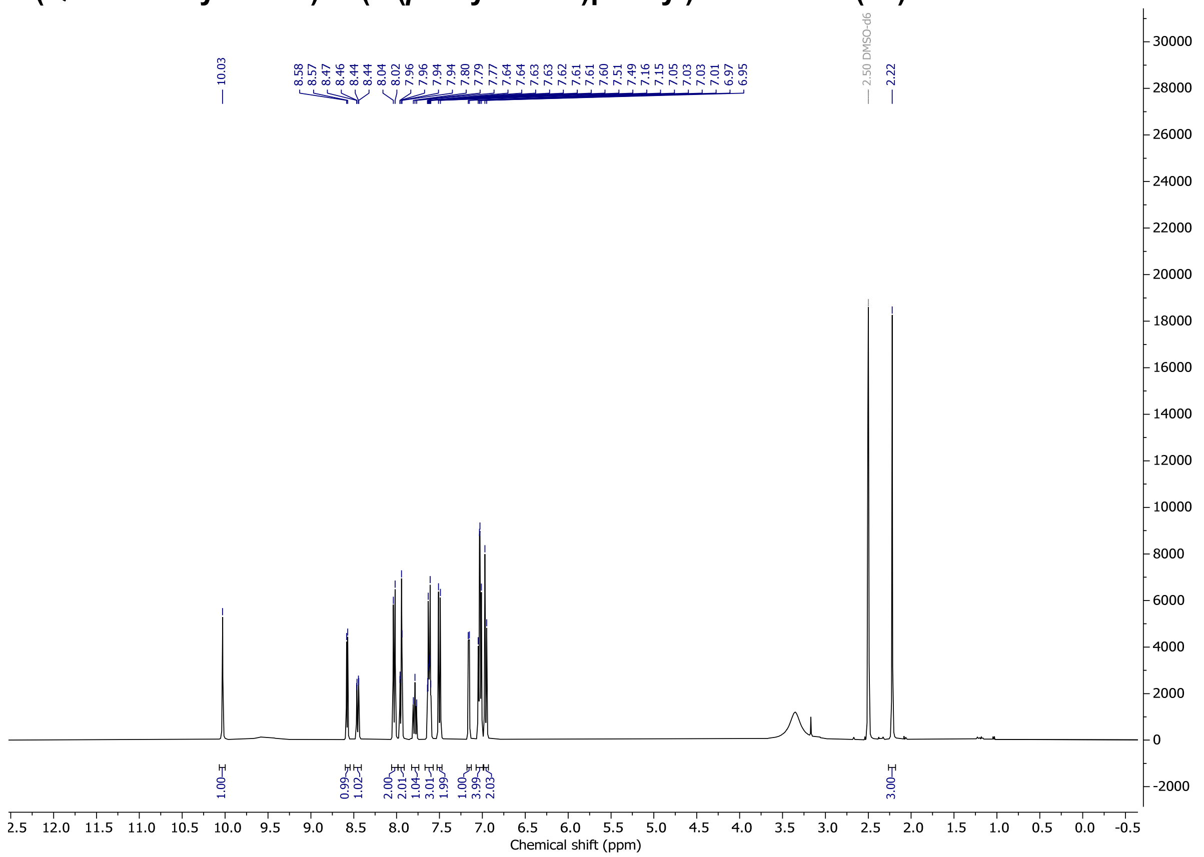

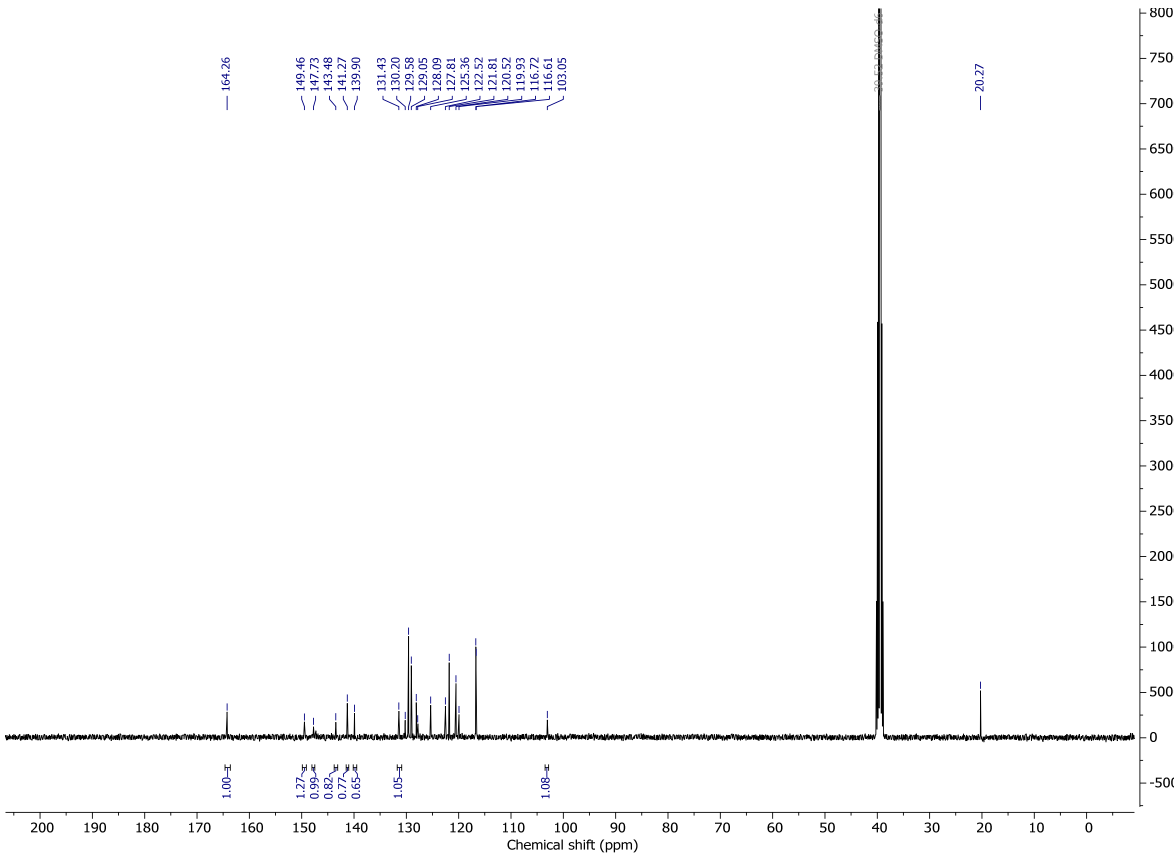

N-(4-(Phenylamino)phenyl)-4-(quinolin-4-ylamino)benzamide (31)

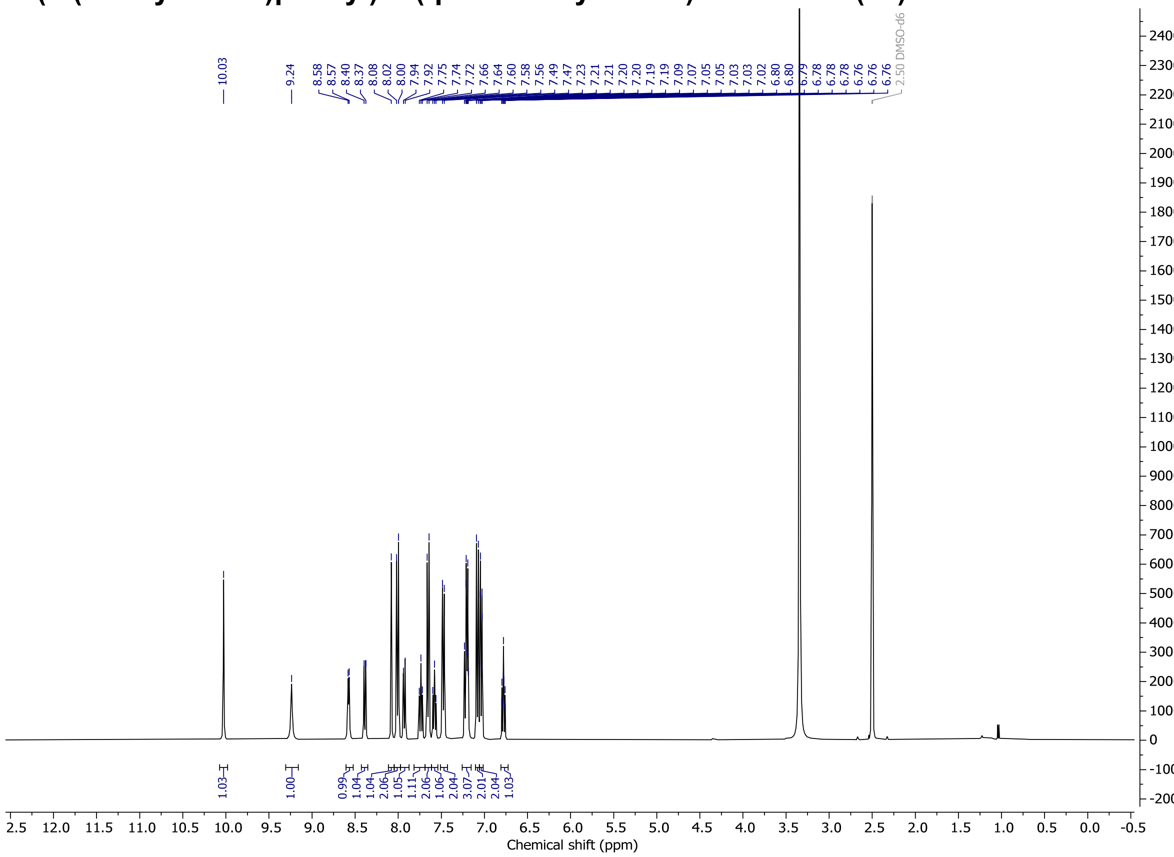

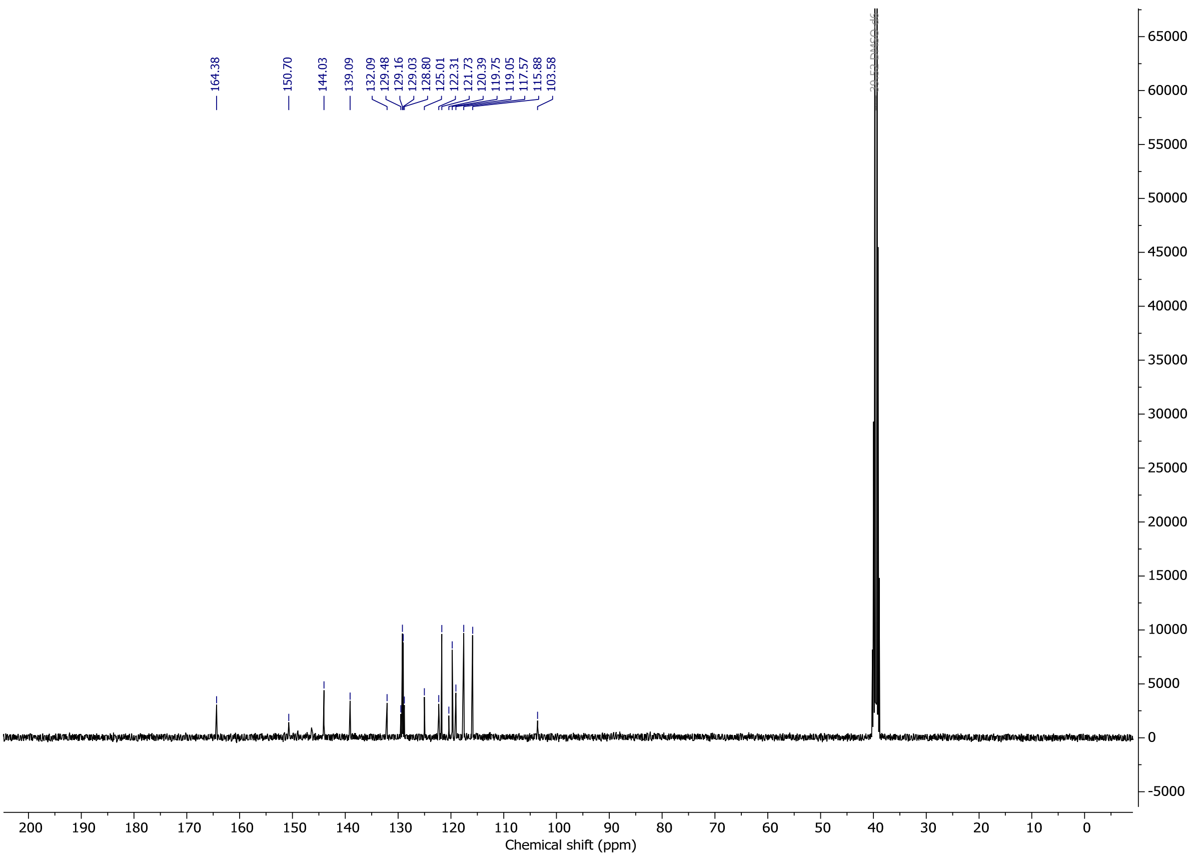

N-(4-((4-Methoxyphenyl)amino)phenyl)-4-(quinolin-4-ylamino)benzamide (32)

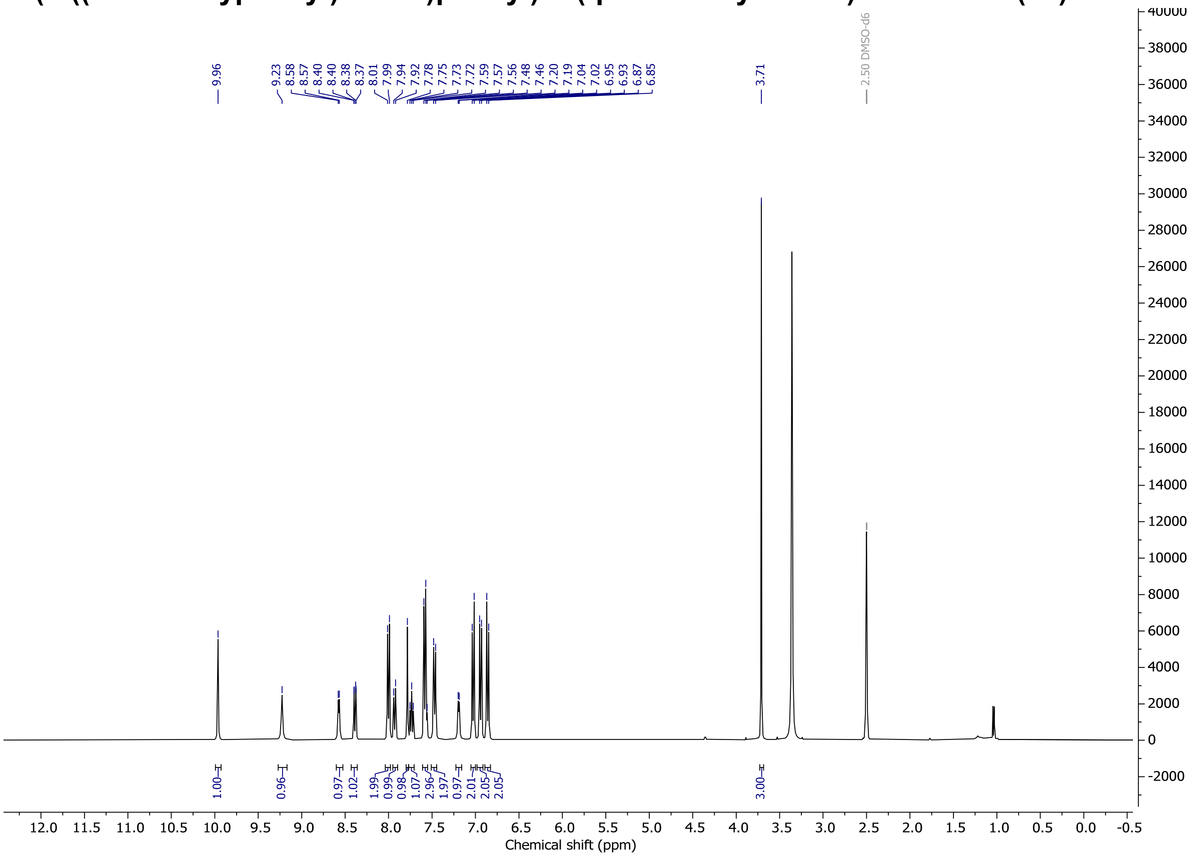

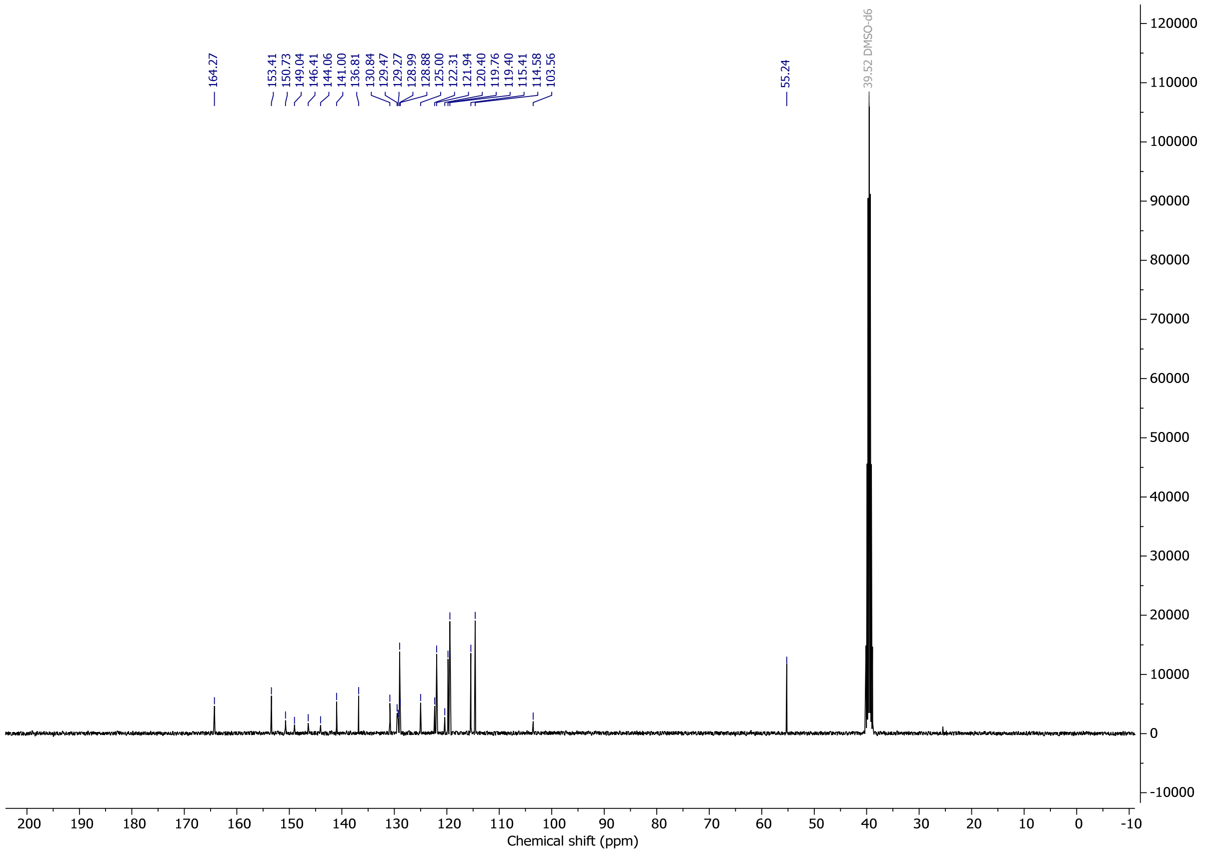

N-(4-((4-Fluorophenyl)amino)phenyl)-4-(quinolin-4-ylamino)benzamide (33)

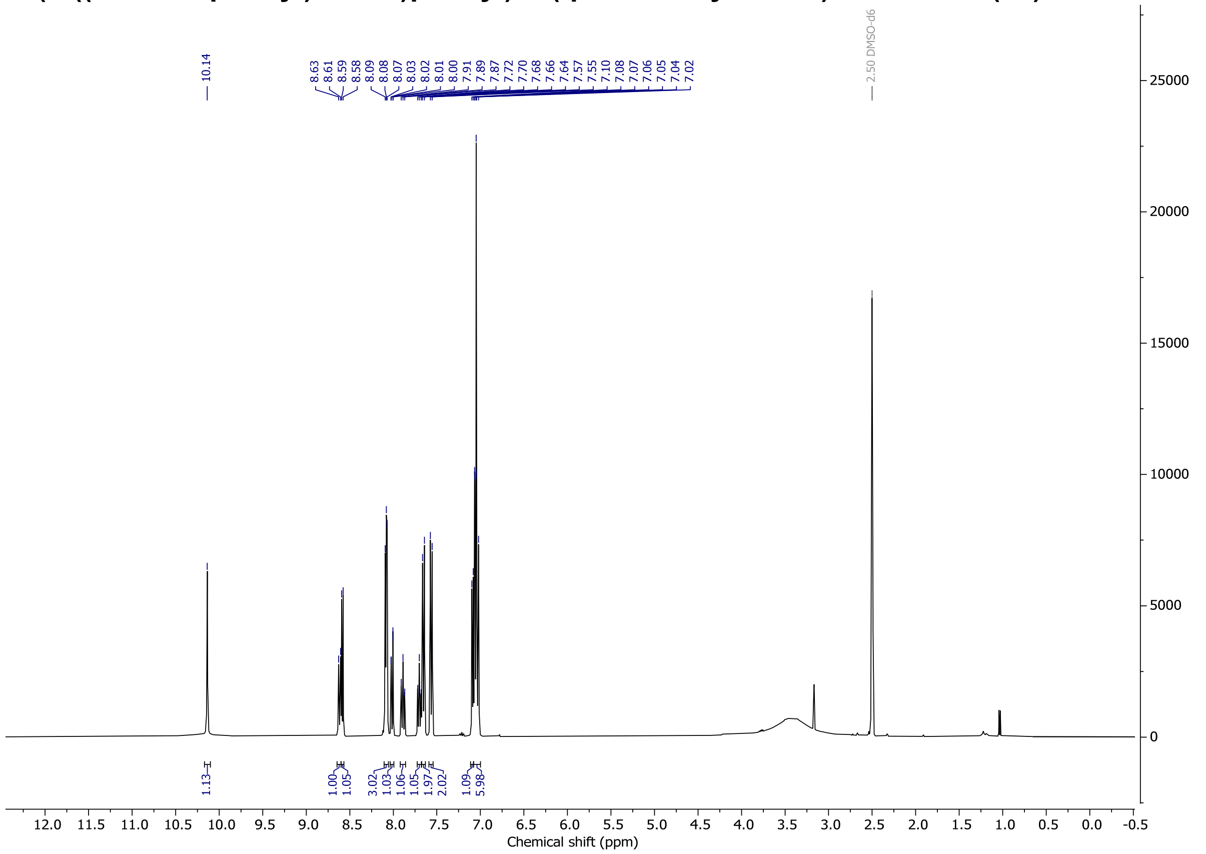

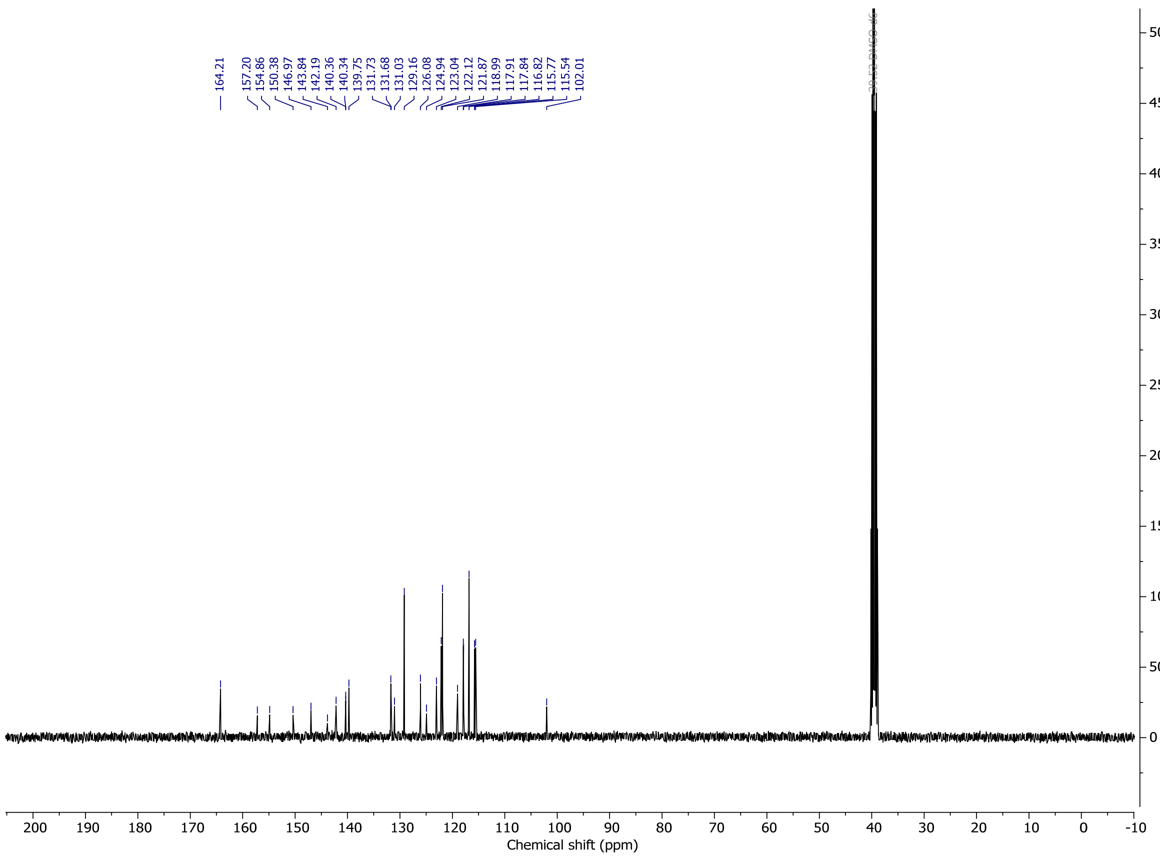

N-(4-(Pyridin-2-ylamino)phenyl)-4-(quinolin-4-ylamino)benzamide (34)

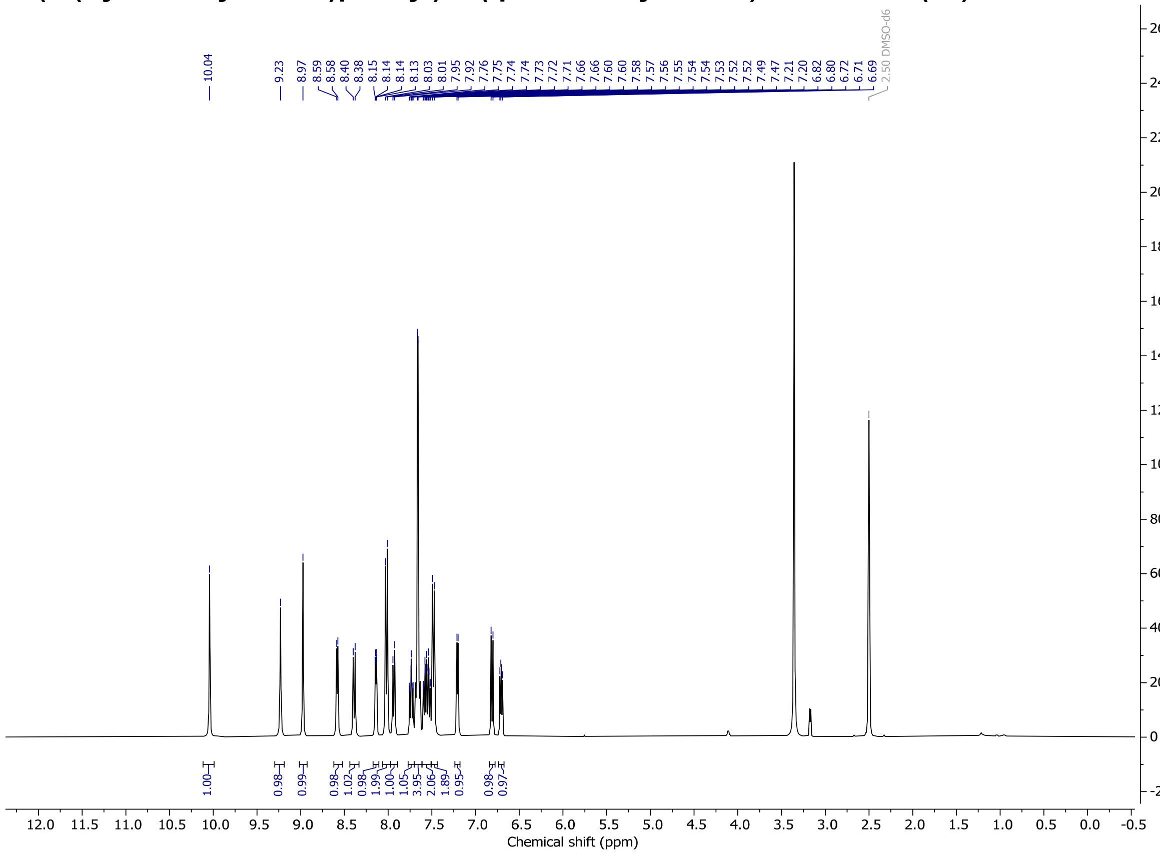

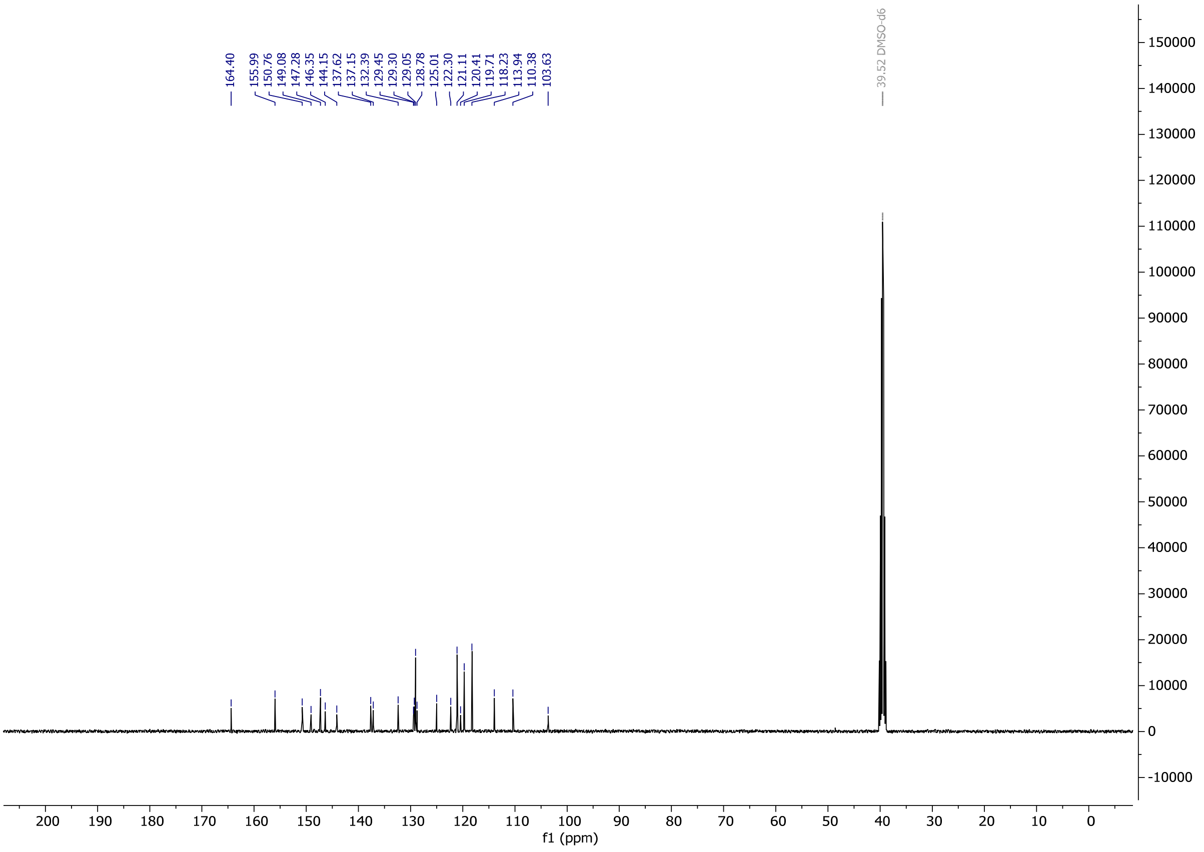

N-(4-((6-Aminopyridin-2-yl)amino)phenyl)-4-(quinolin-4-ylamino)benzamide (35)

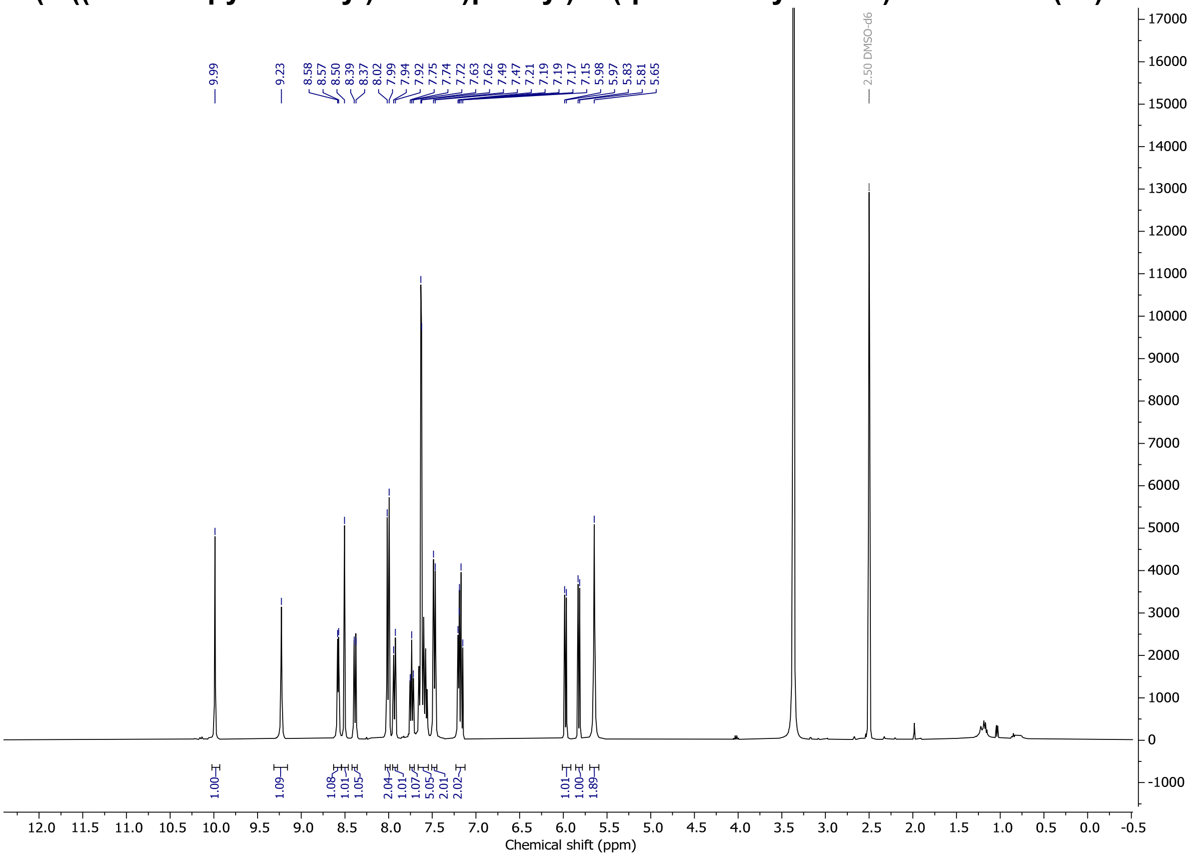

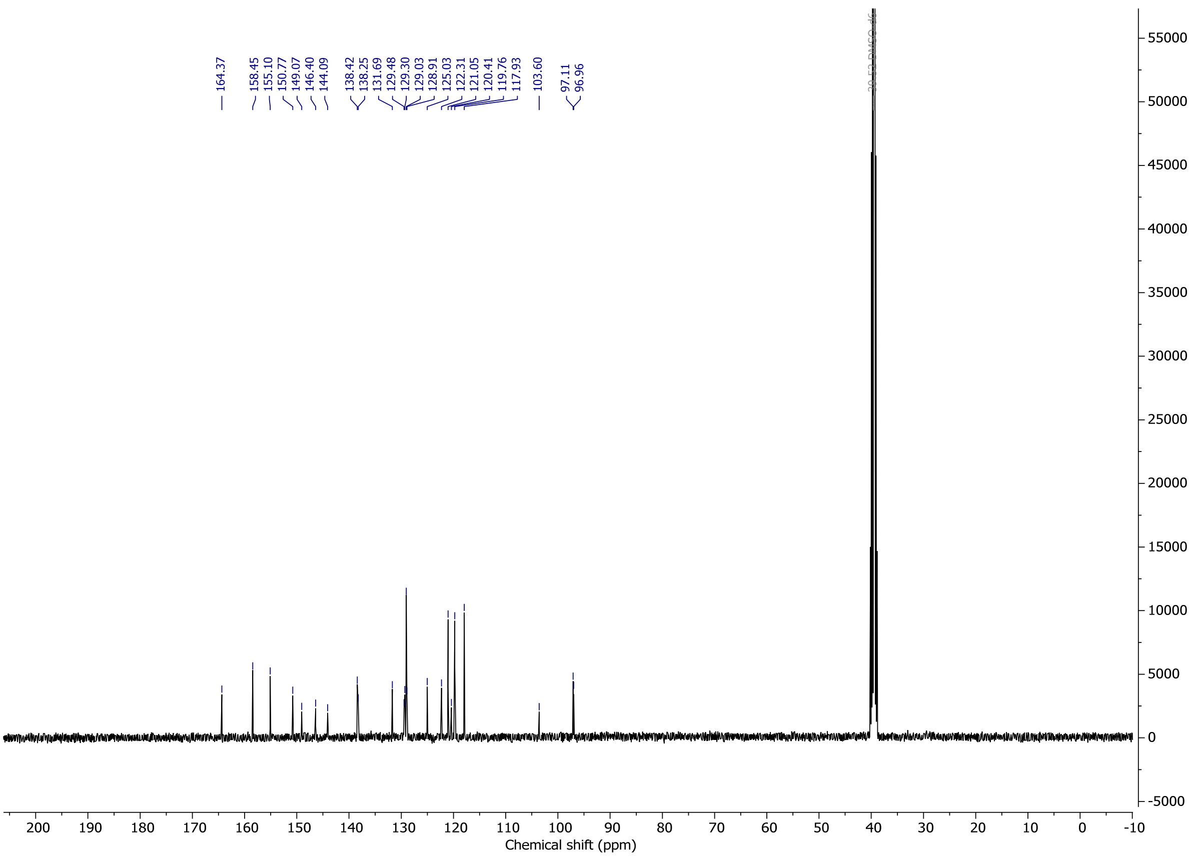

_N_-(4-((2-Aminopyridin-4-yl)amino)phenyl)-4-(quinolin-4-ylamino)benzamide (36)

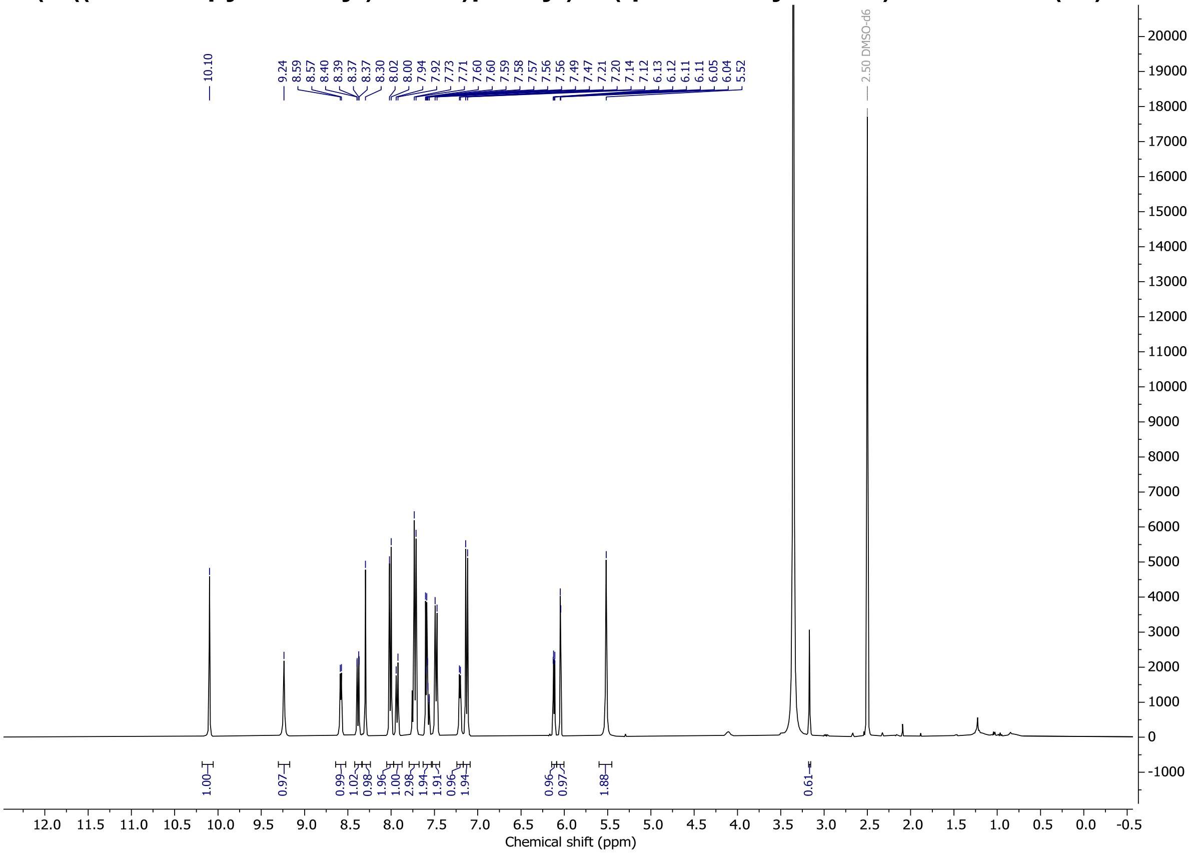

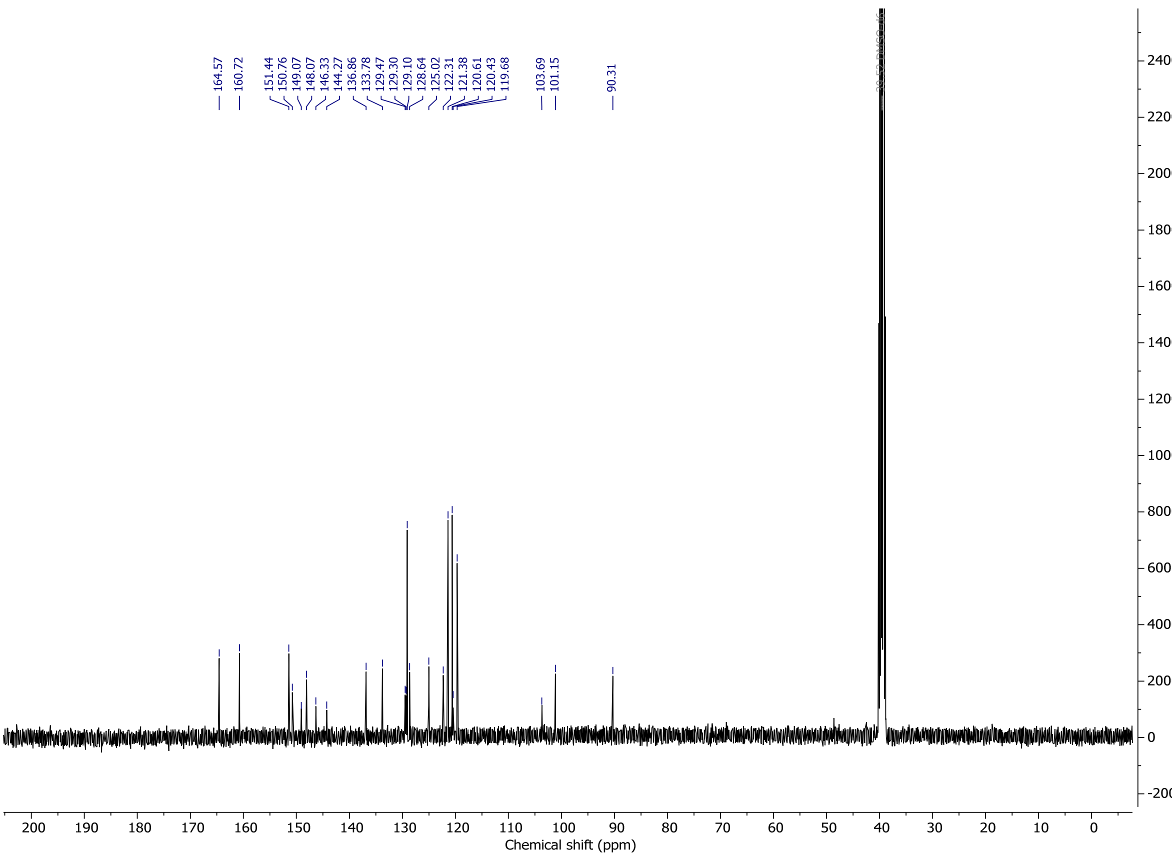

N-(4-((3-Hydroxyphenyl)amino)phenyl)-4-(quinolin-4-ylamino)benzamide (40)

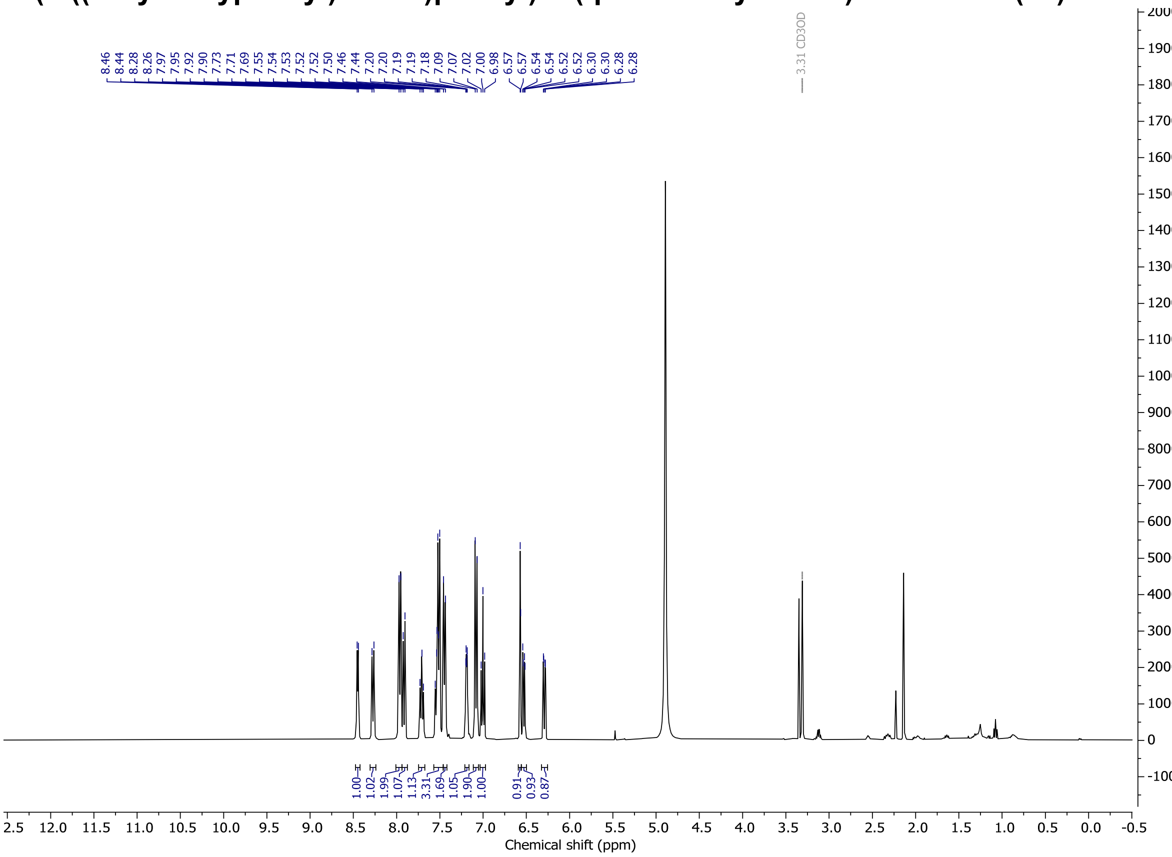

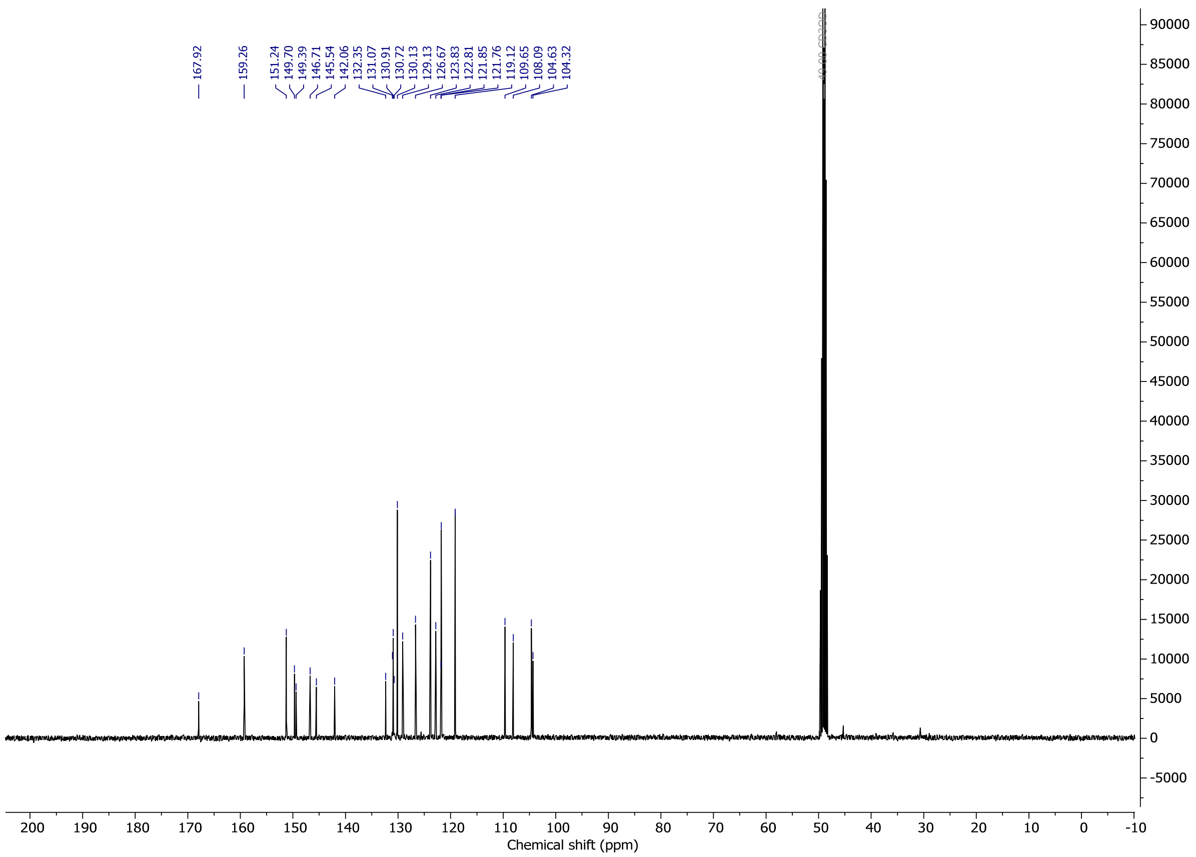

***tert*-Butyl (*tert*-butoxycarbonyl)(2-fluoro-5-((4-(4-(quinolin-4ylamino)benzamido)phenyl) amino)phenyl)carbamate (94)**

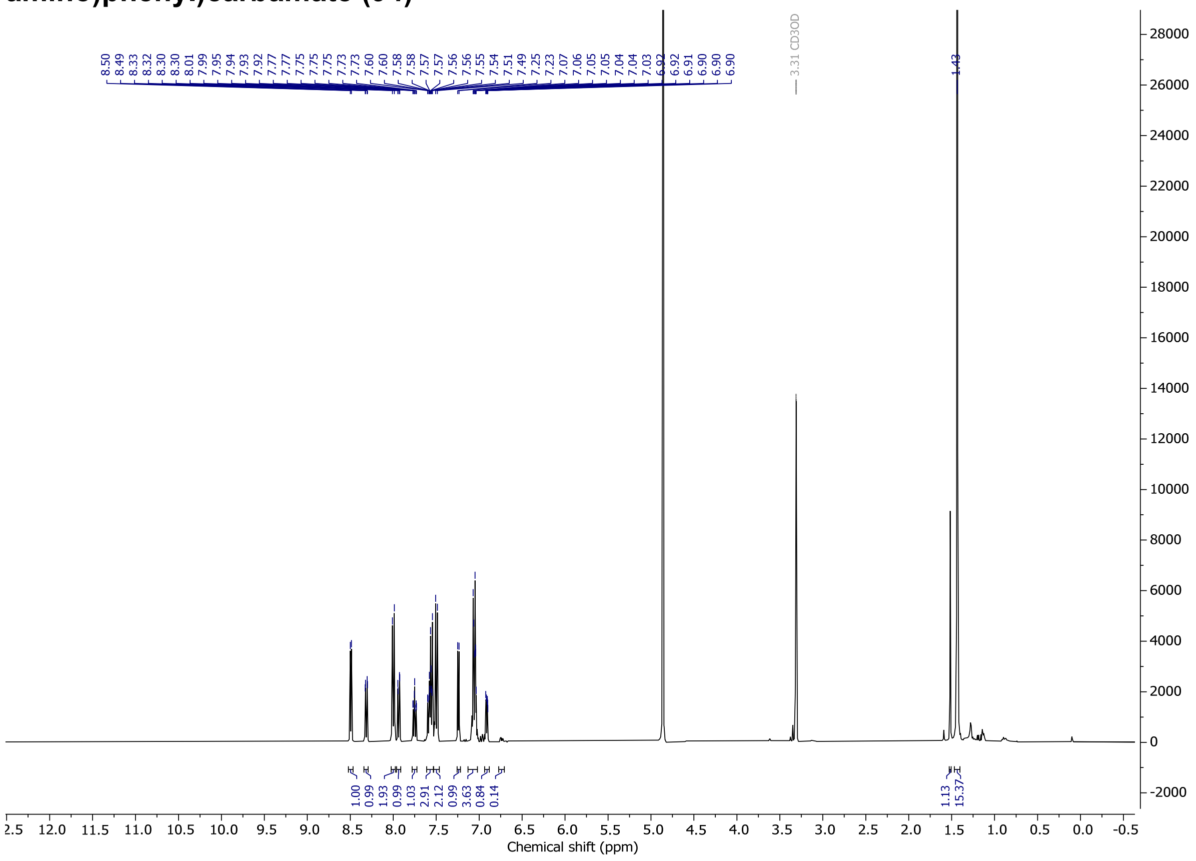

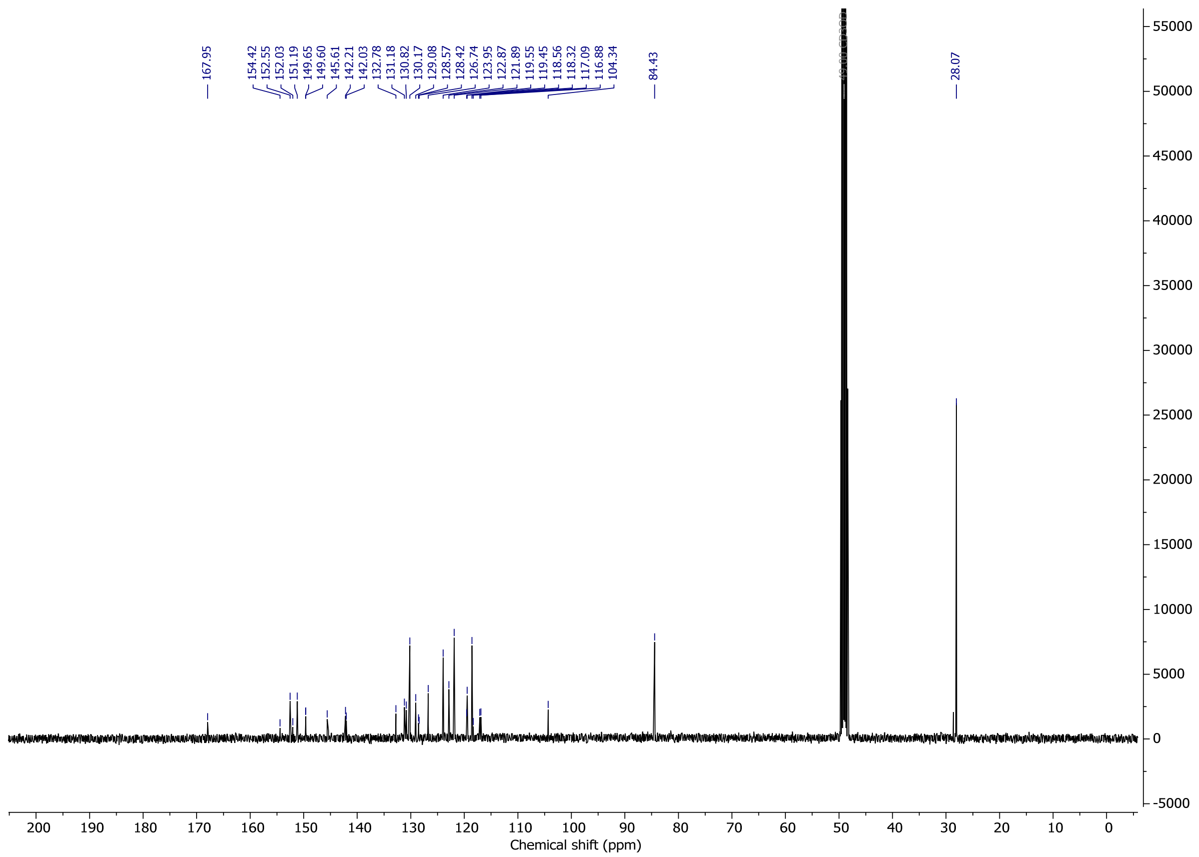

tert-Butyl (_tert_-butoxycarbonyl)(3-fluoro-5-((4-(4-(quinolin-4-ylamino)benzamido)phenyl)amino)phenyl)carbamate (95)

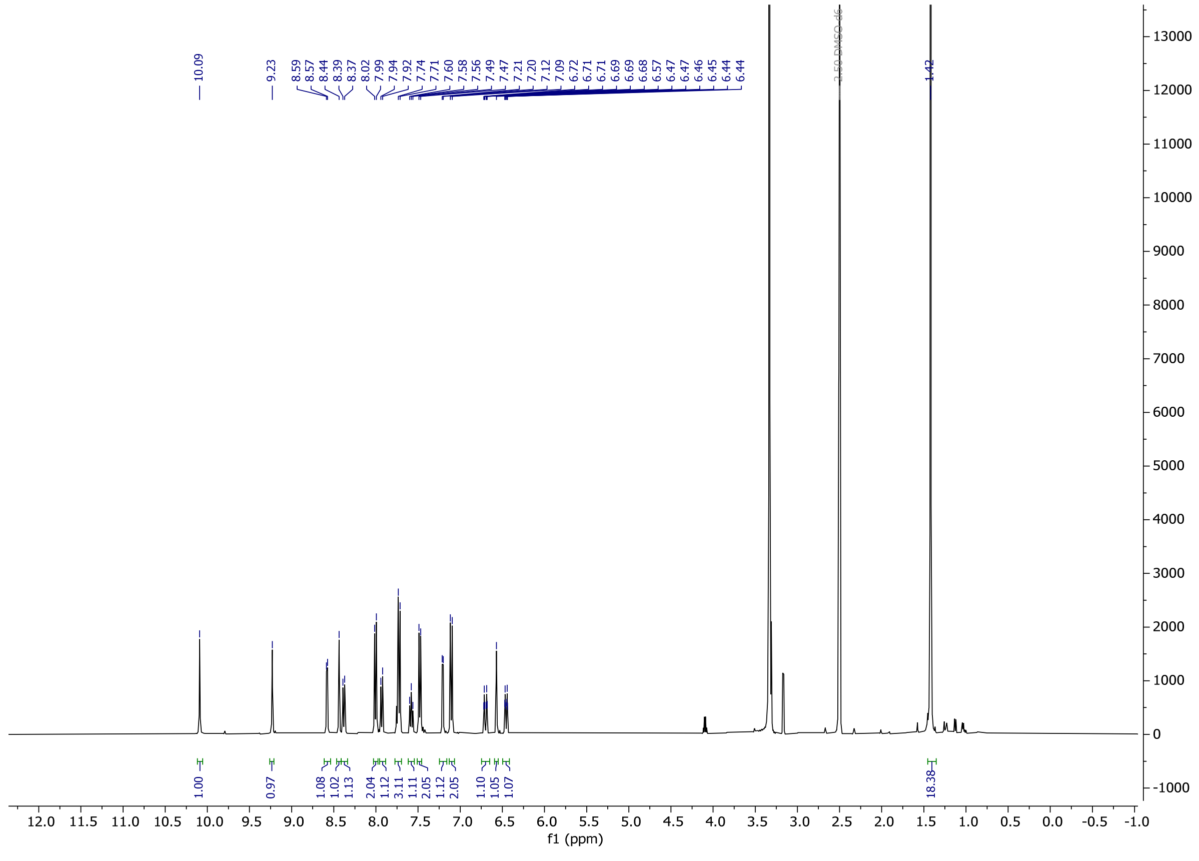

***N*-(4-((2-Amino-6-(trifluoromethyl)pyrimidin-4-yl)amino)phenyl)-4-(quinolin-4-ylamino) benzamide (41)**

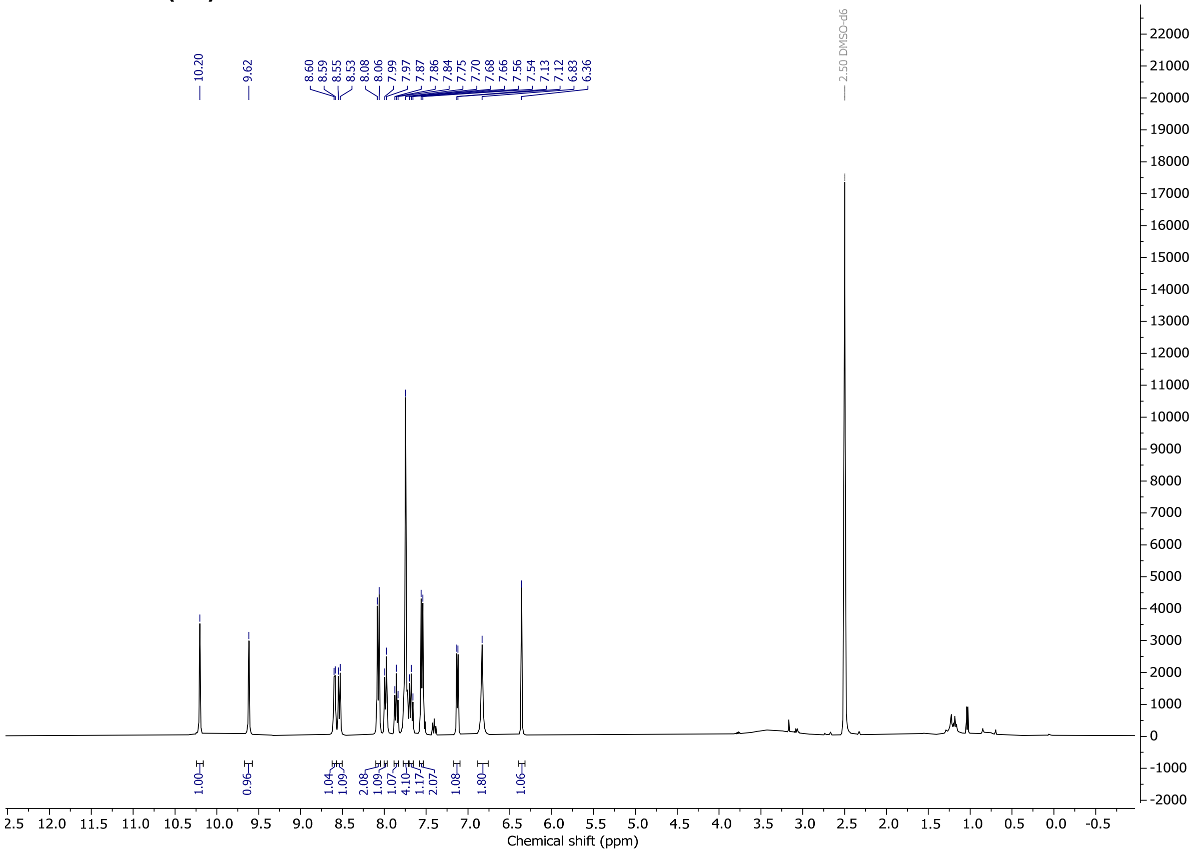

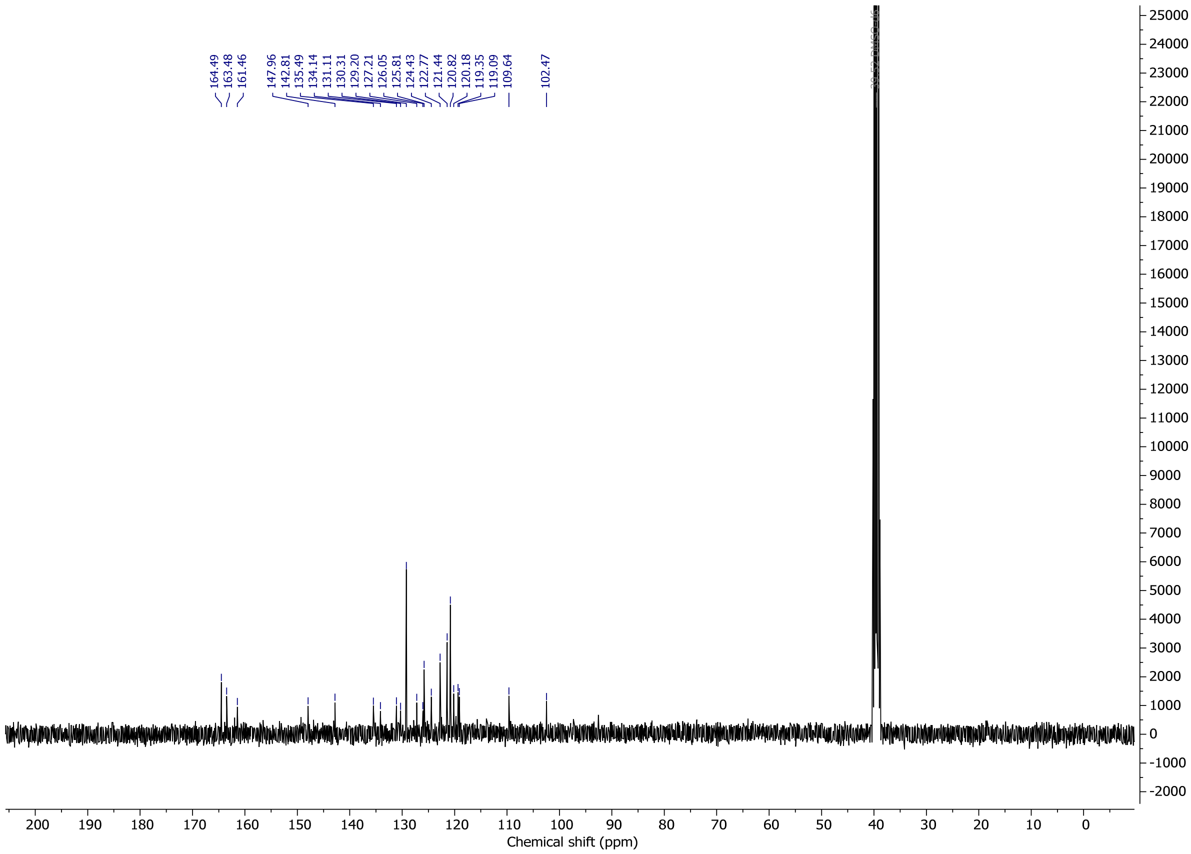

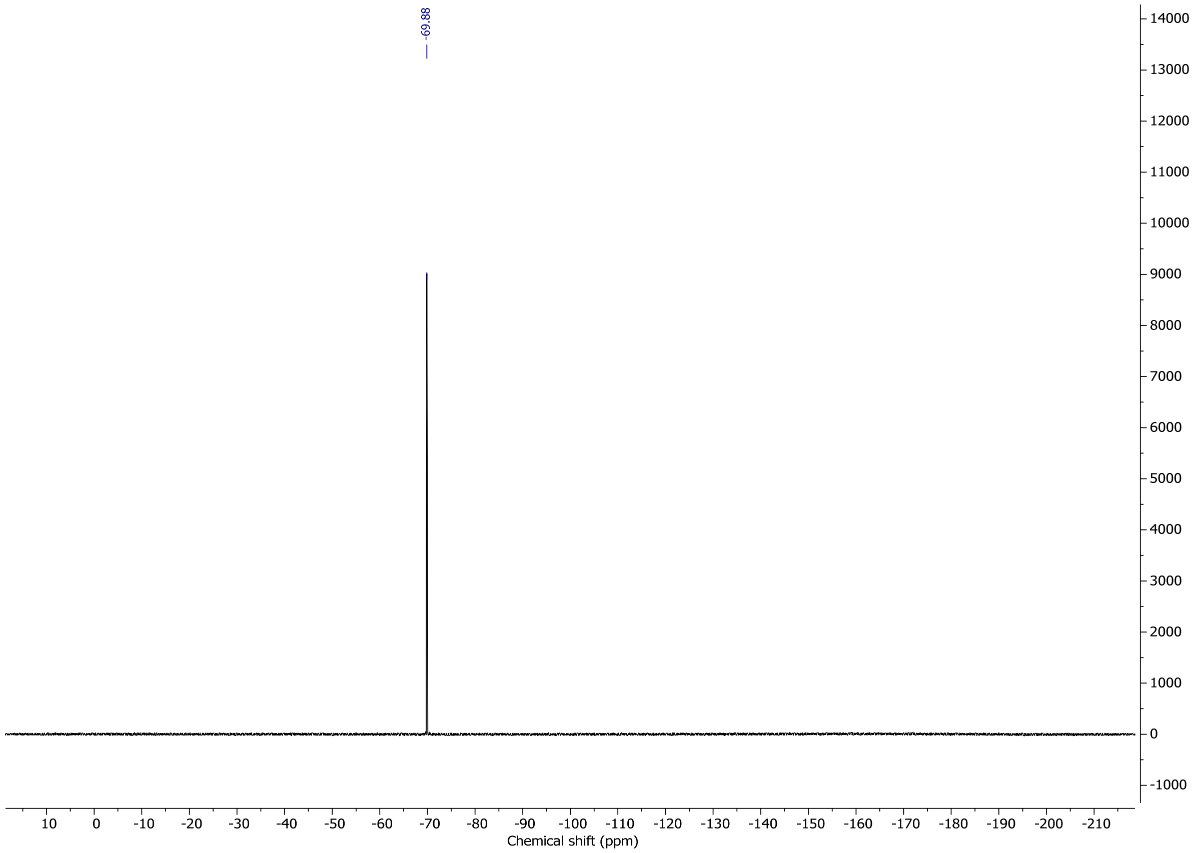

N-(4-((2-Aminopyrimidin-4-yl)amino)phenyl)-4-(quinazolin-4-ylamino)benzamide (47)

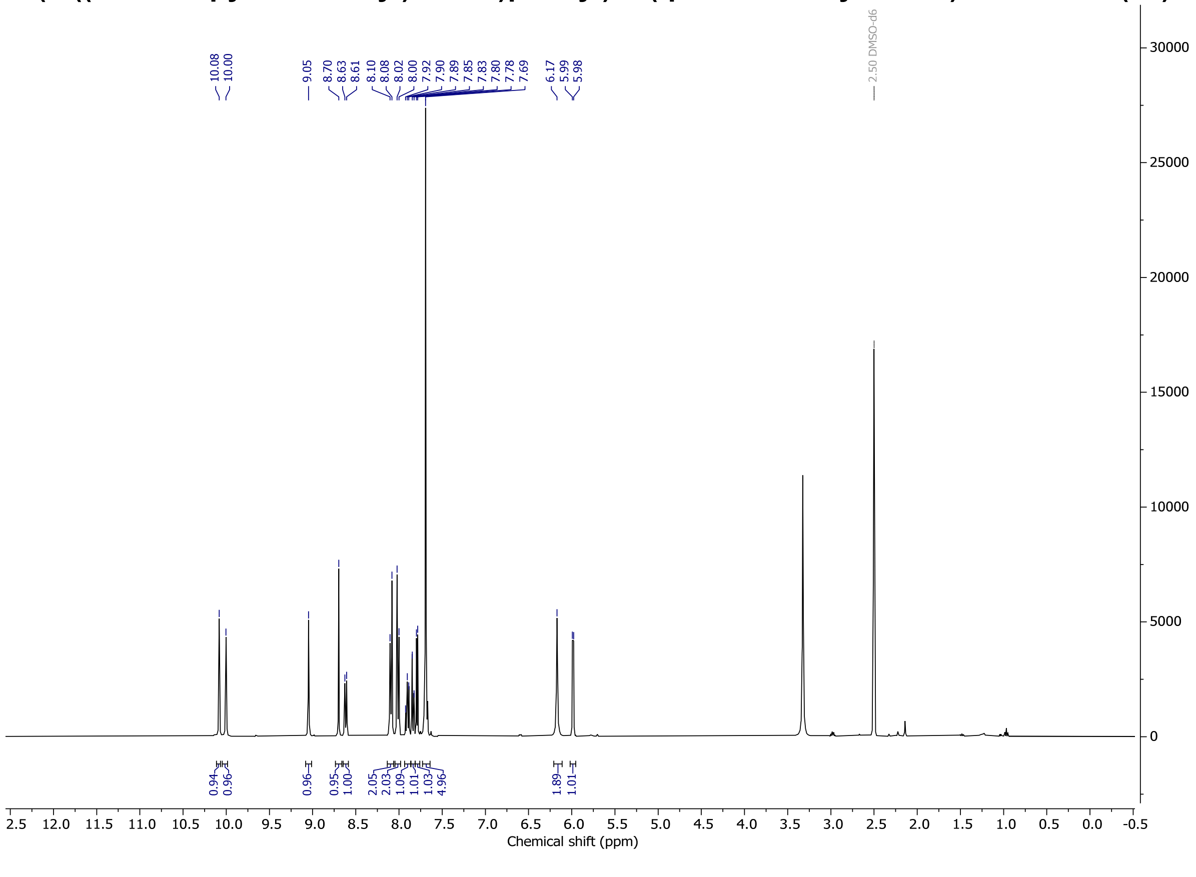

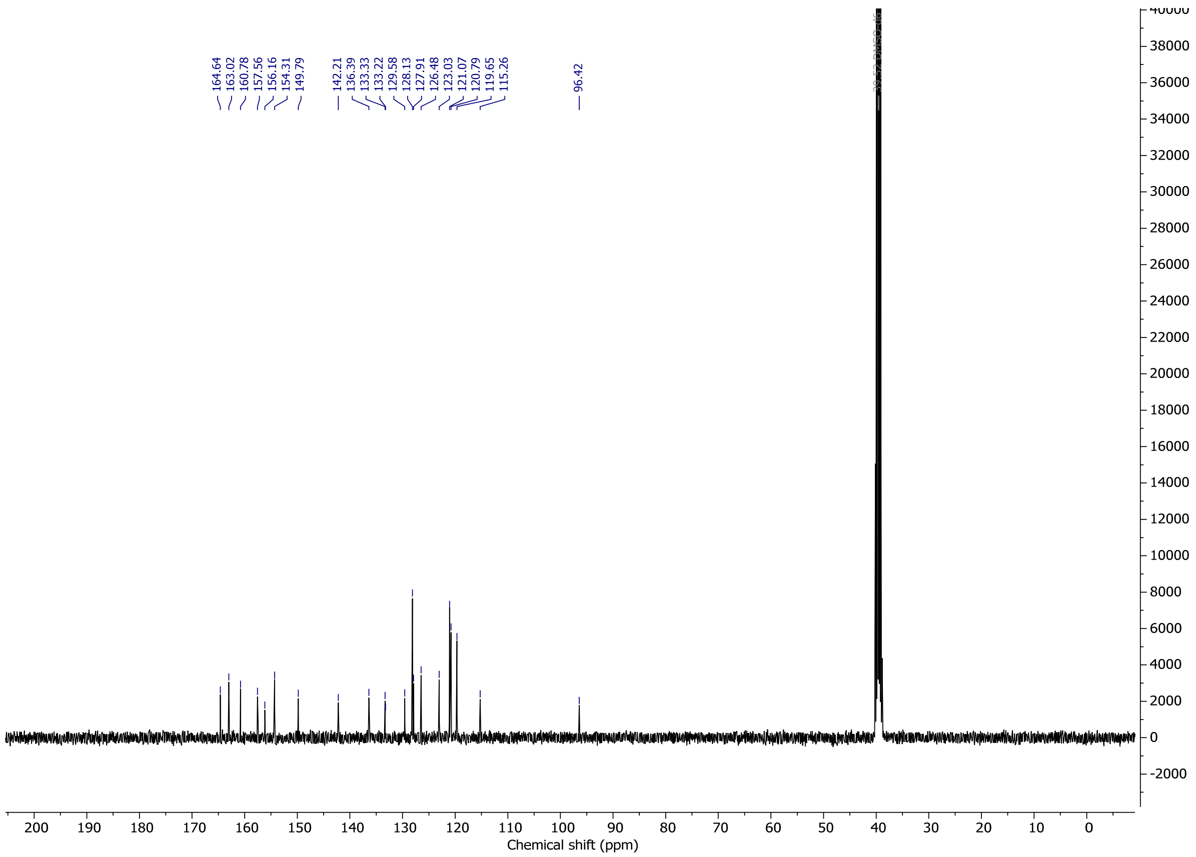

N-(4-((2-(Methylamino)pyrimidin-4-yl)amino)phenyl)-4-(quinolin-4-ylamino)benzamide (42)

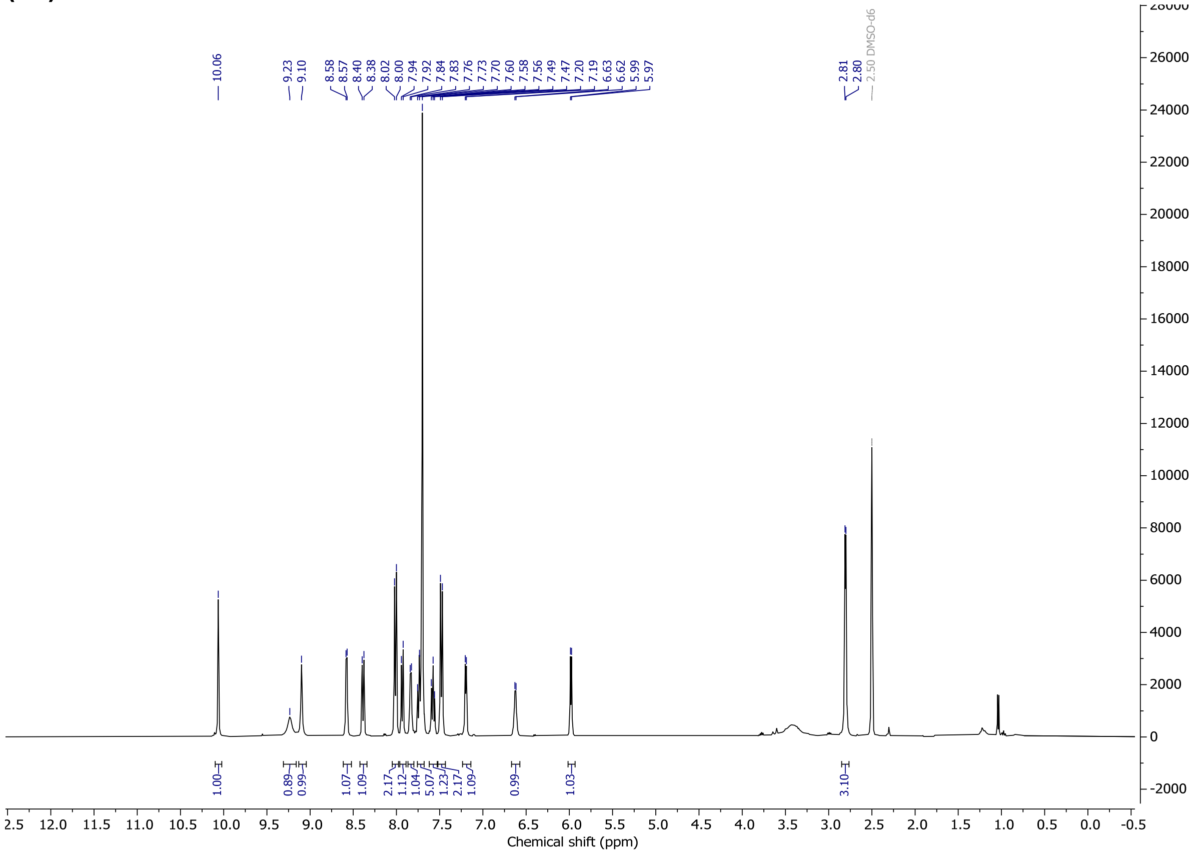

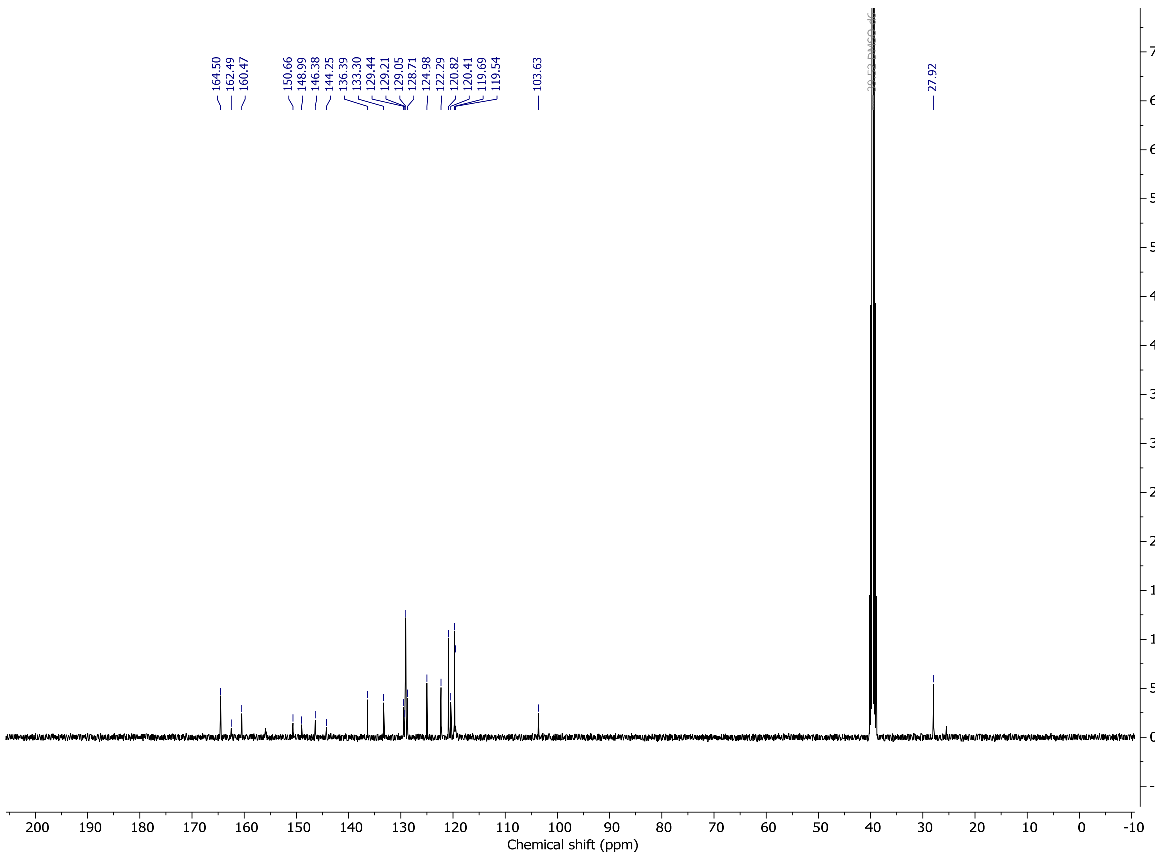

N-(4-((3-Nitrophenyl)amino)phenyl)-4-(quinolin-4-ylamino)benzamide (86)

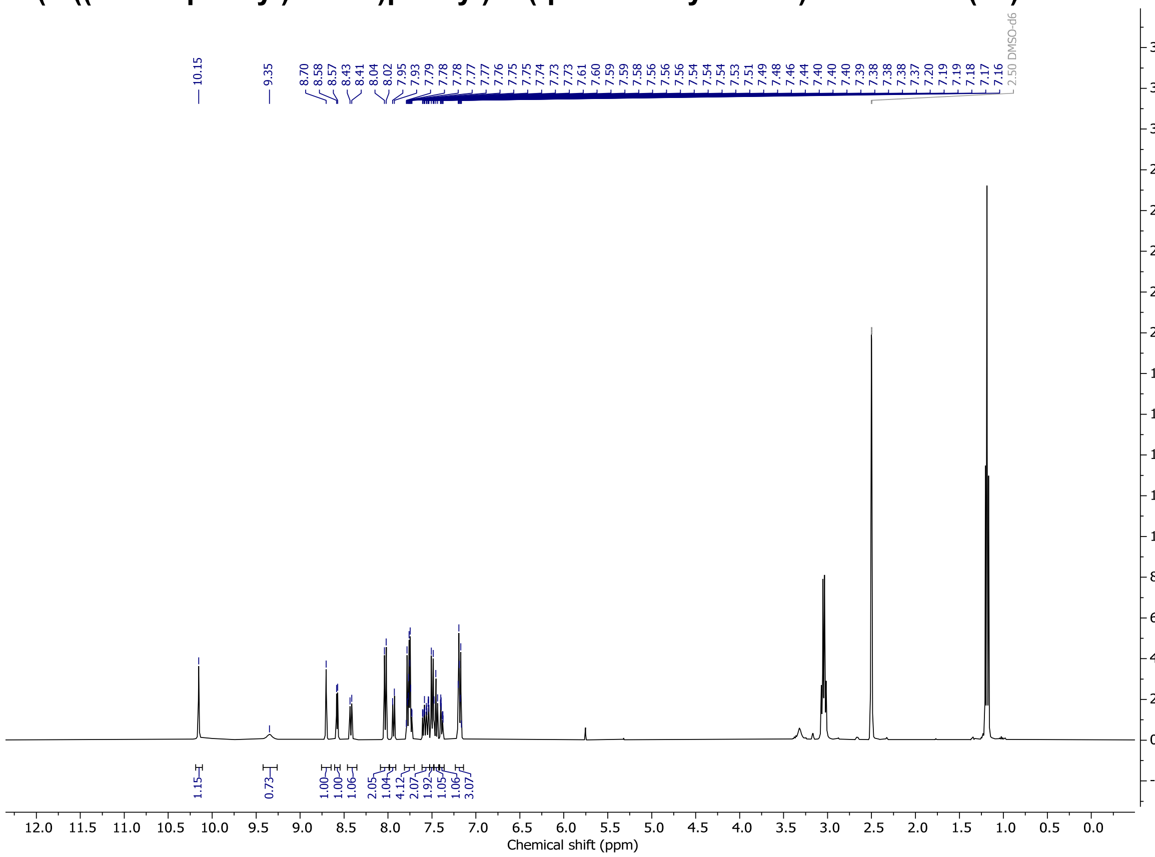

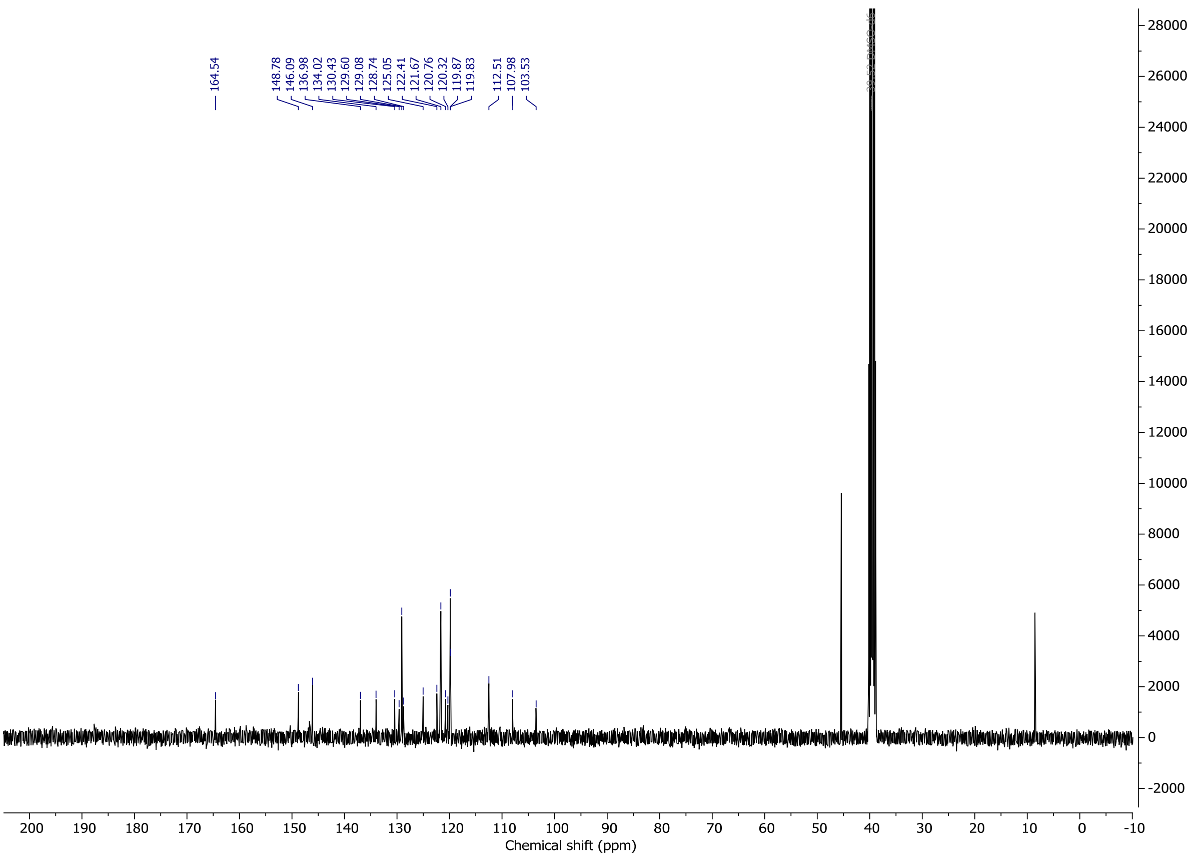

N-(4-((3-Methyl-5-nitrophenyl)amino)phenyl)-4-(quinolin-4-ylamino)benzamide (87)

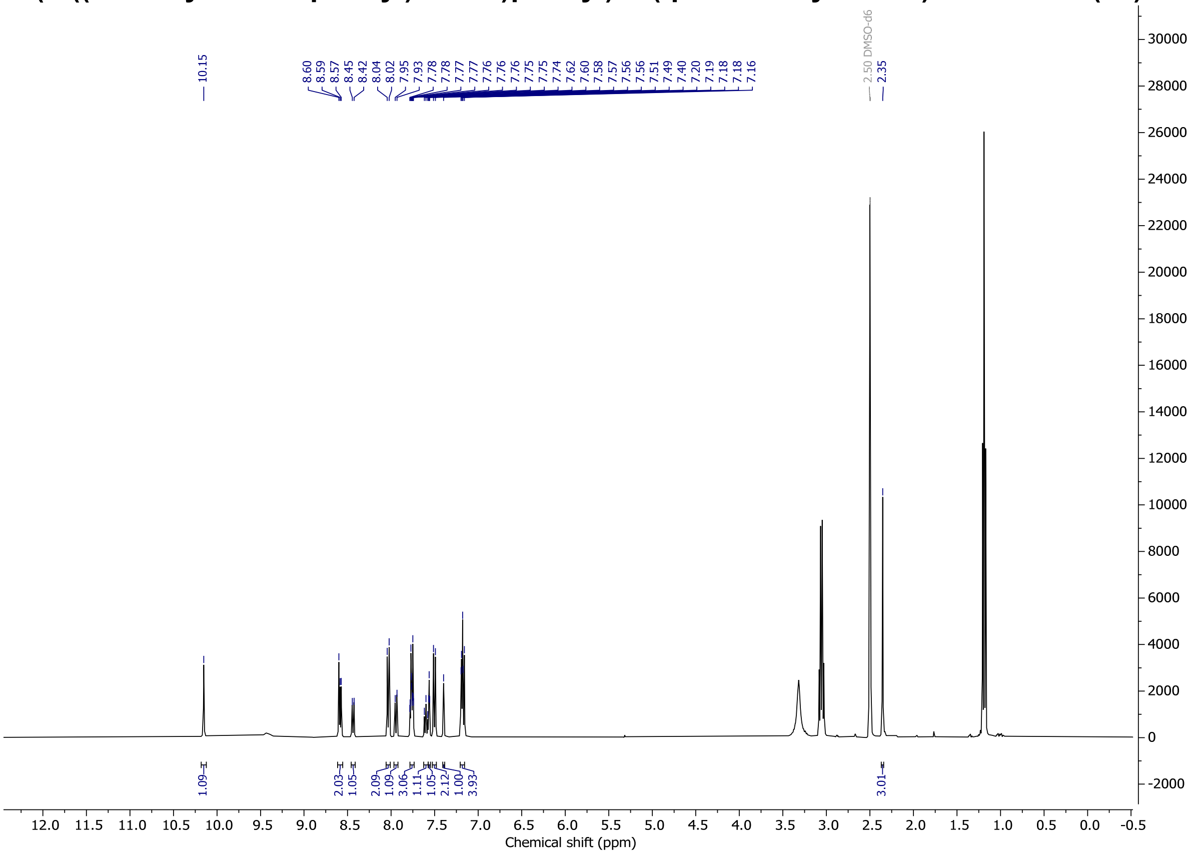

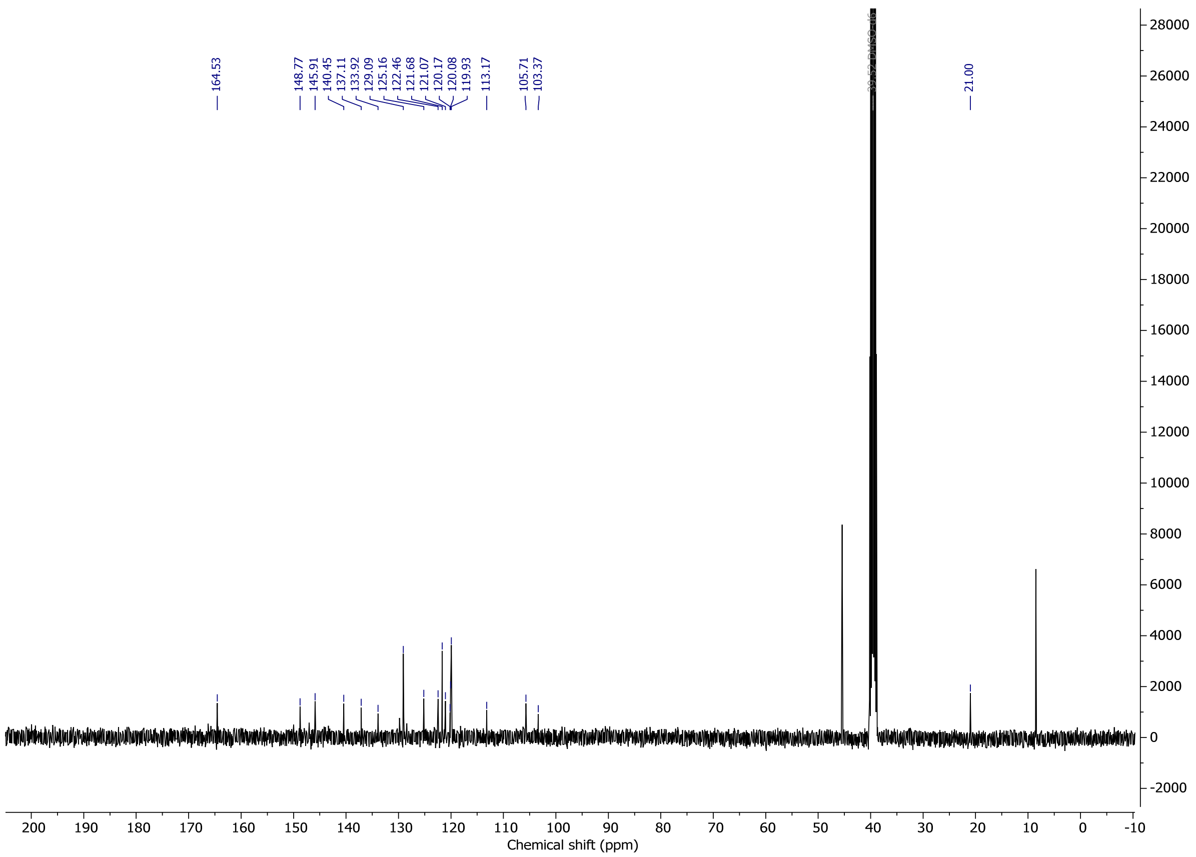

N-(4-((2-Amino-6-methylpyrimidin-4-yl)amino)phenyl)-4-(naphthalen-1-ylamino)benzamide (44)

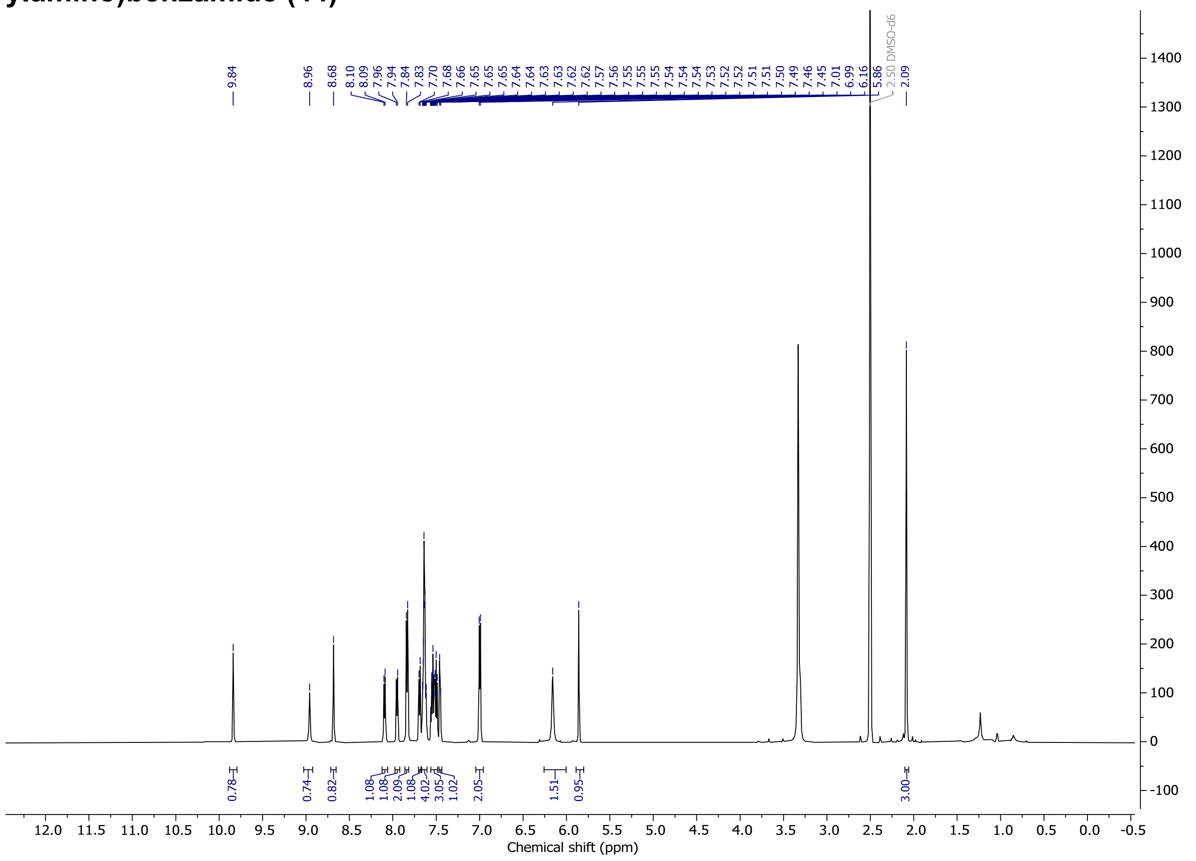

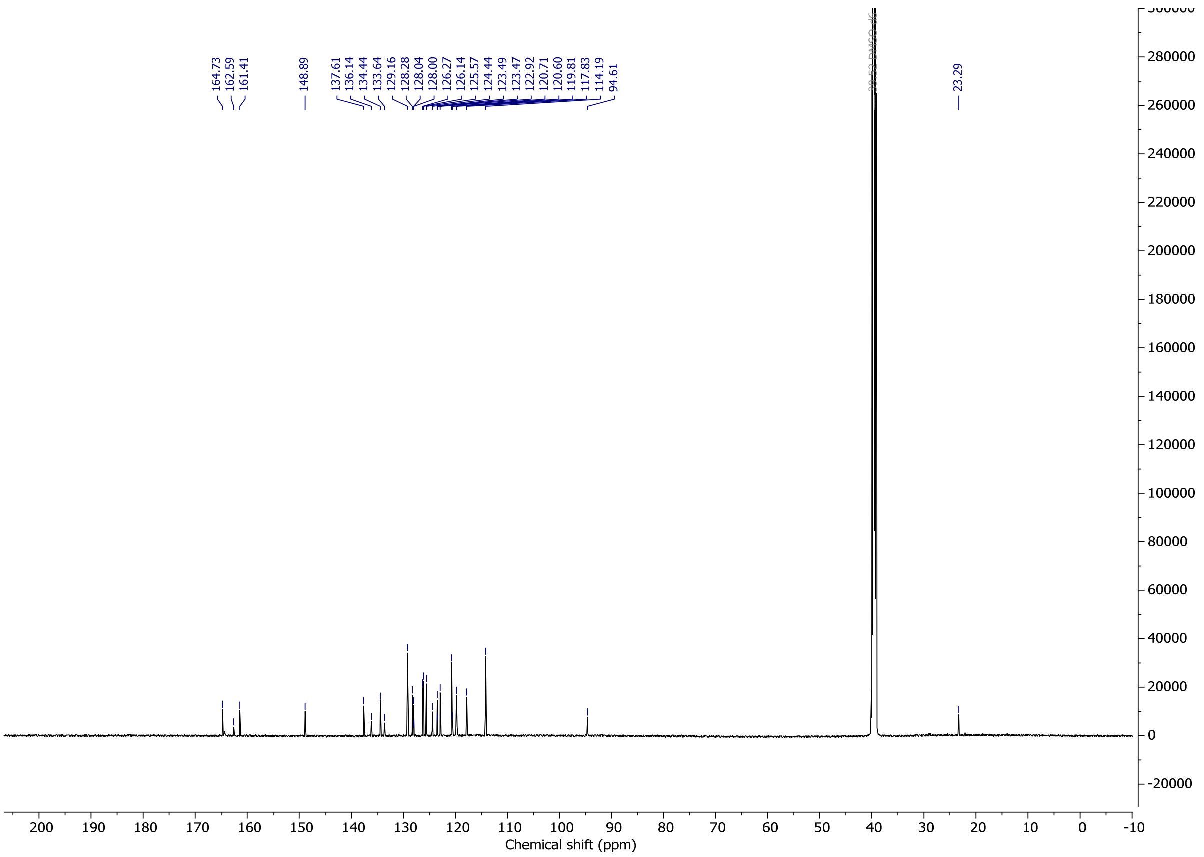

N-(4-((2-Amino-6-methylpyrimidin-4-yl)amino)phenyl)-4-(quinazolin-4-ylamino)benzamide (45)

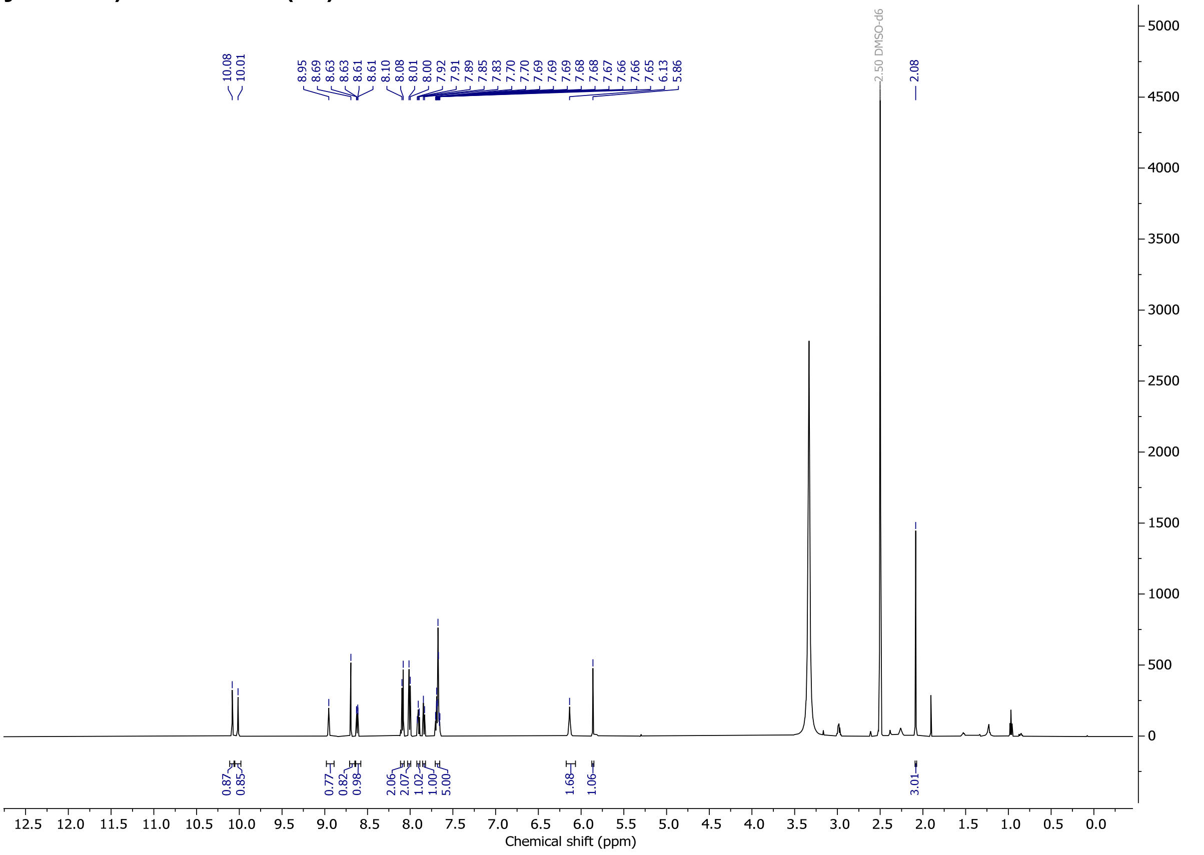

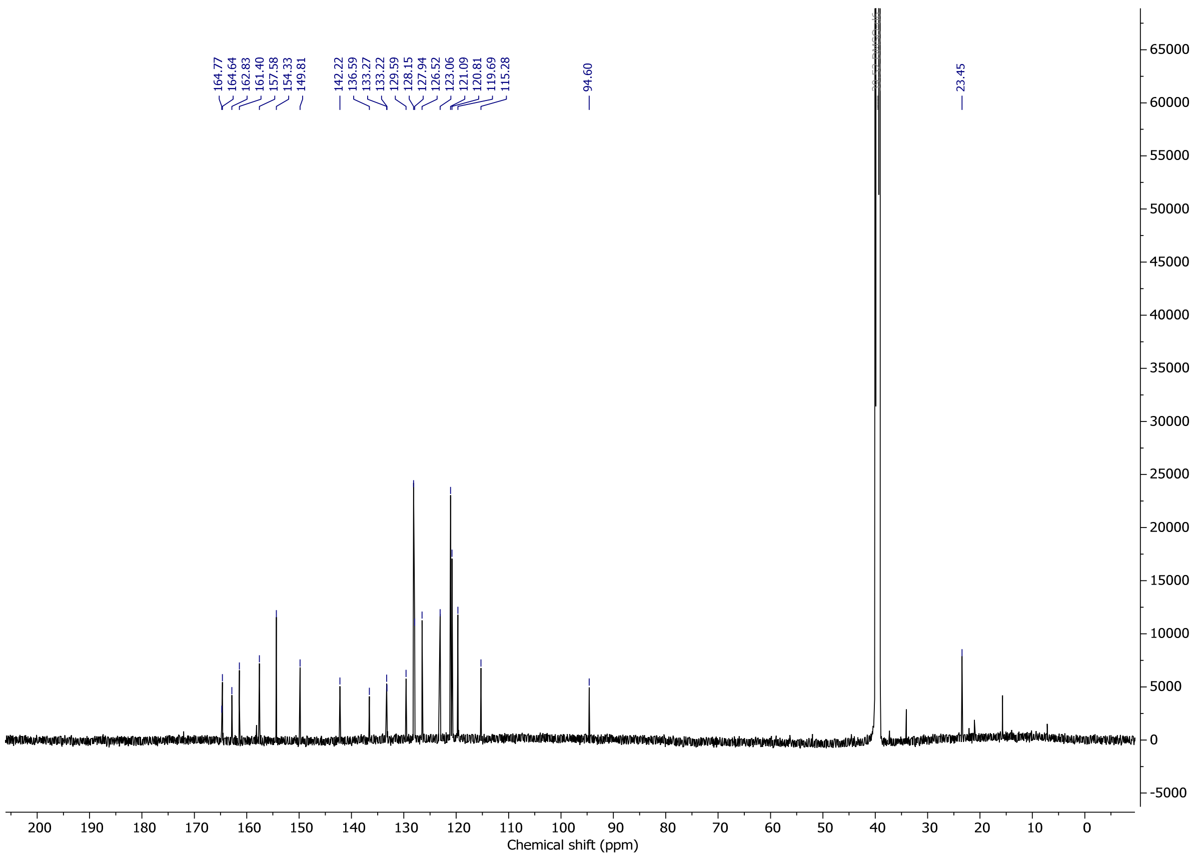

N-(4-((2-Amino-6-methylpyrimidin-4-yl)amino)phenyl)-4-((3a,7a-dihydro-1H-pyrrolo[2,3-b]pyridin-4-yl)amino)benzamide (46)

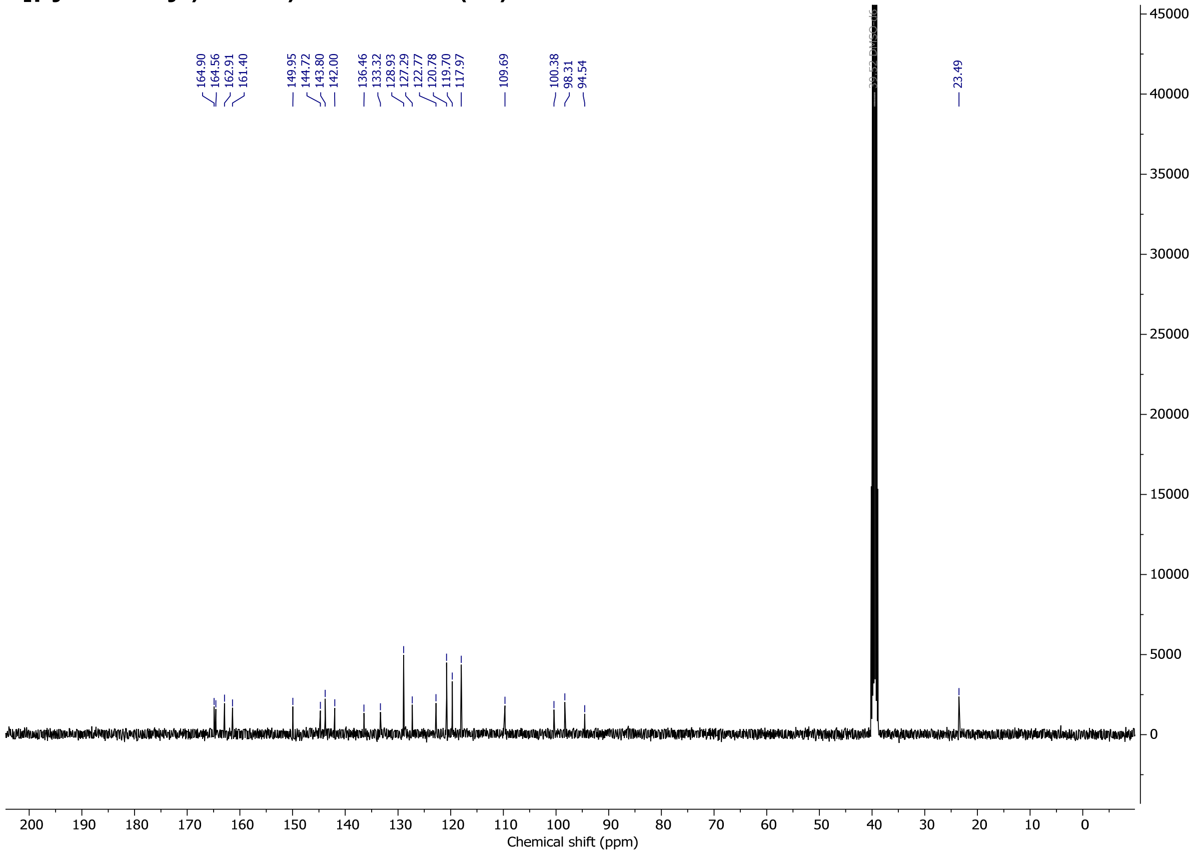

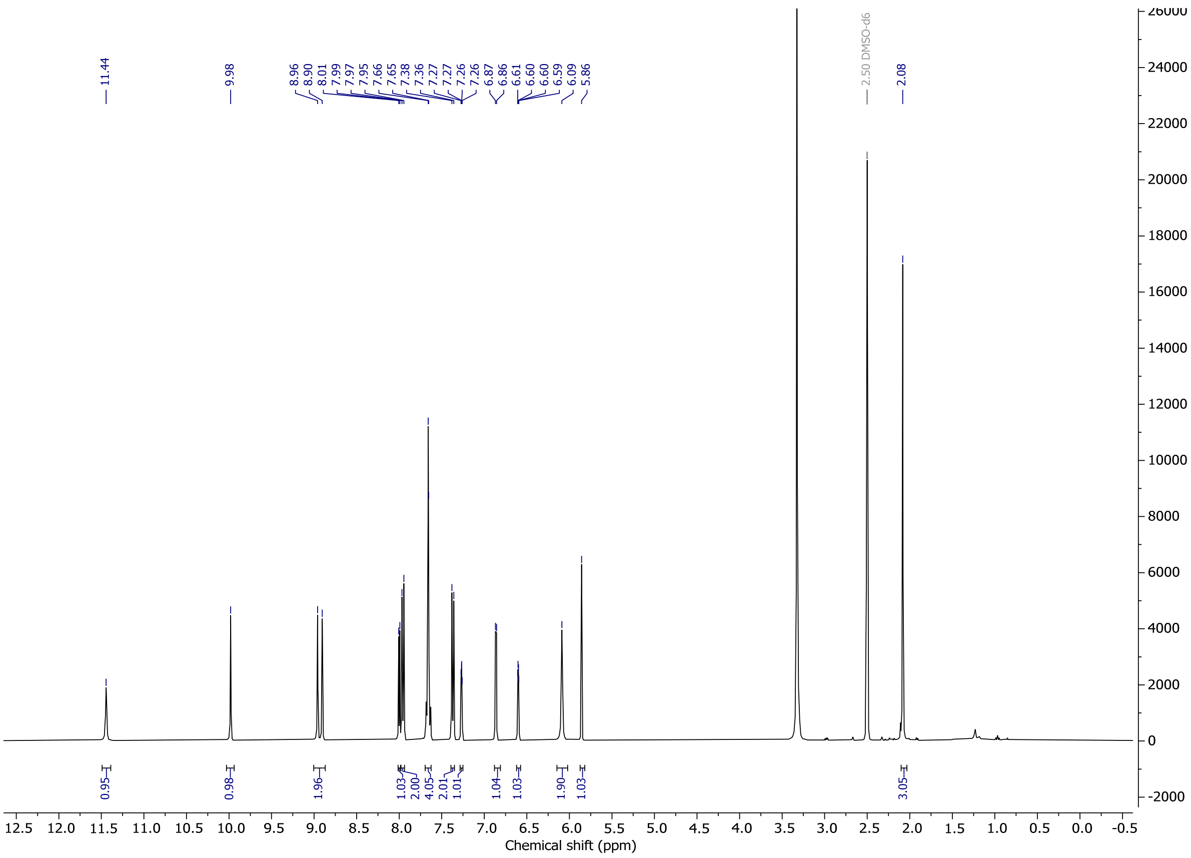

N-(4-((3-Amino-5-fluorophenyl)amino)phenyl)-4-(quinolin-4-ylamino)benzamide (38)

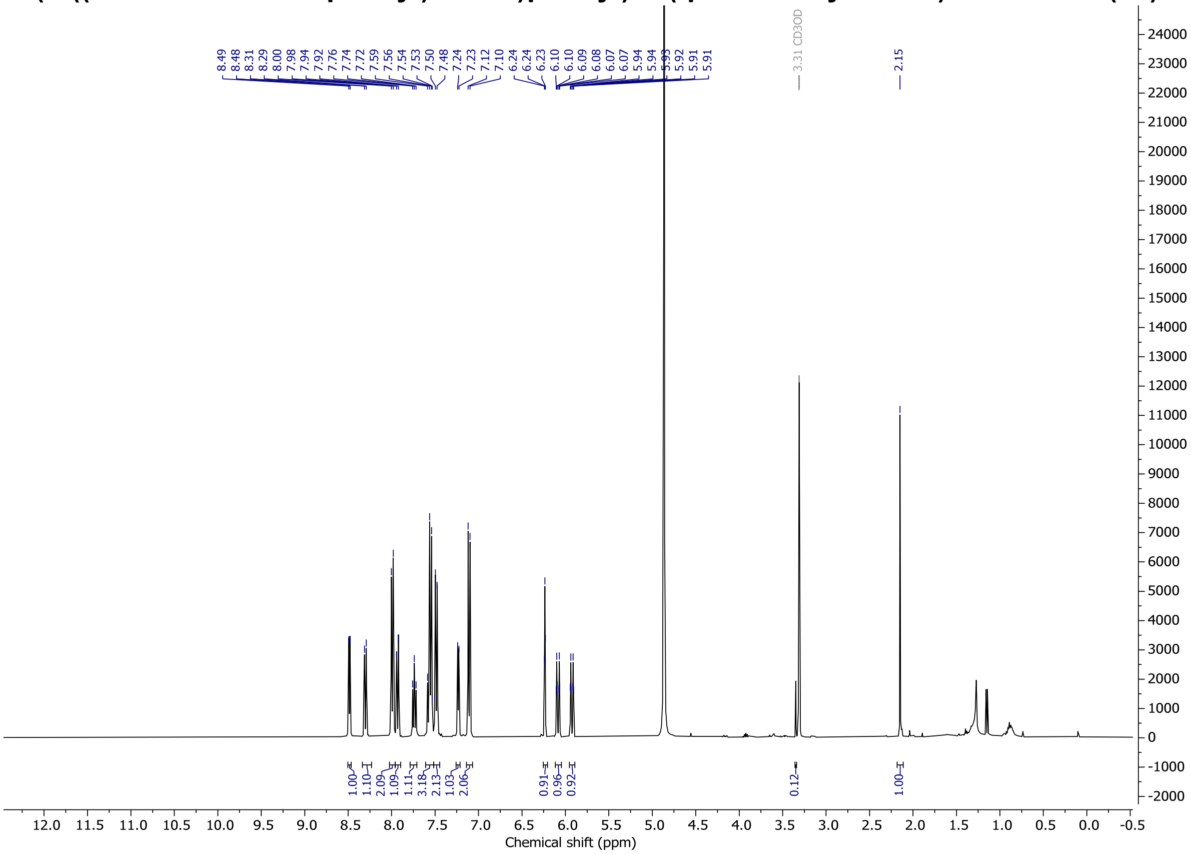

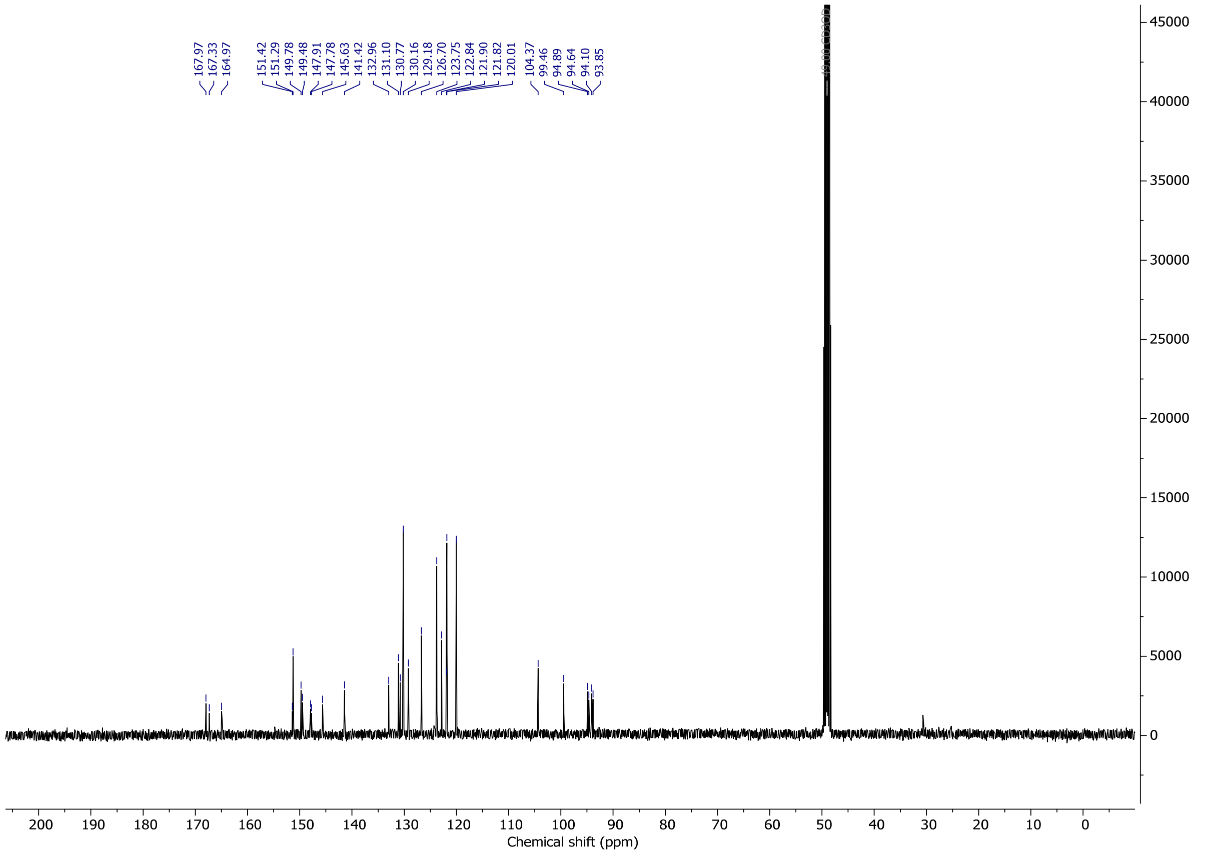

N-(4-((3-Amino-4-fluorophenyl)amino)phenyl)-4-(quinolin-4-ylamino)benzamide (37)

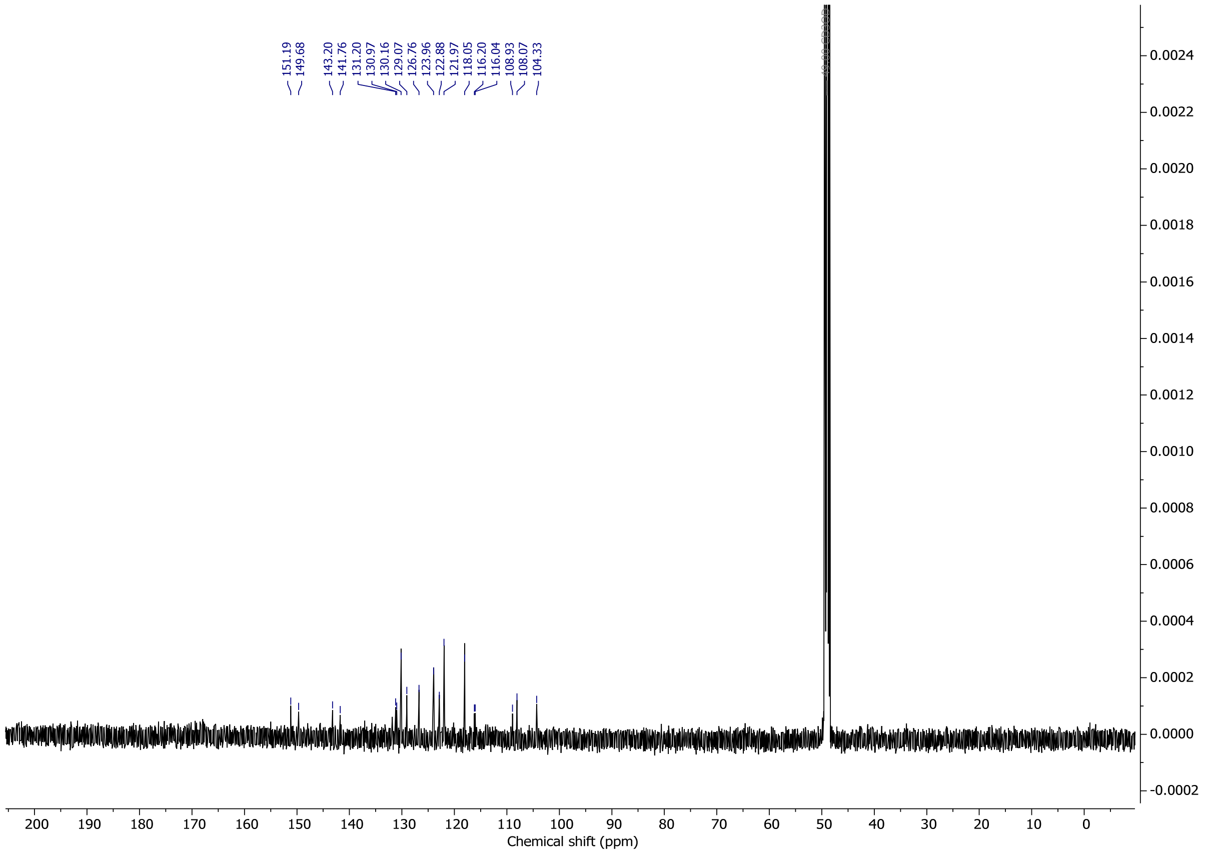

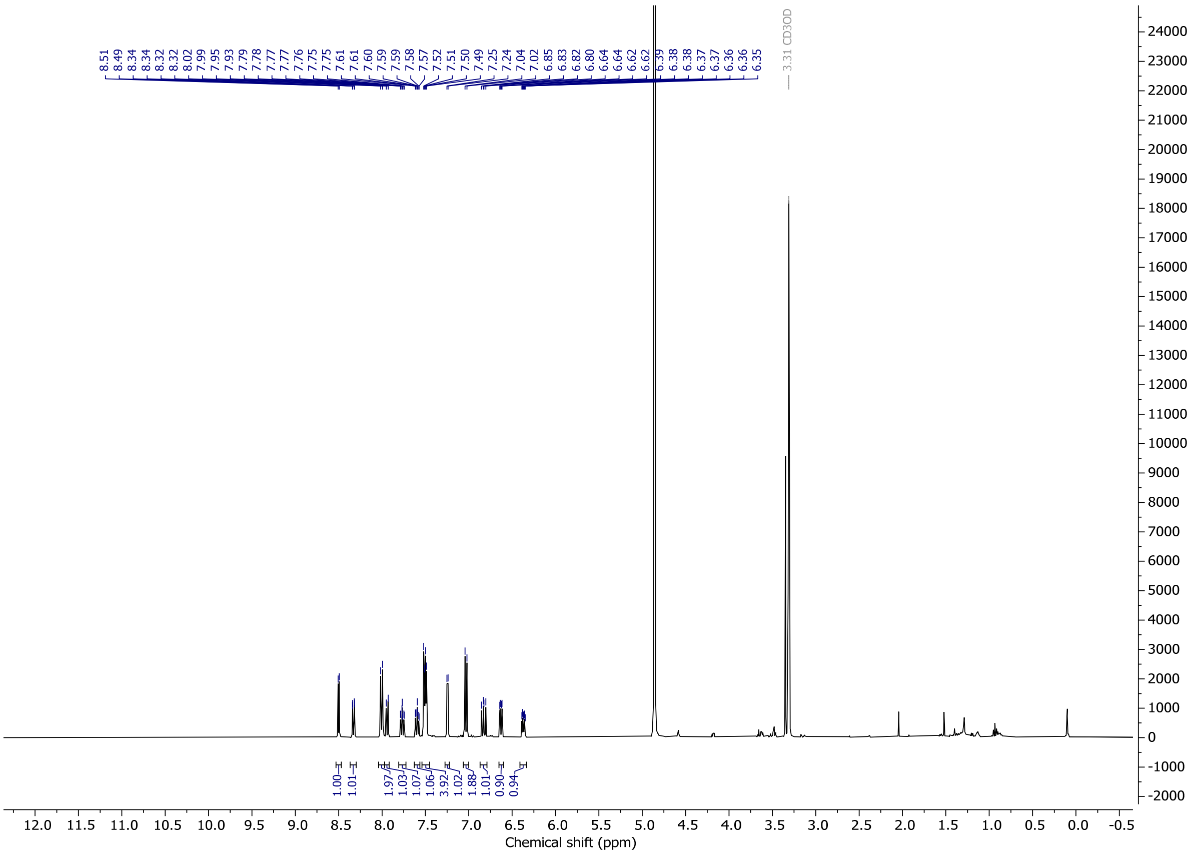

N^4-(4-Aminophenyl)-N^2-methylpyrimidine-2,4-diamine (96)

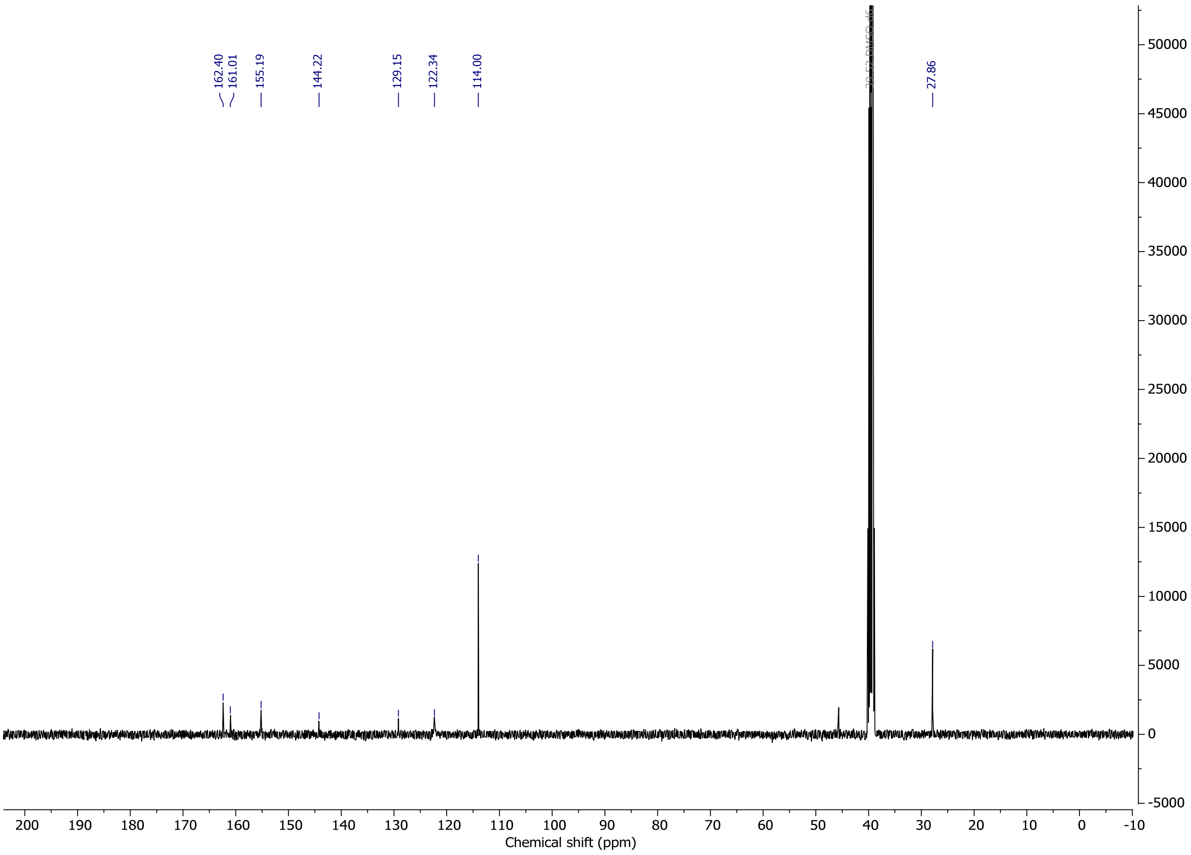

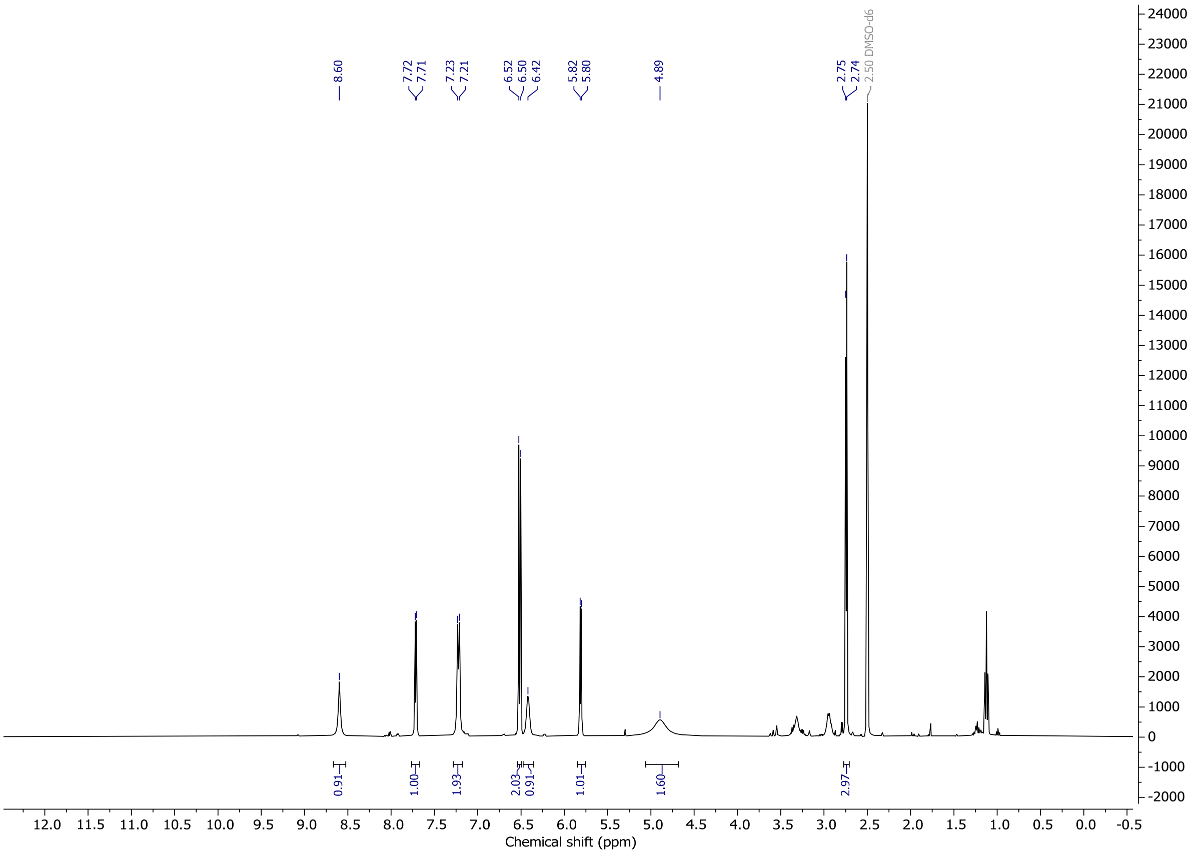

N-(4-Aminophenyl)-4-(quinolin-4-ylamino)benzamide (22)

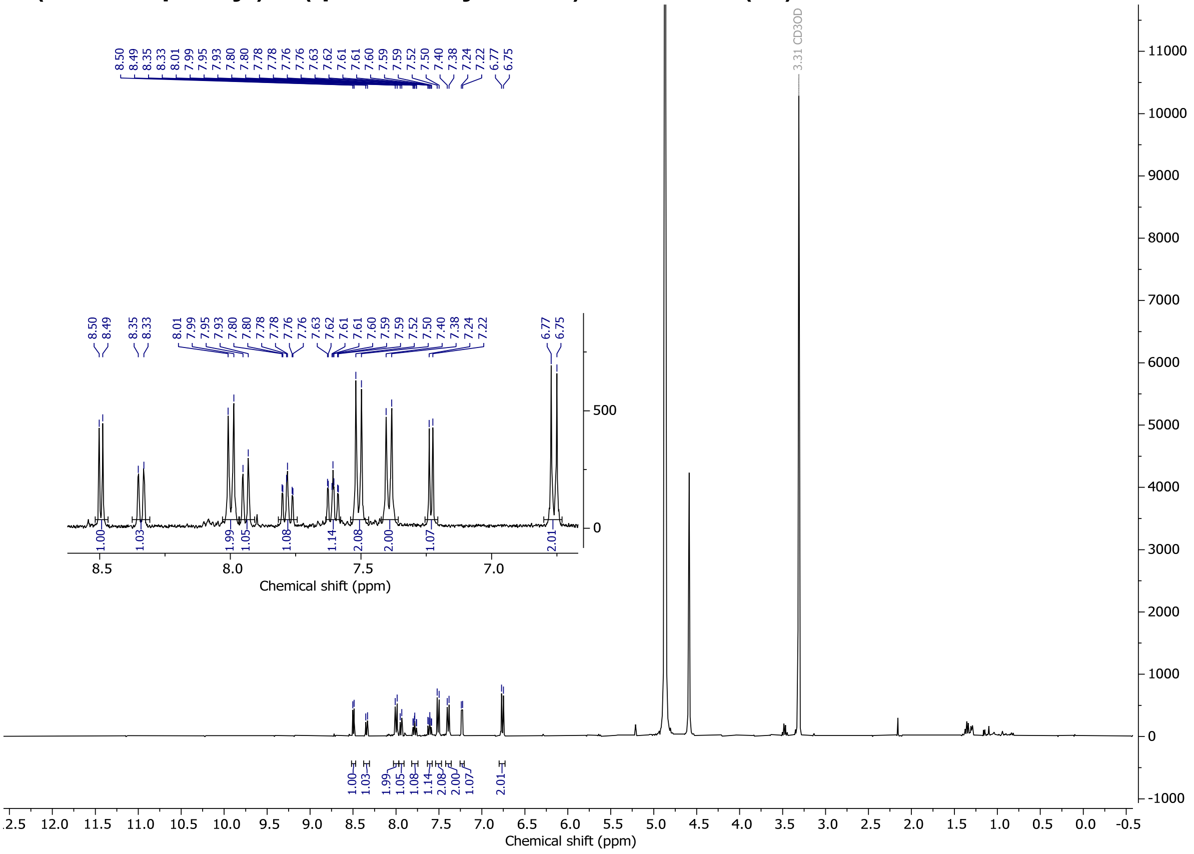

N-(4-((2-Amino-6-methylpyrimidin-4-yl)amino)phenyl)-4-nitrobenzamide (24)

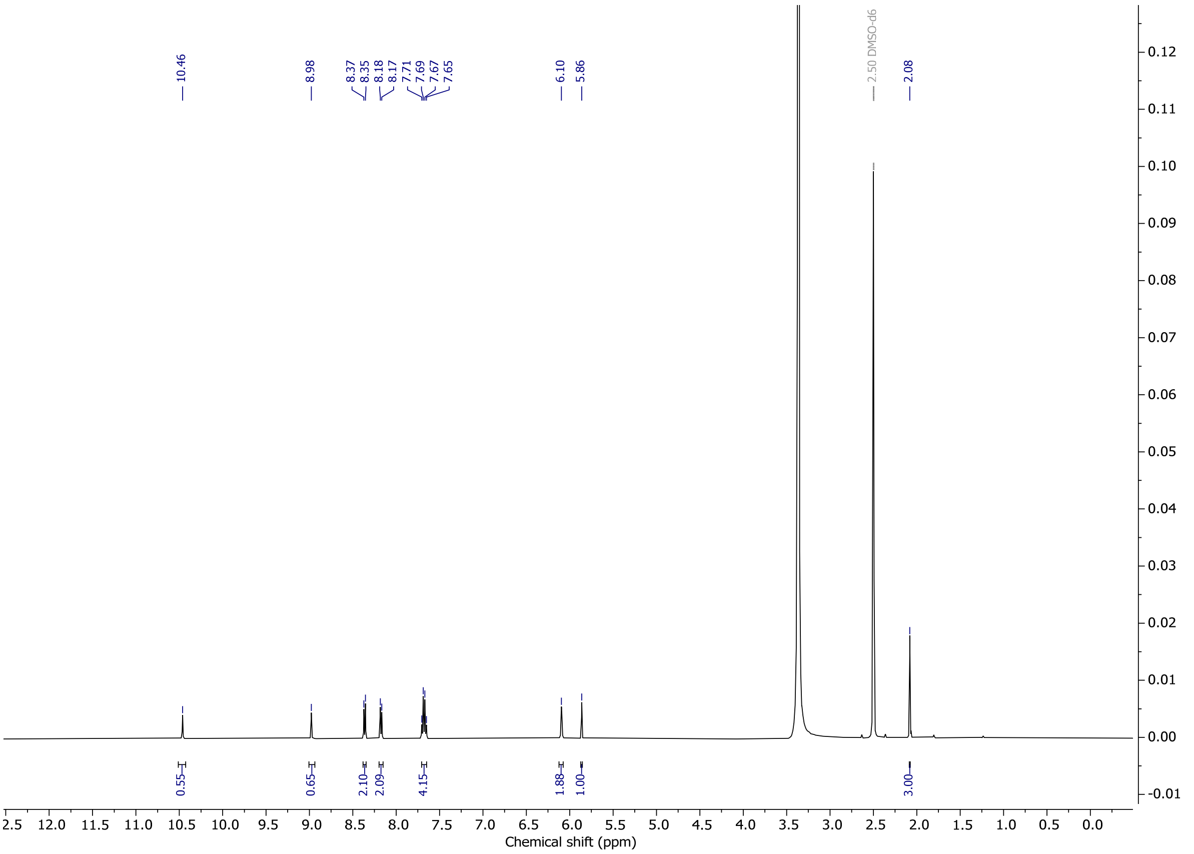

N-(4-((2-Amino-6-methylpyrimidin-4-yl)amino)phenyl)benzamide (23)

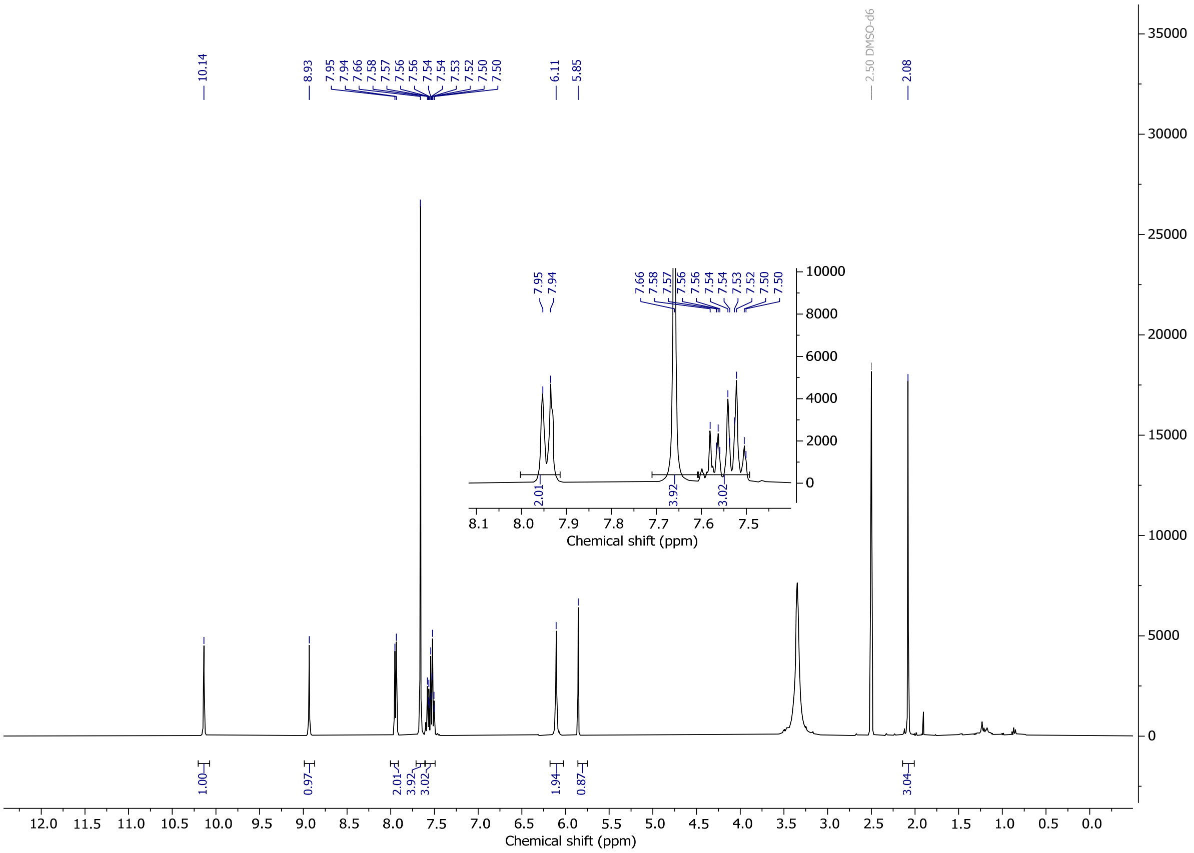

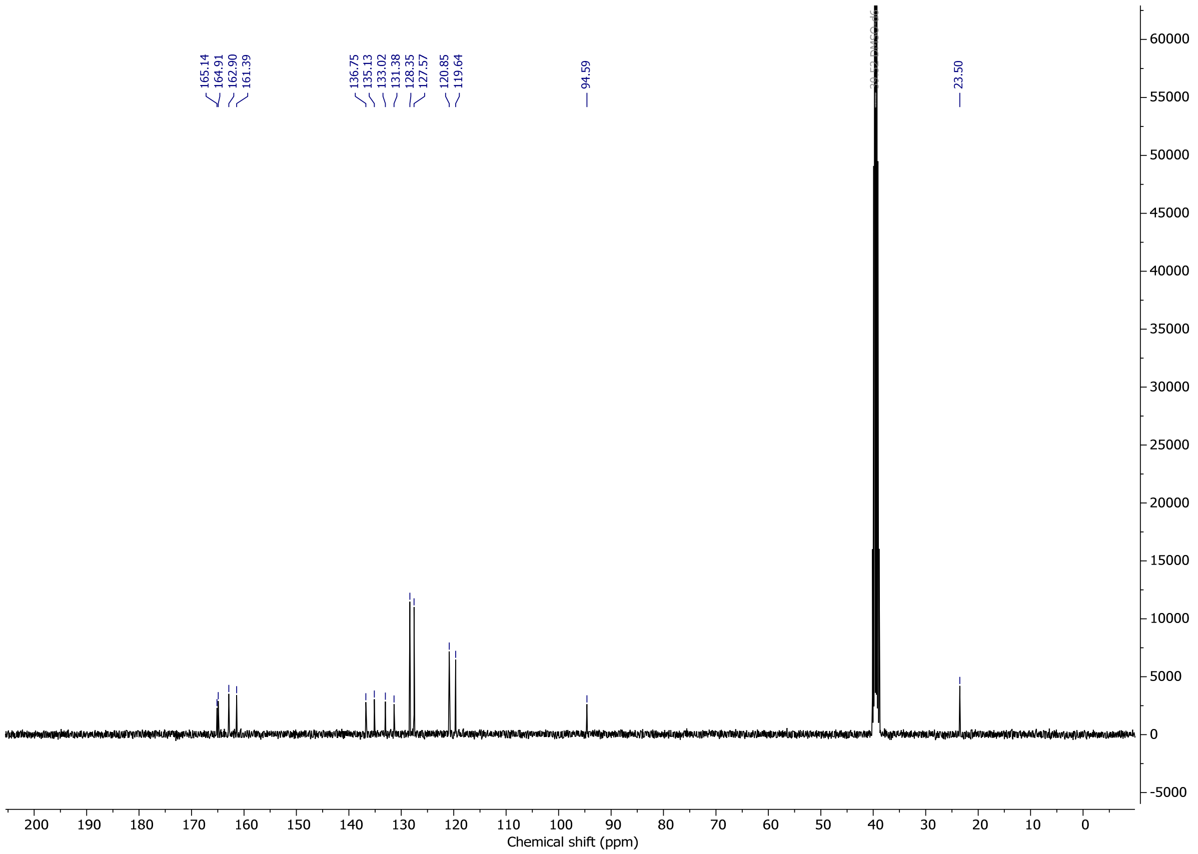

4-((4-((2-Amino-6-methylpyrimidin-4-yl)amino)phenyl)carbamoyl)benzenaminium (25)

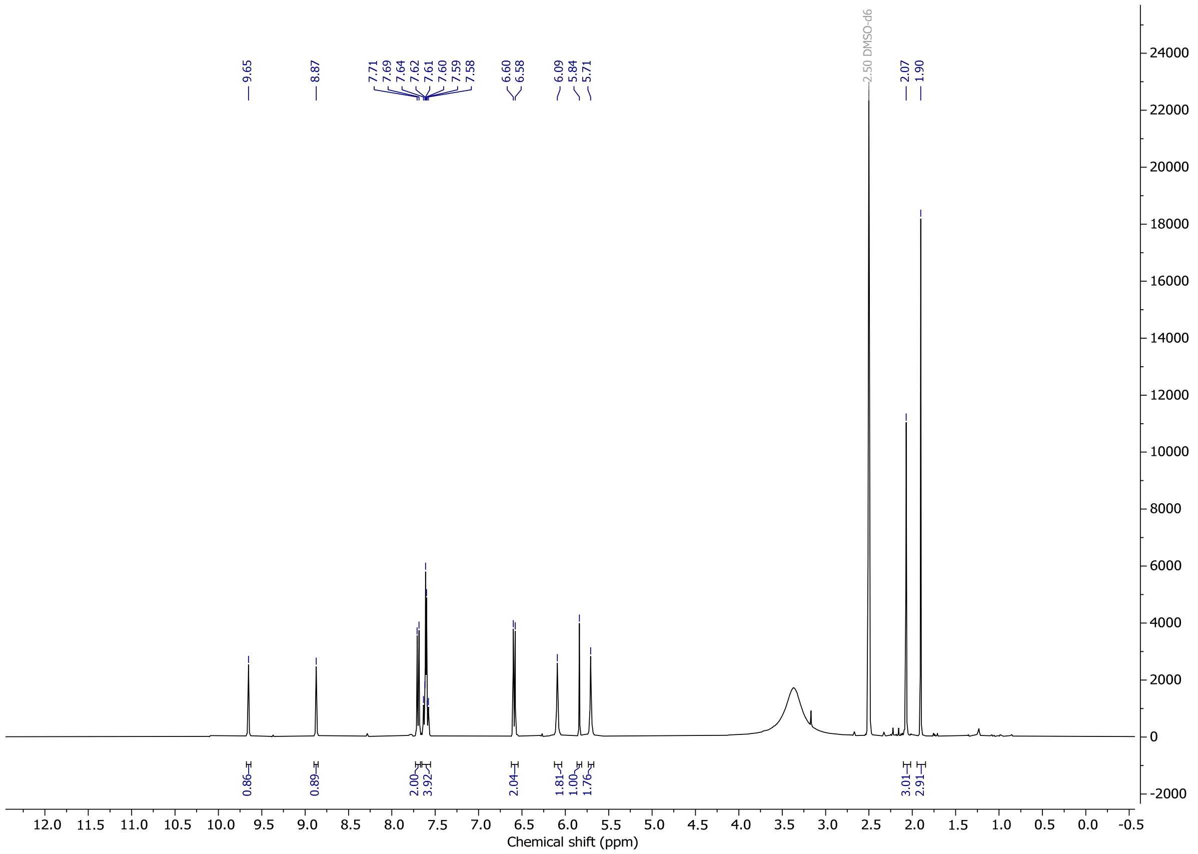

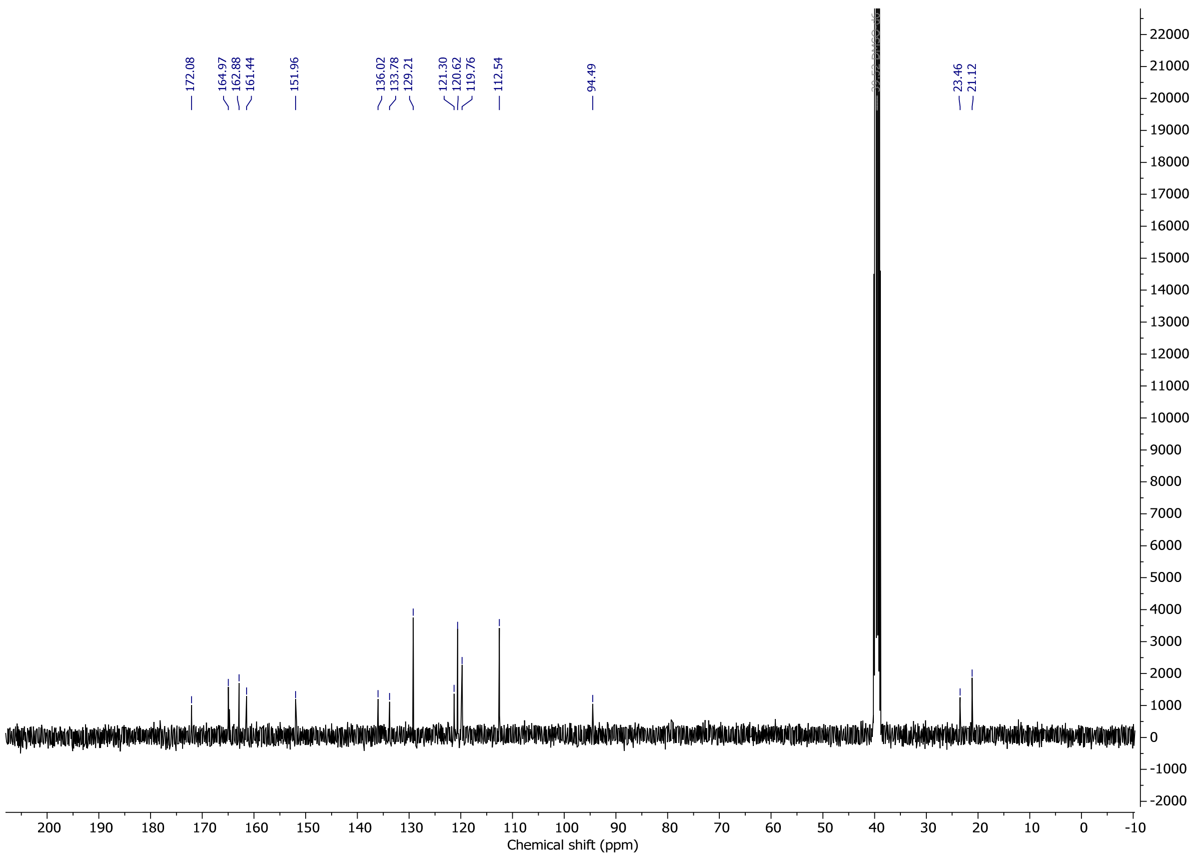
172.08
164.97
162.88
161.44
151.96
136.02
133.78
129.21
121.30
120.62
119.76
112.54
94.49
23.46
21.12
Chemical shift (ppm)
